# Global disease burden of stroke attributable to high fasting plasma glucose in 204 countries and territories from 1990 to 2019: An analysis of the Global Burden of Disease Study

**DOI:** 10.1111/1753-0407.13299

**Published:** 2022-08-04

**Authors:** Yang Liu, Wenxin Wang, Xuewei Huang, Xingyuan Zhang, Lijin Lin, Juan‐Juan Qin, Fang Lei, Jingjing Cai, Bo Cheng

**Affiliations:** ^1^ Department of Stomatology Zhongnan Hospital of Wuhan University Wuhan China; ^2^ Institute of Model Animal, Wuhan University Wuhan China; ^3^ Department of Cardiology Renmin Hospital of Wuhan University Wuhan China; ^4^ School of Basic Medical Science Wuhan University Wuhan China; ^5^ Department of Cardiology The Third Xiangya Hospital, Central South University, Changsha Changsha China

**Keywords:** disability‐adjusted life years, global burden of disease, high fasting plasma glucose, stroke, 全球疾病负担, 伤残调整寿命年, 卒中, 高空腹血糖

## Abstract

**Background:**

High fasting plasma glucose (HFPG) is the leading risk factor contributing to the increase of stroke burden in the past three decades. However, the global distribution of stroke burden specifically attributable to HFPG was not studied in depth. Therefore, we analyzed the HFPG‐attributable burden in stroke and its subtypes in 204 countries and territories from 1990 to 2019.

**Methods:**

Detailed data on stroke burden attributable to HFPG were obtained from the Global Burden of Disease Study 2019. The numbers and age‐standardized rates of stroke disability‐adjusted life years (DALYs), deaths, years lived with disability, and years of life lost between 1990 and 2019 were estimated by age, sex, and region.

**Results:**

In 2019, the age‐standardized rate of DALYs (ASDR) of HFPG‐attributable stroke was 354.95 per 100 000 population, among which 49.0% was from ischemic stroke, 44.3% from intracerebral hemorrhage, and 6.6% from subarachnoid hemorrhage. The ASDRs of HFPG‐attributable stroke in lower sociodemographic index (SDI) regions surpassed those in higher SDI regions in the past three decades. Generally, the population aged over 50 years old accounted for 92% of stroke DALYs attributable to HFPG, and males are more susceptible to HFPG‐attributable stroke than females across their lifetime.

**Conclusions:**

Successful key population initiatives targeting HFPG may mitigate the stroke disease burden. Given the soaring population‐attributable fractions of HFPG for stroke burden worldwide, each country should assess its disease burden and determine targeted prevention and control strategies.

## Graphical abstract

- During the past 30 years, globally, the disease burden of stroke burden has decreased substantially; however, the disease burden of stroke attributable to high fasting plasma glucose (HFPG) failed to achieve a significant decline. The population‐attributable fraction of HFPG for stroke burden has been increasing across different regions regardless of the sociodemographic index (SDI) of each region.
- The age‐standardized rate of disability‐adjusted life years (DALYs) of HFPG‐attributable stroke burden shifted from higher SDI regions to lower SDI regions.
- Older adults and males were the key populations concerning the control of HFPG‐attributable stroke in all SDI regions and all stroke subtypes.
- The increase of HFPG‐attributable stroke burden in females in low SDI regions and the slower decrease of DALYs of HFPG‐attributable stroke rates among 25–69 years old adults in high SDI regions compared with high‐middle SDI regions are worth attention as well.

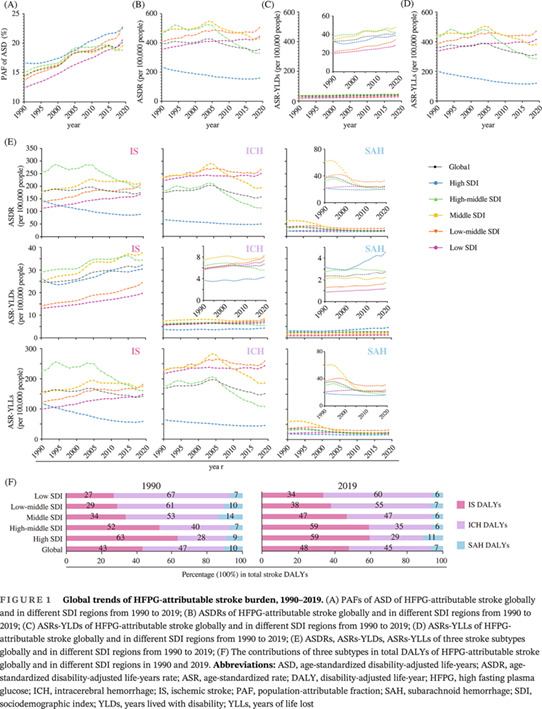

## 摘要

**背景:**

在过去30年中, 空腹血糖升高(HFPG)是导致卒中负担增加的主要危险因素。然而, HFPG导致的卒中负担的全球分布并未得到深入研究。因此, 我们分析了从1990年到2019年204个国家和地区卒中及其亚型的HFPG归因负担。

**方法:**

从2019年全球疾病负担研究中获得可归因于HFPG的中风负担的详细数据。按年龄、性别和地区估计1990年至2019年期间中风残疾调整寿命年(DALY)、死亡数、残疾生存年数和寿命损失年的数量和年龄标化率。

**结果:**

2019年HFPG归因性卒中的DALY年龄标化率(ASDR)为354.95/10万, 其中缺血性卒中占49.0%, 脑出血占44.3%, 蛛网膜下腔出血占6.6%。在过去的30年里, 社会人口统计指数(SDI)较低地区HFPG归因性卒中的ASDR超过了SDI较高地区。一般来说, 50岁以上的人群占HFPG导致的卒中DALY的92%, 男性在一生中比女性更容易患HFPG所致的中风。

**结论:**

针对HFPG的关键人群预防计划可能会减轻中风疾病的负担。鉴于世界范围内卒中负担可归因于HFPG的人口比例飙升, 每个国家都应评估其疾病负担, 并确定有针对性的预防和控制战略。

## 1 INTRODUCTION

Stroke is the second leading cause of death and the third leading cause of disability worldwide.^1^ In the past two decades, the annual prevalence of stroke and its related deaths increased substantially. By 2019, there were 101 million people suffering from stroke and 6.55 million deaths due to stroke globally.^1^ The large increase in the global burden of stroke was partly attributed to population aging and expansion.^2^ Meanwhile, because urbanization and lifestyles are shifting, there are increasing exposure rates to many metabolic risk factors and diseases.^1^, ^3^ Diabetes is one metabolic disease rising rapidly worldwide, with its highest growth rate in low‐ and middle‐income countries.^4^, ^5^ Studies have shown that hyperglycemia is one of the most critical risk factors for stroke and also has a major impact on its long‐term prognosis.^6^, ^7^, ^8^, ^9^ A recent systematic analysis for the Global Burden of Disease (GBD) Study 2019 revealed that high fasting plasma glucose (HFPG) was one of the leading risk factors contributing to the increase in the total number of stroke‐related disability‐adjusted life‐years (DALYs) in the past two decades, but the disease awareness of HFPG is lacking compared to other leading risk factors such as high systolic blood pressure and high body mass index.^1^, ^10^, ^11^ A previous meta‐analysis also showed that individuals with diabetes had increased risks of ischemic stroke (IS) and intracerebral hemorrhage stroke (ICH), with hazard ratios (HR) of 2.27 and 1.56, respectively.^12^ Because of the increasing and pressing stroke burden from uncontrolled glycemic levels and diabetes, it is imperative to conduct a granular analysis on assessing the contribution of HFPG to stroke and its subtypes. The GBD methodology framework and analytical strategies were well accepted to assess the disease burden on a global scale.^1^, ^2^, ^3^, ^13^ Thus, by adopting the approach for estimation of risk factor associated disease burden in the GBD analytic framework, we focused on analyzing the changing patterns of HFPG‐attributable stroke‐associated disease burden in different regions, age groups, and two sexes, aiming to provide essential information for making targeted strategies for the primary prevention of stroke and its subtypes.

## 2 METHODS

### 2.1 Data source

Annual data of ASRs (age‐standardized rates)and numbers of DALYs, deaths, years lived with disability (YLDs), and years of life lost (YLLs) due to HFPG‐attributable stroke burden were obtained from the Global Health Data Exchange GBD Results Tool (http://ghdx.healthdata.org/gbd-results-tool), which was established by GBD collaborators to provide a systematic updated assessment of age‐ and sex‐specific epidemiological data for 369 diseases and injuries and 87 risk factors in 204 countries and territories. According to the sociodemographic index (SDI), which is calculated by integrating the lagging average income per person, education level, and total fertility rate, 204 counties and territories were grouped into five SDI quintiles: high (>0.81), high‐middle (0.70–0.81), middle (0.61–0.69), low‐middle (0.46–0.60), and low (<0.46).^14^ In addition, those countries and territories were also divided into 21 geographic regions based on their geographic contiguity.^14^

### 2.2 Definitions

Detailed diagnosis and estimation methods of GBD 2019 have been published previously.^1^ Stroke was defined according to World Health Organization criteria as rapid development of focal or global brain dysfunction without obvious cause other than vascular origin and lasting for more than 24 hours, which can lead to death when the condition is severe. GBD 2019 models acute strokes using only first‐ever incident events. It is considered an acute stroke within 28 days from the first stroke event, and a chronic stroke from 29 days (including the sequelae of acute strokes and all recurrent stroke events). Stroke consists of three pathological subtypes: IS, ICH, and subarachnoid hemorrhage (SAH).^15^Their corresponding International Classification of Diseases, Tenth Revision codes are G45‐G46.8, I60‐I63.9, I65‐I66.9, I67.0‐I67.3, I67.5‐I67.6, I68.1‐I68.2, and I69.0‐I69.3.^1^ Transient ischemic attacks were not included for estimation.^2^ HFPG was defined as any level of FPG above the theoretical minimum‐risk exposure levels (TMREL), which is 4.8–5.4 mmol/L in GBD study.^16^

### 2.3 Attributable burden estimation

The estimation and analysis methods for risk‐attributable disease burden are available elsewhere.^1^ In brief, based on published systematic reviews and meta‐regression, the relative risk between risk and outcome was first estimated. Similarly, based on the exposure data of each risk factor released in the large population‐based survey or report, a Bayesian meta‐regression model (DisMod‐MR 2.1) and a spatiotemporal Gaussian process regression model were used to pool the data and determine the exposure level of risk and its TMREL. The attributable proportions of age‐standardized mortality rates compared by age, sex, year, and location were evaluated by population‐attributable fractions (PAFs), which presented the age‐standardized mortality rates that could decrease if the exposure to HFPG was eliminated to an alternative ideal situation. The equation of PAF for HFPG is defined as follows: ${\mathrm{PAF}}_{\text{oasgt}}=\left(\int \begin{array}{l} u\\ x=l \end{array}{\mathrm{RR}}_{\text{oasg}}\left(x\right)P_{\mathrm{as}gt}\left(x\right)\mathrm{dx}-{\mathrm{RR}}_{\mathrm{oas}g}\left({\text{TMREL}}_{\mathrm{as}}\right)\right)/\int \begin{array}{l} u\\ x=l \end{array}{\mathrm{RR}}_{\mathrm{oas}g}\left(x\right)P_{\mathrm{as}gt}\left(x\right)\mathrm{dx}$.

Where RR_oasg_(x) is the relative risk as a function of exposure level (x) for HFPG, cause (o), age group (a), and sex (s). P_asgt_ (x) is the distribution of exposure of HFPG according to age group (a), sex (s), location (g), and year (t). Location (g) with the lowest level of observed exposure as l and the highest as u.^17^

DALYs were used to estimate the global disease burden of specific causes, consisting of the burden caused by YLDs (years lived with any short‐term or long‐term health loss weighted for severity by the disability weights) and YLLs (multiplying observed deaths among individuals of a specific age in the year of interest by the age‐specific reference life expectancy estimated using life table methods). A standard Cause of Death Ensemble model approach was used to calculate the cause‐specific mortality rate due to stroke. HFPG‐attributable stroke burden was calculated by multiplying PAFs with cause‐specific stroke DALYs, YLDs, YLLs, and deaths. In addition, 95% uncertainty intervals (UIs) for the estimates were calculated to reflect random and systematic statistical modeling errors.

### 2.4 Statistical analyses

The ASR (per 100 000 individuals) was calculated based on the following formula: $\mathrm{ASR}=\frac{\sum_{i=1}^{A}a_{i}w_{i}}{\sum_{i=1}^{A}w_{i}}\times 100,000$, where *a*_*i*_ denotes the *i* ^*th*^ age class and the number of persons (or weight) (*w*_*i*_) in the same age subgroup *i* of the chosen reference standard population. The value was then divided by the sum of the standard population weights.

Pearson's correlation was used to analyze the relationship between HFPG‐related stroke rate (or PAF) and SDI. Locally weighted scatter plot smoothing was used in the regression analysis to create a smooth line to visualize the relationship between variables. All statistical analyses were conducted in R 4.0.4 (R Core Team), and the result is considered statistically significant when the *p* value was less than .05.

## 3 RESULTS

### 3.1 The Age‐Standardized Rate of DALYs of HFPG‐attributable stroke in lower SDI regions surpassed those in higher SDI regions

**FIGURE 1:**
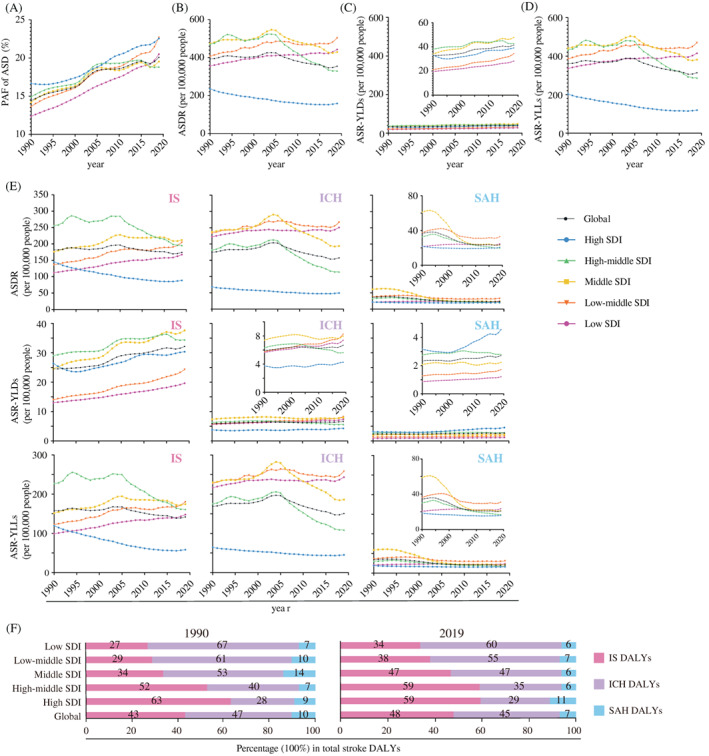
**Global trends of HFPG‐attributable stroke burden, 1990–2019.** (A) PAFs of ASD of HFPG‐attributable stroke globally and in different SDI regions from 1990 to 2019; (B) ASDRs of HFPG‐attributable stroke globally and in different SDI regions from 1990 to 2019; (C) ASRs‐YLDs of HFPG‐attributable stroke globally and in different SDI regions from 1990 to 2019; (D) ASRs‐YLLs of HFPG‐attributable stroke globally and in different SDI regions from 1990 to 2019; (E) ASDRs, ASRs‐YLDs, ASRs‐YLLs of three stroke subtypes globally and in different SDI regions from 1990 to 2019; (F) The contributions of three subtypes in total DALYs of HFPG‐attributable stroke globally and in different SDI regions in 1990 and 2019. **Abbreviations:** ASD, age‐standardized disability‐adjusted life‐years; ASDR, age‐standardized disability‐adjusted life‐years rate; ASR, age‐standardized rate; DALY, disability‐adjusted life‐year; HFPG, high fasting plasma glucose; ICH, intracerebral hemorrhage; IS, ischemic stroke; PAF, population‐attributable fraction; SAH, subarachnoid hemorrhage; SDI, sociodemographic index; YLDs, years lived with disability; YLLs, years of life lost

Global age‐standardized rate of DALYs (ASDRs) of HFPG‐attributable stroke decreased by 36.0% (Figure 1B). However, PAF of age‐standardized DALYs (ASDs) of stroke attributable to HFPG increased from 14.4% to 20.0% from 1990 to 2019 (Figure 1A). The majority of countries and territories experienced increased PAF of ASD of stroke attributable to HFPG (Table S1). ASDRs of HFPG‐attributable stroke consistently increased in low and low‐middle SDI regions and declined in middle, high‐middle, and high SDI regions in the past three decades (Figure 1B). Because the disease burden of DALYs is mainly contributed by YLLs, the global and regional trends and patterns of YLLs were similar to those of DALYs (Figure 1D). However, we noticed the global and regional YLDs of stroke attributable to HFPG constantly increased (Figure 1C). HFPG‐attributable mortalities of stroke globally and at country levels were similar to those of DALYs (Tables 1, 2 and S2, Figures S1 and S2). Therefore, DALYs and their rates of HFPG attributable‐stroke were selected to represent the major burden associated with stroke.

**TABLE 1:** ASRs of stroke burden attributable to HFPG in 1990 and 2019

Characteristic	Total Stroke			Ischemic stroke			Intracerebral hemorrhage			Subarachnoid hemorrhage		
	ASR (per 10^5^), Rate (95% UI)		Percentage change (×100%) (95% UI)	ASR (per 10^5^), Rate (95% UI)		Percentage change (×100%) (95% UI)	ASR (per 10^5^), Rate(95% UI)		Percentage change (×100%) (95% UI)	ASR (per 10^5^), Rate(95% UI)		Percentage change (×100%) (95% UI)
	1990	2019	1990–2019	1990	2019	1990–2019	1990	2019	1990–2019	1990	2019	1990–2019
**DALYs attribute to HFPG**												
Global	393.58 (269.56, 572.63)	354.95 (241.94, 512.10)	−0.10 (−0.18,‐0.01)	182.29 (95.92, 344.33)	173.97 (90.51, 316.30)	−0.05 (−0.13,0.05)	174.67 (112.13, 248.47)	157.42 (99.86, 227.19)	−0.10 (−0.20, 0.01)	36.63 (21.83, 54.75)	23.57 (14.85, 33.87)	−0.36 (−0.45,‐0.14)
Male	435.50 (299.46, 621.73)	416.31 (282.40, 608.20)	−0.04 (−0.15,0.07)	198.07 (104.30, 367)	198.43 (102.69, 362.11)	0.00 (−0.11,0.12)	200.92 (129.83, 287)	192.75 (118.52, 281.89)	−0.04 (−0.17, 0.10)	36.52 (18.71, 58.29)	25.12 (15.53, 36.79)	−0.31 (−0.46,0.25)
Female	357.27 (240.74, 527.48)	299.91 (204.74, 438.82)	−0.16 (−0.24,‐0.05)	168.76 (87.57, 325.81)	152.56 (79.45, 277.21)	−0.10 (−0.17,0.02)	151.91 (95.72, 217.98)	125.26 (79.30, 180.75)	−0.18 (−0.28, −0.04)	36.60 (22.28, 55.00)	22.09 (13.9, 32.39)	−0.40 (−0.49,‐0.29)
High SDI	235.84 (148.76, 380.57)	158.62 (106.07, 240.67)	−0.33 (−0.38,‐0.26)	145.83 (73.00, 286.26)	88.63 (46.11, 167.96)	−0.39 (−0.44,‐0.32)	68.19 (43.92, 96.33)	49.39 (32.13, 69.31)	−0.28 (−0.33, −0.21)	21.82 (13.99, 31.47)	20.60 (13.11, 28.82)	−0.06 (−0.15,0.06)
High‐middle SDI	470.72 (319.04, 698.84)	329.76 (215.96, 500.28)	−0.30 (−0.36,‐0.23)	255.95 (137.62, 470.58)	195.63 (103.58, 351.40)	−0.24 (−0.30,‐0.16)	181.87 (118.10, 261.27)	114.35 (74.48, 163.16)	−0.37 (−0.45, −0.29)	32.89 (20.64, 48.16)	19.78 (12.85, 27.83)	−0.40 (−0.50,‐0.17)
Middle SDI	474.76 (323.56, 674.57)	429.55 (288.98, 639.58)	−0.10 (−0.23,0.05)	176.25 (91.69, 333.93)	212.04 (107.78, 388.66)	0.20 (0.02,0.39)	237.59 (150.27, 344.15)	194.13 (120.76, 287.33)	−0.18 (−0.32, −0.06)	60.93 (33.83, 93.85)	23.39 (14.83, 33.84)	−0.62 (−0.71,‐0.39)
Low‐middle SDI	407.38 (279.80, 582.61)	503.76 (343.42, 722.73)	0.24 (0.07,0.42)	136.44 (70.68, 264.39)	204.54 (103.97, 379.48)	0.50 (0.27,0.75)	233.35 (145.79, 335.39)	266.57 (160.90, 392.13)	0.14 (−0.03, 0.34)	37.59 (18.28, 64.26)	32.65 (17.93, 51.34)	−0.13 (−0.31,0.27)
Low SDI	355.77 (240.60, 516.50)	442.86 (293.86, 640.79)	0.24 (0.08,0.44)	112.17 (56.50, 229.12)	167.39 (83.73, 320.01)	0.49 (0.27,0.75)	222.17 (136.49, 320.88)	250.79 (147.23, 368.53)	0.13 (−0.03, 0.33)	21.43 (7.40, 42.07)	24.68 (10.90, 45.35)	0.15 (−0.08,0.66)
**Deaths attribute to HFPG**												
Global	20.50 (13.14, 33.64)	17.72 (11.32, 28.82)	−0.14 (−0.21,‐0.05)	10.87 (5.28, 23.05)	9.56 (4.55, 20.78)	−0.12 (−0.20,‐0.04)	8.10 (5.05, 12.15)	7.24 (4.37, 11.04)	−0.11 (−0.21, 0.00)	1.53 (0.87, 2.39)	0.92 (0.56, 1.39)	−0.40 (−0.50,‐0.16)
Male	22.62 (14.5, 36.62)	20.85 (13.22, 34.08)	−0.08 (−0.18,0.03)	11.79 (5.76, 24.43)	11.03 (5.14, 23.36)	−0.06 (−0.17,0.04)	9.30 (5.86, 14.05)	8.81 (5.29, 13.69)	−0.05 (−0.18, 0.08)	1.53 (0.70, 2.50)	1.00 (0.61, 1.56)	−0.34 (−0.49,0.29)
Female	18.80 (11.81, 31.67)	15.07 (9.48, 24.98)	−0.20 (−0.28,‐0.10)	10.13 (4.86, 21.92)	8.33 (4, 18.42.00)	−0.18 (−0.25,‐0.07)	7.15 (4.30, 10.84)	5.89 (3.56, 9.01)	−0.18 (−0.29, −0.04)	1.52 (0.88, 2.43)	0.84 (0.52, 1.31)	−0.45 (−0.54,‐0.33)
High SDI	12.78 (7.30, 24.07)	7.54 (4.58, 13.39)	−0.41 (−0.47,‐0.34)	8.85 (4.09, 19.86)	4.53 (2.10, 10.17)	−0.49 (−0.54,‐0.43)	3.18 (1.94, 4.77)	2.29 (1.42, 3.59)	−0.28 (−0.34, −0.20)	0.75 (0.49, 1.10)	0.71 (0.46, 1.06)	−0.05 (−0.15,0.08)
High‐middle SDI	25.31 (15.82, 42.66)	17.44 (10.63, 30.23)	−0.31 (−0.38,‐0.23)	15.36 (7.80, 31.85)	11.26 (5.53, 23.76)	−0.27 (−0.34,‐0.19)	8.63 (5.35, 12.81)	5.43 (3.40, 8.30)	−0.37 (−0.45, −0.29)	1.32 (0.79, 2.02)	0.76 (0.49, 1.13)	−0.43 (−0.53,‐0.18)
Middle SDI	24.81 (15.90, 38.32)	22.17 (13.90, 36.35)	−0.11 (−0.25,0.05)	10.09 (4.82, 21.59)	11.66 (5.47, 25.23)	0.16 (−0.04,0.36)	11.80 (7.08, 18.12)	9.53 (5.55, 15.10)	−0.19 (−0.33, −0.06)	2.92 (1.57, 4.80)	0.98 (0.59, 1.52)	−0.66 (−0.75,‐0.45)
Low‐middle SDI	20.69 (13.35, 32.29)	25.27 (16.08, 39.84)	0.22 (0.05,0.41)	8.21 (3.84, 18.42)	11.73 (5.29, 24.87)	0.43 (0.21,0.69)	10.88 (6.69, 16.31)	12.25 (7.23, 19.16)	0.13 (−0.05, 0.31)	1.60 (0.74, 2.76)	1.30 (0.70, 2.14)	−0.19 (−0.35,0.20)
Low SDI	17.93 (11.42, 28.84)	22.16 (13.83, 34.57)	0.24 (0.07,0.42)	6.65 (2.95, 14.91)	9.51 (4.24, 20.10)	0.43 (0.21,0.69)	10.39 (5.95, 16.13)	11.65 (6.66, 18.12)	0.12 (−0.04, 0.31)	0.88 (0.28, 1.81)	0.99 (0.41, 1.94)	0.13 (−0.11,0.65)
**YLDs attribute to HFPG**												
Global	32.74 (18.97, 53.11)	41.54 (23.95, 66.98)	0.27 (0.20,0.35)	24.51 (12.91, 43.84)	32.13 (16.62, 55.62)	0.31 (0.24,0.40)	5.89 (3.36, 9.02)	6.68 (3.82, 10.29)	0.13 (0.07, 0.21)	2.33 (1.35, 3.52)	2.72 (1.61, 4.17)	0.17 (0.10,0.25)
Male	28.34 (16.60, 44.90)	37.98 (21.91, 61.49)	0.34 (0.25,0.44)	20.72 (10.97, 36.11)	28.86 (14.74, 51.11)	0.39 (0.30,0.49)	5.92 (3.37, 9.10)	7.06 (4.03, 10.94)	0.19 (0.11, 0.29)	1.70 (0.98, 2.66)	2.05 (1.18, 3.22)	0.21 (0.12,0.31)
Female	36.14 (20.64, 58.84)	44.48 (25.61, 71.63)	0.23 (0.16,0.32)	27.41 (14.32, 49.29)	34.87 (18.13, 59.76)	0.27 (0.20,0.37)	5.85 (3.34, 8.96)	6.31 (3.63, 9.67)	0.08 (0.01, 0.15)	2.87 (1.67, 4.35)	3.31 (1.94, 5)	0.15 (0.08,0.24)
High SDI	33.29 (19.10, 55.01)	39.18 (22.67, 63.06)	0.18 (0.10,0.28)	26.35 (13.71, 46.38)	30.36 (15.81, 52.28)	0.15 (0.07,0.26)	3.77 (2.19, 5.72)	4.27 (2.54, 6.52)	0.13 (0.05, 0.23)	3.17 (1.86, 4.81)	4.56 (2.67, 6.91)	0.44 (0.31,0.58)
High‐middle SDI	38.10 (22.26, 60.98)	42.85 (24.99, 68.26)	0.12 (0.05,0.22)	29.04 (15.39, 50.16)	34.42 (18.08, 58.03)	0.19 (0.10,0.28)	6.31 (3.59, 9.65)	5.63 (3.22, 8.70)	−0.11 (−0.17, −0.04)	2.74 (1.60, 4.22)	2.81 (1.66, 4.29)	0.02 (−0.05,0.11)
Middle SDI	34.01 (19.60, 55.17)	47.79 (26.84, 78.83)	0.40 (0.28,0.54)	24.55 (12.73, 44.12)	37.60 (19.13, 65.23)	0.53 (0.41,0.68)	7.40 (4.13, 11.40)	7.96 (4.51, 12.25)	0.08 (0.01, 0.16)	2.07 (1.18, 3.18)	2.22 (1.28, 3.45)	0.08 (0.00,0.17)
Low‐middle SDI	21.06 (12.17, 33.71)	34.30 (20.00, 54.64)	0.63 (0.49,0.79)	14.03 (7.17, 25.13)	24.34 (12.67, 42.48)	0.73 (0.58,0.91)	5.76 (3.31, 8.88)	8.25 (4.61, 12.82)	0.43 (0.30, 0.60)	1.27 (0.70, 2.02)	1.71 (0.94, 2.72)	0.35 (0.23,0.49)
Low SDI	19.52 (11.30, 32.21)	28.24 (16.46, 45.02)	0.45 (0.31,0.61)	13.00 (6.79, 23.72)	19.66 (10.10, 34.90)	0.51 (0.35,0.69)	5.65 (3.21, 8.75)	7.37 (4.03, 11.48)	0.30 (0.17, 0.47)	0.87 (0.49, 1.41)	1.21 (0.67, 1.96)	0.39 (0.25,0.57)
**YLLs attribute to HFPG**												
Global	360.84 (247.18, 523.50)	313.42 (214.33, 454.80)	−0.13 (−0.21,‐0.04)	157.77 (82.59, 305.88)	141.84 (72.42, 263.57)	−0.10 (−0.19,0.00)	168.77 (108.75, 241.68)	150.74 (95.95, 218.02)	−0.11 (−0.21, 0.00)	34.30 (20.05, 51.23)	20.84 (13.09, 30.27)	−0.39 (−0.49,‐0.17)
Male	407.16 (281.06, 579.25)	378.33 (258.95, 556.51)	−0.07 (−0.18,0.05)	177.35 (93.26, 328.91)	169.57 (86.14, 314.89)	−0.04 (−0.16,0.07)	195.00 (126.53, 278.62)	185.69 (114.72, 272.18)	−0.05 (−0.18, 0.09)	34.82 (17.16, 55.85)	23.07 (14.17, 34.23)	−0.34 (−0.48,0.25)
Female	321.13 (217.68, 476.20)	255.43 (175.09, 368.58)	−0.20 (−0.29,‐0.09)	141.34 (72.92, 277.56)	117.70 (60.53, 222.27)	−0.17 (−0.25,‐0.05)	146.06 (91.79, 208.49)	118.95 (74.19, 172.64)	−0.19 (−0.30, −0.05)	33.73 (20.32, 51.33)	18.78 (11.86, 27.94)	−0.44 (−0.53,‐0.33)
High SDI	202.55 (129.77, 329.49)	119.44 (80.46, 183.89)	−0.41 (−0.47,‐0.34)	119.49 (58.84, 241.03)	58.27 (29.53, 119.98)	−0.51 (−0.56,‐0.46)	64.42 (41.4, 91.18)	45.13 (29.5, 63.51)	−0.30 (−0.36, −0.23)	18.65 (11.80, 26.88)	16.05 (10.43, 22.45)	−0.14 (−0.22,‐0.02)
High‐middle SDI	432.62 (295.46, 642.70)	286.91 (190.03, 436.39)	−0.34 (−0.40,‐0.26)	226.91 (120.66, 427.76)	161.21 (84.25, 297.40)	−0.29 (−0.36,‐0.21)	175.56 (114.27, 252.53)	108.72 (70.42, 154.74)	−0.38 (−0.46, −0.30)	30.15 (18.66, 44.39)	16.98 (11.12, 23.83)	−0.44 (−0.53,‐0.19)
Middle SDI	440.75 (299.70, 631.42)	381.76 (258.39, 564.49)	−0.13 (−0.27,0.01)	151.69 (77.21, 292.37)	174.43 (87.17, 329.16)	0.15 (−0.05,0.35)	230.19 (145.7, 335.58)	186.17 (114.39, 275.46)	−0.19 (−0.33, −0.07)	58.86 (32.13, 90.81)	21.16 (13.43, 30.90)	−0.64 (−0.73,‐0.41)
Low‐middle SDI	386.32 (264.10, 552.71)	469.46 (318.28, 675.49)	0.22 (0.05,0.41)	122.41 (61.45, 235.45)	180.20 (89.28, 341.8)	0.47 (0.24,0.75)	227.59 (141.7, 328.22)	258.32 (154.93, 380.25)	0.14 (−0.04, 0.34)	36.32 (17.01, 62.70)	30.94 (16.91, 49.25)	−0.15 (−0.32,0.27)
Low SDI	336.25 (227.19, 494.54)	414.62 (275.06, 598.94)	0.23 (0.07,0.43)	99.17 (48.02, 203.05)	147.73 (71.9, 289.22)	0.49 (0.25,0.77)	216.52 (133.12, 313.69)	243.42 (144.51, 359.05)	0.12 (−0.04, 0.33)	20.56 (6.57, 40.87)	23.47 (10.00, 43.75)	0.14 (−0.10,0.68)

**Abbreviations:** ASR, age‐standardized rate; DALYs, disability‐adjusted life‐years; HFPG, high fasting plasma glucose; SDI, sociodemographic index; UI, uncertainty interval; YLDs, years lived with disability; YLLs, years of life lost.

**TABLE 2:** Numbers of stroke burden attributable to HFPG in 1990 and 2019

	Total stroke			Ischemic stroke			Intracerebral hemorrhage			Subarachnoid hemorrhage		
Characteristic	Total numbers (95% UI) × 10^3^		Percentage change (×100%) (95% UI)	Total numbers (95% UI) × 10^3^		Percentage change (×100%) (95% UI)	Total numbers (95% UI) × 10^3^		Percentage change (×100%) (95% UI)	Total numbers (95% UI) × 10^3^		Percentage change (×100%) (95% UI)
	1990	2019	1990–2019	1990	2019	1990–2019	1990	2019	1990–2019	1990	2019	1990–2019
**DALYs attribute to HFPG**												
Global	15014.91 (10427.71, 21187.27)	28908.40 (19794.82, 41524.30)	0.93 (0.74,1.13)	6520.59 (3533.23, 12018.53)	13900.65 (7342.19, 24911.22)	1.13 (0.95,1.33)	6994.87 (4438.56, 9855.38)	13051.39 (8229.98, 18832.11)	0.87 (0.64,1.10)	1499.45 (888.51, 2267.94)	1956.35 (1229.08, 2812.22)	0.30 (0.12,0.75)
Male	7545.21 (5294.64, 10538.44)	15777.40 (10830.43, 22511.79)	1.09 (0.83,1.37)	3097.20 (1716.56, 5362.68)	7207.37 (3817.04, 13152.25)	1.33 (1.03,1.61)	3739.62 (2415.45, 5341.08)	7573.28 (4680.70, 10937.46)	1.03 (0.74,1.34)	708.38 (365.88, 1131.47)	996.75 (610.26, 1455.52)	0.41 (0.12,1.52)
Female	7469.70 (5090.31, 10819.35)	13130.99 (8958.66, 19235.56)	0.76 (0.57,0.97)	3423.39 (1789.93, 6397.74)	6693.28 (3485.98, 12165.76)	0.96 (0.79,1.18)	3255.25 (2044.18, 4676.41)	5478.11 (3470.37, 7914.74)	0.68 (0.46,0.97)	791.06 (480.36, 1184.66)	959.60 (600.04, 1407.72)	0.21 (0.04,0.44)
High SDI	2507.12 (1564.79, 4046.78)	3046.85 (1946.44, 4935.59)	0.22 (0.11,0.34)	1579.42 (780.09, 3096.22)	1810.40 (897.78, 3592.30)	0.15 (0.04,0.28)	709.61 (458.10, 1007.53)	887.64 (566.70, 1259.73)	0.25 (0.13,0.40)	218.09 (141.59, 312.73)	348.81 (226.52, 486.52)	0.60 (0.40,0.85)
High‐middle SDI	4852.90 (3342.03, 6982.57)	6689.11 (4399.31, 10108.60)	0.38 (0.23,0.55)	2537.15 (1384.87, 4522.42)	3966.48 (2109.60, 7064.72)	0.56 (0.40,0.78)	1956.01 (1266.37, 2790.82)	2327.11 (1524.38, 3320.26)	0.19 (0.04,0.35)	359.74 (225.23, 524.62)	395.52 (256.28, 556.99)	0.10 (−0.08,0.51)
Middle SDI	4548.50 (3126.83, 6416.35)	10294.17 (7078.53, 14673.41)	1.26 (0.91,1.67)	1527.31 (843.02, 2739.85)	4846.23 (2558.69, 8702.54)	2.17 (1.66,2.69)	2395.06 (1494.10, 3508.76)	4849.05 (3033.27, 7090.87)	1.02 (0.68,1.36)	626.13 (346.02, 943.61)	598.89 (376.89, 866.89)	−0.04 (−0.26,0.49)
Low‐middle SDI	2305.29 (1585.78, 3234.18)	6689.99 (4557.04, 9375.43)	1.90 (1.48,2.37)	663.08 (364.96, 1210.41)	2535.59 (1337.37, 4681.94)	2.82 (2.23,3.50)	1402.51 (874.65, 1988.79)	3682.64 (2231.29, 5379.09)	1.63 (1.22,2.12)	239.69 (115.93, 419.26)	471.77 (257.32, 745.20)	0.97 (0.57,1.83)
Low SDI	792.64 (541.21, 1127.83)	2168.22 (1480.64, 3053.19)	1.74 (1.36,2.18)	210.16 (112.25, 382.54)	733.64 (393.11, 1339.85)	2.49 (1.99,3.02)	527.34 (327.35, 749.40)	1294.69 (786.27, 1875.37)	1.46 (1.10,1.90)	55.15 (19.66, 109.23)	139.90 (64.16, 258.98)	1.54 (1.03,2.66)
**Deaths attribute to HFPG**												
Global	705.16 (465.24, 1098.37)	1389.77 (902.76, 2222.83)	0.97 (0.78,1.18)	345.50 (172.63, 705.68)	730.51 (345.26, 1526.07)	1.11 (0.90,1.33)	301.68 (191.53, 439.57)	585.25 (354.54, 890.82)	0.94 (0.70,1.20)	57.99 (33.83, 88.30)	74.00 (45.70, 110.07)	0.28 (0.07,0.78)
Male	335.01 (226.80, 489.91)	727.82 (474.59, 1131.95)	1.17 (0.90,1.47)	154.90 (79.51, 294.42)	364.00 (175.18, 719.85)	1.35 (1.02,1.66)	153.91 (97.01, 224.72)	326.58 (196.69, 502.51)	1.12 (0.81,1.44)	26.20 (12.50, 42.14)	37.24 (22.81, 56.60)	0.42 (0.10,1.78)
Female	370.16 (237.99, 605.42)	661.95 (415.67, 1098.12)	0.79 (0.58,1.01)	190.59 (91.45, 401.69)	366.51 (176.09, 811.31)	0.92 (0.70,1.16)	147.77 (89.10, 220.41)	258.67 (155.68, 396.14)	0.75 (0.50,1.06)	31.79 (18.44, 49.86)	36.77 (22.65, 57.23)	0.16 (−0.04,0.42)
High SDI	135.98 (77.44, 253.76)	164.53 (96.61, 303.70)	0.21 (0.08,0.36)	94.38 (43.73, 210.92)	104.91 (47.03, 244.56)	0.11 (−0.05,0.30)	33.84 (20.14, 50.90)	46.02 (27.91, 76.85)	0.36 (0.20,0.58)	7.76 (5.07, 11.33)	13.59 (8.36, 21.75)	0.75 (0.50,1.11)
High‐middle SDI	236.00 (153.06, 380.86)	346.88 (212.23, 591.69)	0.47 (0.28,0.70)	135.68 (68.87, 267.73)	222.03 (108.48, 460.84)	0.64 (0.42,0.91)	86.84 (54.84, 125.99)	109.69 (68.01, 167.20)	0.26 (0.10,0.48)	13.47 (8.17, 19.91)	15.17 (9.84, 22.39)	0.13 (−0.09,0.61)
Middle SDI	201.35 (132.77, 292.20)	479.59 (313.14, 737.58)	1.38 (0.98,1.83)	72.63 (35.89, 142.50)	238.01 (113.13, 466.83)	2.28 (1.68,2.89)	102.69 (63.41, 151.55)	218.60 (130.18, 337.18)	1.13 (0.76,1.51)	26.04 (14.01, 41.16)	22.99 (14.29, 34.56)	−0.12 (−0.33,0.43)
Low‐middle SDI	98.29 (66.88, 142.19)	303.75 (200.43, 449.19)	2.09 (1.61,2.63)	32.56 (16.04, 63.13)	129.44 (61.11, 256.09)	2.98 (2.30,3.75)	56.96 (35.76, 83.23)	157.02 (92.39, 239.58)	1.76 (1.33,2.27)	8.77 (4.14, 15.11)	17.29 (9.40, 27.72)	0.97 (0.57,1.88)
Low SDI	33.15 (22.20, 48.41)	94.10 (60.59, 138.40)	1.84 (1.44,2.30)	10.04 (4.74, 20.42)	35.68 (16.61, 71.29)	2.55 (1.99,3.14)	21.17 (13.02, 30.85)	53.50 (31.39, 79.33)	1.53 (1.17,1.96)	1.93 (0.62, 3.78)	4.92 (2.09, 9.15)	1.55 (1.00,2.78)
**YLDs attribute to HFPG**												
Global	1280.27 (757.71, 2033.96)	3400.93 (1965.52, 5463.96)	1.66 (1.47,1.84)	937.33 (495.66, 1644.26)	2615.75 (1361.43, 4546.25)	1.79 (1.60,1.98)	245.72 (139.11, 381.43)	559.23 (319.12, 856.83)	1.28 (1.13,1.46)	97.22 (55.66, 149.11)	225.95 (132.35, 346.16)	1.32 (1.13,1.56)
Male	510.93 (306.48, 799.93)	1459.83 (854.61, 2337.43)	1.86 (1.59,2.12)	358.08 (190.07, 607.63)	1091.91 (566.02, 1875.12)	2.05 (1.76,2.31)	118.13 (65.80, 184.32)	285.60 (162.59, 439.89)	1.42 (1.21,1.66)	34.73 (19.04, 56.01)	82.31 (46.34, 128.86)	1.37 (1.12,1.68)
Female	769.34 (445.63, 1234.75)	1941.10 (1113.46, 3130.90)	1.52 (1.36,1.71)	579.25 (300.41, 1033.50)	1523.84 (792.96, 2626.32)	1.63 (1.46,1.83)	127.60 (72.90, 197.91)	273.63 (157.91, 417.82)	1.14 (1.02,1.31)	62.49 (36.16, 94.78)	143.64 (83.45, 217.83)	1.30 (1.11,1.54)
High SDI	351.92 (199.17, 585.11)	726.35 (408.81, 1209.30)	1.06 (0.91,1.25)	281.62 (143.33, 499.75)	575.76 (283.91, 1026.96)	1.04 (0.89,1.25)	38.50 (22.58, 58.55)	72.43 (42.96, 110.80)	0.88 (0.72,1.07)	31.80 (18.62, 48.42)	78.16 (45.69, 118.34)	1.46 (1.17,1.81)
High‐middle SDI	409.08 (241.64, 654.07)	871.46 (503.00, 1388.62)	1.13 (0.94,1.35)	308.00 (163.92, 524.61)	701.48 (368.42, 1180.71)	1.28 (1.07,1.53)	70.56 (39.60, 108.06)	113.88 (64.37, 173.91)	0.61 (0.51,0.75)	30.52 (17.62, 47.61)	56.09 (32.84, 85.72)	0.84 (0.68,1.04)
Middle SDI	345.07 (201.60, 552.82)	1179.27 (675.35, 1926.69)	2.42 (2.03,2.81)	238.15 (124.30, 418.11)	910.09 (468.16, 1576.34)	2.82 (2.43,3.25)	83.42 (46.36, 132.09)	210.80 (119.64, 324.97)	1.53 (1.33,1.78)	23.49 (12.80, 37.20)	58.37 (32.96, 90.98)	1.48 (1.26,1.77)
Low‐middle SDI	127.32 (76.59, 201.60)	473.47 (276.90, 746.54)	2.72 (2.33,3.17)	80.20 (42.39, 136.94)	328.02 (169.42, 568.94)	3.09 (2.62,3.58)	38.21 (21.41, 60.67)	119.78 (67.36, 186.10)	2.13 (1.80,2.53)	8.90 (4.74, 14.94)	25.68 (13.83, 41.36)	1.88 (1.60,2.25)
Low SDI	46.24 (27.74, 72.11)	148.48 (89.44, 231.06)	2.21 (1.92,2.54)	28.91 (15.39, 51.31)	99.08 (51.71, 173.23)	2.43 (2.11,2.79)	14.87 (8.26, 23.38)	41.89 (23.38, 66.15)	1.82 (1.53,2.15)	2.46 (1.33, 4.18)	7.51 (4.05, 12.75)	2.05 (1.75,2.40)
**YLLs attribute to HFPG**												
Global	13734.64 (9569.64, 19362.55)	25507.47 (17508.43, 36678.63)	0.86 (0.67,1.07)	5583.26 (2967.97, 10274.56)	11284.90 (5791.75, 20743.91)	1.02 (0.81,1.23)	6749.15 (4298.33, 9551.81)	12492.16 (7903.06, 18021.34)	0.85 (0.62,1.09)	1402.22 (813.25, 2129.60)	1730.40 (1080.22, 2500.32)	0.23 (0.05,0.70)
Male	7034.28 (4912.38, 9804.22)	14317.57 (9856.73, 20604.78)	1.04 (0.77,1.32)	2739.13 (1496.19, 4787.28)	6115.46 (3163.17, 11124.92)	1.23 (0.91,1.53)	3621.50(2355.37, 5190.10)	7287.68 (4516.37, 10591.23)	1.01 (0.72,1.33)	673.65 (340.02, 1082.01)	914.44 (556.71, 1342.68)	0.36 (0.08,1.53)
Female	6700.36 (4587.10, 9616.46)	11189.89 (7650.36, 16172.32)	0.67 (0.48,0.90)	2844.14 (1478.71, 5471.60)	5169.44 (2658.99, 9776.94)	0.82 (0.62,1.06)	3127.65 (1961.06, 4460.76)	5204.49 (3248.53, 7560.67)	0.66 (0.43,0.95)	728.57 (439.99, 1109.51)	815.96 (512.89, 1206.30)	0.12 (−0.05,0.35)
High SDI	2155.20 (1347.94, 3517.53)	2320.50 (1506.06, 3758.84)	0.08 (−0.03,0.20)	1297.80 (635.67, 2590.33)	1234.64 (596.04, 2584.27)	−0.05 (−0.17,0.10)	671.11 (434.43, 952.93)	815.21 (519.10, 1161.47)	0.21 (0.09,0.37)	186.29 (119.66, 266.34)	270.65 (177.45, 378.64)	0.45 (0.27,0.69)
High‐middle SDI	4443.82 (3066.24, 6337.87)	5817.65 (3852.15, 8866.54)	0.31 (0.16,0.48)	2229.15 (1210.39, 3991.46)	3265.00 (1706.94, 5946.54)	0.46 (0.29,0.69)	1885.45 (1225.75, 2691.33)	2213.23 (1441.49, 3138.33)	0.17 (0.02,0.34)	329.22 (202.33, 483.52)	339.42 (223.43, 476.37)	0.03 (−0.15,0.47)
Middle SDI	4203.43 (2905.60, 6013.54)	9114.90 (6246.73, 13095.72)	1.17 (0.82,1.58)	1289.16 (691.51, 2323.37)	3936.13 (2005.44, 7239.62)	2.05 (1.48,2.61)	2311.64 (1441.14, 3390.67)	4638.24 (2884.53, 6881.92)	1.01 (0.66,1.35)	602.64 (326.78, 914.62)	540.52 (339.61, 778.92)	−0.10 (−0.31,0.41)
Low‐middle SDI	2177.97 (1482.80, 3069.94)	6216.52 (4188.58, 8793.84)	1.85 (1.43,2.33)	582.88 (315.16, 1064.35)	2207.57 (1147.61, 4067.80)	2.79 (2.14,3.50)	1364.30 (849.68, 1926.31)	3562.86 (2169.65, 5210.18)	1.61 (1.20,2.11)	230.79 (108.72, 405.93)	446.10 (244.52, 710.59)	0.93 (0.54,1.82)
Low SDI	746.40 (504.51, 1056.78)	2019.75 (1362.53, 2863.86)	1.71 (1.33,2.17)	181.25 (91.70, 330.79)	634.56 (331.78, 1185.08)	2.50 (1.93,3.10)	512.47 (318.37, 730.05)	1252.80 (760.89, 1808.16)	1.44 (1.08,1.89)	52.68 (17.37, 105.47)	132.38 (57.56, 249.43)	1.51 (1.00,2.71)

**Abbreviations:** DALYs, disability‐adjusted life‐years; HFPG, high fasting plasma glucose; SDI, sociodemographic index; UI, uncertainty interval; YLDs, years lived with disability; YLLs, years of life lost.

Generally, the maximum DALYs of stroke attributable to HFPG shifted from high‐middle SDI region to middle SDI region between 1990 and 2019. Meanwhile, the maximum ASDRs of stroke attributable to HFPG shifted from middle SDI region to low‐middle SDI region **(**Tables 1 and 2 **).** In 2019, globally, among 28.9 million (95% UI: 19.8–41.5) stroke DALYs attributable to HFPG, there were 48.1% that contributed to IS, 45.1% contributed to ICH, and the remaining 6.8% contributed to SAH (Table 2, Figure 1F). The overall ASDRs from HFPG was 354.95 (95% UI: 241.94–512.1) per 100 000 population, which consisted of 49.0% from IS, 44.3% from ICH, and 6.6% from SAH (Table 1). In the past three decades, ASDRs of HFPG‐attributable IS weighted the highest burden among stroke in middle, high‐middle, and high SDI regions **(**Table 1 **)**. However, ASDRs of HFPG‐attributable ICH were the highest among total strokes in low and low‐middle SDI regions **(**Figure 1E, Table 1 **)**. Furthermore, the greater ASDR of HFPG‐attributable IS shifted from high‐middle SDI region to middle and low‐middle SDI regions, whereas the greater ASDRs of HFPG‐attributable ICH remained at low‐middle and low SDI regions during the past three decades **(**Figure 1E, Table 1 **)**.

### 3.2 People older than 50 years were susceptible to the disease burden of stroke attributable to HFPG

**FIGURE 2:**
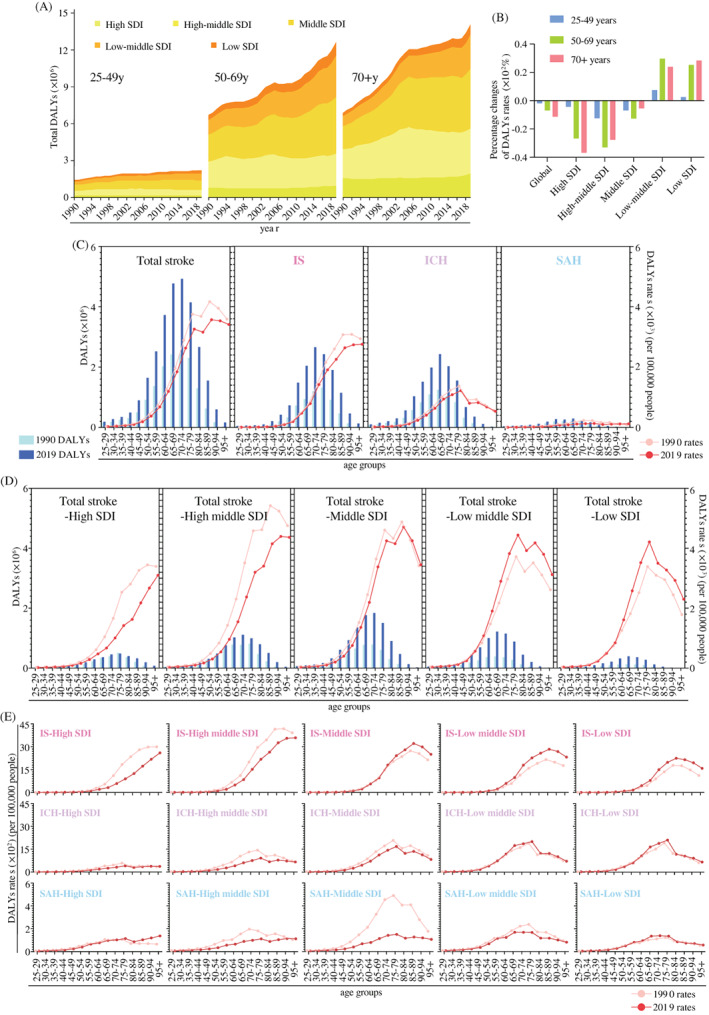
**Trends of DALYs of HFPG‐attributable stroke in different regions by age, 1990–2019.** (A) The total DALYs of HFPG‐attributable stroke in three age groups in different SDI regions from 1990 to 2019; (B) The percentage of changes in DALYs rates and of HFPG‐attributable stroke in three age groups globally and in different SDI regions from 1990 to 2019; (C) The DALYs rates and DALYs in 15 age groups of HFPG‐attributable stroke and three subtypes globally in 1990 and 2019; (D) The DALYs rates and DALYs in 15 age groups of HFPG‐attributable stroke in different SDI regions in 1990 and 2019; (E) The DALYs rates in 15 age groups of 3 HFPG‐attributable stroke subtypes in different SDI regions in 1990 and 2019. **Abbreviations:** DALY, disability‐adjusted life‐year; HFPG, high fasting plasma glucose; ICH, intracerebral hemorrhage; IS, ischemic stroke; SAH, subarachnoid hemorrhage; SDI, sociodemographic index

Global DALYs of stroke attributable to HFPG were accumulating in all SDI regions and age groups, with 28.9 million by 2019 (Table 2), and 92% were contributed by the population over 50 years old (Figure 2A, Table 2). The changes in DALYs rates of stroke attributable to HFPG were also the greatest among people over 50 years old in the past three decades (Figure 2B). Total DALYs of HFPG‐attributable stroke increased with aging before 70–74 years old and declined afterward globally (Figure 2C). The peaks of DALYs rates were at 85–89 age group globally, and the age group with the highest DALYs rates of stroke moved forward to the younger population along with SDI decrease (Figure 2C,D). For example, the highest DALYs rates were above 90 years old in high and high‐middle SDI regions, and the peaks moved forward to the 75–79 age group in low and low‐middle SDI regions (Figure 2D). Regarding stroke subtypes, IS was the dominant subtype of stroke among the population, and the peaks of DALYs rates in IS were consistent with those in all strokes. The peaks of DALYs rates in ICH and SAH were in 75–79 age group at the global level, and the peak remains at similar age groups across different SDI regions (Figure 2E). On the other hand, among the 25–69 years old age group, high SDI region showed a slower decrease of DALYs of HFPG‐attributable stroke rates when compared with high‐middle SDI region (Figure 2B).

### 3.3 Males were more susceptible to the disease burden of stroke attributable to HFPG

**FIGURE 3:**
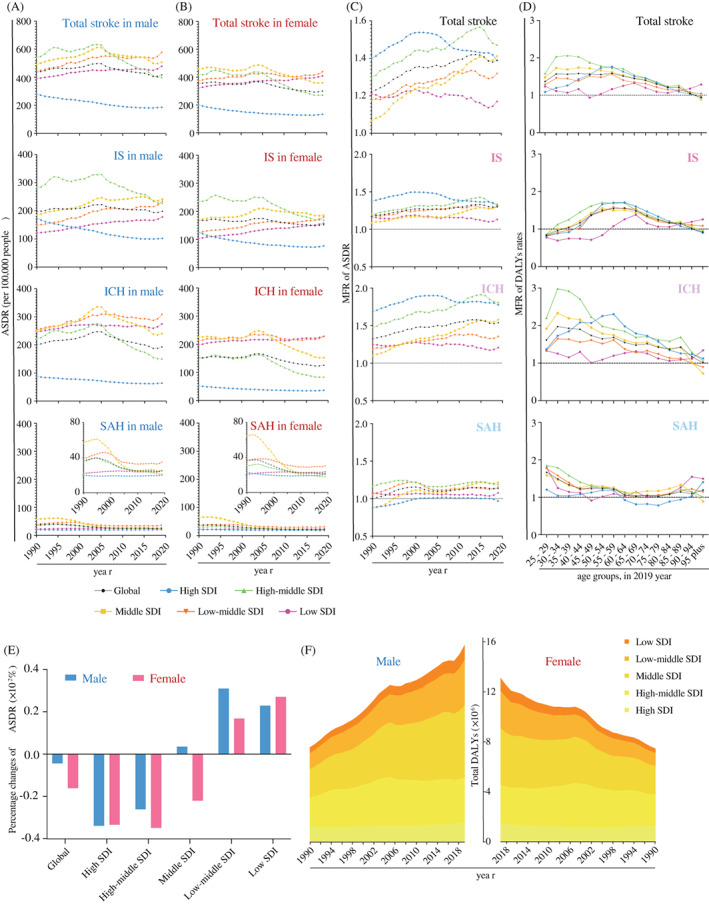
**Trends of DALYs of HFPG‐attributable stroke in different regions by sex, 1990–2019.** (A) The ASDRs of HFPG‐attributable stroke and its subtypes in males globally and in different SDI regions from 1990 to 2019; (B) The ASDRs of HFPG‐attributable stroke and its subtypes in females globally and in different SDI regions from 1990 to 2019; (C) The MFRs of ASDRs of HFPG‐attributable stroke and its subtypes globally and in different SDI regions from 1990 to 2019; (D) The MFRs of DALYs rates of HFPG‐attributable stroke and its subtypes in different age groups globally and in different SDI regions from 1990 to 2019; (E) The percentage of changes of HFPG‐attributable stroke ASDRs in males and females globally and in different SDI regions from 1990 to 2019; (F) The DALYs of HFPG‐attributable stroke in males and females globally and in different SDI regions from 1990 to 2019. **Abbreviations:** ASDR, age‐standardized disability‐adjusted life‐years rate; DALY, disability‐adjusted life‐year; HFPG, high fasting plasma glucose; ICH, intracerebral hemorrhage; IS, ischemic stroke; MFR, male to female ratio; SAH, subarachnoid hemorrhage; SDI, sociodemographic index

In the past 30 years, males have had higher ASDRs of HFPG‐attributable stroke than females in all SDI regions (Figure 3A–C). However, the sex differences in DALYs rate of stroke start diminishing at age 60, a few years after menopause in the female population (Figure 3D). Among all stroke subtypes, the largest burden gap between males and females in stroke ASDRs attributed to HFPG was ICH followed by IS (Figure 3C, Table 1). The global gap between males and females had widened slightly during the first two decades and leveled off in the last decade (Figure 3C,F). The differences in ASDRs of HFPG‐attributed stroke between sexes reached the highest of the past three decades among low‐middle to high‐middle SDI regions where most of the world's population lived, especially with regard to IS and ICH (Figure 3C). Furthermore, globally, the greatest proportion was in males among the 30–34 age groups because of HFPG‐attributable ICH and SAH, whereas among 55–69 age groups it was owing to HFPG‐attributable IS (Figure 3D). Interestingly, the sex difference was the lowest in low SDI regions regarding stroke ASDRs attributed to HFPG. The rate growth of ASDRs attributed to HFPG was even higher in females than in males from 1990 to 2019 (Figure 3E).

### 3.4 The association of SDI levels with HFPG exposure and disease burden of stroke attributable to HFPG

**FIGURE 4:**
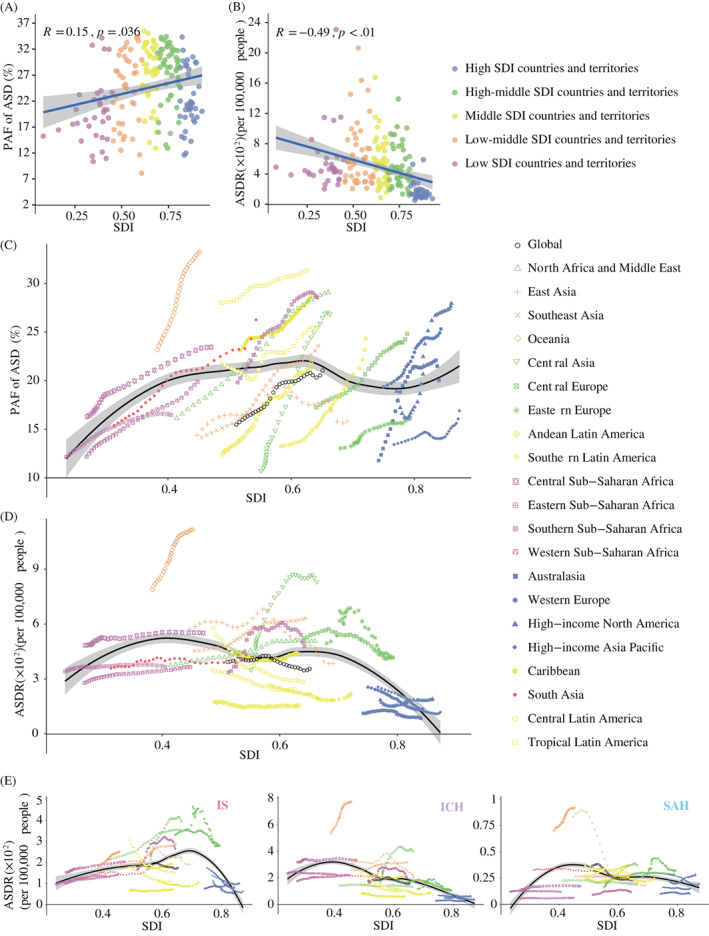
**The PAFs and rates of ASD of HFPG‐attributable stroke across 204 countries and territories and 21 GBD regions by SDI, 1990–2019.** (A) The PAFs of ASD of HFPG‐attributable stroke across 204 countries and territories from low SDI to high SDI in 2019; (B) The ASDRs of HFPG‐attributable stroke across 204 countries and territories from low SDI to high SDI in 2019; (C) The ASDRs of HFPG‐attributable stroke across 21 GBD regions from 1990 to 2019; (D) The PAFs of ASD of HFPG‐attributable stroke across 21 GBD regions from 1990 to 2019; (E)The ASDRs of 3 HFPG‐attributable stroke subtypes across 21 GBD regions from 1990 to 2019. **Abbreviations:** ASD, age‐standardized disability‐adjusted life‐years; ASDR, age‐standardized disability‐adjusted life‐years rate; GBD, Global Burden of Disease; HFPG, high fasting plasma glucose; IPAF, population‐attributable fraction; SDI, sociodemographic index

In 2019, the lowest ASDRs of stroke attributable to HFPG were observed in countries with high SDI regions (158.62 [95% UI: 106.07–240.67] DALYs per 100 000 people), and the highest stroke ASDRs attributable to HFPG were in regions with low‐middle SDI (503.76 [95% UI: 343.42–722.73] DALYs per 100 000 people) (Table 1, Table 2). In addition, positive relationships (R = 0.15, *p* < .05) between SDI levels and PAF of HFPG‐attributable ASDRs were detected (Figure 4A), whereas negative associations (R = −0.49, *p* < .01) were found between SDI levels and ASDRs of HFPG‐attributable stroke (Figure 4B).

In 21 GBD regions, all regions experienced wavily upward trends in PAF of HFPG‐attributable age‐standardized stroke DALYs from 1990 to 2019 (Figure 4C). In contrast, unlike the other regions with declined ASDR of HFPG attributable to stroke, the high‐income North American region, North Africa and Middle East, Central Asia, Southeast Asia, South Asia, and Oceania had seen a small increase in the ASDR of HFPG attributable to stroke in the past 20 years (Figure 4D, Table S3). For stroke subtypes, except in high SDI, the ASDR of IS and ICH both remained the lowest, the IS ASDRs attributable to HFPG increased with the SDI levels from low to high‐middle SDI, and the ICH ASDRs attributable to HFPG decreased with the SDI levels during the study period (Figure 4E).

## 4 DISCUSSION

Our study comprehensively analyzed the HFPG‐attributable disease burden in stroke and its subtypes from 1990 to 2019. The global ASDRs of stroke attributed to HFPG failed to achieve a significant decline, and the corresponding numbers of DALYs even nearly doubled. The HFPG‐attributable stroke burden shifted from higher SDI regions to lower SDI regions. Males almost have higher HFPG‐attributable stroke ASDRs than females across their lifetime, but low SDI region was an exception. The sex gap narrowed after menopause age in females. The population older than 50 years was still the most susceptible population to HFPG‐attributable stroke, and the peak of DALYs rates peak moved toward younger age with SDI decrease.

Many previous studies have demonstrated that HFPG is associated with the occurrence and poor prognosis of stroke.^6^, ^7^, ^8^, ^9^, ^18^, ^19^, ^20^ On the one hand, every modifiable risk factor including HFPG can independently affect the stroke disease burden^21^, ^22^, ^23^; On the other hand, the existence of other modifiable risk factors such as high body mass index can interact with HFPG to affect the stroke disease burden.^24^, ^25^, ^26^ GBD methodology framework has adjusted other risk factors and the interactions between them with HFPG.^1^ Thus, the results of this study reflect the independent effect of HFPG on stroke burden. Notably, in GBD 2019, the TMREL of FPG level is 4.8–5.4 mmol/L, and higher than that range will introduce much more stroke risk. Therefore, individuals with diabetes and prediabetes should pay attention to the increased risk of stroke disease burden.

HFPG disproportionately brings stroke burden to populations in different SDI regions. The HFPG‐attributable stroke burden in developing countries was significantly higher than in developed countries, and the burden gap was also expanding.^1^ This transition is largely attributed to increased exposure to metabolic factors during economic boom and urbanization in developing countries.^2^ Several reasons may explain the more rapid surge in metabolic disease in developing than developed countries. First, some studies claimed that nutritional deficiency in early life might increase the risk of insulin resistance and type 2 diabetes mellitus (DM) in adulthood.^27^ Second, the population in some developing countries, such as Asian countries, is more susceptible to metabolic stress than the European population.^28^, ^29^ Third, the population in developed countries has a higher awareness of control glucose and a better control rate in developing countries.^11^, ^12^, ^30^, ^31^ For instance, 52.3% of patients with DM had adequate glycemic control in the United States, whereas only 23.0% of patients had glucose well controlled in low‐ and middle‐income countries.^32^, ^33^ Therefore, the mounting risk of exposure to the high glucose level in developing countries increases the risk of stroke and other cardiovascular diseases. Moreover, advanced medical resources and treatment strategies, such as magnetic resonance angiography, diffusion‐weighted imaging, and interventional treatments, are more available in developed countries than in less developed countries.^34^, ^35^, ^36^, ^37^, ^38^ Stroke‐associated death and disability were more prevalent in regions with lower SDI.

Adults aged over 50 years were still the population with the heaviest HFPG‐related stroke burden because the elderly more frequently suffered from a higher prevalence of DM, longer duration of hyperglycemia, and more comorbidities.^39^, ^40^ There was a 15‐year age gap between the peaks of DALYs (70–74 age group) and the peaks of DALYs rates (85–89 age group), and this may be explained by the heavier burden from death and disability in the elderly population. Because IS is the dominant subtype of stroke among the population, the peaks of DALYs and DALYs rates in IS were constant with those in all strokes. The peaks of DALYs and DALYs rates in ICH and SAH were 5 and 10 years younger than those in IS at the global level. In different SDI regions, the age group with the highest DALYs rates moved forward to the younger population along with SDI decrease, implying the impacts of medication and blood glucose control on stroke‐associated death and population lifespan were significant. HFPG‐associated DALYs rates in all strokes declined from middle to high SDI regions in the past three decades. However, the rates kept increasing from age above 60 in low and low‐middle SDI regions, indicating HFPG‐attributable strokes were not well controlled in these groups. Another phenomenon that deserves special attention is that adults 25–69 years old in high SDI regions failed to achieve sufficient declines in HFPG‐attributable DALYs rate as those in high‐middle SDI regions, which corresponded with the rapidly increasing DM prevalence,^4^ suggested preventive strategies on glucose control need to be reinforced in the younger population in developed countries.

Males have a higher stroke burden from HFPG than females in all regions, and these gaps have increasingly wider in all regions, except for low SDI area. This information highlights a major issue that needs to be addressed in the comprehensive control of stroke. The prevalence of diabetes in men is significantly higher than in women and is growing at a more rapid speed.^4^, ^5^ In addition, other risk factors, such as smoking, hypertension, and dyslipidemia, are more predominant in males than in females, which may amplify the impact of HFPG on stroke and its outcomes.^41^, ^42^ Therefore, restraining the rising trend of stroke burden in males has to initiate a comprehensive strategy for primary control. Despite the higher burden of males, the potential stroke burden of females due to hyperglycemia could not be ignored, especially when the protective effect of estrogen on metabolism and cardiovascular system disappears after menopause, combined with a longer average life expectancy than males.^43^ We observed that the gap in disease burden of stroke between the males and females had narrowed since menopause (around 55 years old). It is noteworthy that a greater proportion of strokes are due to ICH and SAH in young males. HFPG‐attributable IS is more prevalent in males than females in the middle age group in all SDI regions. The sex difference in the frequencies of stroke subtypes during the entire life span indicated that HFPG‐attributable other forms of vascular pathologies besides ischemia. Interestingly, it is known that females are more prone to aneurysm rupture, with ICH and SAH 1.5 times more common in females.^44^, ^45^ However, HFPG‐attributable DALYs rates of ICH and SAH were more frequent in males in the young age group, as shown in our analysis. This discrepancy suggested that the higher prevalence of ICH and SAH in women may attribute to other etiologies, such as fibromuscular dysplasia, migraine, and Moyamoya disease.^46^, ^47^

In summary, the HFPG‐attributable disease burden of stroke was enormous globally. Particularly in lower SDI regions, the disease burden from HFPG was increasing rapidly with economic growth and population aging. In the past three decades, high SDI regions have made significant progress in mitigating HFPG‐attributable stroke, and other cardiovascular diseases, which suggested that increased public awareness and behavior management via community‐wide health intervention have pivotal roles in controlling HFPG‐attributable disease burden. Based on specific data on HFPG‐attributable disease burden at global and regional levels, each country should assess its disease burden and determine targeted strategic priorities to reduce the exposure to HFPG and subsequent disease burden of stroke.

### 4.1 Limitation

Our study provided an analysis of HFPG‐attributable stroke at a global scale, covering 30 years, laying a basis for evaluating the validity of geographical‐specific prevention and intervention over time. However, the present study is not free from the limitations of many previously published GBD studies. First, our study was carried out based on the GBD database; the results depended on the quality of original stroke epidemiological studies, and some important potential confounders (for example, atrial fibrillation and substance abuse), and different patterns of exposure (for instance, smoking duration) were not included for analysis.^1^ Moreover, high‐quality input data were more limited in lower SDI regions.^48^ Second, in the GBD 2019 framework, transient ischemic attack and other silent strokes were not included for analysis.^2^ Furthermore, apart from the three major subtypes of stroke, more detailed subcategories in each major stroke subtype that reflect different pathophysiological mechanisms were not assessed in the present study. For example, although atherosclerotic and thromboembolic strokes were both included in IS, we cannot tell them apart in GBD 2019. Third, methodologically, GBD studies deal with various situations individually in determining estimates of mortality and morbidity, which may lead to the overestimation of total disability in a population with multiple comorbidities.^48^ Last, estimation of the disease burden based on fasting glucose may lead to under‐diagnosis of the burden from individuals with postprandial hyperglycemia.

## 5 CONCLUSION

Regular updates of disease conditions within the GBD framework are profitable for researchers to assess disease burden timely. Effective prevention and control of HFPG have bright prospects to reduce stroke‐related disease burden. The HFPG‐attributable stroke burden shifted from higher SDI regions to lower SDI regions; thus, policymakers should start by addressing critical and urgent metabolic risk factors in the countries with low SDI, such as hypertension, obesity, and HFPG. Given the soaring PAF of HFPG for stroke burden globally regardless of SDI, each country should assess its disease burden and formulate targeted strategic priorities, and practitioners should accordingly advance the implementation of evidence‐based therapies as well as promote beneficial lifestyle practices.

## SOURCES OF FUNDING

This work was supported by grants from Hubei province unveiling science and technology projects: 2021BEC027, the National Science Foundation of China: 81870171 and 82170436.

## AUTHOR CONTRIBUTIONS

Yang Liu, Jingjing Cai, and Bo Cheng designed the study and wrote the manuscript, Wenxin Wang and Xuewei Huang collected and analyzed the data, Xingyuan Zhang and Lijin Lin performed the statistical analysis, and Juan‐Juan Qin and Fang Lei edited the manuscript and provided valuable suggestions for study design and data analysis. All authors have approved the final version of this paper.

## CONFLICT OF INTEREST

The authors declare no competing interests.

## Supporting information


**Appendix S1** Supporting Information
