# Supplementary material for: Global disease burden of stroke attributable to high fasting plasma glucose in 204 countries and territories from 1990 to 2019: An analysis of the Global Burden of Disease Study
Source: J Diabetes. 2022 Aug 4;14(8):495–513. doi: 10.1111/1753-0407.13299 (PMC9426282; doi:10.1111/1753-0407.13299)
Supplement: Supplementary file 1 — Appendix S1 Supporting Information [file JDB-14-495-s001.pdf]

SUPPLEMENTAL MATERIAL

Title: Global stroke burden attributable to high fasting plasma glucose in 204 countries and territories from 1990–2019

Content Page

Content	Page
Figure S1. The HFPG related PAFs and rates of age-standardized stroke death attributable to HFPG from 1990 to 2019.	1
Figure S2. Trends of HFPG-attributable stroke deaths in different regions by sex and age, 1990-2019.	2
Table S1. PAF of ASD of HFPG-attributable stroke in 1990 and 2019, by 204 countries and territories.	3
Table S2. Stroke burden (DALYs, deaths, YLDs, YLLs) attributable to HFPG in 1990 and 2019, by 204 countries and territories.	20
Table S3. 21 GBD regions and their country composition.	112

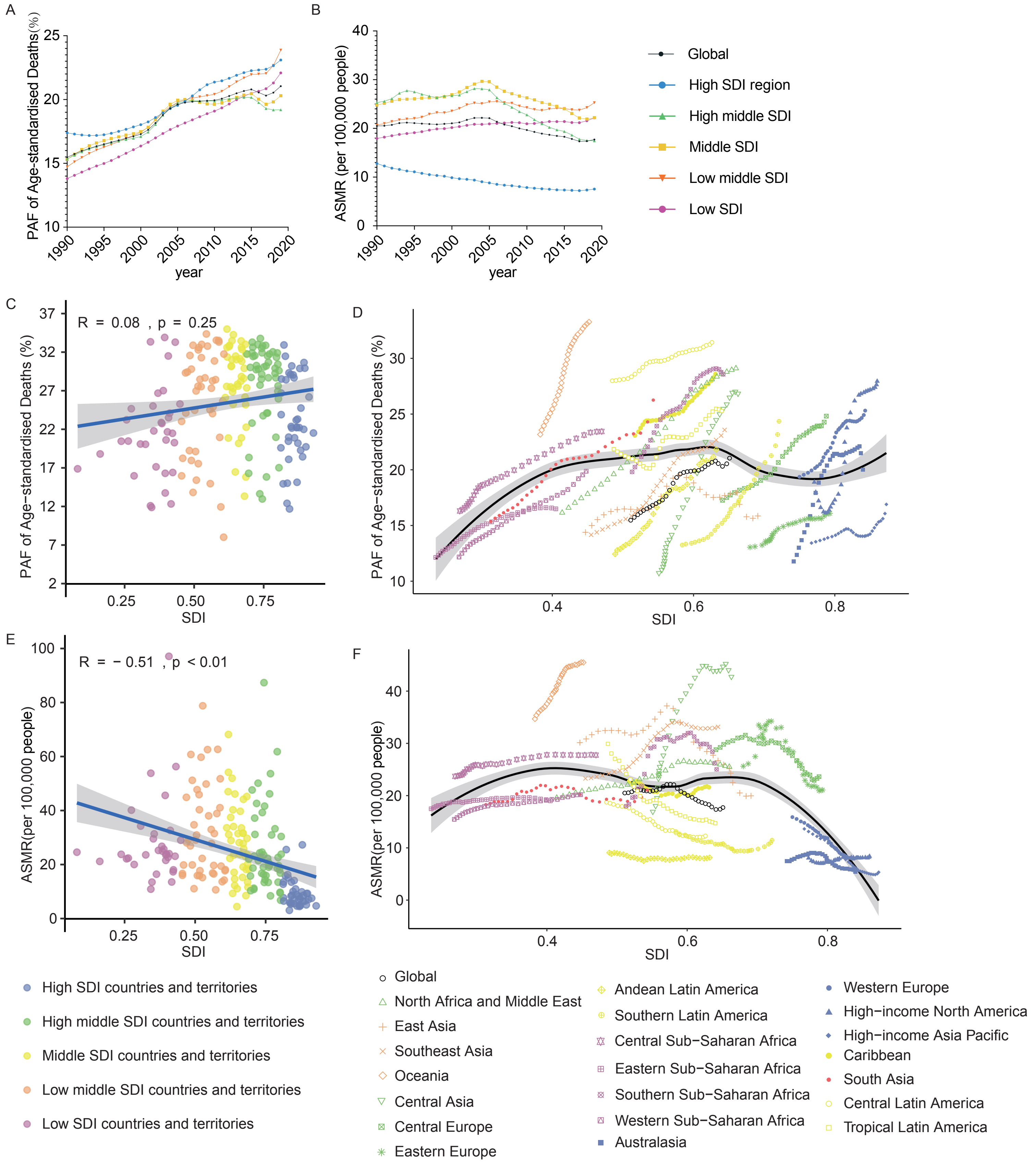

Figure S1. The HFPG related PAFs and rates of age-standardized stroke death attributable to HFPG from 1990 to 2019.

(A) The PAF of HFPG related age-standardized stroke deaths globally and in different SDI regions from 1990 to 2019;

(B) The ASMR of HFPG-attributable stroke globally and in different SDI regions from 1990 to 2019;

(C) The PAFs of HFPG related age-standardized stroke deaths across 204 countries and territories from low SDI to high SDI in 2019;

(D) The PAFs of HFPG related age-standardized stroke deaths across 21 GBD regions from 1990 to 2019;

(E) The ASMR of HFPG-attributable stroke across 204 countries and territories from low SDI to high SDI in 2019;

(F) The ASMR of HFPG-attributable stroke across 21 GBD regions from 1990 to 2019.

Abbreviations: ASMR, age-standardized mortality rate; HFPG, high fasting plasma glucose; PAF, population attributable fraction; SDI, sociodemographic index.

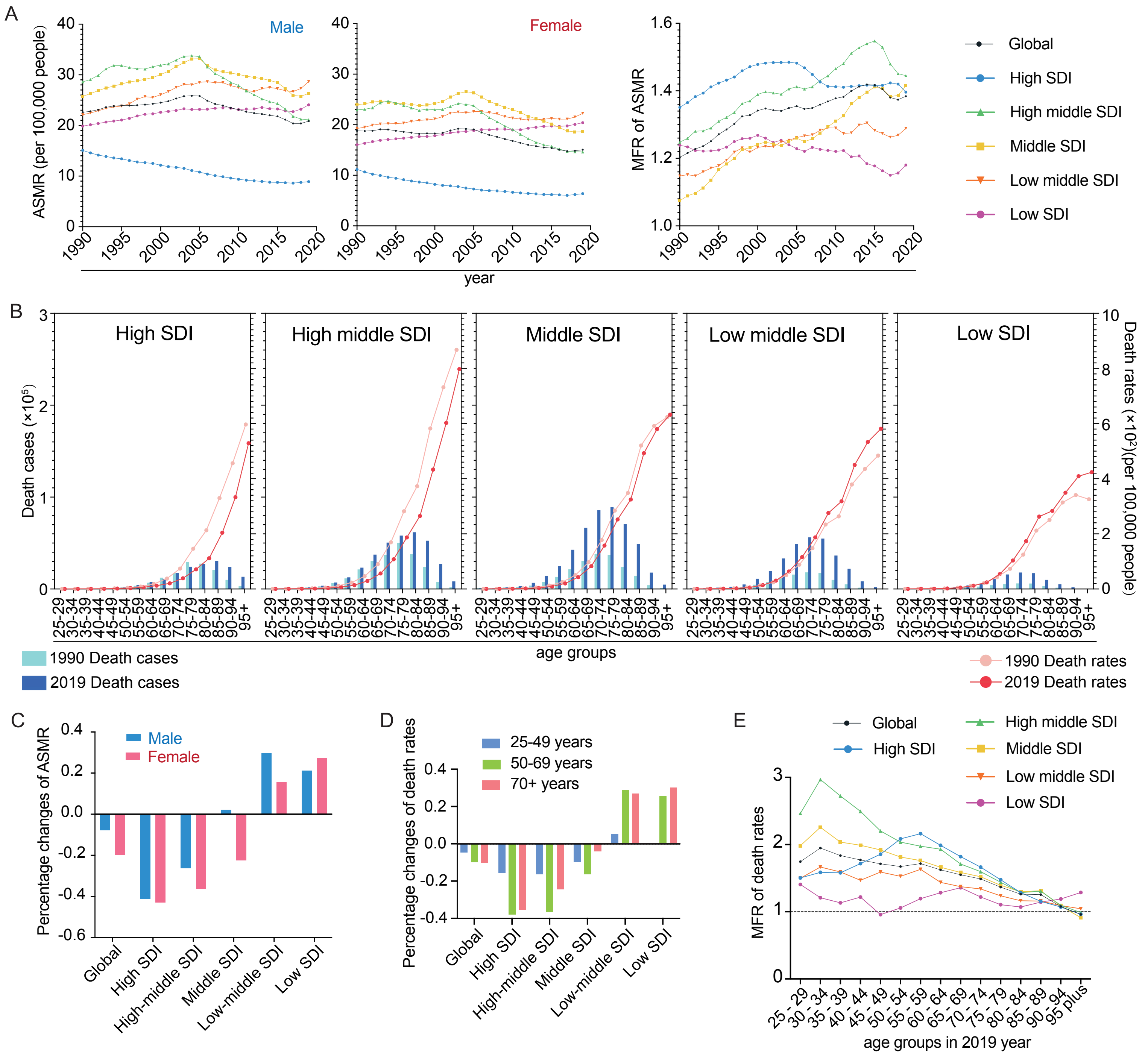

Figure S2. Trends of HFPG-attributable stroke deaths in different regions by sex and age, 1990-2019.

(A) The ASMRs of HFPG-attributable stroke in males and females, and the MFRs globally and in different SDI regions from 1990 to 2019;

(B) The death rates and death cases of HFPG-attributable stroke in 15 age groups in different SDI regions in 1990 and 2019;

(C) The percentage changes in death rates of HFPG-attributable stroke in 3 age groups globally and in different SDI regions from 1990 to 2019;

(D) The percentage changes in ASMRs of HFPG-attributable stroke in 3 age groups globally and in different SDI regions from 1990 to 2019;

(E) The MFRs of death rates of HFPG-attributable stroke in different age groups globally and in different SDI regions from 1990 to 2019.

Abbreviations: ASMR, age-standardized mortality rate; HFPG, high fasting plasma glucose; MFR, ratio of male to female; SDI, sociodemographic index.

Table S1. PAF of ASD of HFPG-attributable stroke in 1990 and 2019, by 204 countries and territories.

Characteristic	SDI	Total Stroke			Ischemic stroke			Intracerebral hemorrhage			Subarachnoid hemorrhage		
		PAF (95% UI) of ASD attributable to HFPG		Percentage change in PAF (95% UI)	PAF (95% UI) of ASD attributable to HFPG		Percentage change in PAF (95% UI)	PAF (95% UI) of ASD attributable to HFPG		Percentage change in PAF (95% UI)	PAF (95% UI) of ASD attributable to HFPG		Percentage change in PAF (95% UI)
		1990	2019	1990-2019	1990	2019	1990-2019	1990	2019	1990-2019	1990	2019	1990-2019
Afghanistan	0.343	0.18 (0.11,0.26)	0.33 (0.22,0.48)	0.87 (0.47,1.42)	0.20 (0.10,0.39)	0.35 (0.18,0.61)	0.71 (0.38,1.24)	0.16 (0.10,0.24)	0.31 (0.19,0.44)	0.90 (0.51,1.48)	0.16 (0.09,0.25)	0.30 (0.18,0.44)	0.91 (0.45,1.56)
Albania	0.681	0.11 (0.07,0.15)	0.13 (0.09,0.19)	0.22 (0.07,0.42)	0.11 (0.06,0.20)	0.14 (0.08,0.25)	0.24 (0.09,0.42)	0.11 (0.07,0.16)	0.13 (0.08,0.20)	0.21 (0.05,0.43)	0.09 (0.06,0.13)	0.11 (0.07,0.16)	0.22 (0.08,0.40)
Algeria	0.652	0.16 (0.10,0.27)	0.31 (0.19,0.51)	0.91 (0.48,1.48)	0.18 (0.09,0.36)	0.32 (0.17,0.60)	0.83 (0.41,1.40)	0.14 (0.09,0.21)	0.27 (0.18,0.39)	0.87 (0.49,1.33)	0.13 (0.08,0.19)	0.24 (0.16,0.35)	0.90 (0.51,1.44)
American Samoa	0.712	0.35 (0.24,0.47)	0.34 (0.24,0.46)	-0.03 (-0.19,0.15)	0.35 (0.20,0.63)	0.32 (0.20,0.55)	-0.08 (-0.23,0.13)	0.35 (0.22,0.50)	0.35 (0.23,0.49)	-0.02 (-0.20,0.19)	0.30 (0.18,0.43)	0.34 (0.20,0.51)	0.13 (-0.11,0.41)
Andorra	0.894	0.11 (0.07,0.18)	0.21 (0.12,0.35)	0.86 (0.48,1.31)	0.12 (0.06,0.23)	0.22 (0.10,0.47)	0.91 (0.46,1.42)	0.11 (0.07,0.16)	0.20 (0.12,0.32)	0.88 (0.44,1.41)	0.09 (0.06,0.13)	0.16 (0.10,0.23)	0.74 (0.36,1.25)
Angola	0.470	0.15 (0.10,0.23)	0.23 (0.15,0.34)	0.51 (0.20,0.95)	0.18 (0.09,0.37)	0.25 (0.12,0.50)	0.39 (0.09,0.87)	0.14 (0.09,0.22)	0.22 (0.12,0.34)	0.53 (0.21,0.96)	0.12 (0.07,0.18)	0.18 (0.10,0.26)	0.46 (0.14,0.88)
Antigua and Barbuda	0.743	0.25 (0.17,0.38)	0.32 (0.22,0.46)	0.29 (0.08,0.57)	0.29 (0.14,0.56)	0.33 (0.17,0.62)	0.17 (-0.01,0.44)	0.24 (0.14,0.37)	0.33 (0.20,0.48)	0.37 (0.12,0.72)	0.18 (0.11,0.27)	0.27 (0.16,0.38)	0.45 (0.18,0.81)
Argentina	0.708	0.12 (0.08,0.17)	0.21 (0.14,0.31)	0.71 (0.37,1.13)	0.14 (0.08,0.25)	0.24 (0.12,0.46)	0.74 (0.36,1.17)	0.12 (0.08,0.17)	0.21 (0.13,0.30)	0.71 (0.36,1.18)	0.10 (0.06,0.15)	0.16 (0.10,0.22)	0.56 (0.30,0.94)
Armenia	0.689	0.13 (0.08,0.23)	0.28 (0.16,0.46)	1.12 (0.58,1.95)	0.13 (0.06,0.27)	0.29 (0.14,0.57)	1.15 (0.59,1.98)	0.13 (0.07,0.21)	0.27 (0.15,0.40)	1.10 (0.55,1.90)	0.11 (0.07,0.18)	0.23 (0.13,0.35)	1.04 (0.52,1.84)

**Australia**	0.839	0.11<br>(0.06,0.19)	0.19<br>(0.11,0.34)	0.79<br>(0.41,1.22)	0.12<br>(0.06,0.25)	0.20<br>(0.09,0.47)	0.73<br>(0.33,1.17)	0.09<br>(0.06,0.14)	0.19<br>(0.11,0.31)	1.03<br>(0.57,1.53)	0.07<br>(0.04,0.10)	0.13<br>(0.08,0.19)	0.95<br>(0.58,1.45)
**Austria**	0.849	0.10<br>(0.06,0.17)	0.21<br>(0.12,0.38)	1.17<br>(0.72,1.66)	0.10<br>(0.05,0.21)	0.23<br>(0.11,0.49)	1.22<br>(0.73,1.72)	0.09<br>(0.06,0.13)	0.21<br>(0.12,0.34)	1.28<br>(0.73,1.91)	0.07<br>(0.05,0.11)	0.16<br>(0.10,0.24)	1.18<br>(0.71,1.85)
**Azerbaijan**	0.683	0.10<br>(0.07,0.14)	0.27<br>(0.17,0.41)	1.80<br>(1.12,2.53)	0.10<br>(0.06,0.18)	0.28<br>(0.14,0.58)	1.80<br>(1.05,2.68)	0.10<br>(0.06,0.14)	0.27<br>(0.15,0.42)	1.78<br>(1.08,2.61)	0.08<br>(0.05,0.13)	0.21<br>(0.12,0.32)	1.48<br>(0.84,2.32)
**Bahamas**	0.796	0.21<br>(0.14,0.31)	0.30<br>(0.20,0.42)	0.43<br>(0.17,0.78)	0.24<br>(0.12,0.45)	0.32<br>(0.17,0.59)	0.35<br>(0.11,0.71)	0.19<br>(0.12,0.30)	0.29<br>(0.18,0.42)	0.50<br>(0.21,0.89)	0.15<br>(0.09,0.22)	0.22<br>(0.14,0.32)	0.49<br>(0.20,0.84)
**Bahrain**	0.751	0.31<br>(0.20,0.48)	0.31<br>(0.20,0.48)	-0.02<br>(-0.17,0.15)	0.33<br>(0.17,0.63)	0.31<br>(0.17,0.57)	-0.06<br>(-0.21,0.14)	0.30<br>(0.18,0.44)	0.30<br>(0.20,0.43)	0.00<br>(-0.16,0.20)	0.19<br>(0.11,0.28)	0.24<br>(0.15,0.36)	0.29<br>(0.02,0.65)
**Bangladesh**	0.483	0.11<br>(0.08,0.16)	0.18<br>(0.11,0.27)	0.62<br>(0.34,1.06)	0.12<br>(0.07,0.24)	0.20<br>(0.10,0.42)	0.64<br>(0.31,1.11)	0.11<br>(0.07,0.15)	0.17<br>(0.10,0.26)	0.59<br>(0.33,0.98)	0.09<br>(0.06,0.14)	0.14<br>(0.09,0.20)	0.48<br>(0.27,0.80)
**Barbados**	0.742	0.22<br>(0.15,0.35)	0.27<br>(0.18,0.41)	0.23<br>(-0.00,0.54)	0.25<br>(0.13,0.49)	0.29<br>(0.15,0.52)	0.14<br>(-0.09,0.44)	0.20<br>(0.13,0.28)	0.27<br>(0.17,0.39)	0.35<br>(0.09,0.68)	0.16<br>(0.10,0.22)	0.21<br>(0.14,0.30)	0.36<br>(0.11,0.69)
**Belarus**	0.745	0.11<br>(0.07,0.16)	0.13<br>(0.09,0.20)	0.19<br>(0.07,0.36)	0.11<br>(0.06,0.20)	0.13<br>(0.08,0.23)	0.19<br>(0.05,0.37)	0.10<br>(0.07,0.15)	0.13<br>(0.08,0.18)	0.21<br>(0.06,0.37)	0.09<br>(0.06,0.14)	0.11<br>(0.07,0.17)	0.19<br>(0.05,0.37)
**Belgium**	0.851	0.12<br>(0.07,0.19)	0.21<br>(0.12,0.35)	0.77<br>(0.36,1.23)	0.13<br>(0.06,0.25)	0.23<br>(0.11,0.48)	0.80<br>(0.36,1.33)	0.11<br>(0.07,0.16)	0.20<br>(0.12,0.33)	0.85<br>(0.40,1.38)	0.09<br>(0.06,0.13)	0.16<br>(0.10,0.23)	0.77<br>(0.39,1.31)
**Belize**	0.603	0.14<br>(0.10,0.21)	0.24<br>(0.16,0.35)	0.72<br>(0.37,1.19)	0.16<br>(0.08,0.28)	0.27<br>(0.14,0.49)	0.70<br>(0.32,1.21)	0.14<br>(0.09,0.19)	0.23<br>(0.14,0.35)	0.72<br>(0.38,1.19)	0.11<br>(0.07,0.16)	0.19<br>(0.12,0.27)	0.75<br>(0.43,1.22)
**Benin**	0.352	0.11<br>(0.07,0.18)	0.22<br>(0.14,0.34)	1.00<br>(0.54,1.64)	0.13<br>(0.06,0.29)	0.26<br>(0.12,0.53)	0.92<br>(0.40,1.60)	0.10<br>(0.06,0.17)	0.21<br>(0.12,0.33)	1.03<br>(0.55,1.73)	0.08<br>(0.04,0.13)	0.14<br>(0.08,0.23)	0.84<br>(0.38,1.54)
**Bermuda**	0.813	0.15<br>(0.10,0.24)	0.22<br>(0.14,0.35)	0.44<br>(0.20,0.76)	0.16<br>(0.09,0.31)	0.24<br>(0.12,0.47)	0.45<br>(0.18,0.80)	0.14<br>(0.09,0.21)	0.21<br>(0.13,0.32)	0.46<br>(0.19,0.85)	0.12<br>(0.08,0.17)	0.17<br>(0.11,0.24)	0.42<br>(0.18,0.75)
**Bhutan**	0.455	0.11<br>(0.08,0.16)	0.22<br>(0.14,0.34)	0.93<br>(0.49,1.52)	0.12<br>(0.06,0.22)	0.24<br>(0.11,0.47)	0.97<br>(0.50,1.53)	0.11<br>(0.07,0.16)	0.21<br>(0.13,0.32)	0.88<br>(0.45,1.45)	0.10<br>(0.07,0.15)	0.17<br>(0.11,0.25)	0.69<br>(0.34,1.20)

**Bolivia (Plurinational State of)**	0.566	0.12 (0.08,0.17)	0.21 (0.14,0.32)	0.78 (0.43,1.22)	0.14 (0.08,0.27)	0.25 (0.12,0.49)	0.74 (0.37,1.17)	0.12 (0.08,0.17)	0.21 (0.13,0.31)	0.79 (0.42,1.29)	0.10 (0.06,0.14)	0.16 (0.10,0.24)	0.67 (0.36,1.21)
**Bosnia and Herzegovina**	0.718	0.15 (0.09,0.25)	0.32 (0.19,0.54)	1.11 (0.69,1.68)	0.16 (0.08,0.29)	0.33 (0.17,0.62)	1.07 (0.61,1.69)	0.14 (0.09,0.21)	0.30 (0.18,0.45)	1.17 (0.73,1.74)	0.13 (0.08,0.19)	0.26 (0.16,0.38)	1.06 (0.66,1.64)
**Botswana**	0.634	0.17 (0.10,0.26)	0.29 (0.19,0.44)	0.73 (0.39,1.23)	0.20 (0.09,0.43)	0.32 (0.15,0.61)	0.60 (0.26,1.14)	0.15 (0.09,0.22)	0.27 (0.16,0.39)	0.81 (0.42,1.38)	0.13 (0.08,0.20)	0.22 (0.14,0.32)	0.63 (0.29,1.15)
**Brazil**	0.640	0.20 (0.13,0.31)	0.23 (0.15,0.36)	0.17 (0.07,0.28)	0.23 (0.11,0.47)	0.27 (0.13,0.52)	0.15 (0.04,0.26)	0.18 (0.11,0.26)	0.22 (0.13,0.32)	0.21 (0.09,0.34)	0.12 (0.07,0.18)	0.16 (0.10,0.23)	0.29 (0.13,0.51)
**Brunei Darussalam**	0.823	0.34 (0.23,0.47)	0.34 (0.24,0.50)	0.02 (-0.11,0.17)	0.35 (0.18,0.65)	0.35 (0.18,0.64)	-0.02 (-0.15,0.14)	0.33 (0.21,0.48)	0.35 (0.22,0.50)	0.04 (-0.11,0.20)	0.29 (0.19,0.41)	0.33 (0.20,0.46)	0.13 (-0.02,0.31)
**Bulgaria**	0.764	0.14 (0.09,0.20)	0.23 (0.14,0.38)	0.69 (0.30,1.17)	0.15 (0.08,0.27)	0.25 (0.12,0.49)	0.68 (0.31,1.14)	0.13 (0.09,0.19)	0.21 (0.13,0.33)	0.62 (0.27,1.07)	0.10 (0.07,0.15)	0.17 (0.11,0.25)	0.59 (0.27,1.09)
**Burkina Faso**	0.257	0.11 (0.07,0.18)	0.20 (0.13,0.31)	0.83 (0.38,1.45)	0.14 (0.07,0.33)	0.24 (0.11,0.51)	0.67 (0.25,1.34)	0.10 (0.06,0.17)	0.20 (0.11,0.31)	0.90 (0.37,1.58)	0.07 (0.04,0.12)	0.12 (0.07,0.21)	0.76 (0.27,1.50)
**Burundi**	0.284	0.10 (0.06,0.15)	0.14 (0.09,0.22)	0.42 (0.11,0.85)	0.13 (0.06,0.27)	0.17 (0.07,0.35)	0.33 (0.01,0.77)	0.09 (0.06,0.14)	0.14 (0.08,0.22)	0.43 (0.11,0.89)	0.08 (0.05,0.12)	0.11 (0.07,0.17)	0.34 (0.05,0.78)
**Cabo Verde**	0.525	0.12 (0.08,0.18)	0.24 (0.16,0.36)	1.01 (0.60,1.58)	0.14 (0.08,0.27)	0.27 (0.14,0.54)	0.98 (0.53,1.54)	0.12 (0.08,0.17)	0.23 (0.14,0.34)	0.97 (0.48,1.62)	0.09 (0.06,0.14)	0.17 (0.11,0.25)	0.83 (0.36,1.55)
**Cambodia**	0.469	0.09 (0.06,0.13)	0.28 (0.18,0.41)	2.19 (1.57,2.90)	0.10 (0.05,0.20)	0.31 (0.14,0.61)	1.94 (1.29,2.67)	0.08 (0.05,0.12)	0.27 (0.15,0.41)	2.25 (1.53,3.10)	0.07 (0.04,0.11)	0.21 (0.12,0.32)	2.01 (1.30,2.96)
**Cameroon**	0.490	0.10 (0.07,0.15)	0.16 (0.11,0.23)	0.52 (0.29,0.87)	0.13 (0.07,0.23)	0.18 (0.10,0.35)	0.46 (0.15,0.88)	0.10 (0.06,0.14)	0.15 (0.10,0.22)	0.54 (0.30,0.88)	0.07 (0.04,0.11)	0.10 (0.06,0.16)	0.48 (0.20,0.88)
**Canada**	0.873	0.09 (0.05,0.16)	0.19 (0.11,0.35)	1.14 (0.61,1.77)	0.10 (0.05,0.21)	0.21 (0.09,0.46)	1.13 (0.56,1.83)	0.09 (0.05,0.13)	0.19 (0.10,0.30)	1.22 (0.66,1.85)	0.06 (0.04,0.10)	0.14 (0.08,0.22)	1.20 (0.68,1.91)
**Central African Republic**	0.274	0.14 (0.10,0.21)	0.23 (0.15,0.34)	0.62 (0.29,1.07)	0.16 (0.08,0.30)	0.26 (0.13,0.49)	0.61 (0.24,1.09)	0.14 (0.09,0.20)	0.22 (0.13,0.34)	0.62 (0.28,1.06)	0.12 (0.08,0.18)	0.20 (0.11,0.30)	0.57 (0.25,1.02)

**Chad**	0.238	0.10<br>(0.07,0.15)	0.18<br>(0.11,0.26)	0.72<br>(0.36,1.19)	0.12<br>(0.06,0.23)	0.21<br>(0.10,0.43)	0.78<br>(0.36,1.30)	0.10<br>(0.06,0.14)	0.17<br>(0.10,0.25)	0.69<br>(0.34,1.22)	0.07<br>(0.04,0.11)	0.11<br>(0.06,0.18)	0.57<br>(0.22,1.18)
**Chile**	0.759	0.16<br>(0.10,0.25)	0.29<br>(0.18,0.46)	0.81<br>(0.45,1.33)	0.18<br>(0.09,0.35)	0.32<br>(0.15,0.64)	0.80<br>(0.41,1.40)	0.15<br>(0.09,0.22)	0.28<br>(0.16,0.40)	0.84<br>(0.48,1.35)	0.11<br>(0.07,0.17)	0.21<br>(0.13,0.30)	0.87<br>(0.47,1.44)
**China**	0.686	0.14<br>(0.10,0.20)	0.16<br>(0.11,0.23)	0.15<br>(-0.03,0.35)	0.15<br>(0.08,0.28)	0.17<br>(0.09,0.30)	0.10<br>(-0.04,0.25)	0.14<br>(0.09,0.20)	0.15<br>(0.10,0.23)	0.14<br>(-0.00,0.26)	0.13<br>(0.08,0.19)	0.15<br>(0.10,0.21)	0.12<br>(-0.03,0.29)
**Colombia**	0.633	0.25<br>(0.16,0.38)	0.27<br>(0.18,0.40)	0.10<br>(-0.05,0.27)	0.30<br>(0.14,0.57)	0.31<br>(0.15,0.61)	0.05<br>(-0.08,0.19)	0.24<br>(0.14,0.36)	0.28<br>(0.17,0.41)	0.19<br>(0.02,0.39)	0.16<br>(0.10,0.23)	0.22<br>(0.14,0.32)	0.38<br>(0.13,0.69)
**Comoros**	0.455	0.09<br>(0.06,0.15)	0.14<br>(0.08,0.24)	0.59<br>(0.14,1.24)	0.11<br>(0.05,0.25)	0.16<br>(0.07,0.39)	0.48<br>(0.03,1.12)	0.08<br>(0.05,0.13)	0.13<br>(0.07,0.22)	0.62<br>(0.13,1.31)	0.07<br>(0.04,0.11)	0.10<br>(0.06,0.17)	0.49<br>(0.05,1.10)
**Congo**	0.568	0.16<br>(0.10,0.23)	0.24<br>(0.16,0.35)	0.50<br>(0.23,0.89)	0.18<br>(0.09,0.35)	0.26<br>(0.13,0.52)	0.45<br>(0.17,0.84)	0.15<br>(0.09,0.22)	0.22<br>(0.14,0.33)	0.50<br>(0.22,0.87)	0.13<br>(0.08,0.19)	0.19<br>(0.11,0.28)	0.42<br>(0.16,0.80)
**Cook Islands**	0.764	0.25<br>(0.17,0.35)	0.32<br>(0.23,0.44)	0.31<br>(0.07,0.57)	0.28<br>(0.15,0.52)	0.33<br>(0.20,0.55)	0.15<br>(-0.06,0.44)	0.24<br>(0.15,0.36)	0.33<br>(0.22,0.46)	0.37<br>(0.06,0.71)	0.19<br>(0.12,0.27)	0.28<br>(0.17,0.41)	0.51<br>(0.18,0.89)
**Costa Rica**	0.680	0.23<br>(0.15,0.36)	0.28<br>(0.19,0.43)	0.22<br>(0.02,0.48)	0.27<br>(0.13,0.55)	0.30<br>(0.16,0.58)	0.14<br>(-0.05,0.39)	0.22<br>(0.14,0.32)	0.28<br>(0.18,0.41)	0.30<br>(0.06,0.61)	0.17<br>(0.11,0.24)	0.23<br>(0.15,0.33)	0.39<br>(0.14,0.70)
**Croatia**	0.794	0.17<br>(0.11,0.29)	0.28<br>(0.17,0.48)	0.63<br>(0.29,1.08)	0.19<br>(0.09,0.39)	0.30<br>(0.14,0.60)	0.57<br>(0.22,1.02)	0.15<br>(0.10,0.23)	0.27<br>(0.16,0.41)	0.76<br>(0.41,1.23)	0.12<br>(0.08,0.18)	0.20<br>(0.13,0.29)	0.64<br>(0.34,1.08)
**Cuba**	0.668	0.20<br>(0.13,0.30)	0.27<br>(0.18,0.42)	0.34<br>(0.09,0.67)	0.23<br>(0.12,0.43)	0.29<br>(0.15,0.53)	0.26<br>(-0.00,0.59)	0.19<br>(0.12,0.27)	0.26<br>(0.16,0.38)	0.40<br>(0.13,0.75)	0.15<br>(0.09,0.21)	0.21<br>(0.13,0.30)	0.39<br>(0.16,0.73)
**Cyprus**	0.841	0.28<br>(0.17,0.51)	0.29<br>(0.17,0.51)	0.02<br>(-0.06,0.12)	0.30<br>(0.13,0.71)	0.30<br>(0.13,0.69)	-0.01<br>(-0.09,0.08)	0.27<br>(0.15,0.43)	0.29<br>(0.17,0.45)	0.06<br>(-0.04,0.19)	0.20<br>(0.12,0.31)	0.22<br>(0.14,0.33)	0.11<br>(-0.04,0.30)
**Czechia**	0.828	0.24<br>(0.14,0.45)	0.32<br>(0.20,0.52)	0.31<br>(0.05,0.69)	0.26<br>(0.12,0.54)	0.33<br>(0.17,0.62)	0.24<br>(-0.00,0.62)	0.21<br>(0.13,0.31)	0.32<br>(0.20,0.45)	0.53<br>(0.22,0.90)	0.15<br>(0.10,0.22)	0.27<br>(0.17,0.38)	0.74<br>(0.44,1.14)
**Côte d'Ivoire**	0.408	0.10<br>(0.07,0.16)	0.20<br>(0.13,0.31)	0.96<br>(0.55,1.50)	0.12<br>(0.06,0.24)	0.24<br>(0.11,0.50)	0.95<br>(0.49,1.53)	0.10<br>(0.06,0.15)	0.19<br>(0.11,0.30)	0.96<br>(0.53,1.55)	0.07<br>(0.04,0.11)	0.12<br>(0.07,0.20)	0.77<br>(0.35,1.51)

**Democratic People's Republic of Korea**	0.558	0.11 (0.08,0.15)	0.15 (0.10,0.21)	0.35 (0.16,0.65)	0.11 (0.07,0.20)	0.16 (0.09,0.27)	0.35 (0.15,0.72)	0.11 (0.07,0.15)	0.14 (0.09,0.21)	0.34 (0.15,0.63)	0.10 (0.06,0.15)	0.13 (0.09,0.19)	0.33 (0.14,0.62)
**Democratic Republic of the Congo**	0.382	0.15 (0.10,0.22)	0.22 (0.14,0.32)	0.45 (0.19,0.86)	0.16 (0.08,0.33)	0.24 (0.12,0.48)	0.45 (0.12,0.90)	0.14 (0.09,0.21)	0.21 (0.13,0.31)	0.45 (0.17,0.86)	0.13 (0.08,0.19)	0.18 (0.11,0.26)	0.37 (0.11,0.76)
**Denmark**	0.890	0.09 (0.06,0.14)	0.18 (0.11,0.31)	1.04 (0.57,1.64)	0.09 (0.05,0.19)	0.19 (0.09,0.41)	1.07 (0.54,1.73)	0.09 (0.06,0.13)	0.18 (0.10,0.28)	1.04 (0.56,1.72)	0.07 (0.05,0.11)	0.14 (0.09,0.21)	0.93 (0.50,1.61)
**Djibouti**	0.459	0.10 (0.06,0.16)	0.17 (0.10,0.27)	0.60 (0.24,1.12)	0.13 (0.06,0.27)	0.19 (0.09,0.40)	0.49 (0.10,1.05)	0.09 (0.06,0.15)	0.15 (0.08,0.25)	0.61 (0.22,1.18)	0.08 (0.05,0.12)	0.12 (0.07,0.19)	0.51 (0.18,1.00)
**Dominica**	0.729	0.26 (0.17,0.39)	0.34 (0.23,0.49)	0.29 (0.09,0.57)	0.29 (0.14,0.56)	0.34 (0.17,0.62)	0.16 (-0.01,0.44)	0.25 (0.15,0.37)	0.35 (0.22,0.48)	0.40 (0.14,0.76)	0.19 (0.12,0.28)	0.29 (0.18,0.41)	0.49 (0.20,0.83)
**Dominican Republic**	0.592	0.08 (0.06,0.12)	0.18 (0.12,0.28)	1.20 (0.72,1.80)	0.09 (0.05,0.18)	0.22 (0.10,0.43)	1.29 (0.73,1.96)	0.08 (0.05,0.11)	0.17 (0.10,0.25)	1.12 (0.68,1.76)	0.06 (0.04,0.09)	0.13 (0.08,0.19)	1.00 (0.63,1.57)
**Ecuador**	0.640	0.13 (0.09,0.21)	0.24 (0.15,0.37)	0.78 (0.47,1.17)	0.17 (0.08,0.36)	0.28 (0.13,0.58)	0.70 (0.38,1.17)	0.12 (0.08,0.18)	0.22 (0.13,0.33)	0.81 (0.48,1.23)	0.10 (0.06,0.14)	0.17 (0.11,0.25)	0.80 (0.46,1.31)
**Egypt**	0.658	0.07 (0.04,0.11)	0.25 (0.15,0.39)	2.59 (1.60,3.89)	0.10 (0.05,0.20)	0.28 (0.14,0.50)	1.79 (0.99,2.78)	0.06 (0.04,0.09)	0.19 (0.11,0.30)	2.34 (1.31,3.79)	0.03 (0.01,0.05)	0.12 (0.07,0.19)	3.46 (1.61,8.89)
**El Salvador**	0.573	0.14 (0.09,0.21)	0.29 (0.20,0.41)	1.05 (0.63,1.64)	0.18 (0.09,0.36)	0.32 (0.16,0.61)	0.78 (0.37,1.36)	0.13 (0.08,0.19)	0.29 (0.17,0.41)	1.13 (0.69,1.72)	0.10 (0.06,0.15)	0.22 (0.14,0.31)	1.20 (0.68,1.91)
**Equatorial Guinea**	0.685	0.15 (0.10,0.21)	0.24 (0.16,0.37)	0.67 (0.26,1.19)	0.16 (0.08,0.31)	0.26 (0.13,0.51)	0.58 (0.20,1.10)	0.14 (0.09,0.21)	0.24 (0.14,0.36)	0.66 (0.31,1.12)	0.12 (0.07,0.19)	0.20 (0.12,0.30)	0.58 (0.24,1.05)
**Eritrea**	0.396	0.09 (0.06,0.13)	0.15 (0.09,0.23)	0.64 (0.25,1.23)	0.11 (0.05,0.22)	0.17 (0.08,0.35)	0.55 (0.11,1.11)	0.09 (0.05,0.13)	0.14 (0.08,0.22)	0.63 (0.25,1.23)	0.08 (0.05,0.12)	0.12 (0.07,0.18)	0.49 (0.18,0.98)
**Estonia**	0.835	0.11 (0.07,0.18)	0.15 (0.10,0.23)	0.30 (0.16,0.51)	0.12 (0.07,0.20)	0.15 (0.09,0.26)	0.30 (0.16,0.52)	0.11 (0.07,0.16)	0.14 (0.10,0.20)	0.32 (0.17,0.51)	0.09 (0.06,0.15)	0.13 (0.08,0.19)	0.39 (0.18,0.67)

**Eswatini**	0.577	0.18<br>(0.12,0.27)	0.27<br>(0.19,0.40)	0.54<br>(0.23,0.93)	0.20<br>(0.10,0.39)	0.30<br>(0.15,0.56)	0.52<br>(0.17,0.97)	0.17<br>(0.11,0.24)	0.26<br>(0.15,0.37)	0.53<br>(0.21,0.93)	0.15<br>(0.10,0.22)	0.22<br>(0.14,0.32)	0.45<br>(0.16,0.86)
**Ethiopia**	0.343	0.11<br>(0.07,0.16)	0.11<br>(0.08,0.17)	0.05<br>(-0.09,0.27)	0.12<br>(0.06,0.24)	0.12<br>(0.06,0.26)	0.00<br>(-0.15,0.18)	0.11<br>(0.07,0.15)	0.11<br>(0.07,0.16)	0.05<br>(-0.09,0.21)	0.09<br>(0.06,0.14)	0.10<br>(0.06,0.14)	0.01<br>(-0.11,0.17)
**Fiji**	0.664	0.31<br>(0.21,0.42)	0.32<br>(0.23,0.44)	0.05<br>(-0.12,0.27)	0.33<br>(0.18,0.58)	0.32<br>(0.19,0.54)	-0.01<br>(-0.19,0.23)	0.31<br>(0.19,0.43)	0.33<br>(0.22,0.46)	0.08<br>(-0.11,0.34)	0.26<br>(0.17,0.38)	0.29<br>(0.18,0.43)	0.11<br>(-0.10,0.41)
**Finland**	0.856	0.17<br>(0.10,0.29)	0.27<br>(0.16,0.46)	0.62<br>(0.31,1.04)	0.18<br>(0.09,0.37)	0.29<br>(0.14,0.60)	0.61<br>(0.27,1.03)	0.15<br>(0.10,0.22)	0.26<br>(0.16,0.40)	0.70<br>(0.34,1.18)	0.13<br>(0.08,0.19)	0.21<br>(0.13,0.29)	0.62<br>(0.29,1.14)
**France**	0.834	0.09<br>(0.06,0.15)	0.12<br>(0.08,0.19)	0.29<br>(0.15,0.52)	0.10<br>(0.05,0.19)	0.13<br>(0.07,0.24)	0.32<br>(0.16,0.60)	0.09<br>(0.06,0.13)	0.11<br>(0.07,0.17)	0.32<br>(0.14,0.55)	0.07<br>(0.04,0.11)	0.09<br>(0.06,0.13)	0.29<br>(0.13,0.51)
**Gabon**	0.656	0.18<br>(0.12,0.27)	0.28<br>(0.18,0.40)	0.51<br>(0.24,0.85)	0.20<br>(0.10,0.40)	0.28<br>(0.14,0.53)	0.40<br>(0.15,0.74)	0.18<br>(0.11,0.26)	0.27<br>(0.16,0.40)	0.56<br>(0.27,0.94)	0.14<br>(0.09,0.21)	0.22<br>(0.14,0.32)	0.53<br>(0.22,0.88)
**Gambia**	0.399	0.09<br>(0.06,0.14)	0.18<br>(0.11,0.28)	0.94<br>(0.55,1.52)	0.11<br>(0.06,0.21)	0.21<br>(0.10,0.41)	0.98<br>(0.53,1.57)	0.09<br>(0.05,0.13)	0.17<br>(0.10,0.27)	0.92<br>(0.50,1.52)	0.06<br>(0.04,0.10)	0.12<br>(0.07,0.18)	0.82<br>(0.37,1.62)
**Georgia**	0.702	0.11<br>(0.07,0.15)	0.30<br>(0.20,0.43)	1.84<br>(1.35,2.41)	0.11<br>(0.06,0.21)	0.32<br>(0.15,0.62)	1.85<br>(1.31,2.43)	0.11<br>(0.07,0.15)	0.30<br>(0.18,0.45)	1.87<br>(1.31,2.47)	0.10<br>(0.06,0.14)	0.26<br>(0.16,0.39)	1.73<br>(1.18,2.37)
**Germany**	0.898	0.25<br>(0.14,0.46)	0.29<br>(0.18,0.50)	0.15<br>(0.02,0.39)	0.28<br>(0.12,0.59)	0.31<br>(0.15,0.63)	0.10<br>(-0.00,0.32)	0.23<br>(0.13,0.35)	0.29<br>(0.17,0.43)	0.27<br>(0.10,0.55)	0.16<br>(0.10,0.24)	0.23<br>(0.14,0.33)	0.47<br>(0.22,0.82)
**Ghana**	0.557	0.12<br>(0.08,0.20)	0.22<br>(0.14,0.34)	0.76<br>(0.42,1.29)	0.15<br>(0.07,0.31)	0.25<br>(0.12,0.50)	0.72<br>(0.35,1.30)	0.11<br>(0.07,0.18)	0.20<br>(0.12,0.30)	0.75<br>(0.42,1.28)	0.08<br>(0.05,0.14)	0.14<br>(0.09,0.21)	0.68<br>(0.33,1.20)
**Greece**	0.794	0.12<br>(0.08,0.21)	0.21<br>(0.13,0.34)	0.67<br>(0.30,1.09)	0.13<br>(0.07,0.27)	0.22<br>(0.10,0.48)	0.67<br>(0.26,1.16)	0.12<br>(0.08,0.17)	0.20<br>(0.12,0.32)	0.71<br>(0.35,1.15)	0.09<br>(0.06,0.13)	0.15<br>(0.10,0.23)	0.70<br>(0.37,1.12)
**Greenland**	0.761	0.07<br>(0.05,0.11)	0.23<br>(0.14,0.38)	2.23<br>(1.51,3.08)	0.08<br>(0.04,0.15)	0.26<br>(0.11,0.57)	2.39<br>(1.49,3.33)	0.07<br>(0.05,0.11)	0.23<br>(0.13,0.36)	2.16<br>(1.38,3.10)	0.06<br>(0.03,0.09)	0.16<br>(0.10,0.25)	1.92<br>(1.15,3.05)
**Grenada**	0.669	0.27<br>(0.17,0.39)	0.34<br>(0.23,0.49)	0.27<br>(0.08,0.54)	0.29<br>(0.14,0.55)	0.34<br>(0.18,0.62)	0.17<br>(-0.01,0.40)	0.25<br>(0.14,0.37)	0.34<br>(0.21,0.49)	0.39<br>(0.13,0.75)	0.19<br>(0.12,0.28)	0.28<br>(0.17,0.40)	0.42<br>(0.16,0.77)

**Guam**	0.813	0.18 (0.12,0.27)	0.25 (0.17,0.34)	0.36 (0.10,0.69)	0.20 (0.10,0.37)	0.27 (0.15,0.48)	0.37 (0.07,0.75)	0.17 (0.11,0.26)	0.24 (0.16,0.34)	0.37 (0.08,0.68)	0.15 (0.09,0.21)	0.19 (0.12,0.28)	0.29 (0.01,0.58)
**Guatemala**	0.526	0.17 (0.11,0.26)	0.30 (0.20,0.43)	0.79 (0.45,1.25)	0.22 (0.10,0.46)	0.33 (0.16,0.66)	0.50 (0.22,0.91)	0.15 (0.09,0.22)	0.30 (0.19,0.43)	1.05 (0.64,1.60)	0.10 (0.06,0.15)	0.21 (0.13,0.32)	1.10 (0.66,1.68)
**Guinea**	0.325	0.09 (0.06,0.13)	0.17 (0.11,0.27)	0.96 (0.54,1.51)	0.10 (0.05,0.20)	0.21 (0.10,0.44)	1.04 (0.55,1.63)	0.08 (0.05,0.12)	0.16 (0.10,0.25)	0.92 (0.52,1.49)	0.06 (0.03,0.10)	0.11 (0.06,0.17)	0.74 (0.33,1.57)
**Guinea-Bissau**	0.355	0.10 (0.07,0.15)	0.19 (0.12,0.28)	0.90 (0.50,1.40)	0.12 (0.06,0.23)	0.23 (0.11,0.46)	0.92 (0.47,1.47)	0.09 (0.06,0.14)	0.18 (0.10,0.27)	0.86 (0.46,1.39)	0.07 (0.04,0.11)	0.13 (0.07,0.20)	0.80 (0.36,1.52)
**Guyana**	0.618	0.30 (0.20,0.42)	0.36 (0.25,0.49)	0.19 (0.00,0.45)	0.33 (0.16,0.60)	0.36 (0.19,0.62)	0.09 (-0.08,0.31)	0.29 (0.17,0.42)	0.36 (0.22,0.51)	0.26 (0.05,0.57)	0.21 (0.13,0.31)	0.28 (0.17,0.42)	0.36 (0.09,0.73)
**Haiti**	0.432	0.22 (0.15,0.32)	0.32 (0.22,0.45)	0.45 (0.21,0.80)	0.26 (0.13,0.50)	0.34 (0.17,0.64)	0.32 (0.12,0.66)	0.21 (0.12,0.31)	0.32 (0.19,0.45)	0.52 (0.25,0.89)	0.18 (0.10,0.28)	0.25 (0.15,0.39)	0.45 (0.11,0.91)
**Honduras**	0.496	0.21 (0.13,0.30)	0.32 (0.21,0.46)	0.54 (0.33,0.86)	0.27 (0.13,0.52)	0.34 (0.17,0.65)	0.28 (0.12,0.53)	0.19 (0.11,0.28)	0.32 (0.19,0.46)	0.69 (0.41,1.09)	0.14 (0.08,0.21)	0.26 (0.16,0.39)	0.91 (0.52,1.52)
**Hungary**	0.791	0.17 (0.10,0.26)	0.28 (0.17,0.46)	0.68 (0.35,1.14)	0.18 (0.09,0.34)	0.30 (0.14,0.56)	0.66 (0.32,1.18)	0.15 (0.10,0.22)	0.26 (0.16,0.37)	0.72 (0.37,1.19)	0.11 (0.07,0.17)	0.19 (0.12,0.28)	0.71 (0.37,1.27)
**Iceland**	0.869	0.11 (0.07,0.17)	0.21 (0.13,0.36)	0.96 (0.53,1.46)	0.11 (0.06,0.22)	0.23 (0.11,0.49)	0.99 (0.52,1.57)	0.10 (0.07,0.15)	0.21 (0.12,0.34)	1.01 (0.53,1.61)	0.09 (0.06,0.13)	0.16 (0.10,0.24)	0.83 (0.45,1.37)
**India**	0.566	0.16 (0.11,0.22)	0.27 (0.19,0.39)	0.75 (0.57,0.97)	0.17 (0.09,0.33)	0.29 (0.15,0.54)	0.68 (0.48,0.92)	0.15 (0.09,0.22)	0.27 (0.16,0.39)	0.78 (0.56,1.03)	0.13 (0.08,0.19)	0.22 (0.14,0.32)	0.70 (0.49,0.95)
**Indonesia**	0.660	0.11 (0.07,0.16)	0.19 (0.12,0.29)	0.72 (0.44,1.06)	0.13 (0.07,0.25)	0.21 (0.10,0.44)	0.67 (0.42,0.96)	0.10 (0.07,0.15)	0.17 (0.10,0.26)	0.69 (0.42,1.00)	0.09 (0.05,0.13)	0.14 (0.09,0.20)	0.62 (0.37,0.93)
**Iran (Islamic Republic of)**	0.670	0.14 (0.09,0.24)	0.28 (0.17,0.47)	0.94 (0.68,1.25)	0.15 (0.08,0.28)	0.29 (0.15,0.54)	0.95 (0.68,1.24)	0.12 (0.08,0.18)	0.24 (0.15,0.34)	0.91 (0.65,1.23)	0.14 (0.08,0.21)	0.23 (0.15,0.34)	0.70 (0.46,1.06)
**Iraq**	0.671	0.20 (0.13,0.29)	0.30 (0.20,0.44)	0.55 (0.21,1.00)	0.21 (0.12,0.37)	0.32 (0.18,0.55)	0.51 (0.16,0.97)	0.18 (0.12,0.25)	0.28 (0.18,0.39)	0.56 (0.23,0.96)	0.16 (0.10,0.23)	0.24 (0.15,0.33)	0.48 (0.15,0.86)

**Ireland**	0.867	0.09<br>(0.06,0.15)	0.22<br>(0.13,0.38)	1.43<br>(0.84,2.21)	0.09<br>(0.05,0.18)	0.24<br>(0.11,0.52)	1.54<br>(0.82,2.46)	0.09<br>(0.06,0.13)	0.22<br>(0.13,0.35)	1.43<br>(0.81,2.30)	0.08<br>(0.05,0.12)	0.16<br>(0.10,0.24)	1.10<br>(0.62,1.94)
**Israel**	0.803	0.14<br>(0.09,0.22)	0.25<br>(0.15,0.42)	0.84<br>(0.46,1.33)	0.14<br>(0.07,0.29)	0.27<br>(0.12,0.57)	0.85<br>(0.43,1.41)	0.14<br>(0.08,0.21)	0.25<br>(0.14,0.39)	0.86<br>(0.47,1.37)	0.10<br>(0.07,0.16)	0.20<br>(0.12,0.30)	0.90<br>(0.47,1.52)
**Italy**	0.801	0.20<br>(0.11,0.34)	0.26<br>(0.16,0.46)	0.32<br>(0.14,0.56)	0.22<br>(0.10,0.43)	0.28<br>(0.12,0.62)	0.28<br>(0.09,0.55)	0.18<br>(0.11,0.26)	0.26<br>(0.16,0.39)	0.45<br>(0.27,0.71)	0.13<br>(0.09,0.19)	0.20<br>(0.13,0.29)	0.54<br>(0.36,0.77)
**Jamaica**	0.684	0.22<br>(0.14,0.36)	0.33<br>(0.22,0.48)	0.47<br>(0.21,0.82)	0.27<br>(0.12,0.56)	0.33<br>(0.16,0.66)	0.26<br>(0.09,0.53)	0.21<br>(0.12,0.33)	0.34<br>(0.20,0.49)	0.61<br>(0.28,1.07)	0.13<br>(0.08,0.20)	0.26<br>(0.16,0.38)	1.03<br>(0.58,1.63)
**Japan**	0.870	0.13<br>(0.08,0.21)	0.14<br>(0.09,0.22)	0.09<br>(-0.03,0.24)	0.14<br>(0.07,0.28)	0.16<br>(0.08,0.31)	0.13<br>(-0.01,0.27)	0.13<br>(0.08,0.18)	0.14<br>(0.09,0.21)	0.13<br>(0.02,0.27)	0.11<br>(0.07,0.16)	0.12<br>(0.07,0.17)	0.06<br>(-0.04,0.17)
**Jordan**	0.731	0.25<br>(0.16,0.41)	0.30<br>(0.18,0.48)	0.20<br>(-0.05,0.50)	0.26<br>(0.14,0.49)	0.31<br>(0.16,0.54)	0.17<br>(-0.07,0.47)	0.22<br>(0.14,0.33)	0.28<br>(0.18,0.40)	0.25<br>(-0.01,0.58)	0.19<br>(0.12,0.28)	0.23<br>(0.15,0.33)	0.23<br>(-0.01,0.53)
**Kazakhstan**	0.723	0.14<br>(0.08,0.22)	0.29<br>(0.19,0.45)	1.15<br>(0.61,1.87)	0.14<br>(0.07,0.27)	0.31<br>(0.15,0.60)	1.18<br>(0.59,1.93)	0.13<br>(0.08,0.20)	0.28<br>(0.16,0.41)	1.15<br>(0.60,1.88)	0.11<br>(0.07,0.17)	0.24<br>(0.15,0.36)	1.12<br>(0.59,1.84)
**Kenya**	0.508	0.11<br>(0.07,0.16)	0.15<br>(0.10,0.22)	0.38<br>(0.23,0.55)	0.12<br>(0.06,0.27)	0.16<br>(0.08,0.32)	0.27<br>(0.08,0.45)	0.10<br>(0.06,0.16)	0.14<br>(0.09,0.22)	0.43<br>(0.24,0.62)	0.08<br>(0.05,0.13)	0.12<br>(0.07,0.17)	0.40<br>(0.22,0.60)
**Kiribati**	0.527	0.26<br>(0.18,0.37)	0.33<br>(0.23,0.45)	0.26<br>(0.00,0.61)	0.28<br>(0.16,0.47)	0.34<br>(0.20,0.54)	0.20<br>(-0.05,0.54)	0.27<br>(0.17,0.39)	0.34<br>(0.22,0.47)	0.26<br>(-0.01,0.62)	0.20<br>(0.12,0.29)	0.28<br>(0.17,0.43)	0.44<br>(0.14,0.78)
**Kuwait**	0.851	0.28<br>(0.18,0.47)	0.30<br>(0.19,0.46)	0.06<br>(-0.11,0.30)	0.31<br>(0.16,0.56)	0.31<br>(0.18,0.54)	0.02<br>(-0.14,0.25)	0.24<br>(0.15,0.36)	0.28<br>(0.18,0.40)	0.15<br>(-0.06,0.41)	0.18<br>(0.12,0.26)	0.25<br>(0.17,0.35)	0.39<br>(0.15,0.69)
**Kyrgyzstan**	0.596	0.08<br>(0.05,0.13)	0.12<br>(0.08,0.18)	0.38<br>(0.18,0.72)	0.09<br>(0.05,0.16)	0.12<br>(0.06,0.23)	0.39<br>(0.16,0.78)	0.08<br>(0.05,0.12)	0.11<br>(0.07,0.16)	0.38<br>(0.18,0.71)	0.07<br>(0.04,0.12)	0.10<br>(0.06,0.15)	0.37<br>(0.18,0.68)
**Lao People's Democratic Republic**	0.490	0.14<br>(0.10,0.21)	0.27<br>(0.18,0.38)	0.87<br>(0.51,1.37)	0.17<br>(0.09,0.34)	0.31<br>(0.15,0.60)	0.82<br>(0.40,1.39)	0.13<br>(0.08,0.20)	0.25<br>(0.15,0.37)	0.87<br>(0.50,1.37)	0.12<br>(0.07,0.18)	0.20<br>(0.12,0.28)	0.64<br>(0.32,1.14)
**Latvia**	0.820	0.11<br>(0.07,0.17)	0.15<br>(0.10,0.24)	0.35<br>(0.18,0.62)	0.12<br>(0.06,0.21)	0.15<br>(0.09,0.27)	0.35<br>(0.18,0.62)	0.11<br>(0.07,0.16)	0.15<br>(0.10,0.20)	0.35<br>(0.19,0.58)	0.10<br>(0.06,0.15)	0.13<br>(0.08,0.19)	0.35<br>(0.17,0.59)

**Lebanon**	0.708	0.19	0.31	0.62	0.21	0.32	0.54	0.17	0.27	0.59	0.14	0.22	0.62
		(0.11,0.34)	(0.19,0.53)	(0.27,1.15)	(0.10,0.44)	(0.17,0.59)	(0.21,1.09)	(0.10,0.26)	(0.17,0.39)	(0.26,1.06)	(0.08,0.21)	(0.14,0.32)	(0.24,1.11)
**Lesotho**	0.507	0.17	0.26	0.55	0.21	0.29	0.36	0.15	0.26	0.66	0.14	0.22	0.65
		(0.10,0.29)	(0.17,0.39)	(0.19,1.17)	(0.08,0.48)	(0.13,0.60)	(0.09,0.94)	(0.08,0.27)	(0.14,0.38)	(0.20,1.40)	(0.07,0.23)	(0.13,0.33)	(0.22,1.36)
**Liberia**	0.370	0.14	0.24	0.71	0.17	0.27	0.58	0.13	0.22	0.76	0.08	0.15	0.86
		(0.09,0.22)	(0.15,0.35)	(0.36,1.24)	(0.08,0.36)	(0.13,0.53)	(0.24,1.11)	(0.08,0.20)	(0.13,0.34)	(0.38,1.33)	(0.03,0.14)	(0.09,0.24)	(0.35,2.53)
**Libya**	0.709	0.21	0.32	0.55	0.25	0.34	0.34	0.18	0.28	0.57	0.10	0.22	1.23
		(0.13,0.34)	(0.20,0.52)	(0.28,0.98)	(0.12,0.50)	(0.18,0.61)	(0.11,0.74)	(0.10,0.26)	(0.17,0.40)	(0.23,1.14)	(0.05,0.16)	(0.13,0.33)	(0.49,2.67)
**Lithuania**	0.843	0.09	0.12	0.29	0.09	0.12	0.28	0.09	0.12	0.32	0.08	0.11	0.32
		(0.06,0.15)	(0.08,0.19)	(0.15,0.46)	(0.05,0.17)	(0.07,0.22)	(0.13,0.47)	(0.06,0.13)	(0.08,0.17)	(0.17,0.50)	(0.05,0.13)	(0.07,0.16)	(0.16,0.51)
**Luxembourg**	0.895	0.10	0.30	2.07	0.10	0.31	2.12	0.09	0.29	2.18	0.08	0.22	1.90
		(0.06,0.16)	(0.18,0.49)	(1.46,2.85)	(0.05,0.19)	(0.14,0.67)	(1.45,2.98)	(0.06,0.13)	(0.17,0.44)	(1.41,3.15)	(0.05,0.11)	(0.14,0.33)	(1.15,2.95)
**Madagascar**	0.396	0.08	0.12	0.44	0.11	0.14	0.33	0.08	0.11	0.45	0.07	0.09	0.37
		(0.06,0.13)	(0.08,0.18)	(0.16,0.84)	(0.06,0.23)	(0.07,0.30)	(0.02,0.78)	(0.05,0.12)	(0.07,0.17)	(0.17,0.86)	(0.04,0.10)	(0.06,0.14)	(0.10,0.79)
**Malawi**	0.384	0.14	0.21	0.56	0.17	0.24	0.36	0.12	0.20	0.66	0.10	0.16	0.57
		(0.08,0.22)	(0.13,0.32)	(0.17,1.11)	(0.07,0.41)	(0.11,0.50)	(0.02,0.91)	(0.07,0.20)	(0.11,0.32)	(0.19,1.28)	(0.06,0.17)	(0.09,0.24)	(0.14,1.20)
**Malaysia**	0.737	0.17	0.25	0.45	0.19	0.28	0.45	0.17	0.24	0.42	0.15	0.20	0.35
		(0.12,0.24)	(0.17,0.36)	(0.17,0.82)	(0.11,0.33)	(0.15,0.52)	(0.13,0.83)	(0.11,0.24)	(0.15,0.34)	(0.16,0.75)	(0.10,0.22)	(0.13,0.29)	(0.09,0.64)
**Maldives**	0.562	0.13	0.24	0.77	0.17	0.27	0.65	0.12	0.22	0.76	0.12	0.19	0.59
		(0.09,0.20)	(0.15,0.37)	(0.39,1.28)	(0.08,0.33)	(0.13,0.56)	(0.27,1.12)	(0.08,0.18)	(0.13,0.33)	(0.38,1.27)	(0.08,0.19)	(0.12,0.30)	(0.28,1.01)
**Mali**	0.263	0.09	0.18	0.93	0.11	0.21	0.95	0.09	0.17	0.92	0.06	0.11	0.66
		(0.06,0.14)	(0.11,0.28)	(0.48,1.57)	(0.06,0.21)	(0.10,0.44)	(0.44,1.61)	(0.05,0.13)	(0.10,0.26)	(0.48,1.60)	(0.03,0.10)	(0.06,0.18)	(0.25,1.38)
**Malta**	0.801	0.24	0.29	0.19	0.26	0.31	0.18	0.22	0.27	0.23	0.16	0.21	0.32
		(0.14,0.41)	(0.17,0.49)	(-0.00,0.49)	(0.12,0.53)	(0.14,0.65)	(-0.03,0.49)	(0.12,0.35)	(0.16,0.41)	(0.01,0.55)	(0.10,0.24)	(0.13,0.30)	(0.08,0.63)
**Marshall Islands**	0.544	0.35	0.37	0.07	0.36	0.36	-0.01	0.36	0.38	0.07	0.30	0.37	0.21
		(0.24,0.48)	(0.25,0.51)	(-0.12,0.30)	(0.20,0.63)	(0.22,0.58)	(-0.18,0.22)	(0.22,0.52)	(0.24,0.54)	(-0.14,0.35)	(0.19,0.44)	(0.21,0.55)	(-0.08,0.53)

**Mauritania**	0.496	0.08 (0.06,0.12)	0.13 (0.08,0.20)	0.52 (0.22,0.97)	0.09 (0.05,0.17)	0.14 (0.07,0.29)	0.53 (0.19,1.09)	0.08 (0.05,0.12)	0.12 (0.07,0.18)	0.49 (0.22,0.94)	0.06 (0.04,0.10)	0.09 (0.05,0.13)	0.39 (0.12,0.85)
**Mauritius**	0.705	0.23 (0.15,0.34)	0.29 (0.20,0.39)	0.26 (-0.01,0.59)	0.26 (0.13,0.49)	0.29 (0.17,0.50)	0.14 (-0.10,0.50)	0.20 (0.13,0.30)	0.29 (0.19,0.40)	0.41 (0.10,0.75)	0.17 (0.10,0.24)	0.25 (0.15,0.36)	0.50 (0.23,0.80)
**Mexico**	0.649	0.28 (0.19,0.42)	0.31 (0.22,0.44)	0.09 (0.01,0.20)	0.31 (0.16,0.56)	0.32 (0.18,0.59)	0.05 (-0.01,0.12)	0.27 (0.17,0.37)	0.31 (0.20,0.42)	0.15 (0.05,0.27)	0.21 (0.14,0.29)	0.26 (0.17,0.37)	0.27 (0.12,0.41)
**Micronesia (Federated States of)**	0.580	0.18 (0.12,0.25)	0.34 (0.24,0.46)	0.91 (0.55,1.40)	0.20 (0.11,0.38)	0.35 (0.19,0.60)	0.72 (0.35,1.25)	0.18 (0.12,0.25)	0.35 (0.22,0.48)	0.96 (0.58,1.47)	0.15 (0.10,0.23)	0.30 (0.19,0.43)	0.91 (0.50,1.45)
**Monaco**	0.902	0.11 (0.07,0.18)	0.19 (0.11,0.33)	0.73 (0.37,1.19)	0.11 (0.06,0.22)	0.20 (0.10,0.41)	0.77 (0.37,1.28)	0.11 (0.07,0.15)	0.18 (0.11,0.27)	0.68 (0.33,1.14)	0.09 (0.06,0.14)	0.14 (0.09,0.21)	0.54 (0.27,0.92)
**Mongolia**	0.606	0.06 (0.04,0.09)	0.08 (0.05,0.11)	0.39 (0.20,0.63)	0.06 (0.03,0.10)	0.08 (0.05,0.15)	0.45 (0.23,0.72)	0.06 (0.04,0.09)	0.08 (0.05,0.12)	0.39 (0.19,0.61)	0.06 (0.03,0.09)	0.08 (0.05,0.12)	0.41 (0.20,0.70)
**Montenegro**	0.791	0.18 (0.11,0.27)	0.30 (0.20,0.44)	0.71 (0.37,1.17)	0.19 (0.10,0.39)	0.32 (0.16,0.62)	0.65 (0.29,1.15)	0.18 (0.10,0.28)	0.30 (0.17,0.46)	0.71 (0.37,1.16)	0.14 (0.09,0.21)	0.24 (0.15,0.35)	0.71 (0.39,1.17)
**Morocco**	0.548	0.13 (0.09,0.21)	0.32 (0.20,0.52)	1.39 (0.79,2.10)	0.15 (0.08,0.29)	0.33 (0.17,0.61)	1.26 (0.75,1.88)	0.12 (0.08,0.18)	0.29 (0.17,0.42)	1.34 (0.86,1.95)	0.11 (0.06,0.16)	0.26 (0.16,0.38)	1.44 (0.89,2.23)
**Mozambique**	0.307	0.11 (0.07,0.16)	0.18 (0.12,0.28)	0.71 (0.32,1.27)	0.13 (0.06,0.28)	0.21 (0.10,0.42)	0.63 (0.17,1.26)	0.10 (0.06,0.16)	0.17 (0.10,0.27)	0.72 (0.30,1.33)	0.09 (0.05,0.13)	0.14 (0.08,0.22)	0.62 (0.27,1.17)
**Myanmar**	0.521	0.14 (0.09,0.21)	0.25 (0.17,0.37)	0.84 (0.46,1.37)	0.17 (0.08,0.33)	0.30 (0.14,0.58)	0.79 (0.38,1.37)	0.13 (0.08,0.19)	0.23 (0.14,0.35)	0.80 (0.42,1.38)	0.11 (0.07,0.17)	0.17 (0.11,0.25)	0.56 (0.23,1.00)
**Namibia**	0.612	0.18 (0.11,0.29)	0.25 (0.15,0.38)	0.38 (0.10,0.73)	0.21 (0.10,0.45)	0.28 (0.13,0.56)	0.30 (0.03,0.66)	0.16 (0.10,0.26)	0.23 (0.13,0.35)	0.42 (0.12,0.82)	0.15 (0.09,0.23)	0.20 (0.12,0.29)	0.34 (0.06,0.70)
**Nauru**	0.618	0.19 (0.13,0.28)	0.32 (0.22,0.44)	0.66 (0.35,1.05)	0.22 (0.12,0.41)	0.34 (0.19,0.57)	0.51 (0.21,0.94)	0.19 (0.12,0.27)	0.32 (0.20,0.45)	0.73 (0.38,1.13)	0.15 (0.09,0.23)	0.25 (0.15,0.37)	0.69 (0.33,1.12)
**Nepal**	0.422	0.11 (0.08,0.16)	0.20 (0.13,0.31)	0.82 (0.49,1.33)	0.12 (0.07,0.22)	0.22 (0.11,0.43)	0.81 (0.42,1.34)	0.11 (0.07,0.16)	0.20 (0.12,0.30)	0.81 (0.47,1.35)	0.10 (0.06,0.14)	0.17 (0.11,0.25)	0.73 (0.43,1.21)

**Netherlands**	0.883	0.13 (0.08,0.25)	0.18 (0.11,0.33)	0.38 (0.05,0.89)	0.15 (0.07,0.32)	0.20 (0.09,0.44)	0.35 (0.00,0.86)	0.12 (0.07,0.20)	0.18 (0.10,0.29)	0.43 (0.09,0.92)	0.09 (0.05,0.13)	0.13 (0.08,0.19)	0.48 (0.19,0.95)
**New Zealand**	0.840	0.10 (0.06,0.16)	0.16 (0.10,0.31)	0.64 (0.31,1.12)	0.10 (0.05,0.21)	0.18 (0.08,0.43)	0.72 (0.30,1.38)	0.10 (0.06,0.14)	0.15 (0.09,0.24)	0.60 (0.28,1.12)	0.08 (0.05,0.12)	0.12 (0.08,0.18)	0.50 (0.23,0.88)
**Nicaragua**	0.517	0.24 (0.16,0.36)	0.32 (0.21,0.49)	0.34 (0.14,0.62)	0.28 (0.13,0.57)	0.33 (0.16,0.65)	0.18 (0.01,0.41)	0.23 (0.13,0.33)	0.33 (0.19,0.48)	0.45 (0.23,0.78)	0.16 (0.10,0.23)	0.26 (0.16,0.37)	0.62 (0.33,1.02)
**Niger**	0.162	0.04 (0.03,0.06)	0.16 (0.10,0.26)	2.73 (1.71,3.91)	0.05 (0.03,0.11)	0.20 (0.08,0.45)	2.68 (1.57,3.83)	0.04 (0.03,0.06)	0.15 (0.08,0.26)	2.75 (1.57,4.12)	0.03 (0.01,0.05)	0.09 (0.04,0.17)	2.36 (1.13,4.84)
**Nigeria**	0.515	0.10 (0.07,0.16)	0.15 (0.09,0.25)	0.46 (0.26,0.71)	0.12 (0.06,0.28)	0.18 (0.08,0.39)	0.41 (0.21,0.69)	0.10 (0.06,0.15)	0.14 (0.08,0.23)	0.45 (0.25,0.72)	0.07 (0.04,0.11)	0.10 (0.05,0.16)	0.33 (0.12,0.61)
**Niue**	0.711	0.30 (0.21,0.41)	0.34 (0.25,0.46)	0.13 (-0.07,0.41)	0.33 (0.17,0.59)	0.33 (0.20,0.57)	0.02 (-0.17,0.30)	0.30 (0.18,0.43)	0.35 (0.23,0.49)	0.17 (-0.07,0.52)	0.23 (0.15,0.34)	0.30 (0.18,0.45)	0.30 (-0.04,0.70)
**North Macedonia**	0.744	0.19 (0.11,0.30)	0.33 (0.20,0.54)	0.74 (0.34,1.30)	0.20 (0.10,0.38)	0.34 (0.16,0.65)	0.66 (0.25,1.23)	0.17 (0.10,0.26)	0.32 (0.19,0.47)	0.86 (0.48,1.40)	0.13 (0.08,0.19)	0.25 (0.16,0.36)	0.94 (0.55,1.50)
**Northern Mariana Islands**	0.771	0.21 (0.14,0.30)	0.32 (0.22,0.44)	0.54 (0.26,0.92)	0.23 (0.13,0.45)	0.33 (0.18,0.59)	0.41 (0.14,0.81)	0.20 (0.13,0.29)	0.32 (0.20,0.46)	0.61 (0.31,1.03)	0.17 (0.11,0.25)	0.26 (0.17,0.38)	0.52 (0.23,0.90)
**Norway**	0.913	0.18 (0.10,0.30)	0.25 (0.15,0.44)	0.41 (0.25,0.61)	0.19 (0.09,0.38)	0.26 (0.12,0.57)	0.39 (0.22,0.59)	0.17 (0.10,0.25)	0.25 (0.14,0.39)	0.49 (0.28,0.76)	0.13 (0.08,0.19)	0.20 (0.13,0.28)	0.51 (0.25,0.83)
**Oman**	0.783	0.17 (0.11,0.25)	0.32 (0.20,0.51)	0.89 (0.43,1.46)	0.18 (0.10,0.32)	0.33 (0.17,0.62)	0.83 (0.42,1.36)	0.16 (0.10,0.25)	0.29 (0.18,0.43)	0.81 (0.41,1.34)	0.11 (0.07,0.17)	0.21 (0.13,0.31)	0.93 (0.45,1.67)
**Pakistan**	0.449	0.14 (0.10,0.19)	0.24 (0.16,0.33)	0.72 (0.42,1.06)	0.15 (0.08,0.27)	0.26 (0.14,0.49)	0.79 (0.46,1.15)	0.14 (0.09,0.19)	0.23 (0.14,0.33)	0.67 (0.38,1.04)	0.12 (0.08,0.18)	0.19 (0.12,0.27)	0.53 (0.28,0.87)
**Palau**	0.738	0.24 (0.17,0.34)	0.34 (0.24,0.46)	0.43 (0.12,0.76)	0.27 (0.15,0.50)	0.35 (0.21,0.57)	0.26 (-0.00,0.60)	0.23 (0.15,0.34)	0.35 (0.22,0.49)	0.51 (0.16,0.96)	0.18 (0.11,0.26)	0.29 (0.17,0.43)	0.59 (0.22,1.04)
**Palestine**	0.588	0.24 (0.14,0.40)	0.34 (0.20,0.57)	0.42 (0.16,0.82)	0.25 (0.12,0.51)	0.35 (0.17,0.66)	0.38 (0.15,0.77)	0.23 (0.13,0.35)	0.33 (0.20,0.48)	0.46 (0.20,0.87)	0.19 (0.11,0.29)	0.27 (0.17,0.40)	0.47 (0.18,0.92)

**Panama**	0.686	0.23<br>(0.14,0.37)	0.30<br>(0.20,0.45)	0.31<br>(0.11,0.59)	0.28<br>(0.12,0.57)	0.32<br>(0.16,0.65)	0.17<br>(0.01,0.42)	0.21<br>(0.13,0.33)	0.31<br>(0.19,0.45)	0.44<br>(0.18,0.83)	0.16<br>(0.10,0.23)	0.24<br>(0.15,0.35)	0.55<br>(0.25,0.93)
**Papua New Guinea**	0.394	0.20<br>(0.14,0.30)	0.34<br>(0.23,0.46)	0.67<br>(0.36,1.09)	0.22<br>(0.12,0.42)	0.34<br>(0.19,0.61)	0.59<br>(0.30,1.03)	0.21<br>(0.13,0.31)	0.35<br>(0.22,0.50)	0.67<br>(0.33,1.11)	0.15<br>(0.09,0.22)	0.27<br>(0.16,0.39)	0.81<br>(0.41,1.28)
**Paraguay**	0.638	0.13<br>(0.08,0.21)	0.25<br>(0.16,0.38)	0.90<br>(0.53,1.45)	0.16<br>(0.07,0.33)	0.29<br>(0.14,0.59)	0.83<br>(0.39,1.45)	0.12<br>(0.08,0.18)	0.23<br>(0.13,0.35)	0.93<br>(0.52,1.49)	0.09<br>(0.06,0.14)	0.17<br>(0.10,0.25)	0.85<br>(0.46,1.48)
**Peru**	0.648	0.09<br>(0.06,0.12)	0.14<br>(0.09,0.22)	0.65<br>(0.32,1.12)	0.10<br>(0.05,0.20)	0.17<br>(0.08,0.35)	0.64<br>(0.29,1.15)	0.08<br>(0.05,0.12)	0.13<br>(0.08,0.20)	0.61<br>(0.30,1.05)	0.06<br>(0.04,0.09)	0.10<br>(0.06,0.14)	0.59<br>(0.30,0.97)
**Philippines**	0.623	0.16<br>(0.10,0.24)	0.18<br>(0.12,0.26)	0.14<br>(-0.05,0.33)	0.19<br>(0.09,0.39)	0.22<br>(0.11,0.41)	0.14<br>(-0.11,0.39)	0.14<br>(0.09,0.21)	0.17<br>(0.10,0.24)	0.18<br>(-0.04,0.37)	0.11<br>(0.07,0.16)	0.12<br>(0.08,0.18)	0.14<br>(-0.05,0.34)
**Poland**	0.802	0.18<br>(0.12,0.27)	0.27<br>(0.17,0.41)	0.49<br>(0.28,0.74)	0.19<br>(0.11,0.34)	0.28<br>(0.15,0.51)	0.47<br>(0.25,0.73)	0.17<br>(0.11,0.23)	0.25<br>(0.16,0.35)	0.50<br>(0.30,0.73)	0.14<br>(0.09,0.20)	0.20<br>(0.13,0.28)	0.46<br>(0.25,0.71)
**Portugal**	0.743	0.18<br>(0.11,0.30)	0.29<br>(0.18,0.47)	0.58<br>(0.21,1.04)	0.19<br>(0.09,0.40)	0.30<br>(0.14,0.60)	0.57<br>(0.18,1.10)	0.17<br>(0.10,0.26)	0.28<br>(0.17,0.42)	0.66<br>(0.30,1.11)	0.13<br>(0.08,0.19)	0.23<br>(0.15,0.33)	0.74<br>(0.40,1.20)
**Puerto Rico**	0.814	0.26<br>(0.17,0.40)	0.28<br>(0.20,0.41)	0.11<br>(-0.09,0.39)	0.28<br>(0.14,0.54)	0.29<br>(0.17,0.51)	0.02<br>(-0.16,0.29)	0.24<br>(0.16,0.35)	0.29<br>(0.19,0.40)	0.19<br>(-0.04,0.52)	0.20<br>(0.13,0.28)	0.26<br>(0.17,0.36)	0.29<br>(0.05,0.65)
**Qatar**	0.830	0.30<br>(0.20,0.47)	0.30<br>(0.20,0.46)	-0.02<br>(-0.18,0.17)	0.33<br>(0.17,0.62)	0.30<br>(0.17,0.56)	-0.09<br>(-0.25,0.14)	0.28<br>(0.18,0.40)	0.29<br>(0.19,0.43)	0.04<br>(-0.14,0.28)	0.24<br>(0.15,0.36)	0.28<br>(0.18,0.41)	0.17<br>(-0.08,0.49)
**Republic of Korea**	0.878	0.14<br>(0.09,0.19)	0.22<br>(0.13,0.37)	0.59<br>(0.20,1.09)	0.14<br>(0.08,0.26)	0.24<br>(0.11,0.49)	0.63<br>(0.24,1.14)	0.13<br>(0.09,0.19)	0.20<br>(0.12,0.31)	0.54<br>(0.24,0.94)	0.12<br>(0.08,0.17)	0.16<br>(0.10,0.24)	0.38<br>(0.14,0.71)
**Republic of Moldova**	0.696	0.12<br>(0.08,0.17)	0.16<br>(0.11,0.24)	0.33<br>(0.10,0.67)	0.12<br>(0.07,0.23)	0.17<br>(0.09,0.30)	0.34<br>(0.08,0.71)	0.12<br>(0.08,0.17)	0.15<br>(0.10,0.22)	0.30<br>(0.13,0.59)	0.10<br>(0.06,0.15)	0.13<br>(0.08,0.19)	0.29<br>(0.13,0.55)
**Romania**	0.760	0.12<br>(0.08,0.18)	0.16<br>(0.10,0.24)	0.31<br>(0.14,0.56)	0.12<br>(0.07,0.22)	0.16<br>(0.09,0.29)	0.31<br>(0.13,0.58)	0.12<br>(0.08,0.17)	0.15<br>(0.10,0.22)	0.31<br>(0.14,0.55)	0.10<br>(0.07,0.15)	0.14<br>(0.09,0.20)	0.33<br>(0.15,0.58)
**Russian Federation**	0.805	0.15<br>(0.10,0.22)	0.18<br>(0.12,0.27)	0.21<br>(0.04,0.42)	0.15<br>(0.09,0.24)	0.18<br>(0.10,0.31)	0.22<br>(0.02,0.45)	0.15<br>(0.10,0.21)	0.18<br>(0.12,0.26)	0.18<br>(0.00,0.41)	0.16<br>(0.10,0.23)	0.18<br>(0.11,0.26)	0.14<br>(-0.03,0.36)

**Rwanda**	0.429	0.11<br>(0.06,0.18)	0.19<br>(0.11,0.29)	0.70<br>(0.24,1.50)	0.15<br>(0.06,0.36)	0.22<br>(0.09,0.50)	0.43<br>(0.06,1.11)	0.10<br>(0.05,0.18)	0.18<br>(0.09,0.30)	0.77<br>(0.25,1.65)	0.08<br>(0.05,0.15)	0.14<br>(0.07,0.24)	0.61<br>(0.11,1.42)
**Saint Kitts and Nevis**	0.746	0.27<br>(0.18,0.41)	0.33<br>(0.22,0.48)	0.21<br>(0.04,0.44)	0.31<br>(0.15,0.60)	0.34<br>(0.17,0.63)	0.10<br>(-0.05,0.29)	0.25<br>(0.15,0.38)	0.33<br>(0.20,0.48)	0.31<br>(0.09,0.63)	0.19<br>(0.12,0.29)	0.27<br>(0.16,0.39)	0.38<br>(0.10,0.78)
**Saint Lucia**	0.670	0.32<br>(0.21,0.47)	0.33<br>(0.23,0.47)	0.04<br>(-0.08,0.19)	0.34<br>(0.17,0.63)	0.33<br>(0.18,0.61)	-0.01<br>(-0.14,0.13)	0.32<br>(0.19,0.46)	0.34<br>(0.22,0.48)	0.09<br>(-0.06,0.29)	0.26<br>(0.16,0.37)	0.29<br>(0.18,0.41)	0.11<br>(-0.07,0.34)
**Saint Vincent and the Grenadines**	0.627	0.29<br>(0.18,0.46)	0.33<br>(0.23,0.48)	0.15<br>(-0.04,0.40)	0.32<br>(0.16,0.60)	0.34<br>(0.18,0.61)	0.06<br>(-0.08,0.23)	0.26<br>(0.16,0.39)	0.34<br>(0.21,0.49)	0.28<br>(0.08,0.54)	0.20<br>(0.12,0.29)	0.27<br>(0.16,0.39)	0.36<br>(0.13,0.67)
**Samoa**	0.641	0.26<br>(0.18,0.38)	0.35<br>(0.24,0.47)	0.33<br>(0.13,0.62)	0.29<br>(0.14,0.55)	0.36<br>(0.19,0.65)	0.22<br>(0.08,0.49)	0.26<br>(0.16,0.39)	0.35<br>(0.22,0.50)	0.35<br>(0.10,0.71)	0.20<br>(0.12,0.30)	0.30<br>(0.18,0.45)	0.49<br>(0.17,0.89)
**San Marino**	0.884	0.12<br>(0.07,0.20)	0.23<br>(0.13,0.42)	0.99<br>(0.59,1.45)	0.12<br>(0.06,0.25)	0.25<br>(0.11,0.56)	1.05<br>(0.60,1.58)	0.11<br>(0.07,0.17)	0.22<br>(0.12,0.34)	0.92<br>(0.52,1.43)	0.10<br>(0.06,0.14)	0.17<br>(0.11,0.26)	0.75<br>(0.40,1.28)
**Sao Tome and Principe**	0.502	0.13<br>(0.08,0.21)	0.21<br>(0.14,0.32)	0.59<br>(0.30,1.01)	0.16<br>(0.08,0.33)	0.24<br>(0.12,0.48)	0.54<br>(0.24,0.98)	0.12<br>(0.08,0.19)	0.19<br>(0.12,0.30)	0.59<br>(0.28,1.03)	0.08<br>(0.05,0.13)	0.14<br>(0.09,0.21)	0.62<br>(0.27,1.29)
**Saudi Arabia**	0.805	0.22<br>(0.15,0.34)	0.30<br>(0.21,0.43)	0.33<br>(0.05,0.69)	0.24<br>(0.13,0.45)	0.31<br>(0.18,0.51)	0.27<br>(-0.05,0.68)	0.21<br>(0.13,0.30)	0.29<br>(0.19,0.41)	0.39<br>(0.07,0.76)	0.17<br>(0.11,0.24)	0.23<br>(0.15,0.33)	0.40<br>(0.11,0.75)
**Senegal**	0.389	0.15<br>(0.10,0.23)	0.24<br>(0.16,0.35)	0.56<br>(0.27,0.95)	0.18<br>(0.09,0.35)	0.27<br>(0.14,0.53)	0.52<br>(0.23,0.90)	0.14<br>(0.09,0.21)	0.23<br>(0.14,0.34)	0.58<br>(0.27,0.99)	0.10<br>(0.05,0.16)	0.16<br>(0.10,0.24)	0.60<br>(0.20,1.38)
**Serbia**	0.767	0.18<br>(0.11,0.31)	0.31<br>(0.18,0.54)	0.70<br>(0.29,1.26)	0.20<br>(0.09,0.39)	0.32<br>(0.15,0.63)	0.64<br>(0.23,1.18)	0.17<br>(0.10,0.25)	0.30<br>(0.18,0.44)	0.77<br>(0.39,1.24)	0.14<br>(0.09,0.20)	0.24<br>(0.15,0.35)	0.78<br>(0.41,1.32)
**Seychelles**	0.724	0.19<br>(0.13,0.29)	0.29<br>(0.20,0.42)	0.52<br>(0.22,0.91)	0.22<br>(0.11,0.44)	0.31<br>(0.17,0.56)	0.43<br>(0.10,0.90)	0.17<br>(0.12,0.25)	0.28<br>(0.17,0.39)	0.58<br>(0.26,0.98)	0.14<br>(0.09,0.21)	0.22<br>(0.14,0.32)	0.54<br>(0.25,0.94)
**Sierra Leone**	0.347	0.06<br>(0.04,0.09)	0.10<br>(0.07,0.15)	0.62<br>(0.31,1.06)	0.07<br>(0.04,0.14)	0.12<br>(0.06,0.25)	0.64<br>(0.30,1.15)	0.06<br>(0.04,0.09)	0.10<br>(0.06,0.14)	0.60<br>(0.31,1.03)	0.04<br>(0.02,0.07)	0.07<br>(0.04,0.11)	0.53<br>(0.19,1.34)
**Singapore**	0.861	0.20<br>(0.14,0.29)	0.20<br>(0.13,0.32)	0.02<br>(-0.19,0.26)	0.21<br>(0.11,0.36)	0.21<br>(0.11,0.42)	0.02<br>(-0.21,0.31)	0.19<br>(0.13,0.27)	0.20<br>(0.13,0.30)	0.05<br>(-0.16,0.32)	0.17<br>(0.11,0.24)	0.17<br>(0.11,0.24)	0.01<br>(-0.13,0.18)

**Slovakia**	0.812	0.14 (0.09,0.21)	0.19 (0.12,0.32)	0.38 (0.12,0.74)	0.15 (0.08,0.26)	0.20 (0.11,0.38)	0.37 (0.12,0.73)	0.13 (0.09,0.18)	0.18 (0.11,0.27)	0.37 (0.14,0.71)	0.12 (0.07,0.16)	0.15 (0.10,0.22)	0.30 (0.11,0.57)
**Slovenia**	0.840	0.16 (0.10,0.28)	0.20 (0.12,0.34)	0.25 (-0.02,0.60)	0.18 (0.09,0.34)	0.21 (0.11,0.43)	0.22 (-0.06,0.59)	0.14 (0.09,0.20)	0.20 (0.12,0.31)	0.43 (0.12,0.90)	0.11 (0.07,0.16)	0.15 (0.10,0.22)	0.34 (0.13,0.64)
**Solomon Islands**	0.407	0.16 (0.11,0.21)	0.32 (0.22,0.44)	1.06 (0.68,1.52)	0.16 (0.10,0.27)	0.33 (0.19,0.57)	1.03 (0.61,1.53)	0.16 (0.10,0.22)	0.32 (0.20,0.46)	1.06 (0.66,1.52)	0.14 (0.09,0.19)	0.29 (0.17,0.42)	1.11 (0.69,1.63)
**Somalia**	0.081	0.10 (0.06,0.15)	0.15 (0.09,0.23)	0.53 (0.16,1.04)	0.12 (0.06,0.27)	0.18 (0.08,0.38)	0.43 (0.05,1.04)	0.09 (0.06,0.14)	0.14 (0.08,0.23)	0.54 (0.17,1.07)	0.08 (0.05,0.12)	0.12 (0.07,0.19)	0.47 (0.14,0.99)
**South Africa**	0.678	0.17 (0.11,0.26)	0.28 (0.18,0.43)	0.68 (0.43,1.00)	0.20 (0.10,0.40)	0.30 (0.15,0.58)	0.50 (0.27,0.79)	0.14 (0.09,0.21)	0.26 (0.16,0.38)	0.80 (0.41,1.22)	0.13 (0.08,0.20)	0.22 (0.14,0.31)	0.65 (0.35,1.05)
**South Sudan**	0.363	0.10 (0.07,0.16)	0.15 (0.10,0.24)	0.48 (0.15,0.98)	0.13 (0.06,0.29)	0.17 (0.08,0.36)	0.35 (0.01,0.85)	0.10 (0.06,0.15)	0.14 (0.08,0.24)	0.51 (0.16,1.02)	0.08 (0.05,0.12)	0.12 (0.07,0.18)	0.45 (0.15,0.94)
**Spain**	0.767	0.22 (0.13,0.41)	0.27 (0.17,0.44)	0.20 (-0.01,0.53)	0.25 (0.11,0.57)	0.29 (0.14,0.62)	0.15 (-0.03,0.45)	0.19 (0.11,0.29)	0.26 (0.16,0.41)	0.40 (0.16,0.72)	0.14 (0.09,0.21)	0.21 (0.13,0.31)	0.44 (0.18,0.80)
**Sri Lanka**	0.690	0.23 (0.14,0.41)	0.31 (0.20,0.50)	0.34 (0.18,0.59)	0.26 (0.11,0.57)	0.33 (0.17,0.64)	0.26 (0.08,0.53)	0.20 (0.11,0.32)	0.29 (0.18,0.42)	0.47 (0.24,0.83)	0.20 (0.11,0.33)	0.27 (0.17,0.39)	0.40 (0.14,0.79)
**Sudan**	0.515	0.14 (0.09,0.22)	0.31 (0.19,0.49)	1.19 (0.65,1.89)	0.16 (0.08,0.36)	0.33 (0.17,0.60)	0.99 (0.49,1.68)	0.13 (0.08,0.19)	0.27 (0.16,0.39)	1.13 (0.68,1.74)	0.11 (0.06,0.18)	0.24 (0.14,0.35)	1.21 (0.68,2.08)
**Suriname**	0.636	0.24 (0.16,0.35)	0.33 (0.24,0.46)	0.39 (0.13,0.74)	0.28 (0.14,0.55)	0.34 (0.18,0.61)	0.21 (-0.01,0.56)	0.23 (0.14,0.34)	0.34 (0.21,0.47)	0.49 (0.17,0.90)	0.17 (0.10,0.24)	0.27 (0.17,0.38)	0.62 (0.31,1.06)
**Sweden**	0.872	0.13 (0.08,0.22)	0.22 (0.13,0.39)	0.67 (0.34,1.12)	0.14 (0.07,0.28)	0.23 (0.10,0.49)	0.64 (0.29,1.14)	0.12 (0.08,0.19)	0.21 (0.12,0.34)	0.73 (0.36,1.22)	0.10 (0.07,0.15)	0.17 (0.11,0.26)	0.71 (0.36,1.26)
**Switzerland**	0.929	0.15 (0.09,0.26)	0.20 (0.12,0.37)	0.36 (0.08,0.76)	0.16 (0.08,0.34)	0.22 (0.10,0.50)	0.38 (0.06,0.83)	0.14 (0.09,0.22)	0.19 (0.12,0.33)	0.37 (0.08,0.78)	0.11 (0.07,0.16)	0.16 (0.10,0.23)	0.41 (0.19,0.73)
**Syrian Arab Republic**	0.619	0.15 (0.10,0.23)	0.29 (0.19,0.45)	0.95 (0.56,1.48)	0.18 (0.10,0.35)	0.32 (0.17,0.59)	0.79 (0.43,1.31)	0.14 (0.09,0.21)	0.27 (0.17,0.39)	0.91 (0.52,1.44)	0.06 (0.03,0.09)	0.14 (0.08,0.21)	1.48 (0.73,2.86)

**Taiwan (Province of China)**	0.868	0.15 (0.10,0.21)	0.21 (0.14,0.31)	0.40 (0.15,0.71)	0.15 (0.09,0.28)	0.23 (0.12,0.46)	0.49 (0.20,0.88)	0.14 (0.09,0.20)	0.19 (0.12,0.27)	0.33 (0.12,0.60)	0.13 (0.09,0.19)	0.17 (0.11,0.24)	0.30 (0.12,0.55)
**Tajikistan**	0.539	0.09 (0.06,0.12)	0.30 (0.18,0.43)	2.33 (1.57,3.19)	0.09 (0.05,0.16)	0.30 (0.14,0.60)	2.27 (1.40,3.19)	0.09 (0.06,0.13)	0.30 (0.16,0.46)	2.34 (1.50,3.33)	0.08 (0.05,0.12)	0.25 (0.15,0.38)	2.09 (1.32,3.09)
**Thailand**	0.687	0.12 (0.08,0.18)	0.18 (0.12,0.28)	0.50 (0.21,0.87)	0.14 (0.07,0.25)	0.23 (0.12,0.43)	0.64 (0.29,1.07)	0.12 (0.08,0.17)	0.16 (0.10,0.23)	0.37 (0.13,0.68)	0.12 (0.08,0.17)	0.16 (0.10,0.24)	0.41 (0.17,0.73)
**Timor-Leste**	0.514	0.11 (0.07,0.16)	0.25 (0.16,0.37)	1.36 (0.87,2.01)	0.13 (0.06,0.25)	0.29 (0.13,0.58)	1.30 (0.73,2.04)	0.10 (0.06,0.15)	0.23 (0.13,0.36)	1.36 (0.81,2.09)	0.08 (0.05,0.13)	0.17 (0.10,0.25)	1.04 (0.54,1.83)
**Togo**	0.417	0.08 (0.06,0.12)	0.11 (0.08,0.16)	0.35 (0.19,0.55)	0.09 (0.05,0.17)	0.12 (0.07,0.22)	0.32 (0.16,0.54)	0.08 (0.05,0.11)	0.10 (0.07,0.15)	0.37 (0.20,0.59)	0.06 (0.03,0.09)	0.08 (0.05,0.12)	0.36 (0.11,0.74)
**Tokelau**	0.626	0.23 (0.15,0.32)	0.33 (0.23,0.44)	0.46 (0.18,0.81)	0.26 (0.13,0.47)	0.34 (0.19,0.59)	0.33 (0.08,0.68)	0.22 (0.14,0.33)	0.33 (0.21,0.47)	0.50 (0.19,0.91)	0.19 (0.12,0.28)	0.28 (0.18,0.41)	0.46 (0.14,0.87)
**Tonga**	0.636	0.25 (0.17,0.35)	0.32 (0.22,0.43)	0.27 (0.05,0.54)	0.26 (0.14,0.48)	0.32 (0.17,0.56)	0.23 (0.01,0.50)	0.25 (0.16,0.36)	0.32 (0.21,0.45)	0.29 (0.05,0.60)	0.20 (0.13,0.29)	0.26 (0.17,0.38)	0.30 (0.07,0.58)
**Trinidad and Tobago**	0.757	0.34 (0.23,0.50)	0.33 (0.23,0.48)	-0.02 (-0.14,0.11)	0.34 (0.18,0.63)	0.33 (0.18,0.61)	-0.03 (-0.16,0.11)	0.35 (0.21,0.50)	0.35 (0.22,0.49)	-0.00 (-0.13,0.15)	0.27 (0.16,0.39)	0.29 (0.18,0.43)	0.08 (-0.08,0.26)
**Tunisia**	0.672	0.20 (0.12,0.35)	0.32 (0.19,0.52)	0.55 (0.25,0.95)	0.23 (0.11,0.49)	0.33 (0.17,0.61)	0.43 (0.15,0.82)	0.18 (0.11,0.28)	0.29 (0.18,0.42)	0.59 (0.26,0.98)	0.14 (0.09,0.22)	0.25 (0.16,0.36)	0.73 (0.34,1.25)
**Turkey**	0.748	0.14 (0.09,0.21)	0.22 (0.14,0.36)	0.64 (0.29,1.12)	0.15 (0.08,0.29)	0.24 (0.12,0.47)	0.60 (0.25,1.11)	0.13 (0.08,0.18)	0.21 (0.13,0.31)	0.64 (0.32,1.12)	0.11 (0.07,0.16)	0.17 (0.11,0.25)	0.53 (0.27,0.96)
**Turkmenistan**	0.670	0.09 (0.06,0.14)	0.20 (0.12,0.32)	1.13 (0.53,1.89)	0.10 (0.05,0.17)	0.22 (0.10,0.43)	1.23 (0.57,2.02)	0.09 (0.06,0.13)	0.19 (0.10,0.30)	1.05 (0.47,1.78)	0.08 (0.05,0.13)	0.16 (0.09,0.25)	0.93 (0.45,1.58)
**Tuvalu**	0.589	0.21 (0.14,0.30)	0.34 (0.23,0.45)	0.60 (0.28,1.02)	0.24 (0.13,0.45)	0.35 (0.20,0.60)	0.45 (0.17,0.86)	0.21 (0.13,0.31)	0.34 (0.21,0.48)	0.63 (0.28,1.09)	0.17 (0.11,0.26)	0.29 (0.17,0.42)	0.66 (0.26,1.20)
**Uganda**	0.404	0.13 (0.08,0.21)	0.20 (0.12,0.29)	0.48 (0.17,0.95)	0.16 (0.08,0.37)	0.22 (0.10,0.45)	0.35 (0.02,0.84)	0.12 (0.07,0.20)	0.19 (0.11,0.30)	0.53 (0.20,1.04)	0.10 (0.06,0.16)	0.15 (0.09,0.23)	0.44 (0.15,0.87)

**Ukraine**	0.736	0.11 (0.07,0.18)	0.14 (0.09,0.20)	0.18 (0.02,0.39)	0.12 (0.07,0.20)	0.14 (0.08,0.23)	0.20 (0.04,0.42)	0.11 (0.07,0.16)	0.13 (0.08,0.18)	0.17 (0.02,0.38)	0.10 (0.07,0.15)	0.12 (0.08,0.18)	0.15 (-0.01,0.35)
**United Arab Emirates**	0.880	0.30 (0.19,0.46)	0.31 (0.20,0.46)	0.01 (-0.17,0.23)	0.32 (0.17,0.56)	0.31 (0.18,0.52)	-0.03 (-0.21,0.23)	0.28 (0.18,0.41)	0.29 (0.19,0.41)	0.04 (-0.17,0.29)	0.21 (0.13,0.30)	0.24 (0.16,0.34)	0.18 (-0.07,0.50)
**United Kingdom**	0.847	0.18 (0.11,0.33)	0.29 (0.19,0.47)	0.58 (0.34,0.86)	0.20 (0.09,0.41)	0.30 (0.15,0.63)	0.50 (0.29,0.77)	0.17 (0.11,0.25)	0.29 (0.19,0.43)	0.71 (0.49,0.98)	0.13 (0.08,0.19)	0.25 (0.16,0.37)	0.94 (0.69,1.23)
**United Republic of Tanzania**	0.423	0.07 (0.05,0.10)	0.15 (0.09,0.25)	1.07 (0.51,1.79)	0.09 (0.04,0.19)	0.17 (0.07,0.38)	0.96 (0.39,1.68)	0.07 (0.04,0.10)	0.13 (0.07,0.22)	1.02 (0.51,1.77)	0.06 (0.03,0.09)	0.11 (0.06,0.17)	0.87 (0.43,1.52)
**United States of America**	0.859	0.20 (0.13,0.30)	0.28 (0.19,0.41)	0.42 (0.25,0.62)	0.21 (0.11,0.38)	0.29 (0.16,0.53)	0.41 (0.25,0.62)	0.20 (0.13,0.27)	0.28 (0.19,0.39)	0.43 (0.28,0.63)	0.17 (0.11,0.23)	0.25 (0.17,0.34)	0.49 (0.30,0.76)
**United States Virgin Islands**	0.799	0.19 (0.13,0.29)	0.32 (0.22,0.45)	0.64 (0.31,1.06)	0.22 (0.11,0.46)	0.33 (0.17,0.61)	0.46 (0.13,0.87)	0.18 (0.11,0.27)	0.32 (0.20,0.46)	0.77 (0.39,1.25)	0.15 (0.09,0.21)	0.26 (0.16,0.38)	0.79 (0.44,1.25)
**Uruguay**	0.697	0.07 (0.05,0.12)	0.18 (0.11,0.28)	1.39 (0.86,2.09)	0.08 (0.04,0.14)	0.19 (0.10,0.38)	1.48 (0.86,2.22)	0.07 (0.04,0.11)	0.18 (0.11,0.27)	1.48 (0.81,2.42)	0.07 (0.04,0.11)	0.13 (0.08,0.19)	1.00 (0.51,1.78)
**Uzbekistan**	0.631	0.08 (0.06,0.12)	0.28 (0.18,0.42)	2.41 (1.68,3.38)	0.08 (0.05,0.15)	0.29 (0.14,0.57)	2.46 (1.57,3.52)	0.08 (0.05,0.12)	0.27 (0.16,0.41)	2.38 (1.51,3.41)	0.08 (0.05,0.11)	0.23 (0.14,0.35)	2.10 (1.24,3.25)
**Vanuatu**	0.485	0.18 (0.13,0.26)	0.31 (0.21,0.42)	0.68 (0.37,1.10)	0.20 (0.11,0.35)	0.33 (0.19,0.57)	0.65 (0.28,1.10)	0.18 (0.12,0.26)	0.31 (0.20,0.43)	0.69 (0.36,1.10)	0.15 (0.10,0.22)	0.25 (0.16,0.36)	0.62 (0.33,1.00)
**Venezuela (Bolivarian Republic of)**	0.607	0.23 (0.15,0.34)	0.30 (0.20,0.43)	0.29 (0.12,0.57)	0.29 (0.13,0.58)	0.33 (0.16,0.64)	0.15 (0.02,0.37)	0.22 (0.13,0.34)	0.30 (0.18,0.44)	0.37 (0.15,0.70)	0.14 (0.09,0.21)	0.22 (0.14,0.32)	0.54 (0.27,0.91)
**Viet Nam**	0.617	0.12 (0.08,0.19)	0.24 (0.16,0.38)	1.09 (0.65,1.69)	0.14 (0.07,0.30)	0.29 (0.13,0.58)	1.03 (0.52,1.65)	0.11 (0.07,0.16)	0.22 (0.12,0.34)	1.05 (0.60,1.64)	0.09 (0.05,0.13)	0.16 (0.10,0.24)	0.83 (0.43,1.38)
**Yemen**	0.412	0.11 (0.07,0.15)	0.23 (0.13,0.37)	1.10 (0.53,1.75)	0.12 (0.07,0.22)	0.25 (0.12,0.47)	1.04 (0.54,1.60)	0.10 (0.07,0.14)	0.20 (0.11,0.30)	0.94 (0.49,1.55)	0.09 (0.06,0.14)	0.18 (0.10,0.29)	0.99 (0.49,1.64)

**Zambia**	0.505	0.12 (0.07,0.19)	0.18 (0.11,0.27)	0.49 (0.14,0.96)	0.15 (0.07,0.33)	0.19 (0.09,0.40)	0.33 (-0.01,0.83)	0.11 (0.06,0.18)	0.17 (0.10,0.27)	0.54 (0.15,1.09)	0.09 (0.06,0.15)	0.14 (0.08,0.21)	0.46 (0.08,0.99)
**Zimbabwe**	0.476	0.18 (0.11,0.32)	0.28 (0.18,0.43)	0.51 (0.22,0.96)	0.21 (0.10,0.48)	0.31 (0.15,0.60)	0.42 (0.10,0.92)	0.16 (0.10,0.24)	0.26 (0.15,0.39)	0.65 (0.28,1.15)	0.12 (0.07,0.18)	0.18 (0.11,0.26)	0.49 (0.16,0.89)

Abbreviations: ASD, age-standardized disability-adjusted life-years; PAF, population attributable fraction; SDI, sociodemographic index; DALYs, disability-adjusted life-years; HFPG, high fasting plasma glucose; UI, uncertainty interval.

Table S2. Stroke burden (DALYs, deaths, YLDs, YLLs) attributable to HFPG in 1990 and 2019, by 204 countries and territories.

Characteristic	SDI	Total Stroke ASR(per 10^5), Rate(95% UI)		Total Stroke Percentage change (95% UI)	Ischemic stroke ASR(per 10^5), Rate(95% UI)		Ischemic stroke Percentage change (95% UI)	Intracerebral hemorrhage ASR(per 10^5), Rate(95% UI)		Intracerebral hemorrhage Percentage change (95% UI)	Subarachnoid hemorrhage ASR(per 10^5), Rate(95% UI)		Subarachnoid hemorrhage Percentage change (95% UI)
		1990	2019	1990-2019	1990	2019	1990-2019	1990	2019	1990-2019	1990	2019	1990-2019
DALYs attributable to HFPG													
Afghanistan	0.343	782.56 (468.42, 1224.30)	1146.74 (695.29, 1821.28)	0.47 (0.02, 1.07)	295.56 (138.58, 603.16)	645.43 (332.72, 1187.73)	1.18 (0.57, 2.11)	420.93 (231.73, 673.34)	430.86 (233.25, 680.3)	0.02 (-0.28, 0.50)	66.07 (14.70, 146.76)	70.45 (21.02, 143.13)	0.07 (-0.30, 0.81)
Albania	0.681	325.44 (216.27, 479.38)	285.53 (174.91, 449.60)	-0.12 (-0.34, 0.17)	74.86 (40.32, 136.32)	77.01 (39.18, 138.60)	0.03 (-0.22, 0.35)	237.70 (150.49, 356.15)	196.19 (112.63, 323.1)	-0.17 (-0.38, 0.11)	12.88 (8.12, 19.55)	12.34 (7.01, 20.15)	-0.04 (-0.39, 0.41)
Algeria	0.652	506.89 (318.07, 852.77)	536.74 (315.17, 918.49)	0.06 (-0.25, 0.47)	296.93 (153.03, 613.16)	408.83 (207.37, 775.79)	0.38 (0.00, 0.92)	182.07 (111.00, 280.13)	109.19 (64.16, 169.56)	-0.40 (-0.58, -0.13)	27.89 (14.40, 44.57)	18.73 (10.74, 28.91)	-0.33 (-0.56, 0.09)
American Samoa	0.712	1064.17 (721.37, 1462.69)	790.84 (540.64, 1096.25)	-0.26 (-0.41, -0.04)	298.65 (160.47, 536.84)	237.21 (140.87, 388.78)	-0.21 (-0.37, 0.03)	698.50 (434.28, 1000.20)	490.91 (308.31, 700.74)	-0.30 (-0.45, -0.06)	67.02 (40.59, 102.53)	62.71 (35.70, 101.65)	-0.06 (-0.34, 0.34)
Andorra	0.894	78.04 (47.96, 133.05)	88.64 (50.15, 159.58)	0.14 (-0.22, 0.60)	49.42 (25.08, 97.80)	54.23 (24.28, 117.07)	0.10 (-0.24, 0.52)	18.92 (11.27, 30.85)	22.27 (11.97, 38.75)	0.18 (-0.27, 0.78)	9.71 (5.55, 16.51)	12.13 (6.91, 19.55)	0.25 (-0.18, 0.88)
Angola	0.470	527.42 (315.51, 810.25)	559.66 (343.72, 845.18)	0.06 (-0.24, 0.55)	165.04 (74.86, 351.71)	227.60 (106.21, 461.00)	0.38 (-0.03, 1.06)	348.71 (191.65, 562.52)	317.70 (167.59, 505.05)	-0.09 (-0.36, 0.34)	13.67 (4.82, 31.63)	14.36 (6.21, 29.64)	0.05 (-0.35, 0.92)

**Antigua and Barbuda**	0.743	535.57 (342.07, 794.77)	435.27 (288.27, 650.33)	-0.19 (-0.36, 0.05)	223.53 (108.29, 437.10)	190.80 (95.24, 356.13)	-0.15 (-0.32, 0.10)	277.00 (160.70, 426.20)	208.23 (120.30, 310.66)	-0.25 (-0.43, 0.00)	35.04 (21.09, 53.16)	36.23 (21.78, 54.32)	0.03 (-0.24, 0.44)
**Argentina**	0.708	275.85 (187.02, 390.19)	213.09 (140.00, 318.64)	-0.23 (-0.39, -0.02)	103.01 (55.72, 187.20)	91.84 (43.72, 175.41)	-0.11 (-0.33, 0.19)	140.54 (92.27, 199.42)	97.23 (59.65, 147.56)	-0.31 (-0.47, -0.08)	32.29 (19.53, 51.50)	24.02 (14.99, 34.83)	-0.26 (-0.42, 0.01)
**Armenia**	0.689	308.35 (180.71, 552.04)	351.16 (208.32, 588.83)	0.14 (-0.19, 0.64)	189.22 (89.55, 384.68)	222.44 (106.26, 448.15)	0.18 (-0.18, 0.73)	101.02 (56.68, 169.54)	101.42 (55.58, 160.27)	0.00 (-0.32, 0.50)	18.11 (10.12, 30.83)	27.30 (15.66, 41.09)	0.51 (-0.02, 1.34)
**Australia**	0.839	111.87 (65.16, 199.57)	84.06 (47.21, 150.26)	-0.25 (-0.41, -0.06)	77.31 (38.37, 164.64)	51.73 (23.81, 116.22)	-0.33 (-0.49, -0.15)	25.67 (15.93, 38.88)	22.79 (12.48, 36.94)	-0.11 (-0.32, 0.15)	8.88 (5.59, 13.39)	9.54 (5.85, 14.10)	0.07 (-0.14, 0.40)
**Austria**	0.849	142.89 (84.80, 249.14)	99.50 (58.18, 176.78)	-0.30 (-0.45, -0.13)	99.63 (48.94, 201.22)	61.99 (28.37, 128.99)	-0.38 (-0.52, -0.22)	34.63 (21.92, 50.72)	25.29 (13.95, 40.37)	-0.27 (-0.46, -0.05)	8.63 (5.47, 12.98)	12.23 (7.50, 18.57)	0.42 (0.09, 0.90)
**Azerbaijan**	0.683	253.87 (173.00, 362.08)	808.75 (500.45, 1247.15)	2.19 (1.28, 3.32)	75.69 (42.26, 135.31)	295.88 (138.65, 598.46)	2.91 (1.64, 4.48)	171.66 (106.51, 251.87)	499.30 (271.09, 828.22)	1.91 (1.06, 3.13)	6.53 (3.68, 10.75)	13.57 (7.28, 23.21)	1.08 (0.35, 2.16)
**Bahamas**	0.796	385.36 (256.50, 570.93)	393.29 (253.89, 574.13)	0.02 (-0.23, 0.36)	168.34 (83.71, 324.94)	164.35 (83.74, 298.59)	-0.02 (-0.26, 0.32)	194.52 (117.23, 297.86)	200.13 (117.96, 296.50)	0.03 (-0.25, 0.42)	22.49 (13.81, 33.93)	28.82 (17.32, 43.58)	0.28 (-0.10, 0.82)
**Bahrain**	0.751	618.96 (394.53, 974.07)	289.49 (179.88, 474.58)	-0.53 (-0.64, -0.40)	366.36 (179.78, 682.31)	193.44 (101.06, 355.48)	-0.47 (-0.60, -0.32)	224.10 (128.06, 331.05)	80.65 (48.80, 123.82)	-0.64 (-0.73, -0.50)	28.50 (16.32, 47.80)	15.40 (8.99, 25.83)	-0.46 (-0.67, -0.18)
**Bangladesh**	0.483	416.35 (280.61, 600.40)	478.52 (292.38, 736.20)	0.15 (-0.16, 0.58)	118.34 (59.17, 236.66)	181.35 (83.19, 364.59)	0.53 (0.07, 1.17)	251.92 (155.17, 368.77)	251.63 (142.75, 402.71)	0.00 (-0.29, 0.40)	46.09 (14.35, 93.16)	45.53 (19.40, 84.45)	-0.01 (-0.35, 1.02)

**Barbados**	0.742	430.12 (276.29, 679.97)	336.67 (215.32, 506.55)	-0.22 (-0.41, 0.04)	246.25 (125.49, 479.20)	181.35 (90.54, 324.05)	-0.26 (-0.45, -0.01)	154.82 (100.08, 223.47)	126.70 (74.77, 186.89)	-0.18 (-0.41, 0.12)	29.05 (18.64, 42.50)	28.62 (17.71, 41.44)	-0.01 (-0.30, 0.39)
**Belarus**	0.745	287.95 (188.94, 439.55)	268.95 (168.55, 421.05)	-0.07 (-0.26, 0.19)	187.85 (103.08, 321.47)	176.81 (96.76, 303.32)	-0.06 (-0.27, 0.22)	80.41 (51.99, 116.97)	71.91 (41.57, 112.40)	-0.11 (-0.34, 0.20)	19.69 (11.43, 30.96)	20.22 (11.21, 32.57)	0.03 (-0.23, 0.42)
**Belgium**	0.851	153.62 (96.30, 253.34)	119.16 (70.77, 203.37)	-0.22 (-0.41, -0.01)	97.70 (49.65, 188.34)	66.95 (30.81, 141.90)	-0.31 (-0.48, -0.10)	46.44 (29.66, 66.47)	39.55 (22.76, 63.65)	-0.15 (-0.36, 0.14)	9.47 (5.94, 14.13)	12.66 (7.98, 18.90)	0.34 (0.02, 0.79)
**Belize**	0.603	212.61 (146.42, 309.57)	278.92 (179.21, 406.56)	0.31 (0.00, 0.73)	86.80 (46.19, 159.38)	115.77 (60.47, 216.98)	0.33 (-0.01, 0.83)	111.16 (72.19, 157.51)	143.13 (83.37, 221.04)	0.29 (-0.03, 0.73)	14.65 (9.17, 22.28)	20.02 (12.05, 29.35)	0.37 (0.03, 0.86)
**Benin**	0.352	335.93 (210.02, 538.42)	536.89 (318.34, 837.39)	0.60 (0.16, 1.22)	116.74 (55.91, 249.28)	187.27 (87.79, 389.86)	0.60 (0.13, 1.32)	212.19 (122.65, 342.41)	340.24 (182.33, 552.46)	0.60 (0.13, 1.34)	6.99 (2.62, 14.79)	9.38 (3.60, 20.77)	0.34 (-0.09, 1.00)
**Bermuda**	0.813	211.03 (136.72, 330.99)	132.97 (82.28, 211.95)	-0.37 (-0.52, -0.18)	121.09 (64.74, 238.87)	78.58 (37.73, 150.80)	-0.35 (-0.51, -0.14)	73.66 (47.62, 112.25)	41.17 (24.49, 63.49)	-0.44 (-0.60, -0.22)	16.28 (10.13, 25.46)	13.22 (7.96, 20.07)	-0.19 (-0.42, 0.16)
**Bhutan**	0.455	242.44 (150.07, 369.17)	294.39 (176.10, 470.70)	0.21 (-0.18, 0.81)	74.77 (37.57, 155.11)	131.32 (60.26, 256.43)	0.76 (0.12, 1.61)	139.88 (78.18, 220.63)	139.44 (76.47, 230.48)	0.00 (-0.35, 0.59)	27.79 (9.22, 57.03)	23.64 (11.67, 42.17)	-0.15 (-0.50, 0.79)
**Bolivia (Plurinational State of)**	0.566	297.02 (193.26, 443.22)	300.06 (179.41, 476.37)	0.01 (-0.28, 0.43)	99.72 (50.97, 188.87)	127.31 (58.08, 245.34)	0.28 (-0.12, 0.82)	150.58 (91.73, 234.80)	125.70 (69.37, 210.78)	-0.17 (-0.44, 0.21)	46.73 (21.83, 83.30)	47.05 (25.28, 75.92)	0.01 (-0.35, 0.67)
**Bosnia and Herzegovina**	0.718	434.26 (260.42, 732.70)	734.80 (426.57, 1266.62)	0.69 (0.23, 1.31)	323.49 (167.72, 607.60)	584.49 (292.40, 1094.23)	0.81 (0.29, 1.49)	75.74 (46.07, 113.77)	108.44 (60.71, 170.25)	0.43 (0.02, 1.01)	35.03 (18.75, 57.54)	41.87 (23.80, 66.26)	0.20 (-0.22, 1.03)

**Botswana**	0.634	562.57 (331.54, 913.27)	760.21 (445.85, 1220.50)	0.35 (-0.06, 1.01)	265.11 (126.75, 570.97)	394.88 (174.64, 767.40)	0.49 (0.03, 1.27)	286.14 (162.62, 452.43)	353.30 (184.89, 576.57)	0.23 (-0.20, 0.87)	11.32 (5.99, 20.34)	12.03 (5.85, 23.13)	0.06 (-0.39, 0.79)
**Brazil**	0.640	584.82 (383.44, 921.20)	281.73 (181.92, 423.92)	-0.52 (-0.56, -0.47)	310.51 (151.94, 615.78)	149.60 (72.22, 284.90)	-0.52 (-0.57, -0.46)	237.36 (147.20, 338.08)	103.13 (63.57, 151.15)	-0.57 (-0.62, -0.51)	36.95 (21.88, 55.34)	29.00 (17.97, 41.68)	-0.22 (-0.33, -0.07)
**Brunei Darussalam**	0.823	1158.03 (793.82, 1639.35)	526.59 (356.96, 788.57)	-0.55 (-0.63, -0.44)	515.32 (249.15, 941.07)	254.92 (129.02, 472.70)	-0.51 (-0.60, -0.37)	507.51 (303.31, 750.83)	199.23 (120.05, 291.66)	-0.61 (-0.69, -0.49)	135.19 (79.01, 196.56)	72.44 (45.03, 104.52)	-0.46 (-0.59, -0.27)
**Bulgaria**	0.764	647.38 (427.71, 970.26)	783.86 (445.49, 1378.56)	0.21 (-0.14, 0.64)	330.40 (173.27, 628.62)	520.51 (240.13, 1054.46)	0.58 (0.13, 1.12)	298.34 (193.86, 432.20)	233.01 (133.21, 374.51)	-0.22 (-0.45, 0.09)	18.65 (11.62, 27.25)	30.34 (18.09, 47.16)	0.63 (0.17, 1.27)
**Burkina Faso**	0.257	226.32 (134.43, 364.54)	418.82 (253.97, 659.47)	0.85 (0.29, 1.66)	69.96 (30.99, 158.95)	116.72 (51.84, 234.05)	0.67 (0.18, 1.42)	151.26 (82.47, 253.95)	294.49 (152.31, 500.08)	0.95 (0.33, 1.98)	5.10 (1.89, 11.73)	7.61 (3.03, 17.08)	0.49 (0.02, 1.39)
**Burundi**	0.284	451.90 (276.97, 705.09)	381.77 (223.24, 615.93)	-0.16 (-0.42, 0.26)	118.50 (53.00, 265.91)	126.06 (52.45, 265.46)	0.06 (-0.26, 0.55)	320.00 (184.35, 497.19)	242.83 (125.80, 409.29)	-0.24 (-0.49, 0.17)	13.40 (4.38, 33.36)	12.88 (3.84, 39.29)	-0.04 (-0.45, 0.59)
**Cabo Verde**	0.525	190.57 (128.11, 277.45)	397.59 (247.18, 607.26)	1.09 (0.53, 1.96)	55.09 (30.07, 105.91)	155.40 (73.37, 306.04)	1.82 (0.99, 2.94)	130.47 (82.85, 191.92)	235.75 (136.92, 385.01)	0.81 (0.29, 1.67)	5.02 (2.13, 10.45)	6.45 (3.21, 12.54)	0.29 (-0.17, 1.26)
**Cambodia**	0.469	360.79 (241.71, 525.20)	852.32 (536.86, 1276.52)	1.36 (0.66, 2.14)	109.99 (54.43, 213.57)	316.29 (139.08, 644.75)	1.88 (0.94, 2.89)	230.02 (145.19, 349.76)	501.36 (278.66, 801.58)	1.18 (0.48, 1.98)	20.77 (10.01, 36.16)	34.68 (19.02, 57.84)	0.67 (0.05, 1.75)
**Cameroon**	0.490	262.07 (171.92, 392.30)	370.55 (226.90, 562.46)	0.41 (0.01, 1.01)	79.05 (39.46, 149.20)	119.82 (59.64, 227.42)	0.52 (0.07, 1.23)	177.61 (110.95, 269.65)	244.34 (140.66, 389.14)	0.38 (-0.03, 0.97)	5.41 (2.31, 10.98)	6.40 (2.71, 13.91)	0.18 (-0.21, 0.89)

**Canada**	0.873	83.62 (50.01, 147.65)	93.69 (53.58, 170.17)	0.12 (-0.15, 0.45)	55.78 (26.76, 115.51)	58.58 (25.24, 129.39)	0.05 (-0.23, 0.39)	18.93 (11.45, 28.70)	23.50 (12.70, 37.95)	0.24 (-0.09, 0.63)	8.90 (5.69, 13.39)	11.61 (6.83, 17.69)	0.30 (-0.03, 0.75)
**Central African Republic**	0.274	602.85 (379.05, 925.22)	854.75 (498.63, 1295.21)	0.42 (0.02, 1.04)	160.56 (78.34, 322.77)	260.67 (117.99, 536.97)	0.62 (0.15, 1.30)	425.10 (254.73, 651.51)	566.62 (301.62, 920.83)	0.33 (-0.04, 0.96)	17.19 (5.84, 37.55)	27.46 (8.00, 71.72)	0.60 (-0.01, 1.39)
**Chad**	0.238	266.31 (178.41, 392.06)	444.67 (273.16, 689.10)	0.67 (0.24, 1.26)	82.12 (43.53, 158.19)	139.98 (65.36, 283.79)	0.70 (0.22, 1.32)	177.60 (110.80, 264.66)	296.15 (173.72, 470.67)	0.67 (0.22, 1.31)	6.59 (2.56, 13.39)	8.54 (3.38, 17.62)	0.30 (-0.06, 0.90)
**Chile**	0.759	289.42 (180.71, 452.10)	241.45 (154.46, 383.24)	-0.17 (-0.34, 0.08)	148.44 (72.97, 300.45)	133.33 (63.91, 262.43)	-0.10 (-0.30, 0.22)	121.46 (74.99, 179.12)	81.50 (48.65, 121.32)	-0.33 (-0.48, -0.12)	19.52 (12.06, 28.79)	26.62 (16.14, 38.81)	0.36 (0.02, 0.84)
**China**	0.686	576.95 (387.33, 834.76)	385.97 (256.13, 577.25)	-0.33 (-0.47, -0.15)	182.62 (95.40, 341.47)	192.34 (99.57, 354.40)	0.05 (-0.17, 0.30)	308.23 (190.91, 454.95)	175.78 (111.27, 265.97)	-0.43 (-0.55, -0.29)	86.10 (46.19, 134.76)	17.86 (10.88, 26.55)	-0.79 (-0.86, -0.62)
**Colombia**	0.633	406.03 (265.74, 615.13)	181.97 (114.17, 278.26)	-0.55 (-0.66, -0.40)	203.46 (94.32, 389.32)	79.35 (37.49, 152.95)	-0.61 (-0.70, -0.50)	167.20 (98.64, 253.17)	63.05 (35.22, 100.20)	-0.62 (-0.72, -0.49)	35.37 (21.81, 52.46)	39.57 (23.01, 62.91)	0.12 (-0.25, 0.60)
**Comoros**	0.455	287.17 (160.27, 466.69)	311.69 (174.30, 534.55)	0.09 (-0.29, 0.74)	104.95 (47.14, 234.72)	136.68 (55.20, 312.74)	0.30 (-0.14, 1.05)	173.35 (92.51, 282.98)	165.32 (80.79, 288.73)	-0.05 (-0.40, 0.67)	8.87 (2.78, 20.30)	9.69 (3.03, 22.94)	0.09 (-0.37, 1.23)
**Congo**	0.568	643.46 (401.45, 973.35)	619.12 (391.18, 948.26)	-0.04 (-0.29, 0.33)	215.29 (105.51, 428.50)	267.30 (127.46, 542.48)	0.24 (-0.10, 0.70)	413.82 (244.43, 633.81)	337.40 (190.54, 528.65)	-0.18 (-0.41, 0.14)	14.35 (6.37, 32.94)	14.41 (6.50, 30.97)	0.00 (-0.36, 0.58)
**Cook Islands**	0.764	643.84 (427.25, 915.61)	505.42 (351.57, 710.95)	-0.21 (-0.41, 0.06)	221.05 (113.72, 404.28)	194.39 (112.78, 331.23)	-0.12 (-0.33, 0.20)	380.15 (235.69, 582.13)	277.70 (170.67, 404.07)	-0.27 (-0.47, 0.03)	42.64 (25.85, 65.19)	33.33 (18.38, 53.42)	-0.22 (-0.46, 0.19)

**Costa Rica**	0.680	229.45 (144.80, 358.79)	159.25 (100.57, 248.83)	-0.31 (-0.47, -0.08)	117.04 (55.39, 239.57)	79.90 (40.01, 152.72)	-0.32 (-0.48, -0.10)	88.25 (54.21, 132.40)	55.54 (32.50, 87.24)	-0.37 (-0.55, -0.14)	24.16 (14.93, 35.75)	23.81 (14.09, 37.07)	-0.01 (-0.28, 0.36)
**Croatia**	0.794	525.78 (324.36, 873.88)	385.28 (228.44, 660.77)	-0.27 (-0.45, 0.00)	331.01 (161.35, 668.11)	256.50 (119.30, 511.81)	-0.23 (-0.43, 0.05)	168.22 (103.91, 246.38)	101.90 (57.20, 160.38)	-0.39 (-0.57, -0.15)	26.55 (16.36, 38.87)	26.87 (15.92, 41.67)	0.01 (-0.25, 0.41)
**Cuba**	0.668	304.63 (198.63, 457.56)	280.15 (174.74, 436.81)	-0.08 (-0.31, 0.22)	157.82 (82.12, 293.99)	162.29 (80.61, 301.20)	0.03 (-0.23, 0.38)	125.78 (81.30, 181.01)	99.45 (57.98, 150.03)	-0.21 (-0.43, 0.09)	21.03 (12.68, 31.29)	18.41 (11.24, 27.33)	-0.12 (-0.36, 0.26)
**Cyprus**	0.841	505.16 (292.31, 910.46)	193.58 (112.51, 349.21)	-0.62 (-0.68, -0.54)	301.66 (128.64, 700.52)	115.44 (50.37, 262.06)	-0.62 (-0.69, -0.54)	163.68 (90.94, 257.31)	59.70 (33.98, 96.09)	-0.64 (-0.70, -0.55)	39.82 (21.74, 64.79)	18.44 (11.44, 27.83)	-0.54 (-0.67, -0.33)
**Czechia**	0.828	770.15 (431.88, 1403.03)	296.20 (181.54, 481.44)	-0.62 (-0.70, -0.49)	595.23 (282.49, 1221.49)	209.18 (106.86, 391.15)	-0.65 (-0.73, -0.53)	143.53 (88.01, 218.86)	59.89 (36.58, 89.62)	-0.58 (-0.69, -0.44)	31.39 (19.44, 45.67)	27.12 (16.59, 40.88)	-0.14 (-0.35, 0.17)
**Côte d'Ivoire**	0.408	284.41 (185.64, 454.75)	442.99 (264.94, 682.81)	0.56 (0.12, 1.16)	103.03 (51.96, 202.66)	164.95 (75.58, 331.09)	0.60 (0.13, 1.26)	176.26 (106.87, 278.71)	271.11 (151.30, 434.07)	0.54 (0.06, 1.16)	5.12 (2.20, 10.98)	6.93 (3.01, 13.42)	0.35 (-0.10, 1.08)
**Democratic People's Republic of Korea**	0.558	482.74 (321.00, 704.69)	596.71 (396.59, 897.96)	0.24 (-0.04, 0.62)	162.46 (92.54, 282.89)	215.18 (120.50, 386.74)	0.32 (0.03, 0.77)	289.43 (178.01, 433.78)	354.44 (214.94, 531.81)	0.22 (-0.07, 0.62)	30.84 (17.37, 47.55)	27.10 (14.54, 46.69)	-0.12 (-0.40, 0.29)
**Democratic Republic of the Congo**	0.382	450.48 (292.76, 690.09)	525.53 (321.66, 822.11)	0.17 (-0.17, 0.65)	141.84 (71.20, 295.20)	185.08 (84.70, 377.90)	0.30 (-0.07, 0.84)	294.93 (179.74, 442.80)	322.37 (183.15, 519.35)	0.09 (-0.24, 0.58)	13.71 (5.31, 31.31)	18.08 (6.24, 46.84)	0.32 (-0.19, 1.14)
**Denmark**	0.890	119.05 (74.41, 195.65)	109.29 (65.05, 183.44)	-0.08 (-0.30, 0.21)	70.27 (36.76, 136.42)	65.58 (30.54, 134.60)	-0.07 (-0.32, 0.24)	35.80 (23.03, 51.66)	30.96 (17.49, 50.42)	-0.14 (-0.36, 0.18)	12.98 (7.83, 20.30)	12.75 (7.62, 19.18)	-0.02 (-0.26, 0.36)

**Djibouti**	0.459	303.14 (182.19, 486.42)	376.24 (217.42, 627.80)	0.24 (-0.14, 0.86)	111.50 (48.07, 240.16)	176.48 (80.64, 394.92)	0.58 (0.07, 1.40)	182.06 (102.32, 293.92)	188.73 (98.06, 312.39)	0.04 (-0.32, 0.59)	9.57 (3.77, 18.24)	11.02 (3.87, 22.94)	0.15 (-0.33, 0.98)
**Dominica**	0.729	454.64 (292.68, 681.05)	459.59 (302.99, 679.86)	0.01 (-0.23, 0.34)	215.25 (101.88, 403.32)	224.40 (115.18, 404.85)	0.04 (-0.17, 0.38)	209.01 (121.39, 330.85)	199.14 (117.31, 296.78)	-0.05 (-0.32, 0.35)	30.39 (17.98, 46.45)	36.05 (20.69, 55.95)	0.19 (-0.16, 0.67)
**Dominican Republic**	0.592	147.70 (98.26, 217.90)	361.86 (210.39, 602.68)	1.45 (0.65, 2.51)	57.25 (30.57, 109.20)	162.29 (72.13, 328.37)	1.83 (0.88, 3.09)	81.59 (50.76, 119.30)	179.10 (93.70, 306.72)	1.20 (0.45, 2.27)	8.86 (5.18, 14.15)	20.47 (11.31, 34.86)	1.31 (0.44, 2.66)
**Ecuador**	0.640	210.10 (134.74, 331.60)	220.64 (136.29, 348.38)	0.05 (-0.22, 0.46)	91.89 (45.21, 198.77)	108.50 (51.41, 221.81)	0.18 (-0.13, 0.62)	88.84 (53.68, 130.42)	75.48 (43.74, 118.71)	-0.15 (-0.39, 0.21)	29.38 (16.75, 45.99)	36.66 (22.09, 56.62)	0.25 (-0.16, 0.91)
**Egypt**	0.658	226.59 (144.11, 365.44)	526.74 (295.21, 920.94)	1.32 (0.50, 2.33)	121.89 (59.43, 240.32)	388.95 (186.85, 733.84)	2.19 (1.05, 3.60)	91.30 (55.61, 141.46)	124.00 (62.65, 227.74)	0.36 (-0.15, 1.10)	13.40 (5.56, 24.50)	13.79 (6.88, 23.98)	0.03 (-0.46, 1.16)
**El Salvador**	0.573	239.19 (157.66, 352.69)	211.90 (132.42, 338.38)	-0.11 (-0.39, 0.31)	82.14 (39.23, 166.33)	90.97 (44.63, 178.88)	0.11 (-0.24, 0.66)	127.41 (79.44, 183.37)	94.78 (52.50, 152.90)	-0.26 (-0.50, 0.14)	29.64 (17.06, 47.21)	26.15 (14.70, 42.28)	-0.12 (-0.47, 0.48)
**Equatorial Guinea**	0.685	557.58 (344.94, 868.69)	453.73 (269.73, 710.26)	-0.19 (-0.48, 0.25)	159.46 (77.34, 326.93)	206.78 (97.67, 411.02)	0.30 (-0.19, 0.93)	382.23 (217.63, 598.92)	237.42 (121.05, 400.60)	-0.38 (-0.62, -0.01)	15.89 (5.53, 36.97)	9.54 (4.62, 17.59)	-0.40 (-0.70, 0.46)
**Eritrea**	0.396	339.73 (208.87, 548.70)	427.12 (258.82, 690.81)	0.26 (-0.15, 0.89)	81.20 (36.97, 176.68)	139.91 (63.57, 285.32)	0.72 (0.08, 1.74)	245.74 (144.81, 385.38)	273.53 (150.15, 449.87)	0.11 (-0.26, 0.67)	12.78 (3.98, 29.07)	13.69 (5.29, 30.10)	0.07 (-0.34, 0.99)
**Estonia**	0.835	370.72 (237.55, 570.16)	120.94 (75.36, 187.15)	-0.67 (-0.74, -0.57)	272.12 (150.76, 467.95)	82.40 (44.86, 144.57)	-0.70 (-0.76, -0.60)	79.29 (51.88, 113.11)	24.85 (14.92, 37.34)	-0.69 (-0.76, -0.58)	19.32 (11.16, 30.15)	13.68 (7.96, 21.02)	-0.29 (-0.47, 0.01)

**Eswatini**	0.577	559.04 (373.25, 842.05)	739.95 (450.56, 1128.28)	0.32 (-0.08, 0.88)	232.67 (118.44, 457.53)	345.96 (158.65, 639.25)	0.49 (0.04, 1.16)	313.93 (198.37, 461.03)	379.75 (210.36, 597.20)	0.21 (-0.20, 0.78)	12.44 (6.82, 22.09)	14.23 (7.71, 24.89)	0.14 (-0.27, 0.82)
**Ethiopia**	0.343	384.86 (243.19, 577.29)	202.36 (126.51, 312.64)	-0.47 (-0.63, -0.26)	68.53 (32.72, 148.27)	61.47 (29.93, 123.36)	-0.10 (-0.40, 0.27)	300.09 (182.55, 455.76)	132.17 (75.09, 198.11)	-0.56 (-0.70, -0.38)	16.24 (4.88, 38.81)	8.72 (3.02, 20.99)	-0.46 (-0.64, 0.17)
**Fiji**	0.664	1028.24 (673.39, 1419.66)	795.55 (539.08, 1146.38)	-0.23 (-0.43, 0.09)	341.39 (181.61, 620.48)	305.93 (174.91, 523.44)	-0.10 (-0.34, 0.29)	566.46 (342.45, 823.23)	391.93 (243.92, 588.78)	-0.31 (-0.51, 0.04)	120.38 (73.81, 183.69)	97.68 (57.73, 149.44)	-0.19 (-0.45, 0.23)
**Finland**	0.856	264.28 (161.45, 455.00)	181.78 (109.66, 311.63)	-0.31 (-0.45, -0.13)	178.86 (90.28, 368.19)	115.67 (55.02, 236.98)	-0.35 (-0.49, -0.17)	54.29 (35.13, 78.95)	43.59 (26.10, 65.94)	-0.20 (-0.40, 0.06)	31.12 (17.77, 48.38)	22.52 (14.50, 32.65)	-0.28 (-0.45, 0.05)
**France**	0.834	92.61 (58.04, 150.15)	52.74 (33.57, 83.28)	-0.43 (-0.51, -0.32)	54.60 (28.19, 104.53)	29.80 (15.64, 57.62)	-0.45 (-0.54, -0.33)	31.03 (20.02, 45.92)	15.76 (10.15, 23.45)	-0.49 (-0.58, -0.39)	6.98 (4.30, 10.70)	7.19 (4.58, 10.47)	0.03 (-0.13, 0.25)
**Gabon**	0.656	554.50 (356.49, 839.13)	573.06 (359.28, 862.31)	0.03 (-0.25, 0.36)	195.54 (96.20, 388.47)	253.06 (122.08, 470.36)	0.29 (-0.04, 0.75)	347.25 (200.84, 550.17)	306.98 (174.27, 501.61)	-0.12 (-0.38, 0.21)	11.72 (5.68, 25.22)	13.01 (6.61, 24.87)	0.11 (-0.30, 0.79)
**Gambia**	0.399	218.52 (138.01, 335.17)	432.85 (258.78, 663.12)	0.98 (0.37, 1.83)	80.55 (41.34, 154.86)	163.59 (74.82, 327.56)	1.03 (0.43, 1.79)	133.46 (80.28, 207.36)	262.08 (140.97, 414.53)	0.96 (0.29, 1.92)	4.51 (1.84, 9.61)	7.18 (2.92, 15.15)	0.59 (0.02, 1.41)
**Georgia**	0.702	459.51 (308.25, 656.24)	931.67 (608.21, 1406.09)	1.03 (0.56, 1.61)	164.86 (84.54, 309.18)	377.08 (182.13, 749.05)	1.29 (0.68, 2.03)	268.07 (171.03, 394.17)	460.29 (264.43, 705.16)	0.72 (0.28, 1.25)	26.58 (16.66, 40.16)	94.31 (56.14, 141.64)	2.55 (1.55, 3.78)
**Germany**	0.898	370.19 (204.54, 667.38)	166.08 (100.68, 281.37)	-0.55 (-0.61, -0.45)	263.56 (116.42, 555.84)	108.09 (50.71, 220.81)	-0.59 (-0.64, -0.50)	84.48 (46.61, 133.10)	40.87 (23.56, 61.48)	-0.52 (-0.60, -0.39)	22.16 (13.28, 34.06)	17.11 (10.35, 24.76)	-0.23 (-0.39, 0.01)

**Ghana**	0.557	383.06 (249.48, 609.48)	596.52 (370.44, 928.24)	0.56 (0.14, 1.16)	165.37 (81.35, 343.73)	289.56 (137.50, 569.51)	0.75 (0.26, 1.47)	210.86 (126.15, 332.70)	297.82 (172.71, 474.25)	0.41 (0.02, 1.01)	6.82 (2.96, 14.00)	9.14 (4.52, 16.47)	0.34 (-0.06, 1.01)
**Greece**	0.794	269.42 (168.54, 441.58)	205.55 (125.08, 342.61)	-0.24 (-0.41, -0.04)	156.98 (82.07, 312.73)	108.32 (48.50, 238.18)	-0.31 (-0.48, -0.09)	104.11 (67.34, 154.23)	84.64 (50.09, 133.33)	-0.19 (-0.37, 0.03)	8.33 (5.42, 12.05)	12.59 (7.88, 18.81)	0.51 (0.18, 0.91)
**Greenland**	0.761	233.64 (152.81, 356.76)	367.79 (221.60, 609.91)	0.57 (0.17, 1.09)	115.10 (57.05, 223.66)	181.49 (80.02, 390.47)	0.58 (0.14, 1.11)	87.83 (54.76, 131.46)	142.77 (75.57, 223.31)	0.63 (0.14, 1.25)	30.72 (18.33, 48.77)	43.53 (23.77, 69.75)	0.42 (-0.04, 1.04)
**Grenada**	0.669	869.90 (560.34, 1318.42)	609.96 (406.56, 899.10)	-0.30 (-0.43, -0.11)	448.63 (217.48, 875.42)	310.75 (156.09, 580.55)	-0.31 (-0.44, -0.10)	373.71 (215.77, 570.37)	255.91 (151.12, 372.10)	-0.32 (-0.48, -0.08)	47.56 (28.21, 74.21)	43.30 (25.98, 64.10)	-0.09 (-0.34, 0.25)
**Guam**	0.813	376.02 (248.82, 567.65)	366.33 (246.13, 523.24)	-0.03 (-0.25, 0.28)	165.46 (87.85, 325.40)	162.91 (91.45, 283.30)	-0.02 (-0.26, 0.32)	179.99 (113.93, 268.19)	173.87 (107.69, 257.58)	-0.03 (-0.28, 0.31)	30.57 (19.32, 46.05)	29.55 (17.11, 44.14)	-0.03 (-0.30, 0.29)
**Guatemala**	0.526	242.11 (152.07, 372.89)	288.57 (180.48, 431.33)	0.19 (-0.11, 0.64)	104.73 (46.10, 213.29)	108.17 (53.52, 208.79)	0.03 (-0.26, 0.53)	124.73 (74.99, 192.73)	154.77 (87.87, 237.34)	0.24 (-0.12, 0.79)	12.65 (7.10, 20.65)	25.62 (14.08, 40.40)	1.02 (0.31, 2.17)
**Guinea**	0.325	225.72 (149.61, 344.69)	437.49 (268.11, 698.12)	0.94 (0.39, 1.72)	69.06 (36.71, 137.94)	143.21 (64.02, 290.26)	1.07 (0.45, 1.89)	150.54 (92.92, 231.47)	285.69 (160.62, 463.40)	0.90 (0.32, 1.74)	6.12 (2.13, 12.93)	8.59 (3.25, 18.86)	0.40 (-0.06, 1.18)
**Guinea-Bissau**	0.355	377.54 (247.82, 576.83)	608.91 (373.81, 942.07)	0.61 (0.12, 1.36)	106.56 (55.61, 214.77)	199.07 (92.30, 390.18)	0.87 (0.27, 1.71)	263.46 (163.56, 410.16)	399.57 (225.17, 651.19)	0.52 (0.03, 1.26)	7.53 (2.89, 15.84)	10.27 (4.14, 22.59)	0.36 (-0.12, 1.20)
**Guyana**	0.618	1883.37 (1223.94, 2675.49)	1148.81 (739.89, 1689.27)	-0.39 (-0.56, -0.16)	711.66 (340.60, 1290.66)	475.40 (247.77, 849.16)	-0.33 (-0.50, -0.08)	1104.96 (647.50, 1660.78)	610.20 (340.92, 942.51)	-0.45 (-0.62, -0.21)	66.75 (39.23, 103.13)	63.21 (36.09, 99.81)	-0.05 (-0.36, 0.42)

**Haiti**	0.432	1118.01 (664.57, 1738.27)	1149.69 (655.64, 1821.12)	0.03 (-0.24, 0.44)	381.02 (179.34, 802.55)	430.95 (196.23, 873.78)	0.13 (-0.17, 0.59)	631.97 (331.52, 975.59)	610.81 (301.58, 1007.90)	-0.03 (-0.31, 0.38)	105.02 (35.28, 214.09)	107.92 (41.11, 226.42)	0.03 (-0.35, 0.73)
**Honduras**	0.496	392.29 (249.20, 605.54)	652.82 (410.10, 983.40)	0.66 (0.23, 1.29)	175.02 (83.67, 332.20)	290.68 (142.56, 537.13)	0.66 (0.21, 1.30)	173.53 (98.00, 286.54)	282.45 (156.49, 469.27)	0.63 (0.15, 1.33)	43.74 (20.60, 80.81)	79.69 (43.18, 131.14)	0.82 (0.18, 1.84)
**Hungary**	0.791	540.78 (334.52, 863.59)	357.23 (211.59, 583.66)	-0.34 (-0.50, -0.12)	358.84 (180.30, 669.09)	258.35 (130.33, 488.22)	-0.28 (-0.46, -0.03)	161.62 (103.76, 239.75)	76.65 (45.09, 115.80)	-0.53 (-0.65, -0.36)	20.32 (12.52, 29.96)	22.23 (13.54, 33.26)	0.09 (-0.18, 0.50)
**Iceland**	0.869	108.00 (69.98, 170.80)	84.46 (49.77, 145.95)	-0.22 (-0.40, 0.01)	66.13 (34.83, 122.66)	52.16 (24.33, 111.04)	-0.21 (-0.41, 0.05)	31.14 (19.91, 45.76)	21.54 (12.22, 34.71)	-0.31 (-0.49, -0.07)	10.72 (6.84, 16.15)	10.76 (6.65, 16.18)	0.00 (-0.24, 0.35)
**India**	0.566	352.96 (237.50, 514.43)	411.59 (275.44, 591.59)	0.17 (-0.04, 0.44)	121.36 (61.90, 234.01)	157.19 (81.42, 293.15)	0.30 (0.04, 0.64)	200.90 (123.26, 296.16)	223.26 (128.63, 338.97)	0.11 (-0.11, 0.39)	30.70 (10.75, 58.51)	31.15 (15.71, 52.27)	0.01 (-0.28, 0.70)
**Indonesia**	0.660	438.12 (293.80, 643.77)	752.86 (469.93, 1166.16)	0.72 (0.33, 1.14)	158.79 (78.42, 300.47)	349.82 (160.51, 710.96)	1.20 (0.72, 1.70)	257.38 (164.28, 380.38)	376.72 (216.49, 575.77)	0.46 (0.13, 0.84)	21.96 (12.11, 37.44)	26.32 (12.64, 45.06)	0.20 (-0.16, 0.78)
**Iran (Islamic Republic of)**	0.670	333.20 (209.55, 554.12)	352.20 (207.74, 596.84)	0.06 (-0.13, 0.29)	257.05 (142.88, 464.15)	287.01 (145.27, 533.19)	0.12 (-0.09, 0.36)	59.24 (37.05, 83.87)	52.50 (32.92, 77.66)	-0.11 (-0.29, 0.12)	16.91 (8.99, 27.72)	12.69 (7.83, 18.27)	-0.25 (-0.49, 0.22)
**Iraq**	0.671	707.29 (469.32, 1088.10)	884.45 (563.87, 1343.07)	0.25 (-0.12, 0.74)	416.09 (233.00, 732.43)	585.61 (327.82, 1009.38)	0.41 (0.00, 0.96)	259.20 (159.00, 389.80)	281.93 (168.60, 408.57)	0.09 (-0.26, 0.59)	31.99 (18.28, 51.14)	16.91 (9.76, 27.04)	-0.47 (-0.67, -0.06)
**Ireland**	0.867	124.22 (77.58, 201.76)	108.80 (64.03, 192.29)	-0.12 (-0.35, 0.17)	78.43 (40.13, 146.54)	67.89 (31.06, 151.61)	-0.13 (-0.39, 0.21)	29.80 (19.08, 43.53)	25.60 (14.79, 41.41)	-0.14 (-0.37, 0.17)	15.99 (9.77, 24.52)	15.31 (9.56, 22.46)	-0.04 (-0.30, 0.35)

**Israel**	0.803	157.28 (101.18, 252.54)	119.41 (70.47, 199.06)	-0.24 (-0.41, -0.04)	82.98 (40.79, 166.10)	64.33 (28.48, 136.78)	-0.22 (-0.42, 0.01)	65.31 (40.58, 99.91)	44.62 (24.84, 69.32)	-0.32 (-0.48, -0.11)	9.00 (5.59, 13.58)	10.46 (6.43, 15.92)	0.16 (-0.14, 0.59)
**Italy**	0.801	275.77 (159.47, 468.98)	146.35 (89.25, 255.64)	-0.47 (-0.55, -0.37)	188.04 (86.61, 385.70)	82.77 (37.92, 183.21)	-0.56 (-0.63, -0.46)	73.38 (44.19, 107.17)	49.91 (29.94, 75.13)	-0.32 (-0.41, -0.19)	14.36 (9.11, 20.69)	13.67 (8.50, 19.97)	-0.05 (-0.17, 0.11)
**Jamaica**	0.684	513.22 (320.13, 833.29)	568.88 (354.82, 862.97)	0.11 (-0.16, 0.48)	248.96 (112.92, 535.09)	240.38 (111.59, 465.00)	-0.03 (-0.25, 0.31)	232.32 (135.50, 370.11)	283.76 (153.13, 441.51)	0.22 (-0.14, 0.69)	31.94 (18.23, 50.72)	44.75 (25.98, 70.46)	0.40 (-0.04, 1.03)
**Japan**	0.870	202.45 (129.21, 325.20)	104.10 (68.42, 160.58)	-0.49 (-0.55, -0.41)	117.84 (59.52, 229.70)	51.60 (25.73, 99.90)	-0.56 (-0.62, -0.50)	59.02 (38.70, 84.87)	32.02 (20.69, 47.14)	-0.46 (-0.51, -0.39)	25.59 (16.23, 37.20)	20.49 (12.94, 30.02)	-0.20 (-0.29, -0.08)
**Jordan**	0.731	726.28 (442.41, 1178.80)	432.51 (257.92, 673.15)	-0.40 (-0.56, -0.21)	528.43 (276.28, 973.16)	331.46 (170.55, 568.24)	-0.37 (-0.53, -0.17)	182.98 (108.35, 271.73)	91.89 (54.66, 139.43)	-0.50 (-0.64, -0.31)	14.86 (8.49, 23.69)	9.16 (5.53, 13.74)	-0.38 (-0.59, -0.08)
**Kazakhstan**	0.723	481.17 (295.65, 799.22)	920.72 (577.84, 1453.64)	0.91 (0.41, 1.57)	286.52 (141.32, 561.80)	535.12 (254.70, 1036.54)	0.87 (0.34, 1.63)	170.58 (101.36, 274.70)	334.83 (193.98, 505.26)	0.96 (0.38, 1.82)	24.07 (13.90, 39.04)	50.76 (29.03, 78.75)	1.11 (0.42, 2.15)
**Kenya**	0.508	237.80 (142.90, 358.53)	309.49 (190.22, 467.08)	0.30 (0.10, 0.56)	80.46 (37.07, 173.41)	109.69 (52.33, 227.78)	0.36 (0.14, 0.63)	150.14 (82.25, 234.20)	189.96 (102.51, 296.11)	0.27 (0.05, 0.54)	7.20 (2.93, 15.41)	9.84 (4.05, 21.39)	0.37 (0.12, 0.80)
**Kiribati**	0.527	2071.09 (1339.80, 2975.00)	2063.84 (1384.68, 2926.20)	0.00 (-0.30, 0.37)	429.54 (244.06, 708.62)	492.61 (276.20, 804.71)	0.15 (-0.20, 0.63)	1503.23 (925.00, 2271.63)	1404.19 (863.55, 2100.96)	-0.07 (-0.35, 0.31)	138.32 (77.86, 214.21)	167.04 (94.19, 263.61)	0.21 (-0.19, 0.75)
**Kuwait**	0.851	299.16 (184.06, 488.76)	281.08 (178.13, 446.73)	-0.06 (-0.26, 0.22)	223.73 (114.51, 411.11)	200.83 (110.84, 352.13)	-0.10 (-0.29, 0.18)	62.22 (38.10, 95.15)	68.31 (42.48, 102.39)	0.10 (-0.19, 0.49)	13.21 (8.25, 20.11)	11.94 (7.42, 17.20)	-0.10 (-0.33, 0.25)

**Kyrgyzstan**	0.596	326.76 (211.82, 489.19)	290.35 (186.93, 454.45)	-0.11 (-0.28, 0.13)	174.26 (95.99, 310.94)	158.27 (81.93, 292.18)	-0.09 (-0.28, 0.19)	134.87 (84.85, 198.53)	111.60 (67.97, 161.96)	-0.17 (-0.34, 0.08)	17.63 (9.97, 28.13)	20.47 (12.07, 30.55)	0.16 (-0.12, 0.58)
**Lao People's Democratic Republic**	0.490	662.26 (419.91, 1020.13)	995.82 (636.01, 1439.49)	0.50 (0.09, 1.07)	223.51 (112.97, 461.52)	394.27 (186.42, 720.86)	0.76 (0.24, 1.52)	405.26 (238.81, 618.79)	563.55 (310.68, 863.60)	0.39 (-0.03, 0.95)	33.49 (14.84, 63.35)	38.00 (19.55, 69.76)	0.13 (-0.25, 0.95)
**Latvia**	0.820	391.80 (259.81, 605.45)	282.66 (176.37, 446.10)	-0.28 (-0.41, -0.10)	266.90 (146.41, 472.54)	210.88 (118.40, 370.59)	-0.21 (-0.35, -0.02)	104.47 (68.57, 146.33)	54.12 (34.44, 78.00)	-0.48 (-0.59, -0.33)	20.42 (12.15, 31.28)	17.65 (10.51, 26.46)	-0.14 (-0.34, 0.24)
**Lebanon**	0.708	218.17 (127.45, 386.42)	234.26 (122.75, 400.57)	0.07 (-0.26, 0.52)	148.01 (69.26, 318.73)	194.00 (91.25, 359.70)	0.31 (-0.09, 0.87)	59.84 (34.33, 97.96)	34.31 (19.07, 56.99)	-0.43 (-0.62, -0.15)	10.32 (5.53, 17.85)	5.95 (3.33, 9.75)	-0.42 (-0.68, 0.06)
**Lesotho**	0.507	485.39 (281.15, 834.34)	933.81 (556.92, 1471.77)	0.92 (0.29, 1.96)	188.12 (69.63, 428.98)	339.66 (141.20, 743.61)	0.81 (0.27, 1.79)	284.15 (147.07, 498.72)	572.83 (297.81, 945.58)	1.02 (0.29, 2.14)	13.12 (5.16, 30.05)	21.32 (10.20, 40.05)	0.62 (-0.06, 1.82)
**Liberia**	0.370	367.32 (221.05, 590.68)	463.34 (277.77, 700.97)	0.26 (-0.11, 0.82)	131.49 (61.11, 281.85)	165.60 (74.53, 329.48)	0.26 (-0.10, 0.80)	227.90 (128.00, 364.43)	288.02 (151.67, 470.36)	0.26 (-0.13, 0.85)	7.93 (2.83, 18.93)	9.73 (3.36, 25.79)	0.23 (-0.24, 0.95)
**Libya**	0.709	413.16 (241.06, 701.69)	502.49 (284.86, 866.15)	0.22 (-0.10, 0.70)	256.90 (115.44, 535.47)	382.74 (195.84, 732.45)	0.49 (0.09, 1.14)	135.19 (73.28, 214.55)	104.00 (57.93, 163.49)	-0.23 (-0.47, 0.16)	21.07 (10.55, 37.83)	15.76 (8.64, 26.31)	-0.25 (-0.54, 0.30)
**Lithuania**	0.843	186.21 (120.33, 293.97)	170.90 (107.71, 276.52)	-0.08 (-0.24, 0.12)	131.28 (73.95, 236.18)	123.18 (69.86, 225.76)	-0.06 (-0.23, 0.15)	40.48 (25.19, 59.56)	33.21 (20.53, 48.96)	-0.18 (-0.35, 0.02)	14.45 (8.27, 22.17)	14.51 (8.61, 22.28)	0.00 (-0.20, 0.28)
**Luxembourg**	0.895	177.56 (108.82, 289.64)	151.44 (93.02, 252.01)	-0.15 (-0.34, 0.11)	114.56 (59.16, 217.04)	87.23 (40.04, 180.74)	-0.24 (-0.42, -0.01)	54.22 (34.00, 77.96)	50.09 (28.91, 77.59)	-0.08 (-0.32, 0.25)	8.79 (5.51, 13.13)	14.12 (8.81, 21.28)	0.61 (0.15, 1.23)

**Madagascar**	0.396	373.00 (241.87, 564.10)	476.14 (289.70, 759.31)	0.28 (-0.09, 0.80)	104.18 (50.67, 221.76)	159.75 (74.74, 329.60)	0.53 (0.07, 1.22)	254.60 (154.87, 383.57)	299.65 (167.16, 486.07)	0.18 (-0.17, 0.68)	14.22 (5.13, 31.00)	16.74 (5.86, 39.80)	0.18 (-0.22, 0.75)
**Malawi**	0.384	391.78 (227.19, 657.51)	482.89 (288.71, 738.63)	0.23 (-0.12, 0.79)	154.15 (64.61, 353.31)	203.77 (91.17, 417.39)	0.32 (-0.05, 0.95)	227.21 (120.72, 395.22)	264.97 (137.78, 430.71)	0.17 (-0.20, 0.76)	10.42 (3.43, 26.92)	14.14 (4.30, 37.46)	0.36 (-0.14, 1.09)
**Malaysia**	0.737	613.20 (431.05, 850.24)	490.67 (320.90, 754.46)	-0.20 (-0.41, 0.09)	212.14 (114.95, 372.44)	226.37 (115.60, 426.20)	0.07 (-0.22, 0.46)	364.90 (239.74, 516.86)	241.61 (144.16, 369.91)	-0.34 (-0.51, -0.08)	36.16 (21.83, 54.26)	22.69 (13.71, 35.16)	-0.37 (-0.58, -0.01)
**Maldives**	0.562	448.27 (288.23, 676.75)	297.65 (181.87, 476.72)	-0.34 (-0.51, -0.10)	158.69 (78.88, 318.62)	148.95 (68.32, 309.10)	-0.06 (-0.34, 0.30)	260.14 (164.56, 390.61)	131.63 (78.87, 208.88)	-0.49 (-0.63, -0.30)	29.43 (12.73, 51.75)	17.07 (9.89, 27.18)	-0.42 (-0.66, 0.26)
**Mali**	0.263	261.11 (169.02, 389.31)	396.65 (234.12, 615.74)	0.52 (0.08, 1.13)	79.28 (41.09, 151.38)	130.29 (62.20, 276.73)	0.64 (0.13, 1.29)	175.46 (107.63, 265.54)	258.51 (139.98, 425.73)	0.47 (0.04, 1.17)	6.37 (2.48, 13.48)	7.85 (3.08, 17.80)	0.23 (-0.16, 0.86)
**Malta**	0.801	355.00 (204.81, 588.96)	160.61 (93.32, 267.18)	-0.55 (-0.64, -0.42)	221.98 (95.76, 446.20)	99.41 (45.07, 205.93)	-0.55 (-0.65, -0.42)	116.92 (65.08, 184.97)	48.90 (28.30, 73.47)	-0.58 (-0.67, -0.46)	16.09 (9.72, 24.42)	12.31 (7.63, 18.07)	-0.24 (-0.39, -0.02)
**Marshall Islands**	0.544	1759.61 (1126.05, 2515.70)	1637.75 (1023.21, 2474.44)	-0.07 (-0.29, 0.25)	389.34 (209.72, 714.12)	371.81 (216.64, 642.85)	-0.05 (-0.28, 0.28)	1200.50 (722.73, 1793.42)	1108.25 (637.31, 1778.98)	-0.08 (-0.34, 0.27)	169.76 (77.11, 309.60)	157.70 (73.65, 279.04)	-0.07 (-0.37, 0.41)
**Mauritania**	0.496	242.37 (163.64, 359.93)	206.81 (125.34, 333.03)	-0.15 (-0.39, 0.21)	89.65 (49.02, 168.68)	86.86 (42.35, 190.02)	-0.03 (-0.32, 0.36)	147.47 (92.36, 220.83)	116.06 (63.92, 186.79)	-0.21 (-0.45, 0.15)	5.25 (2.38, 9.67)	3.89 (1.84, 7.12)	-0.26 (-0.53, 0.15)
**Mauritius**	0.705	814.74 (524.76, 1234.62)	421.75 (285.30, 607.80)	-0.48 (-0.62, -0.30)	444.56 (221.47, 836.51)	204.06 (116.42, 348.14)	-0.54 (-0.66, -0.36)	334.63 (211.29, 490.34)	178.05 (109.90, 258.01)	-0.47 (-0.62, -0.28)	35.55 (22.00, 52.55)	39.64 (23.81, 58.97)	0.11 (-0.18, 0.53)

**Mexico**	0.649	336.21 (229.19, 499.94)	227.29 (156.73, 327.65)	-0.32 (-0.41, -0.20)	185.97 (98.30, 335.49)	110.88 (58.38, 201.83)	-0.40 (-0.48, -0.32)	126.44 (80.95, 175.32)	85.47 (52.72, 121.48)	-0.32 (-0.43, -0.19)	23.80 (15.30, 33.75)	30.95 (19.22, 44.78)	0.30 (-0.07, 0.61)
**Micronesia (Federated States of)**	0.580	965.48 (611.51, 1474.90)	1507.72 (932.55, 2245.66)	0.56 (0.05, 1.28)	223.98 (116.89, 430.99)	366.72 (190.75, 652.91)	0.64 (0.10, 1.45)	651.27 (393.33, 1003.61)	1025.65 (591.84, 1615.34)	0.57 (0.03, 1.31)	90.24 (38.72, 166.60)	115.34 (54.88, 201.48)	0.28 (-0.17, 1.06)
**Monaco**	0.902	141.65 (84.91, 234.24)	117.74 (67.62, 210.85)	-0.17 (-0.41, 0.25)	93.06 (48.11, 176.61)	79.99 (37.94, 166.47)	-0.14 (-0.40, 0.28)	35.82 (21.75, 53.77)	28.17 (16.29, 45.12)	-0.21 (-0.48, 0.24)	12.76 (7.21, 20.41)	9.57 (5.62, 14.72)	-0.25 (-0.50, 0.16)
**Mongolia**	0.606	264.18 (158.61, 423.11)	386.51 (240.49, 586.78)	0.46 (0.07, 0.99)	26.40 (14.19, 54.52)	48.85 (26.51, 90.10)	0.85 (0.33, 1.50)	220.26 (129.36, 359.57)	311.52 (185.92, 484.86)	0.41 (0.02, 0.97)	17.52 (9.03, 31.06)	26.15 (15.11, 42.33)	0.49 (-0.06, 1.46)
**Montenegro**	0.791	622.92 (398.60, 947.10)	1007.48 (640.60, 1507.74)	0.62 (0.19, 1.17)	112.30 (56.99, 224.86)	234.24 (113.05, 451.29)	1.09 (0.55, 1.89)	498.54 (293.71, 791.34)	754.07 (432.11, 1223.16)	0.51 (0.13, 1.04)	12.07 (7.34, 18.35)	19.17 (11.43, 28.62)	0.59 (0.16, 1.20)
**Morocco**	0.548	364.28 (233.16, 605.88)	720.21 (429.30, 1202.06)	0.98 (0.40, 1.73)	199.85 (101.57, 392.57)	535.04 (269.99, 989.58)	1.68 (0.90, 2.65)	138.66 (78.89, 217.19)	159.46 (90.94, 244.04)	0.15 (-0.22, 0.69)	25.76 (10.52, 47.26)	25.70 (13.56, 41.84)	0.00 (-0.38, 0.88)
**Mozambique**	0.307	369.39 (229.84, 570.77)	694.71 (420.03, 1095.06)	0.88 (0.31, 1.74)	131.27 (59.33, 287.90)	278.20 (124.77, 558.83)	1.12 (0.40, 2.27)	224.91 (129.31, 358.00)	398.89 (205.62, 659.25)	0.77 (0.22, 1.66)	13.22 (3.61, 35.36)	17.62 (6.19, 44.98)	0.33 (-0.14, 1.53)
**Myanmar**	0.521	877.46 (558.79, 1327.84)	1075.73 (706.67, 1596.36)	0.23 (-0.09, 0.71)	275.60 (137.73, 559.93)	445.65 (203.50, 857.57)	0.62 (0.14, 1.29)	554.75 (327.19, 827.87)	591.09 (349.49, 917.14)	0.07 (-0.25, 0.51)	47.11 (21.91, 86.14)	38.99 (22.15, 62.44)	-0.17 (-0.48, 0.42)
**Namibia**	0.612	597.72 (364.40, 945.02)	607.00 (356.74, 967.17)	0.02 (-0.25, 0.40)	269.55 (126.09, 570.88)	305.14 (136.46, 617.75)	0.13 (-0.19, 0.60)	314.91 (178.03, 509.28)	290.45 (152.63, 455.63)	-0.08 (-0.37, 0.34)	13.26 (6.97, 23.62)	11.41 (6.11, 20.51)	-0.14 (-0.46, 0.40)

**Nauru**	0.618	1142.24 (745.93, 1699.45)	1675.97 (1084.36, 2435.40)	0.47 (0.14, 0.90)	350.83 (182.35, 639.64)	526.79 (280.67, 887.55)	0.50 (0.15, 1.05)	724.39 (445.94, 1174.42)	1056.57 (622.62, 1649.08)	0.46 (0.09, 0.93)	67.03 (36.77, 106.82)	92.61 (48.52, 150.25)	0.38 (-0.04, 0.99)
**Nepal**	0.422	256.92 (163.66, 383.17)	321.89 (199.68, 507.96)	0.25 (-0.14, 0.78)	79.93 (37.53, 161.93)	127.55 (58.98, 255.88)	0.60 (0.06, 1.29)	147.78 (82.71, 229.20)	165.36 (94.36, 273.42)	0.12 (-0.24, 0.65)	29.21 (9.42, 61.78)	28.98 (11.40, 57.99)	-0.01 (-0.36, 0.69)
**Netherlands**	0.883	149.15 (85.74, 275.15)	103.53 (59.84, 183.63)	-0.31 (-0.48, -0.03)	98.48 (45.33, 220.72)	65.72 (29.99, 143.60)	-0.33 (-0.51, -0.06)	37.67 (22.12, 60.39)	27.41 (15.82, 45.05)	-0.27 (-0.46, 0.00)	13.00 (7.93, 19.57)	10.40 (6.39, 15.42)	-0.20 (-0.39, 0.10)
**New Zealand**	0.840	114.23 (70.41, 187.13)	87.69 (51.43, 166.96)	-0.23 (-0.40, 0.01)	71.02 (35.63, 136.50)	53.77 (24.64, 133.85)	-0.24 (-0.43, 0.04)	27.88 (17.60, 40.43)	20.94 (12.36, 32.97)	-0.25 (-0.41, 0.00)	15.33 (9.19, 22.98)	12.98 (7.83, 19.12)	-0.15 (-0.33, 0.09)
**Nicaragua**	0.517	362.89 (230.18, 541.39)	316.31 (198.67, 484.01)	-0.13 (-0.32, 0.14)	164.71 (75.90, 316.29)	157.11 (75.29, 309.53)	-0.05 (-0.26, 0.27)	173.44 (98.06, 259.59)	133.04 (76.51, 200.81)	-0.23 (-0.43, 0.05)	24.74 (14.41, 36.78)	26.16 (15.40, 40.17)	0.06 (-0.28, 0.53)
**Niger**	0.162	128.98 (82.84, 199.10)	388.03 (204.70, 636.25)	2.01 (0.98, 3.34)	41.51 (19.87, 87.89)	128.44 (52.74, 295.97)	2.09 (1.04, 3.41)	84.53 (50.79, 126.61)	252.10 (117.81, 438.11)	1.98 (0.85, 3.38)	2.94 (1.03, 6.60)	7.49 (2.39, 19.15)	1.55 (0.48, 3.11)
**Nigeria**	0.515	280.30 (164.71, 461.36)	277.61 (162.95, 453.61)	-0.01 (-0.31, 0.38)	99.11 (45.15, 234.93)	115.02 (51.16, 251.35)	0.16 (-0.20, 0.62)	175.85 (93.72, 285.84)	158.27 (82.94, 263.63)	-0.10 (-0.38, 0.29)	5.33 (2.14, 12.10)	4.31 (1.84, 8.74)	-0.19 (-0.46, 0.21)
**Niue**	0.711	1100.06 (714.52, 1558.18)	919.70 (612.72, 1311.19)	-0.16 (-0.38, 0.14)	341.48 (174.46, 625.26)	298.03 (168.53, 512.18)	-0.13 (-0.34, 0.18)	680.60 (402.39, 999.70)	559.57 (341.98, 847.19)	-0.18 (-0.42, 0.16)	77.98 (45.02, 120.86)	62.11 (32.24, 106.76)	-0.20 (-0.51, 0.24)
**North Macedonia**	0.744	954.43 (583.99, 1555.15)	1391.12 (821.40, 2353.56)	0.46 (0.02, 0.98)	623.78 (305.00, 1185.86)	957.68 (454.25, 1869.98)	0.54 (0.08, 1.13)	287.55 (170.03, 427.45)	358.12 (203.38, 566.44)	0.25 (-0.13, 0.73)	43.10 (25.14, 64.72)	75.33 (42.74, 114.97)	0.75 (0.14, 1.60)

**Northern Mariana Islands**	0.771	749.13 (502.09, 1093.20)	717.71 (494.27, 1023.57)	-0.04 (-0.26, 0.26)	233.63 (124.65, 459.55)	222.40 (124.01, 390.03)	-0.05 (-0.27, 0.29)	459.61 (290.87, 690.69)	440.75 (269.95, 640.13)	-0.04 (-0.29, 0.28)	55.89 (33.43, 88.07)	54.56 (32.76, 83.83)	-0.02 (-0.32, 0.37)
**Norway**	0.913	227.00 (134.55, 395.63)	134.19 (79.62, 231.14)	-0.41 (-0.48, -0.33)	157.23 (75.91, 320.28)	85.82 (39.92, 184.43)	-0.45 (-0.53, -0.37)	46.44 (28.46, 70.17)	30.62 (17.73, 47.66)	-0.34 (-0.44, -0.21)	23.33 (14.82, 33.64)	17.75 (11.09, 25.87)	-0.24 (-0.39, -0.05)
**Oman**	0.783	519.10 (324.89, 832.05)	600.57 (370.26, 978.15)	0.16 (-0.21, 0.73)	289.22 (151.78, 536.46)	433.23 (214.32, 799.70)	0.50 (0.05, 1.21)	203.72 (119.96, 322.68)	148.16 (87.55, 220.95)	-0.27 (-0.49, 0.10)	26.16 (13.72, 44.49)	19.18 (8.99, 33.41)	-0.27 (-0.59, 0.38)
**Pakistan**	0.449	367.46 (239.00, 537.12)	587.99 (385.82, 878.26)	0.60 (0.22, 1.18)	122.96 (64.20, 239.68)	238.61 (118.33, 448.05)	0.94 (0.42, 1.73)	207.50 (122.63, 299.35)	304.17 (186.82, 455.50)	0.47 (0.09, 1.05)	37.01 (14.24, 67.88)	45.21 (22.54, 76.50)	0.22 (-0.14, 0.91)
**Palau**	0.738	876.01 (563.63, 1298.97)	1024.64 (676.78, 1456.76)	0.17 (-0.18, 0.65)	325.23 (171.94, 610.68)	396.89 (225.38, 660.85)	0.22 (-0.13, 0.75)	503.41 (298.65, 783.95)	570.69 (332.89, 870.19)	0.13 (-0.24, 0.67)	47.37 (26.54, 76.18)	57.06 (33.14, 90.74)	0.20 (-0.25, 0.91)
**Palestine**	0.588	762.17 (429.45, 1237.99)	733.55 (439.80, 1237.84)	-0.04 (-0.31, 0.36)	491.27 (225.30, 955.10)	559.14 (277.77, 1051.09)	0.14 (-0.15, 0.60)	253.57 (136.71, 411.62)	160.67 (93.50, 243.23)	-0.37 (-0.56, -0.07)	17.33 (9.07, 29.22)	13.74 (8.31, 20.75)	-0.21 (-0.49, 0.30)
**Panama**	0.686	342.63 (210.10, 542.21)	248.77 (154.61, 384.59)	-0.27 (-0.45, 0.01)	161.39 (72.64, 324.19)	112.51 (53.24, 224.87)	-0.30 (-0.48, -0.06)	154.50 (90.58, 247.08)	104.18 (58.03, 163.33)	-0.33 (-0.53, -0.01)	26.74 (15.90, 42.72)	32.08 (18.86, 49.13)	0.20 (-0.18, 0.80)
**Papua New Guinea**	0.394	676.62 (401.68, 1084.12)	1109.45 (678.57, 1655.29)	0.64 (0.20, 1.30)	126.10 (60.78, 249.33)	216.79 (109.78, 404.51)	0.72 (0.28, 1.38)	491.87 (277.08, 823.92)	802.49 (446.79, 1247.21)	0.63 (0.16, 1.34)	58.65 (22.80, 120.15)	90.16 (39.89, 171.89)	0.54 (0.08, 1.38)
**Paraguay**	0.638	276.85 (174.78, 439.66)	323.76 (190.71, 529.00)	0.17 (-0.19, 0.72)	122.07 (55.11, 259.60)	165.36 (74.28, 334.43)	0.35 (-0.08, 1.05)	131.84 (80.01, 196.93)	127.63 (66.57, 204.84)	-0.03 (-0.35, 0.48)	22.94 (13.51, 35.13)	30.77 (17.05, 52.11)	0.34 (-0.15, 1.18)

**Peru**	0.648	127.13 (83.67, 186.85)	86.22 (49.92, 147.35)	-0.32 (-0.54, 0.02)	49.06 (25.53, 93.83)	41.61 (19.26, 86.58)	-0.15 (-0.43, 0.29)	62.77 (39.42, 93.55)	33.04 (18.09, 55.72)	-0.47 (-0.65, -0.21)	15.31 (8.57, 24.73)	11.57 (6.32, 19.99)	-0.24 (-0.52, 0.21)
**Philippines**	0.623	267.69 (171.82, 422.34)	454.50 (300.35, 669.22)	0.70 (0.27, 1.18)	124.99 (62.34, 253.76)	183.41 (94.79, 332.15)	0.47 (0.08, 0.91)	133.48 (80.01, 202.79)	252.64 (145.34, 382.39)	0.89 (0.32, 1.45)	9.22 (5.30, 14.92)	18.46 (11.07, 27.44)	1.00 (0.45, 1.73)
**Poland**	0.802	412.93 (277.36, 620.54)	315.19 (194.24, 494.78)	-0.24 (-0.38, -0.07)	255.67 (141.65, 448.25)	208.66 (107.10, 378.17)	-0.18 (-0.34, -0.01)	126.55 (84.31, 177.04)	81.31 (50.32, 120.88)	-0.36 (-0.49, -0.20)	30.71 (19.61, 44.70)	25.22 (15.79, 36.67)	-0.18 (-0.35, 0.06)
**Portugal**	0.743	594.40 (358.85, 1000.73)	254.39 (160.36, 423.30)	-0.57 (-0.68, -0.44)	383.42 (180.76, 790.73)	148.46 (70.18, 303.73)	-0.61 (-0.71, -0.47)	190.64 (118.56, 291.26)	86.03 (53.28, 128.02)	-0.55 (-0.66, -0.42)	20.33 (13.08, 29.80)	19.90 (12.52, 29.08)	-0.02 (-0.22, 0.27)
**Puerto Rico**	0.814	221.35 (145.07, 333.79)	135.98 (89.74, 200.77)	-0.39 (-0.54, -0.17)	112.27 (55.90, 214.77)	61.61 (34.52, 107.49)	-0.45 (-0.58, -0.26)	88.84 (58.20, 126.81)	56.72 (33.43, 84.49)	-0.36 (-0.55, -0.09)	20.24 (12.70, 29.45)	17.65 (10.49, 26.23)	-0.13 (-0.38, 0.27)
**Qatar**	0.830	496.90 (314.37, 804.93)	269.33 (169.10, 439.41)	-0.46 (-0.61, -0.26)	294.99 (145.78, 572.89)	174.18 (95.91, 335.88)	-0.41 (-0.57, -0.18)	157.23 (92.08, 240.81)	71.34 (44.13, 114.00)	-0.55 (-0.69, -0.33)	44.69 (22.95, 79.65)	23.81 (14.08, 36.93)	-0.47 (-0.70, -0.06)
**Republic of Korea**	0.878	569.94 (390.01, 811.29)	186.20 (108.72, 319.88)	-0.67 (-0.76, -0.50)	257.69 (137.01, 453.71)	114.01 (52.94, 244.13)	-0.56 (-0.68, -0.31)	266.38 (168.19, 372.59)	53.15 (31.87, 82.11)	-0.80 (-0.85, -0.70)	45.87 (27.36, 68.15)	19.05 (11.71, 28.95)	-0.58 (-0.69, -0.40)
**Republic of Moldova**	0.696	395.31 (268.46, 574.94)	321.74 (207.17, 488.82)	-0.19 (-0.36, 0.03)	171.07 (89.99, 315.21)	188.55 (100.46, 331.20)	0.10 (-0.15, 0.46)	211.18 (136.79, 305.00)	120.53 (73.60, 178.23)	-0.43 (-0.55, -0.28)	13.06 (7.92, 19.82)	12.66 (7.71, 18.58)	-0.03 (-0.25, 0.32)
**Romania**	0.760	443.93 (288.72, 672.57)	370.37 (236.00, 584.53)	-0.17 (-0.33, 0.05)	266.93 (144.65, 477.17)	226.80 (117.41, 415.17)	-0.15 (-0.34, 0.10)	150.92 (99.25, 222.36)	112.97 (71.14, 168.98)	-0.25 (-0.43, -0.01)	26.08 (16.06, 40.76)	30.60 (19.40, 45.30)	0.17 (-0.17, 0.66)

Russian Federation	0.805	576.76 (380.85, 875.62)	465.41 (301.74, 708.23)	-0.19 (-0.34, -0.02)	409.52 (237.73, 696.86)	313.05 (177.99, 524.54)	-0.24 (-0.38, -0.06)	136.93 (91.67, 191.99)	115.02 (73.24, 165.52)	-0.16 (-0.34, 0.07)	30.30 (18.23, 44.02)	37.34 (22.98, 55.02)	0.23 (0.01, 0.53)
Rwanda	0.429	522.29 (271.09, 869.07)	425.64 (236.14, 674.61)	-0.19 (-0.45, 0.27)	140.46 (52.14, 329.73)	143.60 (57.10, 335.10)	0.02 (-0.31, 0.54)	366.24 (176.22, 655.78)	268.48 (123.67, 459.16)	-0.27 (-0.53, 0.17)	15.59 (4.47, 39.79)	13.55 (4.26, 33.19)	-0.13 (-0.49, 0.70)
Saint Kitts and Nevis	0.746	1386.66 (903.59, 2071.67)	766.42 (496.97, 1139.57)	-0.45 (-0.55, -0.31)	668.85 (319.35, 1335.58)	380.34 (189.96, 708.97)	-0.43 (-0.54, -0.29)	666.61 (397.61, 1006.71)	346.23 (205.19, 514.96)	-0.48 (-0.61, -0.30)	51.20 (30.89, 77.64)	39.86 (23.45, 62.88)	-0.22 (-0.46, 0.13)
Saint Lucia	0.670	975.28 (638.34, 1428.19)	528.47 (354.13, 784.72)	-0.46 (-0.56, -0.33)	476.23 (238.52, 890.78)	247.63 (130.80, 453.18)	-0.48 (-0.58, -0.34)	446.50 (264.56, 674.59)	243.11 (144.62, 366.60)	-0.46 (-0.58, -0.30)	52.55 (31.58, 80.28)	37.73 (22.55, 55.75)	-0.28 (-0.48, -0.02)
Saint Vincent and the Grenadines	0.627	658.37 (400.56, 1039.00)	561.04 (377.73, 804.00)	-0.15 (-0.33, 0.09)	427.15 (214.29, 804.67)	257.39 (138.11, 471.67)	-0.40 (-0.50, -0.27)	192.78 (115.35, 288.13)	273.33 (163.29, 396.33)	0.42 (0.12, 0.80)	38.43 (22.86, 59.43)	30.32 (18.34, 45.34)	-0.21 (-0.43, 0.06)
Samoa	0.641	1038.92 (642.59, 1485.16)	1101.41 (728.71, 1580.08)	0.06 (-0.18, 0.42)	290.59 (140.66, 565.28)	317.50 (164.27, 581.80)	0.09 (-0.14, 0.46)	672.97 (382.89, 1028.00)	703.05 (419.61, 1054.27)	0.04 (-0.22, 0.47)	75.36 (41.54, 134.19)	80.85 (45.39, 133.98)	0.07 (-0.30, 0.65)
San Marino	0.884	106.73 (65.36, 182.80)	135.98 (72.04, 248.09)	0.27 (-0.16, 0.88)	70.08 (35.88, 138.96)	91.35 (38.23, 202.01)	0.30 (-0.13, 0.92)	28.58 (17.45, 43.61)	34.32 (17.68, 62.65)	0.20 (-0.28, 0.86)	8.07 (4.94, 11.96)	10.31 (5.58, 16.68)	0.28 (-0.18, 0.96)
Sao Tome and Principe	0.502	335.42 (209.33, 516.23)	525.41 (327.70, 822.86)	0.57 (0.16, 1.11)	133.91 (64.36, 294.38)	223.85 (102.41, 432.41)	0.67 (0.22, 1.32)	194.76 (115.32, 300.63)	293.47 (160.33, 490.40)	0.51 (0.08, 1.10)	6.74 (2.75, 13.49)	8.10 (3.79, 15.78)	0.20 (-0.18, 0.81)
Saudi Arabia	0.805	668.93 (416.13, 1065.50)	631.39 (410.47, 929.63)	-0.06 (-0.35, 0.38)	380.80 (199.85, 718.65)	399.14 (223.95, 669.16)	0.05 (-0.31, 0.56)	270.76 (159.04, 406.15)	220.73 (135.31, 329.71)	-0.18 (-0.46, 0.24)	17.37 (9.29, 28.98)	11.52 (6.55, 17.79)	-0.34 (-0.64, 0.18)

**Senegal**	0.389	373.03 (244.40, 562.12)	476.01 (298.00, 726.99)	0.28 (-0.05, 0.75)	141.07 (71.21, 272.56)	178.36 (86.10, 335.16)	0.26 (-0.05, 0.68)	224.37 (137.47, 337.79)	288.73 (165.75, 457.22)	0.29 (-0.11, 0.83)	7.58 (3.06, 15.19)	8.92 (3.66, 18.13)	0.18 (-0.19, 0.71)
**Serbia**	0.767	814.70 (490.79, 1375.87)	853.23 (490.73, 1469.22)	0.05 (-0.27, 0.47)	536.59 (253.83, 1085.64)	636.75 (304.84, 1230.19)	0.19 (-0.18, 0.67)	230.45 (139.35, 342.90)	165.60 (96.95, 263.01)	-0.28 (-0.51, 0.01)	47.66 (28.12, 72.84)	50.88 (29.61, 78.62)	0.07 (-0.28, 0.57)
**Seychelles**	0.724	497.37 (332.74, 765.55)	480.31 (321.06, 702.86)	-0.03 (-0.24, 0.27)	234.86 (118.61, 470.10)	242.62 (130.60, 442.74)	0.03 (-0.21, 0.40)	242.10 (154.82, 351.52)	220.17 (138.25, 328.42)	-0.09 (-0.30, 0.20)	20.41 (10.34, 32.57)	17.52 (8.54, 30.03)	-0.14 (-0.38, 0.20)
**Sierra Leone**	0.347	179.88 (110.58, 279.84)	260.59 (158.65, 422.32)	0.45 (0.06, 1.01)	65.63 (33.57, 123.80)	96.35 (45.58, 201.57)	0.47 (0.05, 1.08)	110.56 (62.87, 171.30)	159.45 (86.90, 259.71)	0.44 (0.03, 1.04)	3.68 (1.37, 8.32)	4.80 (1.87, 10.55)	0.30 (-0.12, 1.00)
**Singapore**	0.861	390.49 (258.48, 568.30)	108.47 (70.49, 174.24)	-0.72 (-0.78, -0.65)	225.68 (123.17, 388.38)	59.31 (31.37, 114.85)	-0.74 (-0.80, -0.66)	139.09 (93.02, 192.64)	36.59 (23.09, 54.11)	-0.74 (-0.79, -0.66)	25.73 (16.64, 35.62)	12.57 (7.88, 18.09)	-0.51 (-0.60, -0.39)
**Slovakia**	0.812	344.75 (230.19, 516.39)	244.10 (148.27, 417.97)	-0.29 (-0.48, -0.05)	205.37 (115.83, 358.04)	172.17 (89.25, 338.69)	-0.16 (-0.38, 0.10)	116.69 (75.15, 163.23)	57.64 (33.95, 89.22)	-0.51 (-0.64, -0.31)	22.70 (14.30, 32.50)	14.29 (8.40, 22.01)	-0.37 (-0.53, -0.13)
**Slovenia**	0.840	357.23 (207.06, 638.93)	147.19 (86.03, 251.42)	-0.59 (-0.72, -0.41)	258.95 (128.17, 526.58)	95.48 (46.28, 193.47)	-0.63 (-0.75, -0.47)	81.18 (48.66, 123.43)	39.25 (21.96, 64.04)	-0.52 (-0.69, -0.27)	17.11 (10.07, 25.97)	12.46 (7.33, 18.95)	-0.27 (-0.49, 0.04)
**Solomon Islands**	0.407	1135.91 (749.83, 1630.75)	2307.05 (1506.12, 3312.49)	1.03 (0.45, 1.78)	198.04 (111.96, 336.45)	457.11 (242.11, 781.15)	1.31 (0.61, 2.17)	847.80 (524.72, 1233.76)	1696.77 (994.40, 2636.50)	1.00 (0.40, 1.78)	90.07 (41.31, 166.11)	153.17 (76.53, 283.91)	0.70 (0.14, 1.58)
**Somalia**	0.081	406.22 (247.20, 640.59)	490.66 (274.59, 783.41)	0.21 (-0.17, 0.86)	107.39 (49.49, 235.17)	155.76 (69.02, 333.88)	0.45 (-0.04, 1.23)	282.24 (157.26, 452.58)	311.91 (151.78, 533.83)	0.11 (-0.27, 0.70)	16.59 (4.18, 48.15)	22.99 (4.95, 76.98)	0.39 (-0.23, 1.24)

**South Africa**	0.678	315.15 (203.66, 495.56)	438.89 (280.84, 666.89)	0.39 (0.17, 0.70)	154.25 (76.59, 323.97)	249.15 (122.57, 477.80)	0.62 (0.33, 1.00)	153.33 (96.56, 218.90)	180.59 (107.45, 260.35)	0.18 (-0.09, 0.48)	7.57 (4.63, 11.20)	9.15 (5.67, 13.23)	0.21 (-0.04, 0.51)
**South Sudan**	0.363	301.30 (181.99, 496.91)	311.94 (175.01, 499.14)	0.04 (-0.27, 0.54)	97.74 (42.75, 215.76)	120.68 (52.01, 265.06)	0.23 (-0.14, 0.93)	195.03 (109.31, 310.73)	182.03 (93.02, 300.72)	-0.07 (-0.38, 0.40)	8.53 (3.29, 19.55)	9.23 (3.21, 22.14)	0.08 (-0.31, 0.72)
**Spain**	0.767	333.34 (190.63, 613.03)	135.54 (83.70, 221.86)	-0.59 (-0.67, -0.48)	222.36 (99.85, 486.37)	73.99 (34.30, 158.89)	-0.67 (-0.73, -0.57)	97.94 (58.14, 150.31)	47.50 (28.24, 72.61)	-0.52 (-0.61, -0.39)	13.04 (8.09, 19.24)	14.05 (8.73, 21.00)	0.08 (-0.14, 0.37)
**Sri Lanka**	0.690	515.08 (308.07, 902.42)	418.28 (258.83, 700.85)	-0.19 (-0.38, 0.08)	305.65 (132.43, 686.63)	257.86 (120.82, 507.77)	-0.16 (-0.36, 0.15)	156.24 (86.89, 248.51)	120.35 (66.57, 189.92)	-0.23 (-0.47, 0.11)	53.19 (26.39, 92.57)	40.07 (22.09, 66.17)	-0.25 (-0.56, 0.32)
**Sudan**	0.515	540.49 (313.98, 925.82)	789.95 (460.45, 1335.49)	0.46 (0.04, 1.10)	257.26 (119.77, 569.55)	554.33 (271.37, 1092.51)	1.15 (0.53, 2.18)	245.72 (134.61, 384.73)	206.16 (111.72, 332.58)	-0.16 (-0.42, 0.21)	37.51 (12.05, 79.09)	29.47 (13.35, 54.84)	-0.21 (-0.51, 0.56)
**Suriname**	0.636	629.80 (420.36, 929.14)	721.87 (491.57, 1040.58)	0.15 (-0.11, 0.53)	253.51 (125.53, 492.53)	271.12 (141.45, 498.22)	0.07 (-0.17, 0.44)	342.59 (209.51, 513.70)	407.56 (246.29, 609.35)	0.19 (-0.10, 0.64)	33.69 (20.64, 50.00)	43.18 (26.29, 65.85)	0.28 (-0.07, 0.81)
**Sweden**	0.872	139.61 (83.84, 239.53)	123.93 (71.64, 220.43)	-0.11 (-0.30, 0.14)	92.76 (45.73, 191.48)	81.02 (38.29, 176.19)	-0.13 (-0.33, 0.15)	34.03 (21.50, 50.81)	31.55 (18.33, 50.54)	-0.07 (-0.28, 0.19)	12.82 (8.27, 18.62)	11.36 (7.06, 17.10)	-0.11 (-0.32, 0.22)
**Switzerland**	0.929	146.30 (87.42, 266.81)	74.66 (44.43, 139.65)	-0.49 (-0.61, -0.33)	95.09 (46.88, 205.76)	47.81 (22.68, 111.10)	-0.50 (-0.62, -0.33)	38.08 (23.25, 59.51)	17.09 (9.99, 28.08)	-0.55 (-0.67, -0.40)	13.13 (8.34, 19.64)	9.76 (6.16, 14.58)	-0.26 (-0.41, -0.03)
**Syrian Arab Republic**	0.619	505.82 (316.92, 815.27)	589.86 (365.59, 968.30)	0.17 (-0.20, 0.70)	258.75 (135.10, 519.04)	347.95 (171.61, 688.58)	0.34 (-0.08, 0.94)	229.44 (138.44, 353.98)	226.81 (130.87, 362.17)	-0.01 (-0.34, 0.50)	17.63 (8.87, 30.42)	15.10 (8.73, 24.50)	-0.14 (-0.48, 0.55)

**Taiwan (Province of China)**	0.868	391.98 (271.41, 554.10)	190.15 (120.86, 296.96)	-0.51 (-0.63, -0.37)	155.21 (83.38, 276.81)	92.63 (45.16, 175.11)	-0.40 (-0.54, -0.22)	223.65 (146.84, 311.54)	82.02 (49.54, 126.17)	-0.63 (-0.72, -0.51)	13.12 (8.57, 18.72)	15.50 (9.36, 23.62)	0.18 (-0.11, 0.60)
**Tajikistan**	0.539	247.13 (163.34, 345.01)	970.48 (571.35, 1468.04)	2.93 (1.79, 4.38)	35.58 (19.15, 63.06)	196.45 (90.39, 412.22)	4.52 (2.72, 7.05)	205.44 (128.42, 294.19)	752.80 (388.93, 1229.86)	2.66 (1.53, 4.08)	6.11 (3.17, 9.95)	21.23 (10.67, 34.76)	2.47 (1.37, 4.12)
**Thailand**	0.687	288.36 (193.90, 416.34)	240.94 (145.94, 383.75)	-0.16 (-0.41, 0.17)	94.09 (50.80, 174.82)	106.45 (52.28, 210.13)	0.13 (-0.20, 0.57)	160.00 (102.88, 232.49)	103.24 (60.35, 164.62)	-0.35 (-0.55, -0.08)	34.27 (19.65, 52.75)	31.24 (17.91, 48.78)	-0.09 (-0.41, 0.43)
**Timor-Leste**	0.514	318.20 (194.34, 511.88)	869.58 (509.99, 1333.66)	1.73 (0.95, 2.83)	117.94 (56.33, 243.31)	367.30 (154.27, 720.28)	2.11 (1.15, 3.54)	182.18 (99.54, 286.83)	471.01 (243.92, 758.51)	1.59 (0.69, 2.67)	18.08 (8.48, 35.67)	31.27 (14.95, 59.39)	0.73 (0.13, 1.84)
**Togo**	0.417	221.50 (148.74, 327.68)	259.74 (171.36, 380.73)	0.17 (-0.08, 0.51)	80.42 (43.27, 147.99)	93.41 (48.87, 170.55)	0.16 (-0.09, 0.49)	136.62 (84.36, 202.81)	161.54 (96.74, 246.54)	0.18 (-0.09, 0.55)	4.46 (1.88, 9.81)	4.79 (2.00, 10.75)	0.07 (-0.26, 0.50)
**Tokelau**	0.626	848.16 (528.98, 1262.72)	876.97 (566.37, 1257.91)	0.03 (-0.23, 0.44)	225.95 (114.85, 439.44)	276.54 (153.50, 482.28)	0.22 (-0.08, 0.67)	542.30 (327.97, 844.87)	533.72 (312.71, 789.90)	-0.02 (-0.29, 0.47)	79.91 (42.20, 139.79)	66.71 (38.42, 105.93)	-0.17 (-0.49, 0.41)
**Tonga**	0.636	485.70 (328.74, 704.27)	534.43 (361.87, 763.05)	0.10 (-0.17, 0.49)	165.84 (89.08, 302.97)	202.36 (106.79, 345.75)	0.22 (-0.06, 0.62)	279.95 (170.16, 412.35)	292.06 (177.74, 429.98)	0.04 (-0.24, 0.47)	39.91 (22.86, 66.52)	40.01 (22.73, 66.36)	0.00 (-0.28, 0.41)
**Trinidad and Tobago**	0.757	879.41 (590.23, 1283.01)	445.50 (284.01, 684.33)	-0.49 (-0.63, -0.33)	450.26 (235.17, 814.65)	231.99 (123.10, 420.41)	-0.48 (-0.61, -0.32)	375.01 (224.98, 548.74)	170.87 (98.72, 267.49)	-0.54 (-0.68, -0.36)	54.14 (32.19, 80.70)	42.64 (23.46, 66.75)	-0.21 (-0.48, 0.16)
**Tunisia**	0.672	401.22 (240.07, 694.48)	470.33 (273.47, 802.48)	0.17 (-0.19, 0.70)	242.07 (113.12, 522.01)	361.23 (182.79, 667.65)	0.49 (0.05, 1.13)	134.11 (75.62, 208.48)	93.11 (52.02, 146.87)	-0.31 (-0.53, 0.08)	25.04 (11.62, 46.97)	15.99 (9.10, 26.23)	-0.36 (-0.63, 0.22)

**Turkey**	0.748	206.44 (132.62, 344.30)	258.96 (153.12, 425.83)	0.25 (-0.16, 0.76)	108.32 (56.55, 211.55)	159.78 (77.00, 316.14)	0.48 (-0.02, 1.09)	79.42 (49.66, 122.15)	82.05 (48.44, 126.47)	0.03 (-0.32, 0.50)	18.70 (9.63, 32.54)	17.13 (10.35, 25.76)	-0.08 (-0.46, 0.75)
**Turkmenistan**	0.670	298.18 (202.21, 431.08)	705.69 (407.88, 1163.89)	1.37 (0.61, 2.34)	135.35 (72.25, 239.11)	362.94 (169.62, 720.14)	1.68 (0.74, 2.96)	144.67 (88.98, 211.19)	295.82 (157.37, 505.63)	1.04 (0.35, 1.98)	18.15 (10.29, 29.56)	46.94 (25.12, 77.89)	1.59 (0.75, 2.81)
**Tuvalu**	0.589	1061.27 (684.29, 1575.69)	1228.11 (811.35, 1768.75)	0.16 (-0.17, 0.65)	251.22 (131.20, 468.16)	343.64 (187.03, 609.30)	0.37 (-0.02, 0.95)	710.91 (432.32, 1099.18)	787.09 (478.72, 1185.29)	0.11 (-0.25, 0.62)	99.14 (44.10, 189.33)	97.38 (52.10, 170.51)	-0.02 (-0.39, 0.68)
**Uganda**	0.404	351.81 (211.40, 564.00)	430.22 (256.01, 657.23)	0.22 (-0.11, 0.70)	118.77 (53.03, 260.53)	158.69 (71.65, 311.94)	0.34 (-0.07, 0.89)	221.27 (122.99, 361.54)	258.41 (136.55, 426.67)	0.17 (-0.17, 0.71)	11.77 (3.47, 30.15)	13.12 (4.60, 31.50)	0.11 (-0.23, 0.86)
**Ukraine**	0.736	363.79 (231.87, 572.04)	343.41 (226.46, 518.79)	-0.06 (-0.23, 0.19)	269.03 (148.94, 467.37)	229.59 (131.79, 381.72)	-0.15 (-0.31, 0.08)	74.97 (46.89, 109.82)	91.20 (58.15, 133.97)	0.22 (-0.06, 0.67)	19.79 (12.03, 31.48)	22.62 (13.93, 34.30)	0.14 (-0.15, 0.57)
**United Arab Emirates**	0.880	1073.36 (641.35, 1704.39)	588.30 (353.82, 922.63)	-0.45 (-0.61, -0.26)	686.96 (331.62, 1252.38)	442.31 (244.94, 758.60)	-0.36 (-0.53, -0.09)	350.89 (197.82, 549.07)	131.05 (73.70, 220.16)	-0.63 (-0.75, -0.43)	35.51 (16.63, 63.79)	14.93 (6.58, 26.85)	-0.58 (-0.74, -0.28)
**United Kingdom**	0.847	243.75 (142.83, 428.84)	167.73 (107.30, 274.11)	-0.31 (-0.42, -0.18)	168.80 (79.57, 350.28)	93.03 (45.32, 192.08)	-0.45 (-0.53, -0.34)	51.26 (31.77, 74.69)	48.76 (31.00, 71.31)	-0.05 (-0.17, 0.11)	23.68 (14.91, 34.15)	25.94 (16.72, 37.59)	0.10 (-0.06, 0.31)
**United Republic of Tanzania**	0.423	192.53 (120.64, 301.11)	331.89 (188.11, 550.38)	0.72 (0.20, 1.50)	61.97 (29.03, 133.81)	151.48 (63.53, 337.95)	1.44 (0.55, 2.59)	123.95 (71.80, 188.31)	171.26 (85.70, 297.07)	0.38 (-0.07, 1.05)	6.62 (2.39, 15.04)	9.15 (3.30, 20.79)	0.38 (-0.09, 1.38)
**United States of America**	0.859	194.83 (129.88, 299.65)	200.74 (136.64, 293.28)	0.03 (-0.09, 0.19)	116.65 (63.03, 211.13)	104.83 (56.03, 188.04)	-0.10 (-0.22, 0.05)	53.89 (35.88, 74.51)	65.44 (43.15, 88.41)	0.21 (0.08, 0.38)	24.29 (15.80, 34.34)	30.46 (20.25, 41.61)	0.25 (0.08, 0.47)

**United States Virgin Islands**	0.799	261.73 (168.61, 398.35)	351.14 (237.64, 512.62)	0.34 (0.01, 0.78)	119.44 (59.66, 238.66)	158.09 (79.62, 293.03)	0.32 (-0.02, 0.79)	114.67 (70.64, 170.45)	160.77 (94.04, 238.44)	0.40 (0.01, 0.93)	27.61 (16.86, 42.72)	32.28 (19.23, 48.17)	0.17 (-0.20, 0.69)
**Uruguay**	0.697	154.33 (96.02, 242.64)	192.52 (120.46, 303.64)	0.25 (-0.04, 0.63)	92.48 (50.01, 169.43)	110.96 (53.50, 213.85)	0.20 (-0.11, 0.58)	42.01 (26.22, 62.99)	55.79 (33.36, 84.91)	0.33 (-0.06, 0.90)	19.84 (10.89, 32.37)	25.77 (16.41, 37.72)	0.30 (-0.10, 0.85)
**Uzbekistan**	0.631	245.65 (165.66, 349.45)	1016.38 (658.81, 1526.72)	3.14 (2.11, 4.61)	116.70 (65.64, 201.57)	499.55 (239.93, 964.87)	3.28 (1.86, 5.04)	121.04 (75.40, 177.22)	489.04 (272.84, 758.61)	3.04 (1.88, 4.77)	7.91 (4.57, 12.59)	27.78 (16.38, 41.67)	2.51 (1.33, 4.62)
**Vanuatu**	0.485	895.89 (565.75, 1351.15)	1453.51 (943.94, 2107.57)	0.62 (0.20, 1.30)	241.35 (131.66, 439.58)	406.51 (220.43, 711.98)	0.68 (0.26, 1.35)	588.93 (348.39, 915.32)	949.39 (556.03, 1464.65)	0.61 (0.16, 1.36)	65.62 (30.98, 120.62)	97.61 (47.53, 175.00)	0.49 (0.03, 1.21)
**Venezuela (Bolivarian Republic of)**	0.607	384.69 (247.67, 570.70)	356.00 (215.01, 547.27)	-0.07 (-0.30, 0.25)	157.97 (69.14, 321.07)	134.99 (64.56, 261.61)	-0.15 (-0.35, 0.15)	195.14 (112.49, 300.84)	183.79 (102.30, 293.29)	-0.06 (-0.32, 0.35)	31.57 (18.94, 48.33)	37.22 (20.77, 58.98)	0.18 (-0.21, 0.71)
**Viet Nam**	0.617	455.63 (285.24, 759.63)	830.29 (504.63, 1342.06)	0.82 (0.29, 1.64)	183.41 (88.46, 394.81)	410.13 (185.77, 825.41)	1.24 (0.51, 2.39)	249.67 (145.03, 396.27)	396.16 (222.44, 651.02)	0.59 (0.08, 1.33)	22.55 (11.60, 41.74)	23.99 (12.59, 42.71)	0.06 (-0.39, 0.94)
**Yemen**	0.412	396.60 (247.33, 610.69)	626.88 (351.01, 1071.41)	0.58 (0.06, 1.31)	173.14 (90.00, 318.05)	422.47 (193.65, 847.83)	1.44 (0.70, 2.45)	193.47 (108.47, 300.45)	174.22 (87.21, 282.99)	-0.10 (-0.40, 0.38)	29.98 (9.88, 62.27)	30.20 (12.22, 61.89)	0.01 (-0.35, 0.71)
**Zambia**	0.505	355.33 (216.83, 578.93)	579.20 (346.18, 886.54)	0.63 (0.14, 1.38)	110.85 (48.20, 272.59)	202.88 (88.80, 430.59)	0.83 (0.24, 1.76)	233.81 (131.26, 371.76)	360.65 (187.28, 591.18)	0.54 (0.04, 1.28)	10.67 (3.96, 24.16)	15.67 (6.55, 33.09)	0.47 (-0.13, 1.60)
**Zimbabwe**	0.476	326.95 (195.01, 578.37)	555.02 (338.79, 933.86)	0.70 (0.23, 1.36)	192.96 (88.58, 427.61)	321.67 (151.81, 633.84)	0.67 (0.21, 1.41)	118.88 (74.11, 193.33)	206.82 (112.69, 330.39)	0.74 (0.22, 1.49)	15.11 (8.05, 31.41)	26.54 (13.05, 54.64)	0.76 (0.23, 1.54)

Deaths attributable to HFPG

**Afghanistan**	0.343	37.97 (21.04, 63.75)	53.81 (30.14, 91.92)	0.42 (0.01, 1.00)	16.35 (7.16, 37.18)	33.22 (15.47, 67.36)	1.03 (0.45, 1.89)	18.61 (9.89, 31.14)	17.56 (9.84, 28.31)	-0.06 (-0.33, 0.35)	3.01 (0.62, 7.14)	3.03 (0.80, 6.29)	0.01 (-0.33, 0.71)
**Albania**	0.681	19.13 (11.90, 30.49)	18.16 (10.45, 30.26)	-0.05 (-0.30, 0.26)	4.24 (2.11, 8.78)	4.41 (2.11, 8.95)	0.04 (-0.26, 0.41)	14.33 (8.58, 23.70)	13.21 (6.99, 23.03)	-0.08 (-0.32, 0.23)	0.56 (0.34, 0.91)	0.54 (0.28, 1.03)	-0.03 (-0.46, 0.49)
**Algeria**	0.652	30.48 (16.92, 57.94)	31.46 (16.33, 62.54)	0.03 (-0.29, 0.43)	19.75 (9.21, 45.81)	25.23 (11.14, 55.55)	0.28 (-0.10, 0.79)	9.48 (5.37, 16.22)	5.41 (3.00, 9.07)	-0.43 (-0.61, -0.18)	1.24 (0.60, 2.16)	0.82 (0.45, 1.37)	-0.34 (-0.58, 0.12)
**American Samoa**	0.712	45.39 (29.63, 65.20)	31.15 (20.83, 45.30)	-0.31 (-0.46, -0.12)	13.93 (6.68, 28.62)	10.31 (5.27, 19.93)	-0.26 (-0.43, 0.00)	29.11 (17.86, 42.86)	18.97 (12.15, 27.33)	-0.35 (-0.49, -0.15)	2.34 (1.44, 3.71)	1.87 (1.16, 2.84)	-0.20 (-0.44, 0.13)
**Andorra**	0.894	4.46 (2.45, 8.59)	5.06 (2.54, 10.37)	0.14 (-0.26, 0.63)	3.17 (1.43, 7.04)	3.37 (1.31, 8.30)	0.06 (-0.32, 0.52)	0.96 (0.55, 1.62)	1.22 (0.61, 2.28)	0.27 (-0.22, 0.94)	0.33 (0.19, 0.56)	0.47 (0.24, 0.82)	0.43 (-0.12, 1.24)
**Angola**	0.470	25.91 (14.61, 43.01)	28.03 (16.74, 45.39)	0.08 (-0.23, 0.63)	9.17 (3.75, 21.19)	12.80 (5.37, 28.70)	0.40 (-0.04, 1.14)	16.17 (8.55, 26.23)	14.65 (7.74, 24.36)	-0.09 (-0.36, 0.34)	0.57 (0.18, 1.40)	0.57 (0.22, 1.25)	0.01 (-0.38, 0.93)
**Antigua and Barbuda**	0.743	29.32 (17.57, 46.95)	23.54 (14.36, 38.54)	-0.20 (-0.36, 0.02)	14.30 (6.41, 31.41)	12.26 (5.51, 26.79)	-0.14 (-0.31, 0.09)	13.56 (7.37, 21.46)	9.84 (5.76, 14.93)	-0.27 (-0.44, -0.03)	1.45 (0.84, 2.33)	1.44 (0.88, 2.11)	-0.01 (-0.27, 0.37)
**Argentina**	0.708	13.28 (8.78, 20.54)	10.79 (6.68, 17.63)	-0.19 (-0.37, 0.05)	6.18 (3.18, 12.19)	5.40 (2.40, 11.69)	-0.13 (-0.35, 0.18)	5.99 (3.88, 8.80)	4.50 (2.60, 6.94)	-0.25 (-0.44, -0.02)	1.11 (0.71, 1.70)	0.89 (0.56, 1.32)	-0.19 (-0.40, 0.12)
**Armenia**	0.689	16.02 (8.70,	18.41 (9.97,	0.15 (-0.19,	10.47 (4.67,	12.52 (5.36,	0.20 (-0.18,	4.86 (2.59,	4.85 (2.63,	0.00 (-0.31,	0.69 (0.36,	1.04 (0.57,	0.50 (-0.08,

		29.90)	35.31)	0.65)	22.57)	28.99)	0.74)	8.57)	8.18)	0.53)	1.28)	1.64)	1.45)
**Australia**	0.839	7.60 (4.04, 15.73)	5.36 (2.69, 10.78)	-0.30 (-0.46, -0.09)	5.75 (2.60, 13.88)	3.53 (1.39, 8.59)	-0.39 (-0.55, -0.20)	1.48 (0.86, 2.50)	1.40 (0.72, 2.51)	-0.06 (-0.29, 0.21)	0.37 (0.23, 0.58)	0.43 (0.24, 0.73)	0.17 (-0.10, 0.54)
**Austria**	0.849	8.85 (4.74, 17.81)	5.51 (2.93, 11.23)	-0.38 (-0.52, -0.20)	6.82 (3.16, 15.34)	3.63 (1.51, 9.22)	-0.47 (-0.60, -0.32)	1.72 (1.02, 2.67)	1.40 (0.74, 2.49)	-0.19 (-0.40, 0.07)	0.31 (0.19, 0.46)	0.48 (0.27, 0.79)	0.56 (0.16, 1.06)
**Azerbaijan**	0.683	11.70 (7.74, 17.47)	45.19 (25.70, 76.97)	2.86 (1.73, 4.29)	3.66 (1.90, 7.12)	17.42 (7.22, 39.64)	3.76 (2.13, 5.87)	7.85 (4.72, 12.20)	27.33 (14.17, 51.02)	2.48 (1.44, 4.00)	0.19 (0.10, 0.32)	0.43 (0.21, 0.83)	1.30 (0.32, 2.93)
**Bahamas**	0.796	20.27 (12.60, 33.77)	19.58 (11.90, 31.46)	-0.03 (-0.27, 0.29)	10.43 (4.74, 22.49)	9.79 (4.55, 21.03)	-0.06 (-0.30, 0.26)	8.99 (5.30, 14.42)	8.76 (5.16, 13.32)	-0.03 (-0.28, 0.34)	0.85 (0.51, 1.36)	1.03 (0.63, 1.55)	0.22 (-0.16, 0.75)
**Bahrain**	0.751	33.04 (19.55, 58.14)	15.95 (8.95, 30.93)	-0.52 (-0.65, -0.37)	21.02 (9.49, 44.98)	11.37 (5.20, 24.90)	-0.46 (-0.61, -0.30)	10.75 (6.08, 17.21)	3.95 (2.26, 6.62)	-0.63 (-0.73, -0.50)	1.27 (0.69, 2.30)	0.63 (0.36, 1.13)	-0.50 (-0.70, -0.23)
**Bangladesh**	0.483	21.35 (13.60, 33.84)	26.91 (15.37, 45.17)	0.26 (-0.11, 0.78)	7.72 (3.62, 17.55)	12.31 (4.98, 28.08)	0.60 (0.06, 1.34)	11.73 (6.99, 18.79)	12.67 (6.63, 23.02)	0.08 (-0.24, 0.52)	1.90 (0.54, 4.05)	1.92 (0.77, 3.83)	0.01 (-0.35, 1.08)
**Barbados**	0.742	26.20 (14.89, 48.29)	19.00 (11.35, 31.68)	-0.27 (-0.46, -0.04)	17.37 (8.13, 39.01)	11.87 (5.58, 23.73)	-0.32 (-0.49, -0.08)	7.66 (4.60, 12.10)	6.02 (3.45, 9.25)	-0.21 (-0.44, 0.08)	1.17 (0.73, 1.82)	1.12 (0.69, 1.66)	-0.04 (-0.34, 0.38)
**Belarus**	0.745	14.02 (8.62, 23.82)	13.38 (7.86, 23.52)	-0.05 (-0.26, 0.23)	10.22 (5.26, 19.56)	9.85 (5.10, 19.56)	-0.04 (-0.26, 0.26)	3.23 (2.04, 4.66)	2.92 (1.71, 4.54)	-0.10 (-0.34, 0.24)	0.57 (0.34, 0.86)	0.62 (0.33, 1.01)	0.09 (-0.25, 0.60)

**Belgium**	0.851	9.24 (5.40, 17.56)	7.23 (3.86, 14.01)	-0.22 (-0.42, 0.03)	6.54 (3.13, 14.40)	4.38 (1.81, 10.73)	-0.33 (-0.52, -0.09)	2.37 (1.43, 3.70)	2.32 (1.25, 4.25)	-0.02 (-0.28, 0.31)	0.33 (0.21, 0.51)	0.53 (0.31, 0.90)	0.60 (0.13, 1.20)
**Belize**	0.603	11.41 (7.21, 18.29)	14.24 (8.76, 22.96)	0.25 (-0.05, 0.66)	5.54 (2.80, 11.52)	6.90 (3.28, 14.42)	0.24 (-0.09, 0.70)	5.32 (3.32, 7.95)	6.60 (3.83, 10.62)	0.24 (-0.09, 0.67)	0.54 (0.33, 0.81)	0.74 (0.45, 1.12)	0.37 (0.00, 0.88)
**Benin**	0.352	18.73 (10.81, 32.99)	29.52 (16.90, 50.53)	0.58 (0.15, 1.23)	7.25 (3.05, 17.86)	11.17 (4.69, 25.69)	0.54 (0.06, 1.28)	11.17 (6.01, 19.59)	17.95 (9.01, 31.09)	0.61 (0.15, 1.32)	0.31 (0.10, 0.74)	0.41 (0.14, 0.98)	0.32 (-0.14, 1.02)
**Bermuda**	0.813	12.16 (7.16, 22.24)	7.70 (4.20, 13.92)	-0.37 (-0.52, -0.17)	7.99 (3.80, 17.81)	5.09 (2.19, 11.01)	-0.36 (-0.52, -0.15)	3.54 (2.14, 5.88)	2.07 (1.15, 3.48)	-0.42 (-0.59, -0.18)	0.64 (0.37, 1.05)	0.54 (0.31, 0.89)	-0.15 (-0.42, 0.23)
**Bhutan**	0.455	11.93 (7.28, 19.75)	16.02 (8.87, 27.39)	0.34 (-0.12, 1.00)	4.45 (2.04, 10.07)	8.17 (3.35, 18.59)	0.84 (0.12, 1.76)	6.35 (3.53, 10.39)	6.84 (3.58, 11.89)	0.08 (-0.30, 0.64)	1.13 (0.33, 2.39)	1.01 (0.46, 1.86)	-0.10 (-0.47, 0.99)
**Bolivia (Plurinational State of)**	0.566	15.17 (9.28, 24.12)	16.51 (9.27, 28.32)	0.09 (-0.24, 0.55)	6.32 (2.96, 13.12)	8.22 (3.52, 17.23)	0.30 (-0.12, 0.89)	6.99 (4.11, 11.42)	6.27 (3.43, 11.26)	-0.10 (-0.38, 0.30)	1.85 (0.82, 3.27)	2.02 (1.07, 3.38)	0.09 (-0.32, 0.85)
**Bosnia and Herzegovina**	0.718	23.24 (12.96, 41.76)	42.62 (21.74, 84.90)	0.83 (0.30, 1.50)	18.05 (8.90, 36.04)	34.96 (15.46, 76.66)	0.94 (0.37, 1.68)	3.68 (2.12, 6.00)	5.76 (3.16, 9.80)	0.56 (0.11, 1.22)	1.51 (0.72, 2.79)	1.90 (1.02, 3.27)	0.26 (-0.21, 1.34)
**Botswana**	0.634	31.36 (17.23, 55.13)	40.27 (21.85, 68.87)	0.28 (-0.10, 0.90)	16.83 (7.22, 39.18)	23.18 (9.48, 51.12)	0.38 (-0.04, 1.11)	14.02 (7.49, 23.61)	16.58 (8.61, 27.92)	0.18 (-0.22, 0.81)	0.50 (0.25, 0.98)	0.51 (0.23, 1.03)	0.00 (-0.45, 0.73)
**Brazil**	0.640	30.34 (18.16, 54.78)	14.67 (8.60, 25.63)	-0.52 (-0.56, -0.47)	18.91 (8.51, 41.94)	9.08 (4.03, 19.73)	-0.52 (-0.57, -0.46)	10.22 (6.14, 15.19)	4.52 (2.67, 6.96)	-0.56 (-0.61, -0.50)	1.21 (0.75, 1.75)	1.08 (0.69, 1.57)	-0.11 (-0.26, 0.06)

**Brunei Darussalam**	0.823	56.34 (35.84, 86.96)	25.54 (15.42, 43.47)	-0.55 (-0.63, -0.42)	27.31 (12.36, 56.06)	13.53 (6.07, 29.71)	-0.50 (-0.62, -0.35)	23.70 (13.77, 35.98)	9.34 (5.51, 14.89)	-0.61 (-0.69, -0.48)	5.33 (3.04, 8.03)	2.68 (1.63, 4.08)	-0.50 (-0.62, -0.28)
**Bulgaria**	0.764	35.47 (21.83, 61.48)	46.18 (24.26, 87.09)	0.30 (-0.09, 0.78)	20.06 (9.80, 43.09)	33.02 (14.23, 73.18)	0.65 (0.18, 1.23)	14.76 (9.02, 23.34)	11.91 (6.32, 21.02)	-0.19 (-0.42, 0.12)	0.64 (0.41, 0.95)	1.25 (0.72, 2.13)	0.95 (0.33, 1.83)
**Burkina Faso**	0.257	12.87 (7.10, 23.34)	22.13 (12.88, 36.78)	0.72 (0.19, 1.46)	4.44 (1.75, 11.83)	6.78 (2.69, 15.99)	0.53 (0.09, 1.24)	8.20 (4.27, 15.19)	15.03 (7.70, 27.23)	0.83 (0.25, 1.78)	0.23 (0.07, 0.61)	0.32 (0.11, 0.78)	0.40 (-0.08, 1.44)
**Burundi**	0.284	22.58 (13.12, 38.32)	20.02 (11.17, 33.64)	-0.11 (-0.39, 0.30)	6.87 (2.68, 17.84)	7.42 (2.91, 17.46)	0.08 (-0.25, 0.62)	15.12 (8.17, 25.26)	12.03 (5.96, 20.92)	-0.20 (-0.46, 0.22)	0.59 (0.17, 1.61)	0.58 (0.15, 1.81)	-0.02 (-0.46, 0.64)
**Cabo Verde**	0.525	9.00 (5.60, 14.79)	22.27 (12.81, 37.19)	1.47 (0.81, 2.46)	2.95 (1.36, 7.02)	9.62 (4.03, 22.40)	2.26 (1.23, 3.78)	5.89 (3.40, 9.92)	12.42 (6.92, 22.26)	1.11 (0.52, 2.00)	0.15 (0.05, 0.35)	0.23 (0.10, 0.48)	0.52 (-0.07, 1.89)
**Cambodia**	0.469	18.30 (11.53, 28.36)	44.88 (26.81, 72.53)	1.45 (0.69, 2.27)	6.58 (3.01, 14.53)	19.14 (7.92, 44.02)	1.91 (0.88, 2.93)	10.85 (6.60, 16.92)	24.30 (12.65, 40.57)	1.24 (0.55, 2.01)	0.88 (0.38, 1.63)	1.44 (0.75, 2.59)	0.64 (0.02, 1.81)
**Cameroon**	0.490	14.69 (9.00, 23.27)	19.85 (11.68, 31.57)	0.35 (-0.05, 0.95)	4.98 (2.21, 10.51)	7.19 (3.23, 15.26)	0.44 (-0.03, 1.18)	9.47 (5.65, 14.92)	12.41 (6.79, 20.71)	0.31 (-0.08, 0.88)	0.24 (0.08, 0.52)	0.25 (0.09, 0.57)	0.07 (-0.31, 0.76)
**Canada**	0.873	4.47 (2.46, 8.93)	4.77 (2.42, 9.90)	0.07 (-0.24, 0.41)	3.16 (1.43, 7.74)	3.00 (1.16, 7.95)	-0.05 (-0.36, 0.31)	0.99 (0.57, 1.59)	1.29 (0.66, 2.37)	0.31 (-0.04, 0.74)	0.32 (0.20, 0.49)	0.48 (0.27, 0.83)	0.49 (0.08, 1.04)
**Central African Republic**	0.274	27.81 (16.83, 45.08)	40.14 (23.20, 65.21)	0.44 (0.05, 1.04)	8.77 (3.79, 19.46)	14.29 (5.68, 32.18)	0.63 (0.16, 1.33)	18.34 (10.67, 29.95)	24.72 (12.91, 41.36)	0.35 (-0.04, 0.95)	0.69 (0.22, 1.59)	1.13 (0.29, 3.03)	0.63 (0.01, 1.43)

**Chad**	0.238	13.68 (8.50, 22.13)	23.77 (13.72, 39.45)	0.74 (0.28, 1.34)	4.75 (2.29, 10.86)	8.26 (3.49, 19.02)	0.74 (0.23, 1.44)	8.67 (5.13, 14.00)	15.16 (8.33, 25.67)	0.75 (0.27, 1.41)	0.26 (0.09, 0.58)	0.36 (0.12, 0.81)	0.37 (-0.05, 1.06)
**Chile**	0.759	15.15 (8.77, 27.44)	13.39 (7.84, 24.45)	-0.12 (-0.32, 0.19)	8.91 (3.99, 20.05)	8.40 (3.66, 19.29)	-0.06 (-0.29, 0.31)	5.53 (3.24, 8.71)	3.89 (2.15, 6.08)	-0.30 (-0.46, -0.07)	0.71 (0.44, 1.06)	1.10 (0.65, 1.65)	0.56 (0.10, 1.18)
**China**	0.686	30.55 (19.47, 47.17)	20.13 (12.41, 32.53)	-0.34 (-0.49, -0.14)	9.94 (4.79, 20.71)	10.18 (4.93, 21.19)	0.02 (-0.24, 0.29)	16.24 (9.39, 25.89)	9.19 (5.51, 14.97)	-0.43 (-0.56, -0.29)	4.37 (2.27, 7.40)	0.77 (0.45, 1.25)	-0.82 (-0.88, -0.66)
**Colombia**	0.633	21.89 (13.13, 37.28)	9.31 (5.42, 16.02)	-0.57 (-0.68, -0.44)	12.24 (5.29, 26.42)	4.64 (1.92, 10.25)	-0.62 (-0.72, -0.51)	8.26 (4.64, 12.94)	3.03 (1.62, 5.06)	-0.63 (-0.73, -0.51)	1.39 (0.84, 2.16)	1.63 (0.91, 2.66)	0.17 (-0.24, 0.67)
**Comoros**	0.455	15.53 (8.25, 27.93)	16.67 (8.85, 30.17)	0.07 (-0.28, 0.71)	6.30 (2.43, 16.75)	7.86 (3.04, 19.15)	0.25 (-0.19, 1.01)	8.82 (4.55, 15.58)	8.38 (3.94, 14.95)	-0.05 (-0.40, 0.59)	0.40 (0.11, 1.00)	0.43 (0.12, 1.13)	0.08 (-0.38, 1.08)
**Congo**	0.568	30.86 (18.21, 50.32)	31.49 (18.77, 52.15)	0.02 (-0.26, 0.39)	11.98 (5.15, 27.46)	15.29 (6.71, 33.56)	0.28 (-0.10, 0.81)	18.31 (10.28, 29.61)	15.63 (8.49, 25.38)	-0.15 (-0.37, 0.20)	0.57 (0.23, 1.34)	0.57 (0.23, 1.21)	0.01 (-0.34, 0.60)
**Cook Islands**	0.764	29.46 (18.92, 45.33)	19.60 (12.65, 29.73)	-0.33 (-0.49, -0.10)	10.54 (4.91, 22.79)	7.90 (3.86, 16.74)	-0.25 (-0.44, 0.05)	17.35 (9.93, 27.91)	10.79 (6.63, 16.29)	-0.38 (-0.53, -0.14)	1.57 (0.91, 2.54)	0.91 (0.49, 1.49)	-0.42 (-0.62, -0.07)
**Costa Rica**	0.680	13.39 (7.65, 24.27)	8.50 (4.86, 15.06)	-0.36 (-0.52, -0.16)	7.96 (3.42, 18.73)	4.98 (2.14, 11.19)	-0.37 (-0.54, -0.17)	4.47 (2.53, 7.20)	2.62 (1.46, 4.28)	-0.41 (-0.58, -0.19)	0.96 (0.57, 1.55)	0.90 (0.51, 1.46)	-0.06 (-0.33, 0.32)
**Croatia**	0.794	29.53 (16.51, 55.06)	22.63 (11.98, 44.28)	-0.23 (-0.44, 0.04)	20.96 (9.40, 46.09)	16.48 (6.95, 36.84)	-0.21 (-0.43, 0.08)	7.64 (4.58, 11.72)	5.09 (2.73, 8.37)	-0.33 (-0.53, -0.06)	0.93 (0.57, 1.37)	1.06 (0.60, 1.74)	0.15 (-0.17, 0.63)

Cuba	0.668	16.38 (9.84, 28.24)	15.25 (8.80, 26.84)	-0.07 (-0.31, 0.23)	9.92 (4.74, 21.09)	9.92 (4.61, 20.91)	0.00 (-0.26, 0.35)	5.74 (3.53, 8.82)	4.65 (2.64, 7.26)	-0.19 (-0.41, 0.11)	0.73 (0.46, 1.09)	0.68 (0.41, 1.04)	-0.06 (-0.33, 0.35)
Cyprus	0.841	35.43 (18.68, 71.38)	13.48 (7.13, 27.78)	-0.62 (-0.69, -0.53)	23.66 (9.42, 58.85)	9.08 (3.54, 23.06)	-0.62 (-0.70, -0.52)	9.75 (5.19, 17.56)	3.54 (1.87, 6.56)	-0.64 (-0.71, -0.55)	2.02 (0.97, 3.63)	0.86 (0.48, 1.54)	-0.58 (-0.70, -0.36)
Czechia	0.828	46.60 (23.25, 98.60)	16.28 (8.88, 31.07)	-0.65 (-0.73, -0.53)	38.45 (16.98, 90.59)	12.49 (5.72, 27.39)	-0.68 (-0.75, -0.56)	6.98 (3.98, 11.19)	2.83 (1.64, 4.54)	-0.59 (-0.70, -0.46)	1.17 (0.69, 1.83)	0.96 (0.57, 1.55)	-0.18 (-0.41, 0.15)
Côte d'Ivoire	0.408	15.14 (9.14, 26.86)	24.00 (13.78, 39.46)	0.59 (0.14, 1.20)	6.16 (2.67, 14.56)	9.65 (4.01, 21.99)	0.57 (0.11, 1.23)	8.77 (5.04, 14.91)	14.06 (7.40, 24.47)	0.60 (0.13, 1.24)	0.21 (0.08, 0.48)	0.29 (0.10, 0.60)	0.39 (-0.11, 1.18)
Democratic People's Republic of Korea	0.558	22.81 (14.55, 34.71)	27.75 (17.83, 42.76)	0.22 (-0.05, 0.62)	7.91 (4.16, 15.59)	10.29 (5.24, 20.60)	0.30 (-0.02, 0.81)	13.54 (7.89, 20.43)	16.33 (9.76, 25.92)	0.21 (-0.07, 0.59)	1.36 (0.74, 2.22)	1.14 (0.57, 2.14)	-0.16 (-0.44, 0.28)
Democratic Republic of the Congo	0.382	22.18 (13.78, 36.24)	26.74 (15.42, 44.14)	0.21 (-0.15, 0.71)	8.20 (3.61, 19.00)	10.82 (4.57, 24.87)	0.32 (-0.09, 0.90)	13.44 (7.77, 21.63)	15.19 (7.99, 25.43)	0.13 (-0.23, 0.63)	0.54 (0.18, 1.29)	0.74 (0.21, 2.01)	0.37 (-0.21, 1.23)
Denmark	0.890	6.63 (3.83, 12.07)	6.48 (3.48, 12.54)	-0.02 (-0.28, 0.31)	4.38 (2.11, 9.46)	4.21 (1.78, 10.11)	-0.04 (-0.32, 0.31)	1.80 (1.08, 2.77)	1.74 (0.91, 3.08)	-0.03 (-0.30, 0.33)	0.45 (0.28, 0.68)	0.53 (0.30, 0.89)	0.19 (-0.16, 0.69)
Djibouti	0.459	15.99 (9.06, 28.67)	19.83 (11.13, 35.63)	0.24 (-0.14, 0.87)	6.54 (2.60, 16.35)	10.13 (4.19, 24.27)	0.55 (0.03, 1.44)	9.04 (4.81, 15.80)	9.23 (4.71, 15.67)	0.02 (-0.32, 0.58)	0.41 (0.14, 0.84)	0.47 (0.15, 1.01)	0.14 (-0.34, 1.06)
Dominica	0.729	25.46 (15.31, 42.62)	24.32 (14.76, 39.42)	-0.04 (-0.26, 0.24)	13.98 (6.05, 30.42)	14.03 (6.39, 28.98)	0.00 (-0.20, 0.31)	10.23 (5.58, 16.79)	8.95 (5.12, 13.94)	-0.13 (-0.36, 0.22)	1.24 (0.70, 1.97)	1.34 (0.77, 2.12)	0.08 (-0.25, 0.53)

**Dominican Republic**	0.592	7.58 (4.75, 12.66)	19.41 (10.27, 34.56)	1.56 (0.74, 2.65)	3.56 (1.62, 8.11)	10.15 (4.04, 23.63)	1.85 (0.96, 3.12)	3.71 (2.22, 5.73)	8.48 (4.28, 15.05)	1.29 (0.55, 2.34)	0.31 (0.18, 0.51)	0.79 (0.44, 1.39)	1.52 (0.50, 3.06)
**Ecuador**	0.640	11.20 (6.68, 20.89)	12.27 (6.79, 22.83)	0.10 (-0.19, 0.52)	5.91 (2.56, 14.55)	7.07 (2.91, 16.70)	0.20 (-0.13, 0.68)	4.18 (2.43, 6.71)	3.68 (1.99, 6.27)	-0.12 (-0.38, 0.25)	1.11 (0.62, 1.81)	1.52 (0.87, 2.52)	0.37 (-0.14, 1.17)
**Egypt**	0.658	11.48 (6.54, 21.24)	23.79 (12.22, 44.20)	1.07 (0.32, 2.05)	6.70 (2.89, 15.60)	18.08 (7.79, 37.47)	1.70 (0.74, 3.13)	4.22 (2.39, 7.19)	5.17 (2.50, 10.04)	0.22 (-0.26, 0.92)	0.56 (0.20, 1.07)	0.54 (0.25, 0.98)	-0.04 (-0.51, 1.12)
**El Salvador**	0.573	12.12 (7.22, 19.61)	10.65 (6.20, 18.45)	-0.12 (-0.40, 0.32)	5.10 (2.17, 11.52)	5.26 (2.23, 11.79)	0.03 (-0.32, 0.60)	5.96 (3.46, 9.56)	4.41 (2.38, 7.57)	-0.26 (-0.50, 0.13)	1.06 (0.58, 1.74)	0.98 (0.53, 1.66)	-0.08 (-0.45, 0.58)
**Equatorial Guinea**	0.685	26.01 (15.29, 42.33)	23.62 (13.26, 39.66)	-0.09 (-0.45, 0.40)	8.61 (3.62, 19.16)	11.84 (5.09, 26.16)	0.38 (-0.22, 1.17)	16.76 (9.30, 27.26)	11.40 (5.66, 20.24)	-0.32 (-0.59, 0.07)	0.63 (0.19, 1.57)	0.37 (0.17, 0.73)	-0.41 (-0.73, 0.55)
**Eritrea**	0.396	15.81 (9.06, 27.40)	21.55 (12.48, 36.77)	0.36 (-0.11, 1.10)	4.40 (1.74, 10.98)	8.02 (3.42, 17.47)	0.82 (0.08, 2.11)	10.88 (6.08, 17.83)	12.96 (6.99, 21.89)	0.19 (-0.20, 0.80)	0.52 (0.15, 1.31)	0.58 (0.21, 1.40)	0.11 (-0.35, 1.12)
**Estonia**	0.835	19.81 (11.51, 34.81)	5.92 (3.37, 10.59)	-0.70 (-0.78, -0.59)	15.94 (8.27, 30.68)	4.48 (2.20, 8.85)	-0.72 (-0.79, -0.61)	3.37 (2.20, 4.95)	1.07 (0.63, 1.71)	-0.68 (-0.76, -0.56)	0.50 (0.31, 0.75)	0.38 (0.22, 0.65)	-0.24 (-0.46, 0.11)
**Eswatini**	0.577	29.69 (18.23, 49.14)	38.88 (22.03, 63.91)	0.31 (-0.10, 0.89)	14.47 (6.53, 32.77)	20.55 (9.05, 42.82)	0.42 (-0.03, 1.15)	14.69 (8.81, 21.88)	17.71 (9.40, 29.09)	0.21 (-0.22, 0.74)	0.53 (0.29, 0.98)	0.61 (0.31, 1.09)	0.16 (-0.28, 0.80)
**Ethiopia**	0.343	18.14 (11.15, 29.47)	10.88 (6.44, 17.78)	-0.40 (-0.58, -0.15)	3.84 (1.63, 9.35)	3.75 (1.68, 8.47)	-0.02 (-0.38, 0.48)	13.62 (7.97, 21.97)	6.75 (3.67, 10.73)	-0.50 (-0.65, -0.30)	0.68 (0.18, 1.77)	0.38 (0.11, 0.97)	-0.44 (-0.64, 0.27)

**Fiji**	0.664	45.40 (28.53, 65.87)	33.57 (21.91, 51.48)	-0.26 (-0.47, 0.05)	16.10 (8.06, 33.09)	13.98 (6.93, 27.61)	-0.13 (-0.37, 0.29)	24.65 (14.62, 37.00)	16.06 (9.95, 25.47)	-0.35 (-0.54, -0.05)	4.65 (2.79, 7.54)	3.52 (2.15, 5.53)	-0.24 (-0.49, 0.15)
**Finland**	0.856	15.40 (8.49, 30.66)	10.95 (5.94, 21.59)	-0.29 (-0.45, -0.07)	11.68 (5.51, 25.64)	7.74 (3.26, 18.23)	-0.34 (-0.50, -0.11)	2.67 (1.62, 4.43)	2.37 (1.31, 4.14)	-0.11 (-0.34, 0.19)	1.06 (0.63, 1.62)	0.84 (0.52, 1.26)	-0.21 (-0.42, 0.16)
**France**	0.834	5.65 (3.23, 10.44)	3.04 (1.77, 5.58)	-0.46 (-0.54, -0.32)	3.84 (1.85, 8.36)	1.88 (0.87, 4.30)	-0.51 (-0.60, -0.37)	1.55 (0.93, 2.45)	0.89 (0.54, 1.47)	-0.43 (-0.54, -0.29)	0.26 (0.16, 0.40)	0.28 (0.17, 0.44)	0.06 (-0.13, 0.34)
**Gabon**	0.656	27.42 (16.60, 43.31)	28.12 (16.59, 45.77)	0.03 (-0.25, 0.36)	10.94 (4.81, 23.34)	13.94 (6.02, 29.08)	0.27 (-0.07, 0.77)	16.02 (8.63, 26.64)	13.69 (7.34, 22.29)	-0.15 (-0.39, 0.15)	0.47 (0.21, 1.05)	0.49 (0.23, 0.94)	0.05 (-0.34, 0.69)
**Gambia**	0.399	11.58 (6.87, 19.11)	23.84 (13.09, 39.28)	1.06 (0.44, 1.88)	4.72 (2.19, 10.53)	9.82 (4.00, 22.21)	1.08 (0.45, 1.91)	6.68 (3.95, 11.19)	13.73 (7.05, 23.86)	1.05 (0.37, 1.93)	0.18 (0.06, 0.40)	0.30 (0.10, 0.72)	0.69 (0.06, 1.55)
**Georgia**	0.702	24.00 (15.09, 38.37)	47.61 (28.69, 77.39)	0.98 (0.51, 1.55)	10.38 (4.98, 21.63)	21.81 (9.38, 48.03)	1.10 (0.53, 1.83)	12.55 (7.68, 19.12)	21.75 (11.93, 34.88)	0.73 (0.29, 1.28)	1.07 (0.66, 1.68)	4.05 (2.30, 6.65)	2.78 (1.72, 4.07)
**Germany**	0.898	23.00 (11.86, 48.88)	9.30 (5.04, 18.79)	-0.60 (-0.65, -0.52)	17.69 (7.29, 42.42)	6.51 (2.85, 15.68)	-0.63 (-0.68, -0.57)	4.41 (2.34, 7.37)	2.14 (1.17, 3.73)	-0.51 (-0.60, -0.40)	0.90 (0.50, 1.53)	0.64 (0.37, 1.01)	-0.29 (-0.44, -0.07)
**Ghana**	0.557	21.50 (12.84, 38.50)	32.97 (19.11, 57.67)	0.53 (0.12, 1.18)	10.34 (4.64, 25.39)	17.46 (7.29, 40.72)	0.69 (0.21, 1.47)	10.88 (6.29, 18.92)	15.13 (8.22, 26.24)	0.39 (0.00, 0.99)	0.28 (0.11, 0.63)	0.37 (0.16, 0.75)	0.32 (-0.09, 1.13)
**Greece**	0.794	18.20 (10.75, 33.19)	13.46 (7.34, 26.06)	-0.26 (-0.44, -0.04)	11.92 (5.80, 26.14)	8.16 (3.31, 20.70)	-0.32 (-0.50, -0.08)	5.95 (3.58, 9.45)	4.79 (2.66, 8.61)	-0.19 (-0.39, 0.03)	0.32 (0.21, 0.49)	0.51 (0.29, 0.84)	0.56 (0.17, 1.02)

**Greenland**	0.761	11.20 (6.83, 19.40)	19.03 (10.39, 35.15)	0.70 (0.26, 1.28)	6.14 (2.90, 13.89)	9.97 (3.88, 25.82)	0.62 (0.12, 1.27)	3.92 (2.37, 6.06)	7.19 (3.68, 12.16)	0.83 (0.32, 1.50)	1.13 (0.67, 1.76)	1.88 (1.00, 3.25)	0.66 (0.10, 1.37)
**Grenada**	0.669	46.81 (28.16, 80.35)	31.61 (19.53, 53.51)	-0.32 (-0.44, -0.17)	27.79 (12.87, 60.88)	18.80 (9.00, 39.05)	-0.32 (-0.46, -0.14)	17.11 (9.72, 27.09)	11.21 (6.54, 16.36)	-0.35 (-0.50, -0.11)	1.91 (1.14, 3.09)	1.59 (0.98, 2.24)	-0.16 (-0.40, 0.20)
**Guam**	0.813	18.39 (10.91, 31.33)	13.50 (8.53, 20.42)	-0.27 (-0.46, 0.00)	8.62 (3.90, 20.22)	6.11 (2.93, 12.36)	-0.29 (-0.50, 0.01)	8.56 (4.90, 14.07)	6.57 (3.95, 10.19)	-0.23 (-0.45, 0.07)	1.20 (0.70, 2.05)	0.83 (0.50, 1.32)	-0.31 (-0.53, 0.01)
**Guatemala**	0.526	13.41 (7.57, 23.24)	15.18 (9.08, 24.95)	0.13 (-0.15, 0.56)	7.06 (2.95, 16.59)	7.13 (2.98, 16.25)	0.01 (-0.29, 0.49)	5.90 (3.32, 10.17)	7.17 (4.03, 11.91)	0.22 (-0.16, 0.77)	0.45 (0.24, 0.79)	0.88 (0.51, 1.41)	0.94 (0.21, 2.22)
**Guinea**	0.325	11.62 (7.24, 19.34)	23.58 (13.70, 39.17)	1.03 (0.45, 1.84)	4.06 (1.89, 9.31)	8.53 (3.53, 19.68)	1.10 (0.46, 2.00)	7.33 (4.33, 11.97)	14.69 (7.77, 25.41)	1.01 (0.41, 1.87)	0.24 (0.07, 0.55)	0.36 (0.11, 0.85)	0.50 (-0.03, 1.38)
**Guinea-Bissau**	0.355	18.30 (11.23, 30.63)	31.35 (18.44, 51.10)	0.71 (0.17, 1.52)	5.94 (2.71, 14.04)	11.39 (4.67, 24.89)	0.92 (0.26, 1.88)	12.07 (7.05, 19.81)	19.54 (10.51, 33.72)	0.62 (0.11, 1.38)	0.30 (0.10, 0.67)	0.42 (0.14, 0.98)	0.41 (-0.10, 1.31)
**Guyana**	0.618	89.87 (57.28, 135.04)	54.15 (34.08, 84.28)	-0.40 (-0.56, -0.20)	39.74 (17.72, 75.94)	26.98 (12.84, 50.11)	-0.32 (-0.49, -0.08)	47.67 (27.42, 72.63)	25.02 (13.99, 38.50)	-0.48 (-0.62, -0.27)	2.47 (1.48, 3.71)	2.16 (1.27, 3.33)	-0.13 (-0.41, 0.28)
**Haiti**	0.432	55.18 (30.89, 91.04)	56.28 (31.79, 93.22)	0.02 (-0.24, 0.41)	23.02 (9.67, 53.22)	25.86 (10.70, 54.74)	0.12 (-0.18, 0.57)	27.91 (14.43, 43.78)	26.33 (13.04, 42.80)	-0.06 (-0.32, 0.32)	4.26 (1.34, 8.83)	4.09 (1.49, 8.62)	-0.04 (-0.39, 0.60)
**Honduras**	0.496	21.16 (12.48, 35.58)	34.71 (20.18, 58.28)	0.64 (0.26, 1.24)	11.02 (4.62, 23.39)	18.31 (8.02, 38.52)	0.66 (0.25, 1.30)	8.35 (4.60, 14.51)	13.15 (7.27, 22.81)	0.57 (0.15, 1.21)	1.80 (0.78, 3.43)	3.25 (1.69, 5.61)	0.81 (0.18, 1.99)

**Hungary**	0.791	29.10 (16.35, 55.36)	18.57 (10.11, 35.50)	-0.36 (-0.53, -0.11)	20.49 (9.43, 46.15)	14.12 (6.39, 31.29)	-0.31 (-0.49, -0.02)	7.93 (4.68, 13.42)	3.60 (2.09, 5.90)	-0.55 (-0.66, -0.38)	0.68 (0.42, 1.02)	0.85 (0.51, 1.36)	0.25 (-0.08, 0.75)
**Iceland**	0.869	6.17 (3.66, 11.22)	4.93 (2.56, 10.11)	-0.20 (-0.41, 0.07)	4.17 (2.03, 8.60)	3.26 (1.37, 8.22)	-0.22 (-0.45, 0.09)	1.60 (0.96, 2.47)	1.23 (0.64, 2.23)	-0.23 (-0.44, 0.03)	0.39 (0.25, 0.59)	0.44 (0.25, 0.77)	0.12 (-0.19, 0.57)
**India**	0.566	18.04 (11.43, 28.68)	19.66 (12.34, 30.20)	0.09 (-0.11, 0.35)	7.44 (3.43, 16.14)	8.79 (4.03, 18.32)	0.18 (-0.06, 0.50)	9.34 (5.57, 14.28)	9.68 (5.44, 15.29)	0.04 (-0.18, 0.29)	1.26 (0.38, 2.48)	1.18 (0.57, 2.07)	-0.06 (-0.33, 0.62)
**Indonesia**	0.660	21.27 (13.34, 34.15)	39.72 (23.04, 69.58)	0.87 (0.43, 1.30)	8.92 (4.01, 19.47)	20.72 (8.55, 47.27)	1.32 (0.78, 1.87)	11.52 (6.97, 17.74)	17.97 (9.44, 29.03)	0.56 (0.22, 0.93)	0.82 (0.43, 1.47)	1.03 (0.48, 1.96)	0.26 (-0.17, 0.91)
**Iran (Islamic Republic of)**	0.670	18.19 (10.16, 34.43)	19.11 (10.26, 38.34)	0.05 (-0.15, 0.30)	14.63 (7.31, 30.94)	16.06 (7.51, 35.54)	0.10 (-0.12, 0.36)	2.71 (1.61, 4.04)	2.47 (1.44, 3.95)	-0.09 (-0.28, 0.16)	0.85 (0.40, 1.54)	0.58 (0.33, 0.92)	-0.32 (-0.55, 0.20)
**Iraq**	0.671	33.96 (21.03, 56.62)	44.16 (27.52, 72.35)	0.30 (-0.09, 0.81)	21.66 (11.07, 40.97)	31.55 (16.05, 58.60)	0.46 (0.01, 1.06)	10.92 (6.54, 16.76)	11.96 (7.27, 17.67)	0.10 (-0.25, 0.59)	1.37 (0.75, 2.21)	0.64 (0.37, 1.01)	-0.53 (-0.72, -0.09)
**Ireland**	0.867	7.35 (4.11, 13.08)	6.98 (3.58, 14.54)	-0.05 (-0.32, 0.30)	5.30 (2.49, 10.84)	4.86 (1.99, 12.32)	-0.08 (-0.39, 0.31)	1.49 (0.91, 2.33)	1.47 (0.79, 2.66)	-0.01 (-0.29, 0.36)	0.56 (0.36, 0.83)	0.65 (0.38, 1.10)	0.16 (-0.18, 0.70)
**Israel**	0.803	8.75 (5.16, 15.58)	6.77 (3.73, 12.63)	-0.23 (-0.42, 0.00)	5.07 (2.32, 11.12)	3.83 (1.60, 9.42)	-0.25 (-0.45, 0.03)	3.35 (1.94, 5.67)	2.50 (1.36, 4.26)	-0.26 (-0.44, -0.03)	0.33 (0.20, 0.53)	0.45 (0.23, 0.79)	0.37 (-0.06, 0.96)
**Italy**	0.801	17.45 (9.18, 34.89)	9.48 (5.16, 19.15)	-0.46 (-0.55, -0.35)	13.14 (5.65, 30.54)	6.04 (2.37, 15.47)	-0.54 (-0.63, -0.43)	3.75 (2.20, 5.99)	2.87 (1.62, 5.14)	-0.24 (-0.36, -0.07)	0.56 (0.35, 0.88)	0.57 (0.34, 0.97)	0.02 (-0.14, 0.23)

**Jamaica**	0.684	29.07 (16.60, 53.20)	29.69 (17.63, 49.75)	0.02 (-0.21, 0.33)	17.01 (7.20, 39.53)	15.52 (6.48, 34.69)	-0.09 (-0.29, 0.20)	10.86 (6.06, 17.96)	12.53 (6.85, 19.85)	0.15 (-0.18, 0.59)	1.20 (0.70, 1.95)	1.65 (0.94, 2.62)	0.37 (-0.06, 0.96)
**Japan**	0.870	11.33 (6.64, 20.64)	4.49 (2.66, 8.03)	-0.60 (-0.66, -0.54)	7.65 (3.64, 17.12)	2.52 (1.14, 5.80)	-0.67 (-0.72, -0.63)	2.81 (1.71, 4.40)	1.40 (0.85, 2.32)	-0.50 (-0.56, -0.43)	0.86 (0.55, 1.25)	0.56 (0.36, 0.87)	-0.34 (-0.42, -0.23)
**Jordan**	0.731	38.42 (21.55, 68.78)	22.54 (12.26, 40.83)	-0.41 (-0.57, -0.21)	28.97 (13.96, 59.88)	17.77 (8.40, 35.37)	-0.39 (-0.55, -0.18)	8.82 (4.99, 13.91)	4.43 (2.48, 7.07)	-0.50 (-0.64, -0.29)	0.63 (0.32, 1.07)	0.35 (0.19, 0.56)	-0.45 (-0.65, -0.10)
**Kazakhstan**	0.723	24.36 (13.96, 43.61)	50.13 (29.10, 89.96)	1.06 (0.51, 1.78)	15.83 (7.30, 34.00)	31.98 (13.82, 68.72)	1.02 (0.42, 1.81)	7.64 (4.27, 12.65)	16.11 (8.95, 26.05)	1.11 (0.46, 2.08)	0.88 (0.48, 1.55)	2.04 (1.17, 3.18)	1.31 (0.52, 2.56)
**Kenya**	0.508	12.87 (7.28, 21.40)	15.92 (9.16, 26.10)	0.24 (0.05, 0.48)	4.76 (1.97, 11.54)	6.27 (2.72, 14.39)	0.32 (0.09, 0.61)	7.78 (4.01, 12.66)	9.23 (4.73, 14.71)	0.19 (-0.03, 0.44)	0.32 (0.12, 0.74)	0.42 (0.15, 0.96)	0.31 (0.07, 0.75)
**Kiribati**	0.527	79.31 (50.95, 115.00)	78.78 (52.14, 112.28)	-0.01 (-0.29, 0.37)	19.17 (9.89, 36.47)	22.51 (11.80, 41.28)	0.17 (-0.19, 0.66)	55.80 (34.52, 83.38)	51.30 (31.84, 76.39)	-0.08 (-0.35, 0.30)	4.33 (2.54, 7.04)	4.98 (2.91, 7.86)	0.15 (-0.24, 0.65)
**Kuwait**	0.851	15.54 (8.56, 28.81)	14.05 (8.27, 25.43)	-0.10 (-0.29, 0.17)	11.95 (5.53, 25.60)	10.47 (5.14, 21.21)	-0.12 (-0.31, 0.14)	3.07 (1.70, 5.23)	3.12 (1.84, 5.04)	0.02 (-0.26, 0.43)	0.52 (0.30, 0.82)	0.46 (0.27, 0.72)	-0.12 (-0.38, 0.29)
**Kyrgyzstan**	0.596	15.35 (9.50, 25.67)	13.64 (8.17, 23.37)	-0.11 (-0.29, 0.16)	9.49 (4.76, 18.74)	8.47 (3.99, 17.66)	-0.11 (-0.30, 0.19)	5.34 (3.38, 7.94)	4.53 (2.77, 6.79)	-0.15 (-0.33, 0.12)	0.53 (0.32, 0.81)	0.65 (0.40, 0.97)	0.22 (-0.09, 0.66)
**Lao People's Democratic Republic**	0.490	32.56 (19.48, 54.96)	51.19 (30.58, 79.34)	0.57 (0.10, 1.26)	12.79 (5.85, 29.75)	22.91 (9.87, 48.61)	0.79 (0.23, 1.69)	18.42 (10.32, 29.79)	26.78 (14.37, 42.10)	0.45 (0.01, 1.05)	1.36 (0.55, 2.67)	1.50 (0.73, 3.04)	0.10 (-0.31, 0.95)

**Latvia**	0.820	21.68 (12.87, 36.78)	15.81 (8.91, 28.02)	-0.27 (-0.42, -0.08)	16.27 (8.22, 30.74)	12.90 (6.67, 24.53)	-0.21 (-0.36, -0.01)	4.81 (3.08, 7.06)	2.41 (1.45, 3.56)	-0.50 (-0.61, -0.35)	0.59 (0.36, 0.89)	0.50 (0.32, 0.74)	-0.15 (-0.37, 0.24)
**Lebanon**	0.708	12.23 (6.36, 24.99)	11.23 (4.98, 22.58)	-0.08 (-0.40, 0.34)	8.65 (3.66, 21.87)	9.52 (3.55, 20.93)	0.10 (-0.29, 0.64)	3.12 (1.71, 5.60)	1.51 (0.77, 2.80)	-0.52 (-0.68, -0.25)	0.47 (0.21, 0.91)	0.20 (0.10, 0.39)	-0.57 (-0.80, -0.08)
**Lesotho**	0.507	27.85 (15.56, 50.01)	47.72 (28.33, 81.21)	0.71 (0.18, 1.57)	12.11 (4.54, 30.44)	20.38 (7.80, 46.78)	0.68 (0.20, 1.50)	15.08 (7.41, 26.42)	26.38 (13.46, 45.03)	0.75 (0.15, 1.72)	0.66 (0.24, 1.61)	0.96 (0.43, 1.82)	0.45 (-0.14, 1.54)
**Liberia**	0.370	20.86 (11.70, 36.67)	25.25 (14.26, 41.08)	0.21 (-0.12, 0.76)	8.34 (3.39, 20.60)	9.90 (3.97, 21.83)	0.19 (-0.14, 0.70)	12.17 (6.36, 21.80)	14.93 (7.58, 25.70)	0.23 (-0.13, 0.80)	0.35 (0.10, 0.92)	0.41 (0.11, 1.20)	0.17 (-0.30, 0.95)
**Libya**	0.709	21.66 (11.44, 42.24)	23.11 (12.17, 44.44)	0.07 (-0.21, 0.50)	14.24 (5.73, 34.06)	18.33 (7.93, 38.71)	0.29 (-0.05, 0.83)	6.48 (3.38, 10.59)	4.19 (2.34, 6.85)	-0.35 (-0.54, -0.02)	0.94 (0.43, 1.78)	0.59 (0.31, 1.02)	-0.38 (-0.61, 0.11)
**Lithuania**	0.843	8.72 (5.16, 15.37)	8.65 (4.99, 16.34)	-0.01 (-0.20, 0.25)	6.80 (3.54, 13.46)	6.93 (3.51, 14.12)	0.02 (-0.18, 0.28)	1.53 (0.97, 2.24)	1.33 (0.82, 1.99)	-0.13 (-0.32, 0.10)	0.39 (0.23, 0.57)	0.39 (0.23, 0.59)	0.01 (-0.22, 0.31)
**Luxembourg**	0.895	10.84 (6.14, 20.39)	9.24 (5.12, 17.66)	-0.15 (-0.36, 0.13)	7.84 (3.82, 17.10)	5.83 (2.43, 14.43)	-0.26 (-0.46, 0.01)	2.70 (1.61, 4.18)	2.85 (1.58, 5.19)	0.06 (-0.23, 0.44)	0.31 (0.20, 0.47)	0.57 (0.33, 0.95)	0.84 (0.30, 1.57)
**Madagascar**	0.396	18.34 (11.08, 30.51)	23.61 (13.70, 39.41)	0.29 (-0.10, 0.86)	6.03 (2.61, 15.65)	9.04 (3.74, 20.21)	0.50 (-0.01, 1.29)	11.73 (6.63, 19.01)	13.90 (7.46, 23.21)	0.18 (-0.17, 0.72)	0.58 (0.18, 1.41)	0.67 (0.20, 1.68)	0.17 (-0.24, 0.79)
**Malawi**	0.384	22.11 (11.93, 38.48)	24.97 (14.15, 41.61)	0.13 (-0.18, 0.64)	9.64 (3.70, 23.75)	11.62 (4.70, 26.46)	0.21 (-0.14, 0.77)	11.98 (6.00, 21.90)	12.75 (6.24, 21.39)	0.06 (-0.25, 0.64)	0.49 (0.14, 1.31)	0.60 (0.16, 1.59)	0.22 (-0.22, 0.91)

**Malaysia**	0.737	29.05 (19.42, 42.90)	24.58 (15.08, 39.85)	-0.15 (-0.40, 0.19)	10.76 (5.31, 21.22)	11.80 (5.30, 23.64)	0.10 (-0.24, 0.58)	16.84 (10.41, 24.96)	11.91 (6.63, 19.69)	-0.29 (-0.49, -0.01)	1.45 (0.83, 2.28)	0.87 (0.51, 1.48)	-0.40 (-0.64, 0.03)
**Maldives**	0.562	23.39 (13.92, 38.71)	16.86 (9.53, 30.31)	-0.28 (-0.49, 0.02)	9.25 (4.05, 22.01)	8.80 (3.76, 20.86)	-0.05 (-0.37, 0.37)	12.70 (7.54, 21.19)	7.23 (3.90, 13.09)	-0.43 (-0.59, -0.21)	1.43 (0.57, 2.83)	0.82 (0.43, 1.56)	-0.42 (-0.69, 0.37)
**Mali**	0.263	13.90 (8.38, 22.62)	22.37 (12.31, 36.71)	0.61 (0.15, 1.22)	4.79 (2.26, 10.63)	8.04 (3.33, 20.19)	0.68 (0.15, 1.42)	8.85 (5.05, 14.34)	13.99 (7.16, 24.50)	0.58 (0.11, 1.25)	0.26 (0.08, 0.63)	0.35 (0.11, 0.90)	0.31 (-0.13, 1.04)
**Malta**	0.801	21.28 (11.37, 38.83)	9.73 (5.25, 19.08)	-0.54 (-0.64, -0.42)	14.34 (5.93, 31.74)	6.68 (2.76, 15.70)	-0.53 (-0.65, -0.39)	6.34 (3.51, 10.90)	2.63 (1.48, 4.39)	-0.59 (-0.67, -0.46)	0.60 (0.35, 0.93)	0.43 (0.26, 0.67)	-0.28 (-0.46, -0.06)
**Marshall Islands**	0.544	74.20 (48.31, 108.75)	62.56 (39.91, 94.34)	-0.16 (-0.36, 0.11)	18.44 (8.59, 39.44)	16.76 (8.59, 34.31)	-0.09 (-0.32, 0.22)	49.29 (29.95, 75.60)	40.91 (24.84, 64.00)	-0.17 (-0.38, 0.14)	6.47 (2.75, 12.36)	4.89 (2.26, 8.79)	-0.24 (-0.47, 0.18)
**Mauritania**	0.496	11.84 (7.63, 19.36)	11.06 (6.05, 19.94)	-0.07 (-0.35, 0.33)	4.87 (2.40, 10.87)	4.98 (2.10, 12.13)	0.02 (-0.32, 0.50)	6.78 (4.23, 10.49)	5.93 (3.11, 10.53)	-0.12 (-0.40, 0.27)	0.19 (0.08, 0.37)	0.14 (0.06, 0.31)	-0.25 (-0.55, 0.21)
**Mauritius**	0.705	40.45 (23.77, 68.53)	18.68 (11.79, 29.49)	-0.54 (-0.66, -0.36)	24.92 (11.47, 52.54)	10.66 (5.25, 20.78)	-0.57 (-0.69, -0.40)	14.36 (8.73, 22.39)	6.80 (4.18, 10.03)	-0.53 (-0.66, -0.34)	1.16 (0.73, 1.75)	1.22 (0.76, 1.79)	0.04 (-0.26, 0.48)
**Mexico**	0.649	18.11 (11.11, 31.02)	11.08 (7.05, 18.44)	-0.39 (-0.47, -0.28)	11.28 (5.31, 24.17)	6.20 (2.97, 13.78)	-0.45 (-0.53, -0.37)	5.96 (3.64, 9.08)	3.74 (2.29, 5.51)	-0.37 (-0.47, -0.26)	0.87 (0.55, 1.26)	1.14 (0.72, 1.67)	0.32 (-0.13, 0.64)
**Micronesia (Federated States of)**	0.580	42.63 (25.35, 68.76)	62.65 (37.94, 93.64)	0.47 (-0.03, 1.14)	11.21 (5.19, 24.31)	17.61 (8.35, 35.55)	0.57 (0.04, 1.44)	27.85 (16.05, 45.04)	40.89 (23.25, 66.58)	0.47 (-0.03, 1.18)	3.56 (1.48, 7.24)	4.16 (1.87, 7.59)	0.17 (-0.29, 1.00)

**Monaco**	0.902	8.99 (4.87, 17.12)	7.06 (3.55, 14.75)	-0.21 (-0.45, 0.21)	6.65 (3.08, 14.36)	5.24 (2.20, 12.53)	-0.21 (-0.45, 0.21)	1.85 (1.08, 2.96)	1.47 (0.79, 2.75)	-0.20 (-0.47, 0.27)	0.49 (0.27, 0.80)	0.35 (0.19, 0.61)	-0.29 (-0.56, 0.14)
**Mongolia**	0.606	11.02 (6.70, 18.40)	17.70 (10.66, 28.22)	0.61 (0.19, 1.18)	1.07 (0.52, 2.52)	2.31 (1.16, 4.88)	1.16 (0.46, 2.03)	9.22 (5.41, 15.39)	14.22 (8.28, 22.94)	0.54 (0.13, 1.11)	0.73 (0.36, 1.28)	1.16 (0.66, 1.97)	0.59 (-0.01, 1.69)
**Montenegro**	0.791	36.94 (21.58, 62.08)	61.79 (35.98, 100.87)	0.67 (0.23, 1.24)	6.62 (3.01, 15.06)	14.32 (6.21, 31.40)	1.16 (0.57, 2.03)	29.87 (15.98, 52.45)	46.70 (25.00, 83.94)	0.56 (0.15, 1.09)	0.45 (0.26, 0.74)	0.76 (0.44, 1.25)	0.69 (0.16, 1.49)
**Morocco**	0.548	19.17 (11.12, 35.30)	38.04 (20.31, 69.32)	0.98 (0.37, 1.76)	11.43 (5.29, 25.88)	29.50 (13.45, 59.62)	1.58 (0.82, 2.57)	6.55 (3.59, 10.79)	7.36 (4.00, 11.50)	0.12 (-0.23, 0.64)	1.18 (0.43, 2.43)	1.18 (0.57, 2.07)	-0.01 (-0.40, 0.98)
**Mozambique**	0.307	19.67 (11.44, 33.16)	34.25 (19.58, 56.00)	0.74 (0.20, 1.55)	7.92 (3.23, 18.64)	15.19 (6.24, 32.76)	0.92 (0.23, 2.03)	11.16 (5.92, 19.39)	18.32 (9.43, 31.47)	0.64 (0.11, 1.52)	0.59 (0.14, 1.63)	0.73 (0.23, 1.85)	0.24 (-0.20, 1.51)
**Myanmar**	0.521	43.65 (26.69, 70.51)	59.73 (37.21, 96.01)	0.37 (0.00, 0.93)	16.71 (7.59, 37.69)	28.59 (12.46, 60.81)	0.71 (0.18, 1.47)	25.04 (14.36, 38.88)	29.51 (16.33, 47.02)	0.18 (-0.16, 0.65)	1.90 (0.84, 3.61)	1.63 (0.88, 2.81)	-0.14 (-0.49, 0.52)
**Namibia**	0.612	33.31 (18.89, 58.06)	33.62 (18.35, 57.56)	0.01 (-0.27, 0.39)	16.98 (7.16, 40.12)	18.69 (8.00, 41.69)	0.10 (-0.21, 0.58)	15.72 (8.46, 26.23)	14.41 (7.36, 23.46)	-0.08 (-0.36, 0.33)	0.61 (0.30, 1.15)	0.52 (0.26, 0.97)	-0.15 (-0.48, 0.41)
**Nauru**	0.618	51.86 (32.40, 79.34)	68.17 (43.69, 100.36)	0.31 (0.02, 0.74)	18.29 (8.54, 38.44)	24.51 (11.86, 46.68)	0.34 (0.03, 0.85)	31.09 (18.17, 50.56)	40.64 (23.48, 63.25)	0.31 (-0.01, 0.76)	2.48 (1.39, 4.05)	3.02 (1.61, 4.79)	0.22 (-0.19, 0.81)
**Nepal**	0.422	12.70 (7.75, 20.01)	17.31 (10.22, 28.92)	0.36 (-0.09, 0.92)	4.82 (2.08, 10.64)	8.08 (3.43, 17.99)	0.68 (0.08, 1.46)	6.67 (3.70, 10.49)	8.00 (4.40, 14.07)	0.20 (-0.21, 0.77)	1.20 (0.35, 2.55)	1.23 (0.46, 2.62)	0.02 (-0.34, 0.81)

Netherlands	0.883	9.18 (4.64, 19.62)	6.36 (3.21, 13.35)	-0.31 (-0.49, -0.01)	6.66 (2.76, 16.90)	4.32 (1.74, 11.19)	-0.35 (-0.54, -0.06)	2.02 (1.12, 3.62)	1.61 (0.85, 3.01)	-0.20 (-0.43, 0.12)	0.50 (0.30, 0.83)	0.43 (0.25, 0.72)	-0.13 (-0.37, 0.21)
New Zealand	0.840	6.80 (3.81, 12.72)	5.78 (3.02, 12.56)	-0.15 (-0.37, 0.17)	4.85 (2.20, 10.71)	3.99 (1.57, 10.56)	-0.18 (-0.41, 0.15)	1.39 (0.84, 2.15)	1.25 (0.70, 2.33)	-0.10 (-0.33, 0.23)	0.55 (0.35, 0.81)	0.53 (0.32, 0.85)	-0.04 (-0.26, 0.31)
Nicaragua	0.517	19.72 (11.64, 31.81)	17.72 (10.29, 30.77)	-0.10 (-0.30, 0.16)	10.18 (4.36, 22.42)	10.09 (4.43, 22.20)	-0.01 (-0.24, 0.29)	8.56 (4.53, 13.31)	6.60 (3.58, 10.49)	-0.23 (-0.41, 0.02)	0.98 (0.53, 1.50)	1.03 (0.60, 1.60)	0.05 (-0.30, 0.55)
Niger	0.162	7.03 (4.15, 11.54)	21.15 (10.90, 36.59)	2.01 (1.05, 3.26)	2.56 (1.09, 6.21)	7.53 (2.88, 19.70)	1.94 (0.95, 3.17)	4.34 (2.40, 7.15)	13.28 (6.14, 23.86)	2.06 (0.99, 3.33)	0.12 (0.04, 0.30)	0.34 (0.09, 0.97)	1.72 (0.59, 3.35)
Nigeria	0.515	15.98 (8.56, 28.58)	15.91 (8.53, 28.29)	0.00 (-0.32, 0.39)	6.34 (2.58, 16.45)	7.13 (2.88, 18.19)	0.12 (-0.24, 0.59)	9.41 (4.81, 16.55)	8.60 (4.30, 15.61)	-0.09 (-0.37, 0.29)	0.23 (0.08, 0.56)	0.19 (0.06, 0.41)	-0.20 (-0.46, 0.20)
Niue	0.711	49.37 (31.26, 72.51)	37.19 (23.47, 55.82)	-0.25 (-0.44, 0.00)	16.83 (7.80, 34.87)	13.46 (6.40, 25.91)	-0.20 (-0.40, 0.07)	29.58 (17.06, 45.53)	21.86 (13.18, 33.96)	-0.26 (-0.45, 0.02)	2.96 (1.74, 4.78)	1.87 (1.03, 3.03)	-0.37 (-0.61, 0.00)
North Macedonia	0.744	55.82 (31.48, 101.34)	87.35 (47.12, 165.22)	0.56 (0.09, 1.17)	39.76 (18.61, 83.28)	65.78 (28.13, 142.34)	0.65 (0.15, 1.33)	14.33 (8.03, 22.78)	18.36 (10.30, 30.88)	0.28 (-0.11, 0.80)	1.72 (0.99, 2.89)	3.20 (1.83, 5.15)	0.86 (0.12, 1.85)
Northern Mariana Islands	0.771	35.42 (22.02, 56.73)	30.95 (20.45, 46.16)	-0.13 (-0.33, 0.16)	12.18 (5.69, 28.09)	10.12 (4.88, 21.40)	-0.17 (-0.37, 0.16)	21.05 (12.53, 33.82)	18.93 (11.45, 28.36)	-0.10 (-0.32, 0.21)	2.19 (1.29, 3.64)	1.90 (1.13, 2.97)	-0.13 (-0.40, 0.26)
Norway	0.913	13.90 (7.44, 27.77)	7.52 (4.04, 15.17)	-0.46 (-0.54, -0.37)	10.62 (4.70, 24.23)	5.07 (2.10, 12.43)	-0.52 (-0.60, -0.43)	2.45 (1.45, 4.04)	1.79 (1.00, 3.33)	-0.27 (-0.39, -0.12)	0.83 (0.51, 1.27)	0.66 (0.39, 1.12)	-0.21 (-0.35, 0.00)

**Oman**	0.783	25.86 (15.44, 43.76)	33.25 (18.48, 61.52)	0.29 (-0.17, 0.96)	15.09 (7.45, 30.68)	24.89 (11.24, 52.99)	0.65 (0.08, 1.49)	9.64 (5.33, 15.75)	7.49 (4.18, 12.31)	-0.22 (-0.47, 0.19)	1.13 (0.57, 1.98)	0.87 (0.38, 1.58)	-0.23 (-0.61, 0.48)
**Pakistan**	0.449	17.72 (11.01, 28.29)	29.00 (17.90, 45.21)	0.64 (0.24, 1.24)	7.20 (3.32, 15.97)	13.76 (6.05, 28.19)	0.91 (0.40, 1.76)	9.08 (5.30, 13.67)	13.47 (7.94, 20.77)	0.48 (0.10, 1.06)	1.44 (0.50, 2.71)	1.77 (0.84, 3.08)	0.23 (-0.15, 0.98)
**Palau**	0.738	38.37 (23.73, 60.85)	39.26 (25.15, 58.70)	0.02 (-0.28, 0.45)	15.50 (7.60, 32.76)	17.00 (8.89, 32.11)	0.10 (-0.24, 0.64)	21.20 (12.49, 34.16)	20.61 (12.65, 31.61)	-0.03 (-0.33, 0.43)	1.68 (0.93, 2.77)	1.64 (1.00, 2.59)	-0.02 (-0.39, 0.64)
**Palestine**	0.588	42.35 (22.51, 76.33)	41.04 (22.21, 78.84)	-0.03 (-0.31, 0.37)	28.52 (12.19, 61.77)	32.49 (14.69, 69.29)	0.14 (-0.17, 0.60)	13.02 (6.79, 21.75)	7.97 (4.39, 12.78)	-0.39 (-0.58, -0.11)	0.81 (0.39, 1.45)	0.58 (0.33, 0.93)	-0.28 (-0.55, 0.22)
**Panama**	0.686	19.96 (11.25, 35.83)	13.47 (7.87, 23.96)	-0.33 (-0.49, -0.08)	10.85 (4.64, 24.64)	7.30 (3.05, 17.16)	-0.33 (-0.51, -0.10)	7.92 (4.42, 13.05)	4.85 (2.66, 7.83)	-0.39 (-0.56, -0.10)	1.19 (0.67, 2.05)	1.32 (0.75, 2.14)	0.11 (-0.25, 0.70)
**Papua New Guinea**	0.394	29.81 (17.17, 49.94)	45.60 (27.58, 71.09)	0.53 (0.13, 1.14)	5.81 (2.38, 13.23)	9.52 (4.26, 19.86)	0.64 (0.16, 1.39)	21.80 (12.27, 38.77)	32.88 (18.42, 52.37)	0.51 (0.10, 1.17)	2.20 (0.75, 4.84)	3.20 (1.29, 6.50)	0.46 (0.02, 1.28)
**Paraguay**	0.638	15.22 (8.52, 27.86)	17.09 (9.25, 31.29)	0.12 (-0.23, 0.68)	8.19 (3.39, 20.51)	10.14 (4.03, 22.54)	0.24 (-0.16, 0.93)	6.22 (3.58, 10.24)	5.79 (3.09, 9.65)	-0.07 (-0.38, 0.40)	0.81 (0.48, 1.26)	1.16 (0.63, 1.96)	0.43 (-0.10, 1.36)
**Peru**	0.648	6.63 (4.11, 10.89)	4.43 (2.35, 8.41)	-0.33 (-0.56, 0.03)	3.17 (1.52, 6.65)	2.52 (1.05, 5.95)	-0.21 (-0.49, 0.24)	2.88 (1.73, 4.37)	1.51 (0.79, 2.73)	-0.48 (-0.66, -0.19)	0.57 (0.32, 0.94)	0.40 (0.22, 0.73)	-0.30 (-0.57, 0.19)
**Philippines**	0.623	16.12 (9.42, 28.10)	22.28 (13.98, 35.47)	0.38 (0.02, 0.80)	8.23 (3.67, 19.09)	9.97 (4.62, 20.57)	0.21 (-0.11, 0.63)	7.50 (4.29, 12.86)	11.64 (6.48, 18.79)	0.55 (0.10, 1.07)	0.38 (0.20, 0.71)	0.67 (0.38, 1.05)	0.75 (0.17, 1.56)

**Poland**	0.802	21.83 (13.35, 36.72)	16.85 (9.42, 29.34)	-0.23 (-0.39, -0.04)	14.89 (7.73, 29.21)	12.25 (5.69, 24.67)	-0.18 (-0.37, 0.04)	5.98 (3.78, 8.97)	3.77 (2.24, 5.80)	-0.37 (-0.50, -0.22)	0.97 (0.64, 1.35)	0.83 (0.52, 1.22)	-0.14 (-0.33, 0.09)
**Portugal**	0.743	37.63 (20.42, 70.57)	16.32 (9.28, 31.86)	-0.57 (-0.68, -0.42)	27.02 (12.26, 60.21)	10.83 (4.68, 25.57)	-0.60 (-0.71, -0.45)	9.79 (5.78, 16.04)	4.61 (2.65, 7.43)	-0.53 (-0.65, -0.36)	0.81 (0.50, 1.23)	0.88 (0.52, 1.40)	0.09 (-0.18, 0.46)
**Puerto Rico**	0.814	12.53 (7.33, 22.11)	6.48 (4.04, 10.63)	-0.48 (-0.62, -0.29)	7.59 (3.46, 16.33)	3.39 (1.54, 7.04)	-0.55 (-0.66, -0.39)	4.19 (2.62, 6.22)	2.48 (1.49, 3.84)	-0.41 (-0.58, -0.17)	0.74 (0.45, 1.13)	0.61 (0.38, 0.92)	-0.18 (-0.43, 0.20)
**Qatar**	0.830	26.14 (14.64, 48.42)	15.07 (8.24, 28.86)	-0.42 (-0.61, -0.17)	16.55 (7.23, 36.97)	10.51 (4.80, 23.65)	-0.36 (-0.57, -0.07)	7.51 (4.11, 12.22)	3.55 (2.02, 6.41)	-0.53 (-0.69, -0.29)	2.08 (0.98, 3.81)	1.01 (0.57, 1.76)	-0.52 (-0.74, -0.08)
**Republic of Korea**	0.878	29.03 (18.54, 45.39)	9.87 (5.27, 19.47)	-0.66 (-0.76, -0.45)	14.33 (7.14, 27.92)	6.44 (2.68, 15.75)	-0.55 (-0.70, -0.27)	12.93 (7.83, 19.26)	2.74 (1.48, 4.78)	-0.79 (-0.84, -0.69)	1.77 (0.97, 2.74)	0.69 (0.40, 1.15)	-0.61 (-0.73, -0.39)
**Republic of Moldova**	0.696	19.97 (12.78, 32.49)	15.77 (9.60, 26.66)	-0.21 (-0.38, 0.03)	10.39 (5.16, 22.31)	10.28 (4.98, 20.79)	-0.01 (-0.26, 0.33)	9.19 (5.82, 13.97)	5.12 (3.17, 7.81)	-0.44 (-0.57, -0.27)	0.39 (0.24, 0.60)	0.36 (0.23, 0.54)	-0.07 (-0.31, 0.34)
**Romania**	0.760	24.19 (14.32, 41.13)	20.80 (12.18, 37.40)	-0.14 (-0.31, 0.07)	16.39 (8.28, 32.62)	14.15 (6.80, 29.66)	-0.14 (-0.33, 0.12)	6.76 (4.28, 10.27)	5.30 (3.26, 8.13)	-0.22 (-0.40, 0.05)	1.04 (0.61, 1.75)	1.35 (0.82, 2.12)	0.30 (-0.11, 0.95)
**Russian Federation**	0.805	29.78 (17.82, 50.90)	23.81 (14.29, 40.61)	-0.20 (-0.35, -0.01)	23.36 (12.68, 44.28)	18.18 (9.75, 34.78)	-0.22 (-0.37, -0.04)	5.57 (3.69, 8.06)	4.45 (2.92, 6.41)	-0.20 (-0.36, -0.01)	0.84 (0.55, 1.19)	1.18 (0.76, 1.70)	0.41 (0.11, 0.80)
**Rwanda**	0.429	27.66 (13.80, 48.51)	23.07 (12.14, 39.43)	-0.17 (-0.43, 0.26)	8.33 (2.83, 21.70)	8.49 (3.10, 21.73)	0.02 (-0.32, 0.55)	18.58 (8.06, 34.65)	13.95 (6.13, 24.39)	-0.25 (-0.50, 0.21)	0.74 (0.18, 2.00)	0.63 (0.18, 1.66)	-0.16 (-0.50, 0.73)

**Saint Kitts and Nevis**	0.746	77.92 (46.22, 132.57)	41.62 (25.41, 70.40)	-0.47 (-0.56, -0.35)	44.03 (19.49, 94.06)	24.31 (11.21, 51.22)	-0.45 (-0.55, -0.32)	31.81 (18.32, 50.39)	15.75 (9.22, 24.00)	-0.50 (-0.62, -0.35)	2.08 (1.21, 3.28)	1.56 (0.94, 2.37)	-0.25 (-0.46, 0.06)
**Saint Lucia**	0.670	53.05 (32.19, 89.06)	27.80 (17.27, 46.19)	-0.48 (-0.58, -0.36)	30.84 (14.44, 66.08)	15.80 (7.16, 33.01)	-0.49 (-0.59, -0.34)	20.23 (11.82, 31.32)	10.64 (6.28, 16.25)	-0.47 (-0.59, -0.33)	1.98 (1.19, 3.13)	1.36 (0.85, 1.98)	-0.31 (-0.50, -0.06)
**Saint Vincent and the Grenadines**	0.627	36.98 (21.01, 68.09)	28.90 (17.93, 47.28)	-0.22 (-0.37, -0.01)	26.59 (12.10, 57.36)	15.87 (7.57, 32.18)	-0.40 (-0.51, -0.28)	8.98 (5.24, 13.73)	11.96 (7.20, 17.91)	0.33 (0.07, 0.67)	1.41 (0.85, 2.19)	1.07 (0.68, 1.56)	-0.24 (-0.45, 0.03)
**Samoa**	0.641	48.07 (28.68, 73.36)	46.13 (29.39, 68.02)	-0.04 (-0.25, 0.27)	14.34 (6.34, 32.03)	14.66 (6.87, 30.40)	0.02 (-0.21, 0.37)	30.67 (17.16, 48.15)	28.76 (16.78, 44.74)	-0.06 (-0.28, 0.28)	3.06 (1.53, 5.94)	2.72 (1.54, 4.50)	-0.11 (-0.41, 0.38)
**San Marino**	0.884	6.70 (3.71, 12.88)	8.62 (3.94, 18.51)	0.29 (-0.19, 0.97)	4.82 (2.22, 10.86)	6.23 (2.34, 15.72)	0.29 (-0.18, 0.92)	1.59 (0.93, 2.64)	2.01 (0.96, 4.10)	0.26 (-0.25, 0.98)	0.28 (0.17, 0.43)	0.38 (0.19, 0.70)	0.35 (-0.23, 1.17)
**Sao Tome and Principe**	0.502	18.88 (10.77, 33.93)	29.07 (16.74, 48.92)	0.54 (0.13, 1.14)	8.34 (3.40, 20.87)	13.47 (5.58, 29.35)	0.62 (0.15, 1.30)	10.27 (5.76, 18.91)	15.28 (7.72, 27.06)	0.49 (0.06, 1.09)	0.27 (0.09, 0.61)	0.32 (0.13, 0.67)	0.18 (-0.21, 0.90)
**Saudi Arabia**	0.805	36.50 (21.17, 62.69)	30.39 (18.73, 50.81)	-0.17 (-0.43, 0.22)	22.73 (10.88, 47.94)	20.77 (10.62, 40.52)	-0.09 (-0.39, 0.40)	13.05 (7.39, 20.66)	9.22 (5.64, 13.93)	-0.29 (-0.54, 0.08)	0.72 (0.37, 1.25)	0.40 (0.23, 0.63)	-0.44 (-0.72, 0.05)
**Senegal**	0.389	20.05 (12.39, 33.17)	25.70 (15.10, 41.13)	0.28 (-0.05, 0.74)	8.37 (3.74, 18.46)	10.51 (4.57, 22.68)	0.26 (-0.07, 0.72)	11.37 (6.64, 19.22)	14.84 (7.98, 24.73)	0.30 (-0.07, 0.83)	0.30 (0.11, 0.67)	0.36 (0.12, 0.80)	0.17 (-0.22, 0.75)
**Serbia**	0.767	46.15 (25.39, 84.66)	53.65 (28.25, 103.08)	0.16 (-0.21, 0.66)	33.06 (14.84, 71.15)	42.61 (18.60, 92.80)	0.29 (-0.13, 0.84)	11.24 (6.41, 17.88)	8.76 (4.89, 15.13)	-0.22 (-0.47, 0.12)	1.85 (1.07, 2.98)	2.28 (1.28, 3.82)	0.23 (-0.20, 0.88)

**Seychelles**	0.724	24.04 (14.48, 41.80)	21.46 (13.43, 35.39)	-0.11 (-0.31, 0.19)	13.13 (5.91, 30.23)	12.12 (5.80, 25.70)	-0.08 (-0.30, 0.28)	10.22 (6.36, 15.97)	8.85 (5.44, 13.39)	-0.13 (-0.35, 0.16)	0.69 (0.34, 1.22)	0.49 (0.20, 0.90)	-0.29 (-0.54, 0.10)
**Sierra Leone**	0.347	9.20 (5.48, 14.91)	13.48 (7.71, 23.92)	0.47 (0.05, 1.04)	3.73 (1.78, 7.94)	5.49 (2.34, 12.99)	0.47 (0.03, 1.09)	5.32 (2.94, 8.35)	7.81 (4.22, 13.17)	0.47 (0.06, 1.06)	0.14 (0.05, 0.33)	0.18 (0.06, 0.43)	0.29 (-0.17, 1.08)
**Singapore**	0.861	18.00 (11.39, 29.14)	4.62 (2.72, 8.44)	-0.74 (-0.81, -0.67)	11.45 (5.89, 22.38)	2.72 (1.22, 6.29)	-0.76 (-0.83, -0.67)	5.76 (3.79, 8.33)	1.55 (0.92, 2.43)	-0.73 (-0.79, -0.64)	0.79 (0.52, 1.12)	0.35 (0.22, 0.54)	-0.56 (-0.65, -0.41)
**Slovakia**	0.812	17.33 (10.67, 29.00)	12.71 (6.83, 23.79)	-0.27 (-0.48, 0.01)	11.19 (5.90, 22.16)	9.48 (4.45, 20.00)	-0.15 (-0.40, 0.15)	5.31 (3.33, 7.70)	2.73 (1.52, 4.53)	-0.49 (-0.63, -0.29)	0.84 (0.52, 1.23)	0.50 (0.29, 0.86)	-0.40 (-0.58, -0.11)
**Slovenia**	0.840	20.08 (10.46, 41.59)	8.37 (4.35, 16.02)	-0.58 (-0.72, -0.39)	15.91 (7.28, 36.09)	5.90 (2.62, 13.11)	-0.63 (-0.75, -0.45)	3.63 (2.16, 5.80)	2.05 (1.08, 3.60)	-0.44 (-0.64, -0.15)	0.54 (0.33, 0.84)	0.42 (0.24, 0.68)	-0.23 (-0.51, 0.16)
**Solomon Islands**	0.407	46.33 (30.74, 67.71)	97.11 (62.67, 140.78)	1.10 (0.52, 1.88)	9.44 (4.84, 18.06)	22.69 (10.75, 43.82)	1.40 (0.62, 2.34)	33.54 (21.01, 49.90)	68.55 (39.75, 103.87)	1.04 (0.47, 1.84)	3.35 (1.49, 6.43)	5.87 (2.87, 11.92)	0.75 (0.17, 1.60)
**Somalia**	0.081	20.17 (11.84, 34.18)	24.57 (13.31, 41.41)	0.22 (-0.17, 0.88)	6.22 (2.54, 15.32)	8.85 (3.54, 20.35)	0.42 (-0.08, 1.27)	13.22 (7.24, 22.60)	14.70 (7.27, 25.36)	0.11 (-0.26, 0.73)	0.73 (0.16, 2.23)	1.02 (0.19, 3.64)	0.39 (-0.25, 1.26)
**South Africa**	0.678	16.35 (9.58, 30.32)	23.05 (13.61, 40.52)	0.41 (0.21, 0.71)	9.25 (4.08, 22.44)	14.36 (6.58, 31.34)	0.55 (0.29, 0.96)	6.81 (4.04, 10.45)	8.31 (4.77, 12.60)	0.22 (-0.01, 0.50)	0.29 (0.17, 0.48)	0.37 (0.22, 0.59)	0.27 (0.04, 0.56)
**South Sudan**	0.363	16.05 (8.98, 28.31)	16.42 (8.54, 28.39)	0.02 (-0.30, 0.58)	5.75 (2.24, 14.69)	6.82 (2.76, 16.38)	0.19 (-0.21, 0.97)	9.93 (5.26, 17.42)	9.19 (4.45, 15.73)	-0.07 (-0.38, 0.39)	0.37 (0.13, 0.90)	0.40 (0.12, 1.04)	0.08 (-0.35, 0.79)

**Spain**	0.767	21.95 (11.25, 45.92)	8.41 (4.77, 16.11)	-0.62 (-0.69, -0.50)	16.00 (6.56, 39.28)	5.02 (2.06, 12.42)	-0.69 (-0.75, -0.60)	5.43 (2.93, 9.42)	2.76 (1.51, 4.92)	-0.49 (-0.59, -0.35)	0.51 (0.30, 0.84)	0.63 (0.36, 1.12)	0.23 (-0.11, 0.68)
**Sri Lanka**	0.690	30.84 (17.26, 61.40)	22.00 (11.63, 40.64)	-0.29 (-0.46, -0.07)	19.76 (8.01, 49.38)	14.80 (5.93, 33.09)	-0.25 (-0.45, 0.03)	8.15 (4.21, 14.09)	5.39 (2.87, 9.08)	-0.34 (-0.55, -0.05)	2.93 (1.38, 5.83)	1.81 (0.94, 3.25)	-0.38 (-0.64, 0.12)
**Sudan**	0.515	28.97 (15.58, 53.35)	40.30 (21.36, 74.89)	0.39 (-0.03, 1.08)	15.46 (6.40, 37.39)	29.79 (13.13, 64.22)	0.93 (0.35, 2.02)	11.75 (5.90, 20.04)	9.21 (4.85, 15.50)	-0.22 (-0.45, 0.17)	1.76 (0.50, 3.94)	1.30 (0.54, 2.61)	-0.26 (-0.54, 0.56)
**Suriname**	0.636	32.66 (20.21, 52.94)	34.08 (21.83, 52.59)	0.04 (-0.18, 0.38)	15.63 (7.16, 32.64)	15.47 (7.33, 30.95)	-0.01 (-0.24, 0.32)	15.77 (9.12, 24.69)	17.11 (10.16, 25.96)	0.09 (-0.17, 0.49)	1.26 (0.77, 1.87)	1.50 (0.91, 2.27)	0.19 (-0.16, 0.70)
**Sweden**	0.872	8.24 (4.48, 16.20)	7.42 (3.80, 15.72)	-0.10 (-0.31, 0.19)	5.99 (2.75, 13.75)	5.13 (2.04, 13.38)	-0.14 (-0.37, 0.19)	1.76 (1.04, 2.88)	1.80 (0.98, 3.26)	0.02 (-0.23, 0.32)	0.49 (0.30, 0.75)	0.50 (0.29, 0.85)	0.02 (-0.25, 0.40)
**Switzerland**	0.929	9.23 (5.02, 19.22)	4.69 (2.44, 10.31)	-0.49 (-0.62, -0.31)	6.64 (2.95, 16.07)	3.25 (1.30, 8.53)	-0.51 (-0.65, -0.31)	2.08 (1.18, 3.56)	1.04 (0.56, 2.02)	-0.50 (-0.64, -0.30)	0.52 (0.31, 0.86)	0.40 (0.23, 0.70)	-0.23 (-0.41, 0.07)
**Syrian Arab Republic**	0.619	26.24 (15.37, 46.48)	31.12 (17.62, 55.61)	0.19 (-0.20, 0.76)	14.43 (6.45, 33.29)	19.14 (8.43, 42.75)	0.33 (-0.12, 0.99)	11.03 (6.20, 18.05)	11.32 (6.08, 19.21)	0.03 (-0.31, 0.55)	0.79 (0.36, 1.45)	0.65 (0.35, 1.15)	-0.17 (-0.52, 0.58)
**Taiwan (Province of China)**	0.868	18.84 (12.30, 29.55)	8.32 (4.86, 13.80)	-0.56 (-0.68, -0.40)	7.98 (3.97, 15.98)	3.96 (1.73, 8.82)	-0.50 (-0.65, -0.31)	10.40 (6.63, 15.49)	3.79 (2.15, 6.31)	-0.64 (-0.74, -0.51)	0.46 (0.30, 0.66)	0.57 (0.33, 0.94)	0.24 (-0.13, 0.76)
**Tajikistan**	0.539	11.29 (7.26, 16.25)	49.81 (28.72, 77.96)	3.41 (2.17, 4.98)	1.82 (0.89, 3.66)	11.25 (4.79, 24.65)	5.18 (3.14, 8.13)	9.27 (5.51, 13.78)	37.78 (19.45, 63.85)	3.08 (1.89, 4.53)	0.20 (0.08, 0.36)	0.78 (0.35, 1.35)	2.94 (1.59, 5.43)

**Thailand**	0.687	14.12 (9.09, 22.21)	11.63 (6.36, 19.90)	-0.18 (-0.45, 0.18)	4.70 (2.33, 10.08)	5.30 (2.29, 11.95)	0.13 (-0.28, 0.66)	7.81 (4.74, 11.99)	4.89 (2.58, 8.80)	-0.37 (-0.58, -0.10)	1.61 (0.87, 2.74)	1.44 (0.76, 2.50)	-0.10 (-0.45, 0.44)
**Timor-Leste**	0.514	16.66 (9.57, 29.16)	45.89 (25.69, 76.48)	1.75 (0.94, 2.92)	6.97 (2.94, 16.12)	21.48 (8.47, 47.05)	2.08 (1.08, 3.71)	8.94 (4.69, 15.27)	23.13 (11.71, 38.48)	1.59 (0.75, 2.77)	0.75 (0.32, 1.61)	1.28 (0.58, 2.57)	0.70 (0.05, 2.04)
**Togo**	0.417	11.67 (7.45, 18.39)	13.54 (8.50, 21.10)	0.16 (-0.08, 0.50)	4.71 (2.36, 9.38)	5.39 (2.63, 10.33)	0.14 (-0.12, 0.51)	6.78 (4.12, 10.35)	7.96 (4.69, 12.75)	0.17 (-0.08, 0.53)	0.18 (0.07, 0.39)	0.18 (0.07, 0.44)	0.03 (-0.30, 0.50)
**Tokelau**	0.626	39.71 (24.59, 62.98)	37.46 (23.38, 57.11)	-0.06 (-0.30, 0.33)	11.54 (5.08, 25.33)	13.10 (6.36, 26.39)	0.14 (-0.17, 0.61)	24.94 (14.37, 40.28)	22.09 (13.06, 34.01)	-0.11 (-0.35, 0.28)	3.23 (1.53, 6.06)	2.26 (1.29, 3.57)	-0.30 (-0.58, 0.22)
**Tonga**	0.636	21.91 (14.08, 33.39)	23.45 (14.79, 35.73)	0.07 (-0.21, 0.46)	7.56 (3.63, 16.56)	9.29 (4.31, 18.70)	0.23 (-0.10, 0.76)	12.82 (7.43, 20.20)	12.77 (7.54, 20.28)	0.00 (-0.28, 0.41)	1.54 (0.77, 2.93)	1.39 (0.72, 2.59)	-0.09 (-0.38, 0.32)
**Trinidad and Tobago**	0.757	44.69 (27.78, 74.57)	22.31 (13.03, 37.37)	-0.50 (-0.63, -0.35)	26.72 (12.54, 55.51)	13.54 (6.36, 27.68)	-0.49 (-0.62, -0.33)	15.99 (9.57, 23.98)	7.24 (4.20, 11.25)	-0.55 (-0.68, -0.37)	1.98 (1.22, 2.98)	1.54 (0.90, 2.41)	-0.23 (-0.49, 0.12)
**Tunisia**	0.672	24.65 (13.34, 47.81)	25.86 (13.55, 48.42)	0.05 (-0.28, 0.53)	15.93 (6.77, 37.73)	20.71 (9.33, 43.28)	0.30 (-0.09, 0.87)	7.49 (3.88, 12.74)	4.47 (2.44, 7.72)	-0.40 (-0.59, -0.09)	1.24 (0.51, 2.53)	0.68 (0.36, 1.17)	-0.45 (-0.69, 0.09)
**Turkey**	0.748	10.50 (6.25, 19.27)	14.42 (7.75, 26.68)	0.37 (-0.11, 0.99)	6.01 (2.80, 13.86)	9.47 (4.09, 21.79)	0.58 (-0.01, 1.33)	3.71 (2.23, 6.29)	4.21 (2.29, 7.13)	0.13 (-0.27, 0.66)	0.78 (0.35, 1.43)	0.75 (0.43, 1.25)	-0.04 (-0.46, 1.03)
**Turkmenistan**	0.670	13.22 (8.72, 20.29)	31.61 (17.06, 53.88)	1.39 (0.62, 2.39)	6.84 (3.47, 13.42)	17.88 (7.63, 38.50)	1.62 (0.71, 2.87)	5.80 (3.56, 8.64)	12.07 (6.32, 20.64)	1.08 (0.35, 2.04)	0.59 (0.32, 0.96)	1.66 (0.87, 2.76)	1.82 (0.81, 3.26)

**Tuvalu**	0.589	48.13 (30.63, 75.26)	51.77 (33.33, 77.75)	0.08 (-0.23, 0.55)	12.67 (5.84, 27.17)	16.62 (8.16, 34.51)	0.31 (-0.09, 0.98)	31.40 (18.55, 49.26)	31.80 (19.43, 48.74)	0.01 (-0.30, 0.47)	4.06 (1.67, 8.32)	3.35 (1.73, 5.86)	-0.17 (-0.49, 0.45)
**Uganda**	0.404	19.17 (10.83, 33.23)	22.66 (12.93, 36.18)	0.18 (-0.14, 0.67)	7.12 (2.78, 17.69)	9.17 (3.89, 21.12)	0.29 (-0.10, 0.87)	11.51 (5.95, 20.41)	12.93 (6.45, 21.92)	0.12 (-0.20, 0.64)	0.54 (0.13, 1.50)	0.57 (0.17, 1.41)	0.06 (-0.28, 0.85)
**Ukraine**	0.736	19.85 (11.45, 34.63)	16.74 (10.08, 28.44)	-0.16 (-0.31, 0.07)	16.15 (8.48, 30.49)	12.74 (6.68, 23.98)	-0.21 (-0.36, 0.00)	2.94 (1.84, 4.43)	3.25 (2.11, 4.71)	0.10 (-0.15, 0.56)	0.75 (0.41, 1.33)	0.75 (0.46, 1.14)	0.00 (-0.30, 0.51)
**United Arab Emirates**	0.880	56.20 (30.91, 98.96)	27.30 (14.60, 46.66)	-0.51 (-0.65, -0.34)	38.18 (17.05, 77.52)	21.41 (10.43, 39.58)	-0.44 (-0.60, -0.21)	16.50 (8.96, 27.04)	5.34 (2.98, 9.18)	-0.68 (-0.79, -0.51)	1.52 (0.69, 2.79)	0.55 (0.21, 1.03)	-0.64 (-0.79, -0.37)
**United Kingdom**	0.847	15.01 (8.00, 30.56)	10.03 (5.69, 19.23)	-0.33 (-0.44, -0.20)	11.54 (5.19, 26.56)	6.40 (2.65, 15.67)	-0.45 (-0.55, -0.32)	2.59 (1.53, 4.17)	2.61 (1.54, 4.45)	0.01 (-0.14, 0.19)	0.88 (0.56, 1.31)	1.02 (0.65, 1.58)	0.17 (0.00, 0.39)
**United Republic of Tanzania**	0.423	10.09 (5.77, 17.35)	18.02 (9.67, 32.73)	0.79 (0.20, 1.60)	3.71 (1.50, 8.99)	8.92 (3.43, 22.34)	1.40 (0.51, 2.68)	6.10 (3.34, 10.06)	8.70 (4.17, 15.86)	0.43 (-0.06, 1.15)	0.28 (0.09, 0.67)	0.40 (0.12, 0.93)	0.42 (-0.10, 1.53)
**United States of America**	0.859	9.07 (5.59, 15.37)	8.82 (5.46, 14.66)	-0.03 (-0.16, 0.14)	5.93 (2.87, 12.40)	4.75 (2.24, 10.34)	-0.20 (-0.34, -0.03)	2.40 (1.50, 3.55)	3.03 (1.94, 4.52)	0.26 (0.11, 0.45)	0.74 (0.50, 1.02)	1.04 (0.68, 1.53)	0.41 (0.18, 0.72)
**United States Virgin Islands**	0.799	14.52 (8.61, 25.18)	18.07 (11.24, 28.78)	0.24 (-0.06, 0.67)	7.85 (3.56, 17.91)	9.63 (4.47, 20.34)	0.23 (-0.09, 0.69)	5.56 (3.16, 8.86)	7.20 (4.22, 11.16)	0.29 (-0.05, 0.80)	1.11 (0.66, 1.79)	1.25 (0.74, 1.89)	0.13 (-0.22, 0.64)
**Uruguay**	0.697	7.79 (4.47, 13.96)	10.32 (5.89, 18.72)	0.33 (0.02, 0.73)	5.41 (2.68, 10.95)	6.66 (3.03, 14.43)	0.23 (-0.09, 0.62)	1.76 (1.07, 2.72)	2.65 (1.51, 4.25)	0.51 (0.09, 1.08)	0.62 (0.36, 0.98)	1.01 (0.63, 1.52)	0.64 (0.07, 1.39)

**Uzbekistan**	0.631	11.02 (7.14, 17.28)	51.95 (31.27, 84.43)	3.71 (2.59, 5.19)	5.55 (2.86, 10.52)	26.88 (11.90, 57.04)	3.84 (2.23, 5.63)	5.22 (3.08, 8.03)	23.99 (12.77, 40.38)	3.59 (2.35, 5.67)	0.25 (0.13, 0.41)	1.08 (0.59, 1.71)	3.41 (1.87, 6.54)
**Vanuatu**	0.485	39.41 (23.33, 63.31)	60.75 (37.45, 90.11)	0.54 (0.13, 1.18)	12.06 (5.73, 25.48)	19.21 (9.04, 37.50)	0.59 (0.15, 1.33)	25.00 (14.61, 40.14)	38.20 (21.77, 58.31)	0.53 (0.11, 1.23)	2.36 (1.05, 4.47)	3.33 (1.58, 6.22)	0.41 (-0.03, 1.13)
**Venezuela (Bolivarian Republic of)**	0.607	20.62 (12.31, 34.61)	18.12 (10.48, 29.72)	-0.12 (-0.33, 0.17)	9.92 (4.07, 22.21)	8.28 (3.46, 17.77)	-0.17 (-0.37, 0.12)	9.50 (5.15, 15.28)	8.43 (4.65, 14.14)	-0.11 (-0.36, 0.24)	1.19 (0.69, 1.95)	1.40 (0.80, 2.23)	0.17 (-0.22, 0.73)
**Viet Nam**	0.617	25.76 (14.96, 46.41)	46.99 (27.11, 83.07)	0.82 (0.26, 1.62)	11.84 (5.15, 29.24)	25.65 (10.58, 56.78)	1.17 (0.45, 2.33)	12.92 (7.04, 22.05)	20.32 (10.78, 34.74)	0.57 (0.06, 1.29)	0.99 (0.47, 2.01)	1.02 (0.49, 1.97)	0.02 (-0.46, 0.94)
**Yemen**	0.412	20.73 (12.20, 34.88)	32.64 (17.21, 59.14)	0.57 (0.08, 1.32)	10.17 (4.96, 20.70)	23.15 (9.89, 51.64)	1.28 (0.57, 2.24)	9.17 (5.12, 14.74)	8.08 (4.02, 13.51)	-0.12 (-0.42, 0.33)	1.40 (0.43, 3.02)	1.42 (0.54, 3.06)	0.01 (-0.35, 0.75)
**Zambia**	0.505	19.75 (11.12, 34.70)	30.94 (18.17, 49.05)	0.57 (0.10, 1.32)	6.93 (2.64, 18.87)	12.26 (4.92, 30.11)	0.77 (0.15, 1.77)	12.31 (6.36, 22.31)	17.98 (8.80, 29.98)	0.46 (0.00, 1.21)	0.51 (0.16, 1.29)	0.70 (0.27, 1.56)	0.37 (-0.20, 1.56)
**Zimbabwe**	0.476	19.66 (10.41, 40.44)	29.19 (16.88, 53.01)	0.48 (0.10, 1.09)	12.97 (5.37, 32.89)	18.81 (7.82, 42.55)	0.45 (0.06, 1.10)	6.03 (3.51, 10.57)	9.31 (4.98, 16.00)	0.54 (0.08, 1.23)	0.66 (0.33, 1.47)	1.06 (0.50, 2.29)	0.61 (0.11, 1.37)
YLDs attributable to HFPG													
**Afghanistan**	0.343	42.29 (23.24, 70.55)	79.69 (44.37, 131.04)	0.88 (0.52, 1.43)	30.76 (15.23, 56.33)	63.44 (31.96, 112.23)	1.06 (0.63, 1.72)	10.20 (5.57, 16.65)	13.92 (7.63, 22.27)	0.36 (0.06, 0.81)	1.32 (0.68, 2.21)	2.33 (1.19, 3.97)	0.76 (0.34, 1.41)
**Albania**	0.681	21.21 (12.24,	24.57 (14.68,	0.16 (0.02,	13.01 (6.80,	16.08 (8.75,	0.24 (0.08,	6.05 (3.44,	5.85 (3.39,	-0.03 (-0.18,	2.15 (1.16,	2.64 (1.46,	0.23 (0.03,

		33.53)	38.39)	0.33)	22.12)	26.95)	0.45)	9.55)	9.13)	0.14)	3.48)	4.18)	0.48)
**Algeria**	0.652	43.50 (24.75, 72.53)	73.32 (40.36, 121.07)	0.69 (0.33, 1.16)	35.16 (18.59, 63.72)	64.11 (32.85, 110.53)	0.82 (0.43, 1.35)	6.80 (3.94, 10.45)	7.33 (4.13, 11.15)	0.08 (-0.15, 0.37)	1.54 (0.80, 2.61)	1.88 (0.97, 3.12)	0.22 (-0.13, 0.59)
**American Samoa**	0.712	129.19 (76.70, 196.86)	119.92 (74.87, 180.14)	-0.07 (-0.22, 0.10)	77.90 (41.32, 131.53)	72.56 (41.40, 119.44)	-0.07 (-0.24, 0.16)	42.72 (24.05, 66.64)	38.08 (21.41, 60.79)	-0.11 (-0.28, 0.10)	8.56 (4.87, 13.60)	9.28 (5.08, 15.24)	0.08 (-0.15, 0.39)
**Andorra**	0.894	13.28 (7.47, 22.61)	18.52 (10.11, 32.23)	0.39 (0.12, 0.73)	10.28 (5.37, 18.51)	14.37 (7.08, 27.36)	0.40 (0.10, 0.76)	1.43 (0.82, 2.25)	1.86 (1.03, 3.03)	0.30 (0.02, 0.69)	1.57 (0.89, 2.44)	2.29 (1.27, 3.57)	0.46 (0.16, 0.89)
**Angola**	0.470	33.29 (18.79, 56.34)	41.81 (23.00, 70.58)	0.26 (0.01, 0.62)	22.75 (10.87, 44.55)	30.61 (14.52, 57.80)	0.35 (0.07, 0.75)	9.64 (5.36, 15.34)	10.04 (5.44, 16.11)	0.04 (-0.19, 0.33)	0.90 (0.50, 1.49)	1.16 (0.62, 1.95)	0.29 (-0.02, 0.75)
**Antigua and Barbuda**	0.743	26.25 (14.76, 44.27)	29.26 (16.94, 46.79)	0.11 (-0.07, 0.39)	19.76 (10.12, 36.09)	22.06 (11.42, 37.39)	0.12 (-0.08, 0.42)	4.77 (2.68, 7.67)	5.07 (2.84, 8.04)	0.06 (-0.15, 0.34)	1.72 (0.98, 2.74)	2.13 (1.20, 3.36)	0.24 (-0.04, 0.62)
**Argentina**	0.708	19.43 (11.55, 31.65)	22.57 (13.34, 36.66)	0.16 (-0.05, 0.45)	13.35 (7.13, 23.01)	16.73 (8.48, 28.97)	0.25 (0.01, 0.60)	4.40 (2.49, 6.99)	4.07 (2.29, 6.39)	-0.07 (-0.25, 0.18)	1.68 (0.88, 2.81)	1.77 (0.96, 2.83)	0.06 (-0.15, 0.35)
**Armenia**	0.689	30.09 (15.96, 52.48)	43.94 (24.06, 73.79)	0.46 (0.06, 1.07)	22.10 (10.14, 43.11)	32.08 (15.27, 60.91)	0.45 (0.05, 1.15)	5.39 (2.84, 8.85)	7.43 (3.84, 11.90)	0.38 (0.00, 0.91)	2.60 (1.33, 4.56)	4.42 (2.38, 7.12)	0.70 (0.21, 1.46)
**Australia**	0.839	13.16 (7.05, 23.94)	16.33 (8.36, 29.61)	0.24 (-0.01, 0.55)	11.41 (5.66, 21.72)	13.79 (6.62, 26.60)	0.21 (-0.06, 0.53)	0.95 (0.53, 1.50)	1.27 (0.71, 2.07)	0.33 (0.03, 0.72)	0.81 (0.45, 1.31)	1.27 (0.71, 2.07)	0.57 (0.26, 1.00)

**Austria**	0.849	15.31 (8.37, 26.50)	23.50 (12.34, 41.07)	0.53 (0.21, 0.90)	11.94 (5.81, 22.40)	18.22 (8.34, 34.19)	0.53 (0.16, 0.93)	1.82 (1.03, 2.80)	2.33 (1.25, 3.80)	0.28 (-0.01, 0.66)	1.56 (0.84, 2.54)	2.95 (1.61, 4.78)	0.89 (0.44, 1.54)
**Azerbaijan**	0.683	21.51 (12.92, 34.25)	58.35 (32.56, 96.67)	1.71 (1.07, 2.44)	12.52 (6.81, 21.88)	35.87 (16.83, 67.17)	1.86 (1.11, 2.71)	7.37 (4.06, 11.51)	18.73 (9.42, 31.56)	1.54 (0.84, 2.40)	1.63 (0.90, 2.66)	3.75 (1.97, 6.27)	1.31 (0.67, 2.15)
**Bahamas**	0.796	23.29 (13.09, 38.89)	28.69 (16.77, 46.83)	0.23 (0.00, 0.55)	17.80 (9.00, 32.47)	21.73 (11.11, 37.53)	0.22 (-0.02, 0.55)	4.23 (2.38, 6.76)	5.23 (2.84, 8.21)	0.23 (-0.03, 0.58)	1.25 (0.72, 2.00)	1.74 (0.98, 2.72)	0.40 (0.09, 0.81)
**Bahrain**	0.751	70.22 (37.97, 117.52)	52.28 (30.52, 81.95)	-0.26 (-0.38, -0.09)	58.91 (28.80, 104.14)	43.43 (22.75, 72.63)	-0.26 (-0.39, -0.08)	9.82 (5.35, 15.35)	7.00 (3.89, 10.69)	-0.29 (-0.43, -0.11)	1.49 (0.79, 2.45)	1.85 (0.93, 3.22)	0.24 (-0.07, 0.68)
**Bangladesh**	0.483	14.95 (8.96, 23.63)	23.61 (14.00, 36.99)	0.58 (0.32, 0.92)	7.65 (4.18, 13.58)	13.26 (6.78, 24.05)	0.73 (0.42, 1.15)	6.26 (3.46, 10.11)	8.85 (5.04, 14.10)	0.41 (0.19, 0.77)	1.04 (0.53, 1.77)	1.50 (0.79, 2.51)	0.45 (0.18, 0.80)
**Barbados**	0.742	24.25 (13.74, 39.14)	26.09 (15.11, 40.94)	0.08 (-0.15, 0.35)	19.41 (10.17, 33.88)	20.56 (10.85, 34.35)	0.06 (-0.17, 0.36)	3.34 (1.98, 5.08)	3.75 (2.12, 5.66)	0.12 (-0.11, 0.41)	1.50 (0.87, 2.37)	1.79 (1.04, 2.77)	0.19 (-0.08, 0.57)
**Belarus**	0.745	28.96 (16.70, 45.57)	31.51 (18.36, 49.53)	0.09 (-0.06, 0.28)	21.62 (11.69, 36.17)	23.78 (12.95, 39.36)	0.10 (-0.06, 0.32)	4.24 (2.38, 6.78)	4.27 (2.42, 6.62)	0.01 (-0.14, 0.20)	3.09 (1.69, 4.98)	3.45 (1.89, 5.54)	0.11 (-0.06, 0.34)
**Belgium**	0.851	16.24 (9.11, 28.07)	19.34 (10.50, 34.26)	0.19 (-0.06, 0.49)	13.00 (6.67, 23.79)	15.15 (7.40, 28.59)	0.16 (-0.10, 0.51)	1.81 (1.03, 2.80)	2.10 (1.16, 3.43)	0.16 (-0.09, 0.49)	1.42 (0.81, 2.27)	2.09 (1.17, 3.39)	0.47 (0.16, 0.88)
**Belize**	0.603	12.92 (7.66, 20.89)	21.22 (12.29, 34.38)	0.64 (0.32, 1.10)	9.52 (5.20, 16.20)	16.07 (8.53, 28.21)	0.69 (0.34, 1.18)	2.47 (1.40, 3.81)	3.68 (2.05, 5.81)	0.49 (0.17, 0.98)	0.93 (0.53, 1.50)	1.47 (0.83, 2.41)	0.57 (0.21, 1.09)

**Benin**	0.352	20.56 (11.53, 36.46)	37.39 (20.94, 63.54)	0.82 (0.40, 1.37)	14.26 (6.77, 28.48)	26.30 (12.04, 49.74)	0.84 (0.38, 1.45)	5.69 (3.25, 9.00)	9.98 (5.10, 16.30)	0.75 (0.33, 1.32)	0.61 (0.34, 1.01)	1.10 (0.58, 1.86)	0.80 (0.31, 1.46)
**Bermuda**	0.813	15.86 (9.16, 25.68)	16.92 (9.84, 28.51)	0.07 (-0.09, 0.29)	12.39 (6.69, 21.53)	13.27 (7.00, 23.58)	0.07 (-0.10, 0.32)	2.34 (1.38, 3.61)	2.27 (1.29, 3.52)	-0.03 (-0.21, 0.21)	1.13 (0.65, 1.81)	1.38 (0.78, 2.22)	0.23 (-0.01, 0.52)
**Bhutan**	0.455	13.88 (8.31, 21.70)	20.69 (11.88, 33.96)	0.49 (0.18, 0.90)	8.09 (4.34, 13.64)	13.69 (6.95, 23.95)	0.69 (0.31, 1.17)	4.76 (2.73, 7.35)	5.76 (3.20, 9.31)	0.21 (-0.07, 0.62)	1.03 (0.53, 1.79)	1.23 (0.65, 2.09)	0.19 (-0.04, 0.55)
**Bolivia (Plurinational State of)**	0.566	13.87 (8.44, 22.46)	18.07 (10.51, 28.84)	0.30 (0.07, 0.61)	9.01 (4.82, 16.04)	12.60 (6.11, 22.19)	0.40 (0.12, 0.75)	3.31 (1.92, 5.13)	3.54 (1.97, 5.60)	0.07 (-0.14, 0.36)	1.55 (0.85, 2.51)	1.93 (1.04, 3.12)	0.24 (0.01, 0.59)
**Bosnia and Herzegovina**	0.718	40.78 (21.93, 69.63)	88.49 (48.25, 150.22)	1.17 (0.70, 1.78)	34.72 (17.25, 61.73)	77.43 (38.53, 140.15)	1.23 (0.71, 1.88)	2.68 (1.54, 4.23)	5.24 (2.88, 8.23)	0.96 (0.54, 1.52)	3.37 (1.89, 5.44)	5.81 (3.23, 9.30)	0.72 (0.33, 1.22)
**Botswana**	0.634	37.39 (19.93, 67.26)	59.77 (31.80, 101.75)	0.60 (0.27, 1.11)	29.29 (14.08, 58.36)	48.11 (22.22, 88.28)	0.64 (0.29, 1.20)	7.34 (4.14, 11.55)	10.49 (5.72, 16.41)	0.43 (0.11, 0.89)	0.76 (0.41, 1.25)	1.18 (0.65, 1.91)	0.55 (0.16, 1.17)
**Brazil**	0.640	34.98 (19.76, 56.76)	25.46 (14.48, 41.45)	-0.27 (-0.34, -0.20)	25.44 (12.28, 46.75)	18.30 (8.90, 33.17)	-0.28 (-0.36, -0.20)	7.56 (4.20, 11.68)	5.31 (3.03, 8.29)	-0.30 (-0.38, -0.21)	1.98 (1.11, 3.15)	1.86 (1.06, 2.92)	-0.06 (-0.17, 0.07)
**Brunei Darussalam**	0.823	143.24 (84.58, 229.40)	98.12 (57.27, 155.67)	-0.32 (-0.40, -0.22)	103.19 (50.84, 179.65)	68.51 (33.99, 119.34)	-0.34 (-0.43, -0.23)	27.43 (15.37, 42.16)	18.15 (10.23, 28.40)	-0.34 (-0.45, -0.21)	12.62 (7.04, 19.98)	11.46 (6.37, 18.23)	-0.09 (-0.25, 0.14)
**Bulgaria**	0.764	44.79 (26.03, 74.21)	66.30 (35.63, 117.82)	0.48 (0.14, 0.91)	33.92 (17.13, 59.67)	54.84 (26.61, 105.02)	0.62 (0.22, 1.12)	8.42 (4.78, 12.97)	7.88 (4.22, 12.89)	-0.06 (-0.27, 0.21)	2.46 (1.40, 3.89)	3.57 (1.99, 5.78)	0.45 (0.16, 0.88)

**Burkina Faso**	0.257	15.55 (8.36, 28.34)	27.42 (15.08, 47.40)	0.76 (0.32, 1.38)	10.67 (5.08, 22.18)	18.28 (8.26, 35.96)	0.71 (0.26, 1.43)	4.33 (2.34, 7.16)	8.18 (4.16, 13.44)	0.89 (0.39, 1.57)	0.55 (0.29, 0.96)	0.96 (0.49, 1.63)	0.75 (0.20, 1.48)
**Burundi**	0.284	23.90 (13.41, 41.40)	22.17 (12.53, 39.85)	-0.07 (-0.25, 0.18)	16.47 (8.23, 33.36)	16.62 (8.11, 31.84)	0.01 (-0.21, 0.31)	6.82 (3.87, 10.80)	4.86 (2.63, 7.86)	-0.29 (-0.43, -0.07)	0.61 (0.34, 0.97)	0.69 (0.37, 1.11)	0.13 (-0.11, 0.48)
**Cabo Verde**	0.525	22.40 (13.31, 35.01)	40.17 (23.61, 64.60)	0.79 (0.45, 1.27)	14.14 (7.46, 24.56)	27.60 (14.04, 49.43)	0.95 (0.52, 1.50)	7.32 (4.30, 11.14)	11.07 (6.32, 16.79)	0.51 (0.18, 0.96)	0.94 (0.51, 1.49)	1.49 (0.84, 2.35)	0.58 (0.21, 1.18)
**Cambodia**	0.469	25.26 (14.73, 40.50)	71.62 (39.76, 121.62)	1.84 (1.23, 2.54)	15.94 (8.14, 28.86)	48.08 (21.44, 92.83)	2.02 (1.32, 2.79)	7.67 (4.30, 12.11)	19.47 (10.05, 31.67)	1.54 (0.90, 2.30)	1.65 (0.91, 2.56)	4.07 (2.15, 6.71)	1.47 (0.83, 2.24)
**Cameroon**	0.490	17.22 (10.27, 26.70)	27.27 (16.61, 43.25)	0.58 (0.35, 0.95)	11.24 (5.97, 19.69)	18.23 (9.85, 31.73)	0.62 (0.33, 1.04)	5.46 (3.11, 8.50)	8.25 (4.76, 12.53)	0.51 (0.28, 0.81)	0.51 (0.29, 0.82)	0.79 (0.44, 1.26)	0.53 (0.21, 0.94)
**Canada**	0.873	18.50 (9.69, 34.14)	27.94 (13.88, 52.46)	0.51 (0.13, 0.97)	16.05 (7.63, 31.79)	24.03 (10.59, 48.51)	0.50 (0.10, 0.96)	1.21 (0.69, 1.93)	1.85 (1.00, 3.08)	0.53 (0.15, 1.06)	1.24 (0.69, 2.02)	2.06 (1.09, 3.32)	0.66 (0.24, 1.28)
**Central African Republic**	0.274	27.31 (16.45, 44.33)	41.13 (23.82, 69.11)	0.51 (0.23, 0.90)	17.49 (9.12, 31.78)	27.31 (13.59, 52.14)	0.56 (0.23, 0.99)	8.99 (5.19, 13.92)	12.56 (6.76, 19.93)	0.40 (0.11, 0.77)	0.82 (0.46, 1.32)	1.27 (0.68, 2.04)	0.54 (0.19, 1.05)
**Chad**	0.238	18.20 (10.73, 29.39)	30.34 (17.70, 52.27)	0.67 (0.32, 1.13)	12.01 (6.48, 21.29)	20.63 (10.36, 39.55)	0.72 (0.33, 1.20)	5.57 (3.16, 8.73)	8.82 (4.90, 13.97)	0.58 (0.26, 1.07)	0.62 (0.33, 1.00)	0.89 (0.49, 1.44)	0.44 (0.10, 0.94)
**Chile**	0.759	24.47 (13.45, 42.91)	30.96 (17.19, 51.00)	0.27 (0.00, 0.65)	18.49 (8.99, 34.18)	23.58 (11.39, 42.87)	0.28 (0.00, 0.70)	4.61 (2.58, 7.34)	5.35 (2.91, 8.37)	0.16 (-0.10, 0.51)	1.37 (0.72, 2.28)	2.03 (1.12, 3.24)	0.49 (0.14, 0.96)

**China**	0.686	37.42 (20.99, 62.57)	49.73 (27.58, 80.63)	0.33 (0.14, 0.52)	27.13 (13.80, 50.04)	41.57 (21.15, 71.30)	0.53 (0.33, 0.76)	7.84 (4.29, 12.21)	5.95 (3.31, 9.25)	-0.24 (-0.34, -0.14)	2.45 (1.38, 3.87)	2.21 (1.27, 3.43)	-0.09 (-0.20, 0.02)
**Colombia**	0.633	30.75 (17.04, 51.46)	21.58 (12.09, 35.46)	-0.30 (-0.41, -0.17)	23.58 (11.32, 42.39)	15.91 (7.68, 28.38)	-0.33 (-0.43, -0.19)	5.08 (2.83, 7.97)	3.39 (1.97, 5.28)	-0.33 (-0.46, -0.17)	2.08 (1.16, 3.34)	2.28 (1.27, 3.57)	0.10 (-0.13, 0.41)
**Comoros**	0.455	22.83 (12.59, 41.09)	26.22 (13.04, 49.10)	0.15 (-0.18, 0.63)	17.17 (8.49, 34.24)	20.84 (9.10, 42.67)	0.21 (-0.15, 0.75)	5.06 (2.83, 7.90)	4.63 (2.36, 8.04)	-0.09 (-0.34, 0.32)	0.59 (0.33, 0.97)	0.74 (0.38, 1.28)	0.26 (-0.10, 0.82)
**Congo**	0.568	40.03 (23.57, 67.04)	47.52 (26.65, 79.47)	0.19 (-0.02, 0.49)	27.58 (14.15, 49.98)	35.54 (18.01, 65.14)	0.29 (0.05, 0.61)	11.49 (6.35, 17.70)	10.78 (5.82, 17.05)	-0.06 (-0.24, 0.18)	0.95 (0.52, 1.50)	1.20 (0.68, 1.94)	0.26 (0.00, 0.63)
**Cook Islands**	0.764	89.00 (52.78, 142.14)	112.01 (69.07, 170.04)	0.26 (0.03, 0.53)	59.09 (29.15, 105.34)	71.28 (39.31, 119.75)	0.21 (-0.03, 0.51)	24.05 (13.62, 37.89)	31.80 (17.80, 48.56)	0.32 (0.03, 0.65)	5.86 (3.33, 9.25)	8.93 (5.01, 13.81)	0.52 (0.18, 0.92)
**Costa Rica**	0.680	20.33 (11.67, 34.03)	22.39 (13.06, 35.13)	0.10 (-0.09, 0.35)	15.26 (7.89, 27.59)	16.54 (8.61, 27.74)	0.08 (-0.12, 0.34)	3.40 (1.98, 5.29)	3.71 (2.14, 5.79)	0.09 (-0.13, 0.40)	1.67 (0.93, 2.62)	2.14 (1.21, 3.41)	0.28 (0.00, 0.68)
**Croatia**	0.794	44.16 (24.14, 76.22)	49.71 (26.58, 81.98)	0.13 (-0.11, 0.44)	35.00 (16.80, 65.06)	40.68 (19.99, 70.58)	0.16 (-0.10, 0.51)	5.74 (3.20, 8.82)	4.91 (2.63, 7.89)	-0.15 (-0.33, 0.10)	3.43 (1.87, 5.46)	4.12 (2.36, 6.69)	0.20 (-0.05, 0.57)
**Cuba**	0.668	21.68 (12.72, 35.19)	23.53 (13.51, 38.11)	0.09 (-0.11, 0.35)	16.77 (8.97, 28.93)	18.65 (10.05, 31.95)	0.11 (-0.11, 0.40)	3.49 (2.05, 5.38)	3.28 (1.88, 5.03)	-0.06 (-0.24, 0.17)	1.42 (0.84, 2.25)	1.61 (0.93, 2.59)	0.14 (-0.08, 0.46)
**Cyprus**	0.841	33.66 (18.49, 57.36)	25.65 (14.20, 43.77)	-0.24 (-0.33, -0.12)	25.24 (11.03, 48.58)	18.80 (8.80, 35.17)	-0.26 (-0.35, -0.12)	4.61 (2.45, 7.46)	3.44 (1.94, 5.37)	-0.25 (-0.38, -0.09)	3.81 (2.04, 6.09)	3.41 (1.89, 5.41)	-0.11 (-0.27, 0.10)

**Czechia**	0.828	70.09 (36.70, 124.75)	58.86 (33.41, 98.64)	-0.16 (-0.34, 0.10)	59.99 (28.67, 110.71)	47.72 (24.38, 83.71)	-0.20 (-0.38, 0.06)	5.66 (3.17, 9.16)	5.17 (2.95, 8.19)	-0.09 (-0.29, 0.16)	4.44 (2.54, 7.09)	5.98 (3.32, 9.58)	0.35 (0.04, 0.74)
**Côte d'Ivoire**	0.408	22.35 (13.14, 36.51)	39.25 (22.11, 65.43)	0.76 (0.37, 1.24)	15.33 (8.08, 28.26)	27.77 (12.91, 51.70)	0.81 (0.39, 1.34)	6.49 (3.69, 10.14)	10.54 (5.67, 17.47)	0.63 (0.26, 1.10)	0.53 (0.29, 0.85)	0.93 (0.50, 1.56)	0.75 (0.30, 1.41)
**Democratic People's Republic of Korea**	0.558	40.85 (23.61, 65.55)	53.78 (31.07, 91.27)	0.32 (0.10, 0.65)	29.82 (15.47, 50.35)	40.75 (21.32, 73.66)	0.37 (0.13, 0.77)	9.02 (5.11, 14.24)	10.77 (5.95, 16.80)	0.19 (0.00, 0.43)	2.01 (1.15, 3.26)	2.25 (1.26, 3.55)	0.12 (-0.06, 0.42)
**Democratic Republic of the Congo**	0.382	26.04 (15.67, 41.53)	31.26 (18.01, 51.49)	0.20 (-0.01, 0.48)	17.64 (9.33, 32.06)	22.16 (11.32, 40.38)	0.26 (0.01, 0.59)	7.41 (4.30, 11.35)	7.82 (4.36, 12.10)	0.06 (-0.14, 0.34)	0.99 (0.56, 1.55)	1.28 (0.72, 2.08)	0.30 (0.03, 0.66)
**Denmark**	0.890	14.15 (7.89, 24.28)	17.21 (9.37, 29.66)	0.22 (-0.04, 0.53)	11.41 (5.85, 21.12)	13.75 (6.76, 25.72)	0.21 (-0.06, 0.55)	1.53 (0.86, 2.41)	1.74 (0.98, 2.82)	0.14 (-0.11, 0.48)	1.22 (0.66, 1.96)	1.72 (0.98, 2.74)	0.41 (0.10, 0.88)
**Djibouti**	0.459	24.25 (12.90, 44.17)	31.40 (16.36, 58.70)	0.29 (0.00, 0.70)	18.53 (8.68, 37.33)	25.06 (11.76, 51.55)	0.35 (0.02, 0.82)	5.09 (2.80, 8.06)	5.52 (2.93, 9.05)	0.08 (-0.15, 0.42)	0.63 (0.35, 1.03)	0.82 (0.43, 1.36)	0.29 (-0.02, 0.71)
**Dominica**	0.729	25.54 (14.45, 42.64)	31.41 (18.59, 50.00)	0.23 (0.01, 0.52)	19.71 (9.87, 35.23)	24.17 (12.88, 41.42)	0.23 (0.00, 0.54)	4.23 (2.35, 6.69)	5.14 (2.97, 7.83)	0.22 (-0.02, 0.57)	1.61 (0.93, 2.57)	2.10 (1.21, 3.33)	0.31 (0.02, 0.67)
**Dominican Republic**	0.592	8.93 (5.21, 14.48)	21.07 (11.78, 36.69)	1.36 (0.88, 1.97)	6.66 (3.55, 11.76)	16.22 (8.01, 29.57)	1.44 (0.90, 2.14)	1.67 (0.95, 2.61)	3.60 (1.98, 5.77)	1.16 (0.70, 1.89)	0.61 (0.35, 0.99)	1.24 (0.70, 2.01)	1.05 (0.61, 1.71)
**Ecuador**	0.640	15.58 (8.89, 27.88)	23.44 (13.16, 38.57)	0.50 (0.23, 0.88)	11.19 (5.67, 21.47)	17.13 (8.34, 31.05)	0.53 (0.22, 0.96)	2.89 (1.62, 4.51)	3.96 (2.23, 6.26)	0.37 (0.09, 0.73)	1.50 (0.82, 2.42)	2.36 (1.39, 3.77)	0.57 (0.22, 1.02)

**Egypt**	0.658	24.63 (13.72, 41.28)	77.11 (40.68, 131.35)	2.13 (1.34, 3.12)	20.25 (10.10, 36.31)	68.29 (33.52, 121.06)	2.37 (1.45, 3.47)	3.61 (2.01, 5.71)	7.45 (3.93, 11.81)	1.06 (0.55, 1.71)	0.77 (0.39, 1.29)	1.38 (0.71, 2.30)	0.80 (0.32, 1.49)
**El Salvador**	0.573	13.26 (7.57, 22.13)	22.44 (13.04, 36.06)	0.69 (0.34, 1.18)	9.32 (4.65, 17.27)	16.35 (8.18, 29.32)	0.75 (0.36, 1.31)	2.77 (1.62, 4.36)	4.14 (2.30, 6.47)	0.49 (0.16, 0.96)	1.16 (0.67, 1.86)	1.95 (1.11, 3.15)	0.68 (0.26, 1.22)
**Equatorial Guinea**	0.685	31.16 (18.24, 50.71)	44.93 (25.61, 77.13)	0.44 (0.16, 0.83)	20.59 (10.67, 38.19)	33.72 (16.81, 60.94)	0.64 (0.32, 1.10)	9.61 (5.45, 15.06)	10.02 (5.60, 15.69)	0.04 (-0.17, 0.33)	0.95 (0.53, 1.52)	1.19 (0.67, 1.89)	0.25 (-0.02, 0.60)
**Eritrea**	0.396	19.65 (11.36, 33.66)	24.69 (13.39, 44.89)	0.26 (-0.03, 0.68)	13.36 (6.69, 26.33)	18.35 (8.76, 36.19)	0.37 (0.02, 0.85)	5.65 (3.16, 8.86)	5.53 (3.01, 8.97)	-0.02 (-0.23, 0.30)	0.64 (0.34, 1.04)	0.80 (0.43, 1.32)	0.26 (-0.05, 0.73)
**Estonia**	0.835	31.31 (18.21, 51.02)	23.57 (14.23, 36.54)	-0.25 (-0.36, -0.10)	23.58 (12.43, 40.18)	17.15 (9.33, 28.16)	-0.27 (-0.40, -0.11)	4.27 (2.42, 6.69)	2.93 (1.66, 4.57)	-0.31 (-0.42, -0.18)	3.46 (1.87, 5.67)	3.48 (1.98, 5.50)	0.01 (-0.19, 0.27)
**Eswatini**	0.577	36.16 (20.72, 63.23)	53.39 (29.96, 89.98)	0.48 (0.16, 0.87)	27.47 (13.94, 52.61)	41.65 (20.81, 74.11)	0.52 (0.16, 0.97)	7.83 (4.59, 11.87)	10.53 (5.85, 16.30)	0.34 (0.04, 0.71)	0.86 (0.49, 1.35)	1.21 (0.68, 1.93)	0.40 (0.06, 0.88)
**Ethiopia**	0.343	17.19 (10.10, 27.51)	13.77 (7.82, 23.31)	-0.20 (-0.30, -0.09)	10.44 (5.51, 19.38)	9.99 (5.18, 18.26)	-0.04 (-0.15, 0.08)	6.01 (3.43, 9.56)	3.19 (1.78, 5.07)	-0.47 (-0.53, -0.39)	0.74 (0.41, 1.18)	0.59 (0.32, 0.94)	-0.20 (-0.30, -0.09)
**Fiji**	0.664	110.08 (64.43, 172.40)	111.11 (69.02, 166.58)	0.01 (-0.16, 0.23)	72.83 (36.32, 123.67)	74.51 (41.45, 124.87)	0.02 (-0.17, 0.27)	27.76 (15.46, 43.42)	26.47 (15.00, 41.17)	-0.05 (-0.25, 0.22)	9.49 (5.37, 15.22)	10.13 (5.66, 16.19)	0.07 (-0.16, 0.36)
**Finland**	0.856	28.21 (15.79, 52.87)	32.44 (18.43, 53.49)	0.15 (-0.10, 0.43)	22.95 (11.64, 47.12)	24.77 (12.49, 44.45)	0.08 (-0.17, 0.36)	3.17 (1.85, 4.91)	4.14 (2.39, 6.44)	0.31 (0.04, 0.70)	2.09 (1.19, 3.31)	3.54 (2.03, 5.58)	0.69 (0.34, 1.27)

**France**	0.834	10.10 (5.72, 17.08)	10.49 (6.03, 17.45)	0.04 (-0.10, 0.21)	7.89 (4.07, 14.01)	8.30 (4.28, 14.57)	0.05 (-0.10, 0.24)	1.38 (0.78, 2.18)	1.14 (0.65, 1.78)	-0.17 (-0.31, 0.02)	0.84 (0.46, 1.36)	1.06 (0.60, 1.65)	0.27 (0.04, 0.57)
**Gabon**	0.656	39.05 (22.73, 67.41)	50.21 (28.84, 81.58)	0.29 (0.08, 0.57)	27.89 (14.21, 53.21)	37.57 (18.73, 65.29)	0.35 (0.11, 0.66)	10.20 (5.74, 15.90)	11.28 (6.33, 17.57)	0.11 (-0.10, 0.38)	0.96 (0.53, 1.52)	1.36 (0.75, 2.14)	0.42 (0.11, 0.87)
**Gambia**	0.399	19.14 (11.14, 32.07)	34.83 (19.22, 60.16)	0.82 (0.43, 1.34)	13.40 (7.03, 24.65)	25.06 (11.98, 46.19)	0.87 (0.43, 1.42)	5.21 (2.99, 8.28)	8.86 (4.69, 14.56)	0.70 (0.33, 1.25)	0.53 (0.28, 0.86)	0.91 (0.49, 1.49)	0.71 (0.29, 1.42)
**Georgia**	0.702	27.74 (16.48, 43.83)	66.43 (38.47, 105.99)	1.40 (0.90, 1.99)	15.63 (8.25, 26.68)	39.80 (19.49, 74.97)	1.55 (0.99, 2.23)	9.56 (5.36, 15.05)	19.41 (10.07, 31.23)	1.03 (0.54, 1.62)	2.54 (1.41, 4.14)	7.22 (3.79, 11.53)	1.84 (1.06, 2.73)
**Germany**	0.898	37.87 (19.39, 69.12)	32.67 (17.69, 56.51)	-0.14 (-0.26, 0.07)	31.86 (13.84, 63.66)	26.44 (12.75, 49.73)	-0.17 (-0.29, 0.04)	3.37 (1.79, 5.53)	2.95 (1.62, 4.73)	-0.12 (-0.32, 0.13)	2.65 (1.41, 4.27)	3.28 (1.75, 5.12)	0.24 (-0.04, 0.60)
**Ghana**	0.557	26.79 (14.84, 46.27)	49.55 (27.56, 85.98)	0.85 (0.46, 1.37)	19.87 (9.81, 38.22)	37.70 (18.06, 70.72)	0.90 (0.46, 1.49)	6.24 (3.54, 9.83)	10.74 (6.02, 16.99)	0.72 (0.37, 1.21)	0.68 (0.39, 1.10)	1.11 (0.62, 1.79)	0.63 (0.27, 1.17)
**Greece**	0.794	19.70 (11.12, 33.65)	22.04 (12.27, 37.37)	0.12 (-0.12, 0.40)	15.18 (7.56, 28.54)	16.62 (7.96, 30.92)	0.09 (-0.16, 0.40)	3.32 (1.93, 5.29)	3.50 (1.96, 5.60)	0.05 (-0.17, 0.34)	1.20 (0.68, 1.91)	1.92 (1.11, 3.05)	0.60 (0.26, 1.12)
**Greenland**	0.761	28.22 (14.88, 51.23)	59.86 (28.44, 113.28)	1.12 (0.58, 1.69)	24.46 (11.81, 46.53)	52.01 (22.53, 102.80)	1.13 (0.53, 1.70)	2.59 (1.47, 4.18)	5.01 (2.73, 8.30)	0.94 (0.43, 1.65)	1.18 (0.65, 1.90)	2.84 (1.51, 4.88)	1.42 (0.73, 2.45)
**Grenada**	0.669	33.66 (18.32, 54.41)	37.24 (20.90, 58.88)	0.11 (-0.07, 0.37)	26.75 (12.95, 46.81)	29.36 (15.32, 49.55)	0.10 (-0.09, 0.36)	5.12 (2.69, 8.08)	5.77 (3.27, 9.11)	0.13 (-0.10, 0.44)	1.80 (1.00, 2.89)	2.11 (1.18, 3.33)	0.18 (-0.08, 0.55)

**Guam**	0.813	64.90 (37.43, 106.49)	93.57 (55.77, 145.37)	0.44 (0.18, 0.80)	45.29 (23.28, 83.17)	64.69 (33.56, 109.61)	0.43 (0.15, 0.82)	14.87 (8.53, 23.00)	21.80 (12.35, 33.53)	0.47 (0.19, 0.81)	4.74 (2.74, 7.51)	7.07 (4.06, 10.92)	0.49 (0.19, 0.91)
**Guatemala**	0.526	17.81 (10.06, 30.26)	25.42 (14.59, 39.81)	0.43 (0.15, 0.84)	12.45 (5.94, 23.96)	17.74 (9.08, 31.52)	0.42 (0.13, 0.89)	4.32 (2.40, 7.01)	5.79 (3.20, 9.03)	0.34 (0.05, 0.74)	1.04 (0.57, 1.69)	1.89 (1.05, 3.06)	0.82 (0.40, 1.43)
**Guinea**	0.325	14.71 (8.63, 23.27)	29.14 (16.27, 49.33)	0.98 (0.55, 1.57)	9.66 (5.09, 17.67)	19.92 (9.62, 38.11)	1.06 (0.55, 1.69)	4.50 (2.52, 6.96)	8.32 (4.57, 13.36)	0.85 (0.46, 1.44)	0.55 (0.30, 0.88)	0.89 (0.47, 1.44)	0.62 (0.22, 1.25)
**Guinea-Bissau**	0.355	23.08 (14.03, 37.87)	40.77 (23.32, 68.44)	0.77 (0.40, 1.24)	14.66 (7.73, 26.88)	27.65 (13.51, 51.73)	0.89 (0.44, 1.43)	7.81 (4.39, 12.15)	12.12 (6.46, 19.75)	0.55 (0.20, 1.03)	0.61 (0.35, 0.99)	1.00 (0.53, 1.68)	0.65 (0.25, 1.26)
**Guyana**	0.618	47.32 (27.54, 76.98)	46.47 (27.40, 73.46)	-0.02 (-0.18, 0.22)	34.33 (16.75, 59.84)	34.48 (18.09, 59.06)	0.00 (-0.17, 0.26)	11.09 (5.96, 17.69)	9.76 (5.27, 15.14)	-0.12 (-0.31, 0.14)	1.89 (1.06, 3.05)	2.23 (1.24, 3.49)	0.18 (-0.10, 0.55)
**Haiti**	0.432	30.77 (17.53, 49.17)	41.32 (24.21, 64.48)	0.34 (0.12, 0.67)	20.97 (10.31, 37.07)	28.90 (14.60, 49.97)	0.38 (0.16, 0.74)	7.80 (4.16, 12.88)	9.61 (5.26, 14.93)	0.23 (-0.02, 0.60)	2.00 (1.08, 3.27)	2.81 (1.54, 4.50)	0.40 (0.10, 0.83)
**Honduras**	0.496	19.65 (10.80, 32.91)	24.45 (13.93, 39.01)	0.24 (0.04, 0.53)	15.04 (7.47, 26.93)	18.38 (9.36, 32.12)	0.22 (0.02, 0.53)	3.20 (1.70, 4.96)	4.09 (2.35, 6.22)	0.28 (0.02, 0.63)	1.41 (0.79, 2.26)	1.99 (1.12, 3.21)	0.41 (0.10, 0.79)
**Hungary**	0.791	56.79 (30.76, 97.74)	60.58 (32.44, 103.75)	0.07 (-0.17, 0.39)	47.76 (23.94, 86.84)	52.11 (25.38, 93.64)	0.09 (-0.16, 0.44)	6.37 (3.56, 9.81)	5.04 (2.76, 8.10)	-0.21 (-0.38, 0.00)	2.66 (1.47, 4.35)	3.42 (1.90, 5.49)	0.28 (0.01, 0.71)
**Iceland**	0.869	14.14 (8.08, 23.60)	18.67 (10.40, 32.29)	0.32 (0.06, 0.65)	11.27 (5.95, 19.66)	14.76 (7.44, 28.17)	0.31 (0.03, 0.66)	1.38 (0.79, 2.15)	1.66 (0.92, 2.68)	0.20 (-0.07, 0.55)	1.49 (0.84, 2.37)	2.25 (1.26, 3.70)	0.51 (0.19, 0.96)

**India**	0.566	14.79 (8.89, 23.22)	26.21 (15.36, 41.69)	0.77 (0.58, 0.99)	9.41 (4.95, 16.34)	17.39 (8.92, 30.12)	0.85 (0.65, 1.07)	4.36 (2.42, 6.86)	7.34 (3.96, 11.52)	0.68 (0.48, 0.92)	1.01 (0.53, 1.73)	1.48 (0.77, 2.50)	0.45 (0.28, 0.67)
**Indonesia**	0.660	41.77 (23.97, 68.25)	73.49 (41.39, 127.07)	0.76 (0.49, 1.06)	27.21 (13.51, 49.98)	52.00 (24.57, 102.13)	0.91 (0.62, 1.24)	12.39 (6.84, 19.74)	18.43 (10.12, 29.52)	0.49 (0.28, 0.76)	2.17 (1.23, 3.43)	3.06 (1.68, 4.90)	0.41 (0.21, 0.68)
**Iran (Islamic Republic of)**	0.670	31.67 (17.44, 53.63)	52.62 (27.70, 91.96)	0.66 (0.42, 0.93)	28.05 (14.54, 48.39)	47.08 (23.38, 84.37)	0.68 (0.42, 0.96)	2.77 (1.60, 4.32)	4.23 (2.47, 6.62)	0.53 (0.28, 0.82)	0.85 (0.46, 1.43)	1.32 (0.72, 2.12)	0.55 (0.31, 0.85)
**Iraq**	0.671	65.93 (38.92, 109.78)	89.86 (52.04, 140.81)	0.36 (0.07, 0.74)	54.30 (29.31, 95.09)	75.91 (39.98, 124.68)	0.40 (0.08, 0.82)	9.91 (5.79, 15.35)	12.21 (6.94, 18.39)	0.23 (-0.02, 0.56)	1.72 (0.94, 2.81)	1.75 (0.93, 2.94)	0.01 (-0.31, 0.33)
**Ireland**	0.867	12.03 (6.80, 19.88)	17.92 (9.75, 31.76)	0.49 (0.16, 0.94)	9.11 (4.72, 16.01)	13.68 (6.61, 26.92)	0.50 (0.13, 1.00)	1.41 (0.81, 2.25)	2.03 (1.15, 3.23)	0.44 (0.11, 1.01)	1.51 (0.82, 2.53)	2.22 (1.29, 3.56)	0.47 (0.10, 1.00)
**Israel**	0.803	17.62 (9.85, 30.69)	22.47 (12.25, 38.61)	0.28 (0.02, 0.61)	14.17 (7.20, 25.97)	17.87 (8.44, 33.40)	0.26 (0.00, 0.63)	1.95 (1.10, 3.15)	2.23 (1.22, 3.54)	0.14 (-0.11, 0.49)	1.50 (0.84, 2.41)	2.37 (1.35, 3.75)	0.58 (0.24, 1.08)
**Italy**	0.801	23.43 (12.55, 41.16)	22.24 (12.25, 38.33)	-0.05 (-0.16, 0.08)	19.25 (9.33, 36.57)	17.50 (8.38, 32.76)	-0.09 (-0.20, 0.04)	2.33 (1.33, 3.64)	2.16 (1.25, 3.37)	-0.08 (-0.18, 0.06)	1.85 (1.05, 2.93)	2.58 (1.44, 4.09)	0.40 (0.23, 0.61)
**Jamaica**	0.684	22.35 (12.20, 39.15)	33.78 (18.97, 54.50)	0.51 (0.22, 0.97)	17.22 (8.19, 32.89)	24.61 (12.22, 43.48)	0.43 (0.14, 0.88)	3.57 (1.94, 5.71)	6.66 (3.52, 10.35)	0.86 (0.41, 1.52)	1.55 (0.86, 2.58)	2.51 (1.36, 4.05)	0.62 (0.22, 1.20)
**Japan**	0.870	32.00 (18.70, 52.66)	33.19 (19.33, 53.74)	0.04 (-0.07, 0.16)	22.16 (11.21, 39.24)	21.05 (10.39, 40.20)	-0.05 (-0.16, 0.07)	5.40 (3.11, 8.36)	5.12 (2.95, 8.11)	-0.05 (-0.16, 0.07)	4.44 (2.54, 7.02)	7.02 (3.93, 11.07)	0.58 (0.37, 0.78)

**Jordan**	0.731	87.29 (48.70, 148.81)	79.05 (43.47, 128.69)	-0.09 (-0.28, 0.13)	77.45 (40.43, 135.76)	69.95 (36.20, 116.66)	-0.10 (-0.29, 0.14)	8.26 (4.71, 12.68)	7.10 (4.10, 10.94)	-0.14 (-0.31, 0.09)	1.59 (0.85, 2.63)	2.00 (1.07, 3.22)	0.26 (0.00, 0.57)
**Kazakhstan**	0.723	44.29 (23.81, 80.57)	73.44 (41.31, 120.50)	0.66 (0.20, 1.28)	32.41 (15.93, 64.28)	53.32 (25.75, 97.62)	0.65 (0.15, 1.32)	9.07 (4.89, 14.84)	15.13 (8.02, 24.52)	0.67 (0.22, 1.26)	2.81 (1.47, 4.84)	4.99 (2.75, 8.05)	0.78 (0.26, 1.55)
**Kenya**	0.508	20.10 (11.12, 36.64)	23.74 (13.27, 40.88)	0.18 (0.04, 0.33)	14.87 (7.36, 31.13)	17.71 (8.77, 33.29)	0.19 (0.03, 0.36)	4.68 (2.62, 7.53)	5.33 (2.96, 8.51)	0.14 (0.00, 0.29)	0.55 (0.30, 0.87)	0.69 (0.39, 1.10)	0.26 (0.11, 0.42)
**Kiribati**	0.527	127.58 (76.26, 189.14)	150.09 (93.14, 221.73)	0.18 (-0.07, 0.48)	61.79 (33.06, 104.70)	78.40 (43.31, 131.33)	0.27 (0.01, 0.60)	57.58 (31.95, 90.67)	59.92 (33.23, 93.04)	0.04 (-0.20, 0.34)	8.22 (4.59, 13.25)	11.77 (6.59, 18.92)	0.43 (0.11, 0.79)
**Kuwait**	0.851	55.09 (30.73, 90.54)	54.02 (31.72, 85.73)	-0.02 (-0.19, 0.22)	47.13 (23.82, 80.85)	45.27 (24.62, 76.45)	-0.04 (-0.21, 0.21)	6.20 (3.57, 9.55)	6.49 (3.86, 9.86)	0.05 (-0.17, 0.33)	1.75 (0.93, 2.94)	2.26 (1.20, 3.79)	0.29 (0.01, 0.63)
**Kyrgyzstan**	0.596	23.77 (13.44, 38.32)	22.15 (12.85, 36.82)	-0.07 (-0.22, 0.21)	15.82 (8.14, 27.83)	15.20 (7.87, 27.72)	-0.04 (-0.22, 0.26)	6.01 (3.31, 9.53)	4.67 (2.61, 7.44)	-0.22 (-0.36, -0.02)	1.94 (1.04, 3.15)	2.28 (1.24, 3.75)	0.18 (-0.05, 0.56)
**Lao People's Democratic Republic**	0.490	54.74 (32.41, 92.89)	86.39 (50.77, 143.17)	0.58 (0.26, 1.01)	34.35 (17.67, 65.47)	59.57 (27.52, 106.21)	0.73 (0.33, 1.24)	17.44 (9.89, 27.49)	22.59 (12.43, 35.63)	0.30 (0.01, 0.67)	2.95 (1.67, 4.65)	4.23 (2.33, 6.58)	0.43 (0.12, 0.89)
**Latvia**	0.820	33.10 (19.18, 52.88)	35.32 (20.44, 57.11)	0.07 (-0.07, 0.30)	25.31 (13.55, 42.68)	27.80 (15.13, 46.60)	0.10 (-0.06, 0.35)	4.51 (2.50, 6.93)	3.75 (2.13, 5.72)	-0.17 (-0.30, 0.01)	3.27 (1.80, 5.30)	3.77 (2.12, 6.00)	0.15 (-0.04, 0.44)
**Lebanon**	0.708	39.08 (21.48, 68.79)	67.09 (36.00, 113.20)	0.72 (0.36, 1.26)	33.00 (16.38, 60.74)	59.43 (30.05, 104.86)	0.80 (0.41, 1.39)	4.79 (2.64, 7.63)	5.76 (3.25, 9.06)	0.20 (-0.05, 0.55)	1.29 (0.68, 2.11)	1.91 (1.01, 3.17)	0.48 (0.11, 1.01)

**Lesotho**	0.507	26.19 (13.10, 49.18)	47.74 (27.07, 81.44)	0.82 (0.42, 1.59)	19.63 (8.21, 41.58)	33.89 (15.47, 65.28)	0.73 (0.33, 1.46)	5.86 (2.92, 10.43)	12.63 (6.47, 20.32)	1.16 (0.56, 2.04)	0.71 (0.36, 1.30)	1.23 (0.65, 2.04)	0.73 (0.20, 1.53)
**Liberia**	0.370	25.37 (13.90, 41.66)	36.62 (20.68, 60.99)	0.44 (0.14, 0.91)	17.54 (8.39, 33.27)	25.55 (12.45, 46.91)	0.46 (0.12, 0.96)	7.08 (3.93, 11.59)	9.88 (5.33, 15.54)	0.40 (0.08, 0.83)	0.75 (0.43, 1.23)	1.19 (0.63, 1.87)	0.59 (0.20, 1.19)
**Libya**	0.709	50.10 (26.23, 87.27)	81.36 (44.63, 135.57)	0.62 (0.33, 1.09)	41.81 (19.60, 77.14)	71.09 (35.10, 124.20)	0.70 (0.36, 1.25)	6.86 (3.79, 11.10)	8.02 (4.35, 12.53)	0.17 (-0.08, 0.56)	1.43 (0.77, 2.37)	2.25 (1.11, 3.95)	0.58 (0.10, 1.17)
**Lithuania**	0.843	24.08 (13.64, 39.32)	26.64 (15.32, 42.89)	0.11 (-0.03, 0.28)	18.89 (10.02, 32.00)	20.84 (11.17, 34.35)	0.10 (-0.05, 0.29)	2.65 (1.45, 4.21)	2.83 (1.62, 4.43)	0.07 (-0.09, 0.27)	2.54 (1.38, 4.09)	2.97 (1.66, 4.78)	0.17 (-0.03, 0.38)
**Luxembourg**	0.895	15.83 (8.45, 26.82)	25.38 (14.24, 42.54)	0.60 (0.28, 1.05)	12.68 (6.31, 22.83)	19.43 (9.22, 35.99)	0.53 (0.18, 1.00)	1.88 (1.05, 2.97)	3.01 (1.68, 4.78)	0.61 (0.21, 1.14)	1.27 (0.71, 2.10)	2.93 (1.66, 4.62)	1.31 (0.70, 2.17)
**Madagascar**	0.396	24.53 (14.21, 42.07)	31.44 (17.33, 55.06)	0.28 (0.05, 0.60)	16.82 (8.75, 32.92)	22.81 (11.18, 44.57)	0.36 (0.09, 0.73)	7.02 (3.92, 11.01)	7.77 (4.32, 12.12)	0.11 (-0.08, 0.40)	0.69 (0.38, 1.11)	0.87 (0.48, 1.42)	0.25 (-0.03, 0.61)
**Malawi**	0.384	24.57 (12.69, 46.52)	33.32 (17.36, 57.75)	0.36 (0.00, 0.87)	19.44 (8.69, 41.02)	26.45 (12.14, 50.84)	0.36 (0.00, 0.94)	4.49 (2.40, 7.71)	5.92 (3.13, 9.76)	0.32 (-0.05, 0.77)	0.63 (0.33, 1.09)	0.95 (0.48, 1.60)	0.50 (0.06, 1.18)
**Malaysia**	0.737	64.21 (38.18, 100.31)	75.17 (44.90, 122.63)	0.17 (-0.06, 0.45)	43.18 (22.93, 74.40)	54.47 (28.90, 91.98)	0.26 (0.00, 0.59)	17.34 (10.08, 26.24)	16.68 (9.53, 25.40)	-0.04 (-0.21, 0.20)	3.69 (2.14, 5.68)	4.02 (2.35, 6.24)	0.09 (-0.12, 0.38)
**Maldives**	0.562	45.07 (26.40, 76.55)	47.52 (26.54, 80.29)	0.05 (-0.15, 0.33)	30.24 (15.24, 56.71)	35.03 (16.57, 63.92)	0.16 (-0.08, 0.48)	12.18 (7.05, 19.17)	9.35 (5.22, 14.77)	-0.23 (-0.38, -0.04)	2.64 (1.50, 4.17)	3.14 (1.80, 4.95)	0.19 (-0.03, 0.46)

Mali	0.263	17.15 (10.07, 27.40)	27.62 (15.28, 48.00)	0.61 (0.26, 1.11)	11.08 (5.82, 19.80)	18.85 (9.17, 36.23)	0.70 (0.29, 1.24)	5.48 (3.08, 8.58)	7.87 (4.24, 12.84)	0.44 (0.12, 0.89)	0.59 (0.32, 0.96)	0.90 (0.50, 1.46)	0.52 (0.16, 1.09)
Malta	0.801	30.81 (15.75, 53.27)	26.05 (14.24, 44.15)	-0.15 (-0.31, 0.08)	24.29 (10.87, 46.28)	19.52 (9.23, 36.18)	-0.20 (-0.34, 0.05)	3.75 (2.06, 6.11)	3.21 (1.82, 5.02)	-0.14 (-0.33, 0.12)	2.76 (1.52, 4.46)	3.31 (1.86, 5.19)	0.20 (-0.04, 0.54)
Marshall Islands	0.544	147.51 (91.07, 226.19)	155.26 (96.92, 231.36)	0.05 (-0.10, 0.25)	79.92 (41.03, 138.58)	82.90 (45.66, 138.76)	0.04 (-0.15, 0.27)	55.02 (30.32, 84.50)	58.84 (30.85, 94.69)	0.07 (-0.13, 0.32)	12.58 (6.81, 20.03)	13.52 (7.18, 21.91)	0.08 (-0.14, 0.35)
Mauritania	0.496	20.28 (12.13, 32.31)	22.86 (12.97, 39.07)	0.13 (-0.07, 0.43)	14.19 (7.66, 24.43)	16.56 (8.50, 31.42)	0.17 (-0.05, 0.53)	5.52 (3.08, 8.67)	5.61 (3.18, 8.83)	0.02 (-0.17, 0.28)	0.57 (0.31, 0.95)	0.68 (0.38, 1.10)	0.20 (-0.07, 0.54)
Mauritius	0.705	90.35 (50.04, 150.26)	71.58 (44.39, 109.84)	-0.21 (-0.38, 0.03)	69.05 (33.01, 122.97)	50.48 (27.44, 84.70)	-0.27 (-0.44, -0.03)	17.07 (9.44, 25.87)	15.57 (8.95, 23.54)	-0.09 (-0.29, 0.16)	4.23 (2.40, 6.71)	5.53 (3.16, 8.62)	0.31 (0.01, 0.68)
Mexico	0.649	33.59 (19.56, 52.77)	28.92 (16.87, 44.68)	-0.14 (-0.20, -0.05)	26.66 (14.07, 44.99)	22.09 (12.00, 36.52)	-0.17 (-0.24, -0.08)	5.08 (2.98, 7.73)	4.59 (2.67, 7.10)	-0.10 (-0.17, 0.00)	1.86 (1.09, 2.91)	2.24 (1.31, 3.52)	0.20 (0.09, 0.35)
Micronesia (Federated States of)	0.580	75.84 (44.98, 122.43)	125.35 (77.94, 185.12)	0.65 (0.35, 1.08)	42.45 (21.82, 73.72)	69.83 (37.61, 117.36)	0.64 (0.30, 1.13)	27.48 (15.68, 42.51)	45.56 (25.74, 71.90)	0.66 (0.34, 1.08)	5.92 (3.34, 9.49)	9.96 (5.63, 15.81)	0.68 (0.31, 1.18)
Monaco	0.902	14.09 (7.87, 23.88)	21.08 (11.51, 36.47)	0.50 (0.20, 0.93)	11.40 (5.79, 20.56)	17.22 (8.52, 31.30)	0.51 (0.20, 0.97)	1.44 (0.81, 2.26)	2.06 (1.19, 3.32)	0.43 (0.13, 0.86)	1.25 (0.69, 1.98)	1.80 (1.02, 2.86)	0.44 (0.14, 0.87)
Mongolia	0.606	17.23 (9.91, 27.00)	19.63 (11.92, 30.31)	0.14 (-0.03, 0.36)	6.10 (3.17, 10.52)	8.13 (4.32, 13.76)	0.33 (0.14, 0.59)	9.78 (5.19, 15.46)	9.81 (5.49, 15.63)	0.00 (-0.17, 0.22)	1.35 (0.72, 2.25)	1.69 (0.93, 2.72)	0.25 (0.03, 0.53)

**Montenegro**	0.791	41.51 (24.64, 66.84)	67.22 (39.47, 106.49)	0.62 (0.30, 1.10)	24.74 (12.38, 44.97)	42.84 (20.74, 76.52)	0.73 (0.35, 1.28)	14.04 (7.83, 22.29)	20.11 (11.09, 31.54)	0.43 (0.14, 0.83)	2.73 (1.53, 4.33)	4.26 (2.40, 6.94)	0.56 (0.22, 1.02)
**Morocco**	0.548	33.29 (18.46, 57.84)	79.32 (42.88, 136.36)	1.38 (0.87, 2.02)	26.99 (13.74, 49.43)	69.59 (34.52, 125.34)	1.58 (1.02, 2.29)	5.12 (2.89, 8.00)	7.91 (4.32, 12.34)	0.54 (0.23, 0.98)	1.18 (0.62, 1.97)	1.83 (0.95, 3.11)	0.55 (0.18, 1.11)
**Mozambique**	0.307	21.19 (11.89, 38.07)	39.74 (21.95, 72.10)	0.88 (0.46, 1.50)	15.38 (7.48, 31.67)	29.54 (13.90, 57.16)	0.92 (0.43, 1.61)	5.19 (2.90, 8.17)	9.28 (5.00, 14.92)	0.79 (0.40, 1.34)	0.62 (0.34, 1.01)	0.92 (0.49, 1.53)	0.49 (0.15, 1.02)
**Myanmar**	0.521	34.88 (20.07, 58.54)	56.05 (31.68, 91.20)	0.61 (0.30, 1.05)	22.71 (11.42, 41.56)	39.98 (19.06, 72.62)	0.76 (0.38, 1.29)	10.02 (5.52, 15.57)	12.75 (6.88, 20.01)	0.27 (0.01, 0.66)	2.15 (1.23, 3.50)	3.32 (1.87, 5.11)	0.54 (0.23, 1.00)
**Namibia**	0.612	40.78 (21.73, 71.79)	43.98 (23.80, 76.58)	0.08 (-0.14, 0.36)	31.84 (15.20, 62.28)	35.16 (16.11, 65.80)	0.10 (-0.13, 0.43)	8.00 (4.31, 12.85)	7.77 (4.33, 12.73)	-0.03 (-0.24, 0.24)	0.94 (0.50, 1.55)	1.05 (0.56, 1.67)	0.12 (-0.17, 0.48)
**Nauru**	0.618	89.66 (52.78, 144.20)	145.30 (87.76, 224.96)	0.62 (0.32, 1.04)	57.10 (28.62, 99.64)	91.53 (46.71, 156.31)	0.60 (0.26, 1.07)	28.03 (16.07, 43.12)	45.70 (24.89, 71.40)	0.63 (0.29, 1.02)	4.52 (2.50, 6.93)	8.07 (4.36, 13.07)	0.79 (0.40, 1.31)
**Nepal**	0.422	9.81 (5.88, 15.10)	14.86 (8.68, 23.12)	0.51 (0.26, 0.89)	5.74 (3.09, 9.73)	9.37 (4.90, 16.22)	0.63 (0.34, 1.06)	3.27 (1.84, 5.11)	4.34 (2.35, 6.73)	0.33 (0.08, 0.72)	0.79 (0.41, 1.37)	1.15 (0.59, 1.96)	0.45 (0.17, 0.86)
**Netherlands**	0.883	17.78 (9.18, 33.69)	17.78 (9.50, 32.03)	0.00 (-0.23, 0.35)	14.55 (6.79, 29.42)	14.42 (6.88, 27.89)	-0.01 (-0.26, 0.34)	1.73 (0.94, 2.79)	1.77 (1.00, 2.88)	0.02 (-0.23, 0.36)	1.50 (0.81, 2.49)	1.59 (0.89, 2.54)	0.06 (-0.17, 0.42)
**New Zealand**	0.840	11.54 (6.18, 19.87)	13.50 (7.26, 25.08)	0.17 (-0.06, 0.48)	9.37 (4.65, 17.76)	10.84 (5.31, 21.55)	0.16 (-0.09, 0.52)	1.01 (0.57, 1.61)	1.07 (0.61, 1.75)	0.06 (-0.15, 0.34)	1.17 (0.63, 1.93)	1.59 (0.92, 2.59)	0.37 (0.12, 0.69)

**Nicaragua**	0.517	24.40 (13.57, 40.59)	25.31 (14.87, 40.38)	0.04 (-0.12, 0.30)	18.45 (8.88, 34.51)	18.77 (9.40, 32.88)	0.02 (-0.15, 0.30)	4.38 (2.44, 7.07)	4.46 (2.56, 6.99)	0.02 (-0.17, 0.29)	1.57 (0.86, 2.56)	2.08 (1.16, 3.47)	0.33 (0.03, 0.74)
**Niger**	0.162	8.10 (4.74, 13.65)	25.42 (12.93, 45.08)	2.14 (1.26, 3.18)	5.40 (2.79, 10.33)	17.62 (7.76, 36.55)	2.26 (1.22, 3.43)	2.43 (1.34, 3.86)	7.00 (3.29, 12.23)	1.88 (0.86, 3.05)	0.27 (0.14, 0.44)	0.80 (0.40, 1.46)	2.02 (0.97, 3.54)
**Nigeria**	0.515	17.60 (9.69, 30.84)	22.73 (12.32, 40.86)	0.29 (0.13, 0.52)	12.03 (5.77, 24.08)	16.41 (7.70, 32.84)	0.36 (0.19, 0.62)	5.05 (2.81, 8.12)	5.71 (3.14, 9.42)	0.13 (-0.01, 0.33)	0.52 (0.29, 0.85)	0.62 (0.34, 1.01)	0.17 (0.04, 0.37)
**Niue**	0.711	110.27 (64.21, 173.25)	118.30 (72.46, 178.79)	0.07 (-0.12, 0.35)	71.14 (35.24, 122.59)	74.45 (41.47, 126.55)	0.05 (-0.17, 0.35)	31.66 (17.30, 49.67)	35.00 (19.23, 55.52)	0.11 (-0.14, 0.49)	7.47 (4.24, 11.85)	8.85 (4.99, 14.73)	0.18 (-0.11, 0.58)
**North Macedonia**	0.744	62.34 (32.97, 105.59)	97.56 (52.99, 162.41)	0.56 (0.23, 1.05)	50.14 (24.11, 93.59)	79.39 (38.48, 138.80)	0.58 (0.23, 1.12)	7.77 (4.26, 12.41)	10.89 (5.76, 17.21)	0.40 (0.10, 0.80)	4.43 (2.47, 7.17)	7.27 (3.96, 11.41)	0.64 (0.28, 1.18)
**Northern Mariana Islands**	0.771	82.62 (46.94, 134.96)	106.28 (64.44, 165.52)	0.29 (0.04, 0.59)	52.96 (26.41, 96.89)	66.49 (35.91, 115.76)	0.26 (-0.02, 0.62)	24.44 (13.82, 37.96)	32.16 (18.39, 50.04)	0.32 (0.07, 0.67)	5.22 (2.96, 8.22)	7.63 (4.43, 11.72)	0.46 (0.17, 0.89)
**Norway**	0.913	27.37 (15.15, 47.73)	36.84 (20.56, 64.45)	0.35 (0.20, 0.52)	21.82 (10.96, 40.26)	28.94 (14.08, 55.07)	0.33 (0.16, 0.52)	1.96 (1.15, 3.07)	2.78 (1.61, 4.41)	0.42 (0.22, 0.65)	3.59 (2.05, 5.60)	5.11 (2.92, 7.99)	0.43 (0.20, 0.69)
**Oman**	0.783	43.69 (24.62, 71.02)	71.85 (39.08, 118.27)	0.64 (0.30, 1.08)	35.72 (18.59, 60.26)	62.39 (30.58, 106.97)	0.75 (0.38, 1.22)	6.70 (3.75, 10.60)	7.90 (4.43, 12.19)	0.18 (-0.06, 0.51)	1.27 (0.67, 2.10)	1.56 (0.85, 2.49)	0.23 (-0.03, 0.59)
**Pakistan**	0.449	21.51 (13.09, 33.20)	35.70 (21.32, 56.18)	0.66 (0.37, 0.98)	12.60 (6.91, 20.83)	22.90 (12.00, 39.64)	0.82 (0.50, 1.19)	7.63 (4.29, 12.03)	11.12 (6.18, 17.24)	0.46 (0.22, 0.79)	1.28 (0.65, 2.18)	1.68 (0.91, 2.81)	0.31 (0.10, 0.60)

**Palau**	0.738	94.53 (54.56, 153.74)	130.45 (80.36, 200.01)	0.38 (0.11, 0.71)	65.99 (32.80, 118.98)	88.11 (46.50, 148.63)	0.34 (0.06, 0.71)	23.57 (12.86, 36.28)	34.31 (18.72, 51.80)	0.46 (0.12, 0.88)	4.97 (2.84, 7.75)	8.03 (4.47, 12.63)	0.62 (0.25, 1.14)
**Palestine**	0.588	52.90 (27.90, 93.14)	75.42 (39.68, 127.06)	0.43 (0.17, 0.85)	45.98 (21.96, 86.52)	67.04 (33.20, 118.35)	0.46 (0.18, 0.90)	5.68 (3.04, 8.98)	6.52 (3.60, 10.07)	0.15 (-0.10, 0.49)	1.24 (0.68, 2.06)	1.87 (0.96, 3.15)	0.50 (0.13, 0.94)
**Panama**	0.686	23.46 (12.72, 39.90)	26.97 (15.64, 43.70)	0.15 (-0.04, 0.43)	17.61 (8.25, 32.62)	19.67 (9.87, 34.42)	0.12 (-0.07, 0.42)	4.24 (2.31, 6.87)	4.91 (2.77, 7.64)	0.16 (-0.08, 0.47)	1.61 (0.89, 2.61)	2.38 (1.34, 3.72)	0.48 (0.16, 0.90)
**Papua New Guinea**	0.394	63.55 (37.17, 103.33)	112.61 (69.67, 171.93)	0.77 (0.44, 1.20)	32.12 (16.35, 59.89)	56.98 (29.77, 98.56)	0.77 (0.42, 1.21)	26.03 (14.21, 42.08)	45.82 (24.75, 71.70)	0.76 (0.38, 1.22)	5.40 (2.91, 8.75)	9.81 (5.14, 15.78)	0.82 (0.38, 1.34)
**Paraguay**	0.638	17.55 (10.01, 29.71)	27.71 (15.40, 46.39)	0.58 (0.26, 1.06)	11.79 (5.67, 22.16)	19.61 (9.18, 36.36)	0.66 (0.29, 1.20)	4.44 (2.61, 6.94)	6.04 (3.25, 9.56)	0.36 (0.07, 0.80)	1.32 (0.73, 2.15)	2.06 (1.14, 3.29)	0.56 (0.20, 1.15)
**Peru**	0.648	8.98 (5.20, 14.32)	11.74 (6.71, 20.15)	0.31 (0.06, 0.67)	5.93 (3.13, 10.52)	8.31 (4.22, 15.94)	0.40 (0.12, 0.79)	2.08 (1.20, 3.36)	2.20 (1.23, 3.51)	0.06 (-0.15, 0.38)	0.97 (0.54, 1.59)	1.23 (0.69, 1.98)	0.27 (0.02, 0.64)
**Philippines**	0.623	42.24 (23.88, 70.60)	53.90 (30.38, 88.27)	0.28 (0.13, 0.45)	29.53 (14.35, 55.07)	38.11 (18.28, 69.13)	0.29 (0.11, 0.50)	10.54 (5.85, 16.48)	13.03 (7.17, 20.67)	0.24 (0.11, 0.40)	2.17 (1.23, 3.41)	2.76 (1.54, 4.38)	0.27 (0.14, 0.44)
**Poland**	0.802	44.31 (25.37, 70.26)	47.95 (27.31, 77.07)	0.08 (-0.08, 0.27)	35.14 (18.58, 59.02)	39.40 (20.75, 66.10)	0.12 (-0.05, 0.33)	5.15 (2.90, 7.95)	4.52 (2.60, 7.01)	-0.12 (-0.24, 0.02)	4.02 (2.25, 6.30)	4.02 (2.30, 6.28)	0.00 (-0.15, 0.19)
**Portugal**	0.743	40.54 (21.68, 74.54)	30.38 (17.01, 49.84)	-0.25 (-0.43, -0.04)	33.34 (16.16, 65.33)	23.95 (12.08, 41.94)	-0.28 (-0.46, -0.06)	5.21 (3.01, 8.20)	3.95 (2.26, 6.27)	-0.24 (-0.42, -0.04)	1.99 (1.12, 3.16)	2.49 (1.44, 3.94)	0.25 (-0.02, 0.61)

**Puerto Rico**	0.814	23.97 (14.14, 39.22)	24.94 (15.09, 38.49)	0.04 (-0.15, 0.31)	18.40 (9.91, 31.34)	19.45 (11.15, 32.04)	0.06 (-0.15, 0.35)	3.52 (2.03, 5.43)	3.18 (1.84, 4.91)	-0.10 (-0.31, 0.18)	2.05 (1.20, 3.22)	2.31 (1.37, 3.53)	0.13 (-0.15, 0.51)
**Qatar**	0.830	71.69 (40.26, 115.89)	55.99 (32.90, 87.89)	-0.22 (-0.35, -0.05)	58.70 (29.69, 101.74)	45.01 (24.32, 75.05)	-0.23 (-0.38, -0.03)	10.63 (6.00, 16.69)	8.19 (4.60, 12.89)	-0.23 (-0.39, 0.00)	2.36 (1.26, 3.91)	2.79 (1.34, 4.92)	0.18 (-0.23, 0.60)
**Republic of Korea**	0.878	59.11 (34.33, 94.77)	42.65 (22.96, 71.69)	-0.28 (-0.43, -0.09)	42.81 (22.24, 72.20)	32.52 (15.33, 60.77)	-0.24 (-0.40, -0.02)	11.04 (6.33, 16.77)	5.60 (3.13, 9.01)	-0.49 (-0.60, -0.36)	5.26 (2.90, 8.14)	4.54 (2.50, 7.39)	-0.14 (-0.32, 0.15)
**Republic of Moldova**	0.696	27.98 (16.61, 44.65)	30.98 (18.08, 48.62)	0.11 (-0.06, 0.34)	17.75 (9.54, 30.44)	22.45 (11.66, 37.06)	0.26 (0.06, 0.55)	7.67 (4.38, 11.92)	5.38 (3.02, 8.50)	-0.30 (-0.41, -0.14)	2.56 (1.43, 4.14)	3.16 (1.76, 4.98)	0.23 (0.02, 0.48)
**Romania**	0.760	40.02 (22.87, 64.49)	39.09 (22.36, 61.37)	-0.02 (-0.16, 0.17)	31.68 (16.39, 52.81)	31.38 (16.50, 52.62)	-0.01 (-0.17, 0.20)	5.85 (3.31, 9.26)	4.82 (2.77, 7.66)	-0.18 (-0.31, 0.01)	2.49 (1.38, 3.96)	2.88 (1.66, 4.70)	0.16 (-0.04, 0.40)
**Russian Federation**	0.805	50.46 (30.13, 79.14)	47.85 (28.79, 76.18)	-0.05 (-0.20, 0.15)	38.80 (21.39, 64.05)	36.03 (19.72, 60.36)	-0.07 (-0.23, 0.13)	6.88 (3.83, 10.63)	6.55 (3.63, 10.18)	-0.05 (-0.19, 0.15)	4.78 (2.65, 7.72)	5.28 (2.96, 8.46)	0.11 (-0.07, 0.37)
**Rwanda**	0.429	26.39 (13.13, 50.31)	26.90 (13.67, 50.54)	0.02 (-0.26, 0.47)	18.41 (7.74, 40.60)	20.40 (8.65, 43.08)	0.11 (-0.20, 0.62)	7.32 (3.62, 12.85)	5.64 (2.66, 9.86)	-0.23 (-0.48, 0.16)	0.66 (0.33, 1.18)	0.86 (0.42, 1.52)	0.31 (-0.11, 0.98)
**Saint Kitts and Nevis**	0.746	46.38 (25.58, 76.25)	39.07 (22.29, 62.76)	-0.16 (-0.30, 0.01)	36.10 (17.69, 63.36)	30.75 (16.24, 53.11)	-0.15 (-0.29, 0.04)	8.46 (4.48, 13.11)	6.51 (3.55, 9.86)	-0.23 (-0.38, -0.03)	1.82 (1.00, 2.85)	1.81 (1.02, 2.84)	-0.01 (-0.23, 0.28)
**Saint Lucia**	0.670	48.26 (27.50, 76.52)	37.08 (21.83, 57.60)	-0.23 (-0.35, -0.10)	37.65 (19.02, 64.99)	28.50 (15.12, 48.22)	-0.24 (-0.36, -0.09)	8.09 (4.34, 12.64)	6.26 (3.48, 9.64)	-0.23 (-0.36, -0.05)	2.52 (1.42, 4.08)	2.32 (1.37, 3.71)	-0.08 (-0.27, 0.19)

**Saint Vincent and the Grenadines**	0.627	29.28 (16.19, 48.40)	36.95 (21.57, 57.39)	0.26 (0.08, 0.52)	23.78 (11.76, 41.90)	28.29 (14.92, 47.53)	0.19 (0.00, 0.43)	3.87 (2.12, 6.06)	6.64 (3.65, 10.26)	0.72 (0.39, 1.18)	1.63 (0.90, 2.68)	2.01 (1.16, 3.12)	0.23 (-0.02, 0.59)
**Samoa**	0.641	102.60 (60.30, 163.53)	130.06 (79.27, 201.87)	0.27 (0.09, 0.56)	63.19 (30.97, 118.36)	78.38 (40.48, 138.97)	0.24 (0.07, 0.54)	32.01 (17.26, 51.43)	41.89 (22.88, 65.69)	0.31 (0.04, 0.72)	7.40 (4.01, 12.19)	9.79 (5.35, 15.30)	0.32 (0.05, 0.72)
**San Marino**	0.884	14.25 (8.10, 24.68)	21.98 (11.64, 38.13)	0.54 (0.24, 0.93)	11.29 (5.85, 20.83)	17.52 (8.38, 33.24)	0.55 (0.23, 0.95)	1.56 (0.88, 2.52)	2.23 (1.23, 3.58)	0.43 (0.13, 0.87)	1.40 (0.79, 2.26)	2.24 (1.25, 3.62)	0.60 (0.27, 1.06)
**Sao Tome and Principe**	0.502	33.49 (19.37, 57.04)	53.63 (31.18, 91.55)	0.60 (0.32, 1.03)	23.15 (11.89, 43.51)	38.24 (19.38, 68.67)	0.65 (0.33, 1.13)	9.44 (5.41, 14.51)	14.14 (8.04, 22.65)	0.50 (0.23, 0.88)	0.90 (0.51, 1.45)	1.24 (0.70, 2.00)	0.37 (0.08, 0.80)
**Saudi Arabia**	0.805	57.52 (32.96, 95.20)	84.72 (50.49, 127.87)	0.47 (0.14, 0.90)	43.65 (22.37, 79.09)	66.02 (35.44, 107.22)	0.51 (0.13, 1.02)	12.61 (7.13, 19.40)	16.92 (9.70, 25.94)	0.34 (0.05, 0.70)	1.27 (0.69, 2.10)	1.78 (0.93, 2.97)	0.40 (0.08, 0.80)
**Senegal**	0.389	31.53 (18.50, 51.53)	41.51 (24.16, 67.08)	0.32 (0.08, 0.64)	22.36 (11.79, 39.99)	29.57 (15.04, 52.73)	0.32 (0.07, 0.67)	8.29 (4.67, 12.73)	10.69 (5.75, 17.05)	0.29 (0.03, 0.64)	0.89 (0.50, 1.38)	1.25 (0.69, 2.00)	0.41 (0.10, 0.83)
**Serbia**	0.767	60.64 (32.37, 107.67)	82.64 (42.93, 138.30)	0.36 (0.06, 0.76)	49.27 (23.42, 93.98)	69.76 (32.73, 124.57)	0.42 (0.07, 0.85)	6.88 (3.81, 10.93)	7.12 (3.92, 11.33)	0.03 (-0.19, 0.36)	4.49 (2.47, 7.36)	5.76 (3.20, 9.29)	0.28 (0.00, 0.72)
**Seychelles**	0.724	63.90 (37.17, 105.35)	89.50 (53.01, 141.76)	0.40 (0.11, 0.79)	46.44 (24.04, 82.31)	64.68 (33.02, 109.30)	0.39 (0.07, 0.83)	14.54 (8.37, 22.37)	20.02 (11.05, 31.21)	0.38 (0.08, 0.75)	2.93 (1.71, 4.52)	4.80 (2.70, 7.57)	0.64 (0.28, 1.13)
**Sierra Leone**	0.347	14.62 (8.31, 24.09)	22.40 (12.72, 38.52)	0.53 (0.26, 0.96)	10.08 (5.35, 17.83)	15.80 (7.95, 30.00)	0.57 (0.26, 1.05)	4.14 (2.26, 6.59)	5.99 (3.33, 9.68)	0.45 (0.18, 0.79)	0.40 (0.21, 0.66)	0.61 (0.33, 0.98)	0.51 (0.20, 1.00)

**Singapore**	0.861	70.07 (40.95, 112.17)	36.73 (21.67, 59.96)	-0.48 (-0.58, -0.36)	53.15 (28.45, 89.48)	25.74 (13.70, 44.04)	-0.52 (-0.62, -0.38)	11.25 (6.73, 17.14)	6.20 (3.60, 9.50)	-0.45 (-0.56, -0.31)	5.66 (3.34, 8.78)	4.78 (2.80, 7.31)	-0.16 (-0.30, 0.03)
**Slovakia**	0.812	38.96 (22.32, 63.90)	40.00 (22.35, 68.07)	0.03 (-0.15, 0.27)	31.65 (16.86, 53.61)	33.58 (17.31, 60.22)	0.06 (-0.13, 0.33)	4.21 (2.37, 6.65)	3.22 (1.80, 5.10)	-0.24 (-0.38, -0.05)	3.10 (1.75, 4.89)	3.20 (1.83, 5.18)	0.03 (-0.15, 0.25)
**Slovenia**	0.840	37.52 (20.80, 65.79)	26.58 (15.02, 43.36)	-0.29 (-0.43, -0.12)	30.71 (15.19, 58.19)	20.75 (10.57, 36.29)	-0.32 (-0.47, -0.14)	3.52 (2.00, 5.48)	2.55 (1.35, 4.18)	-0.28 (-0.43, -0.08)	3.29 (1.81, 5.23)	3.29 (1.87, 5.10)	0.00 (-0.19, 0.24)
**Solomon Islands**	0.407	59.52 (37.23, 89.72)	115.57 (71.45, 172.73)	0.94 (0.57, 1.37)	28.72 (16.13, 48.11)	59.33 (30.12, 103.59)	1.07 (0.66, 1.57)	26.69 (15.56, 41.77)	48.20 (26.90, 77.73)	0.81 (0.40, 1.26)	4.11 (2.33, 6.60)	8.04 (4.45, 13.11)	0.96 (0.50, 1.51)
**Somalia**	0.081	20.61 (11.69, 37.26)	24.94 (13.96, 43.88)	0.21 (-0.06, 0.61)	14.14 (6.77, 28.29)	18.26 (8.85, 35.45)	0.29 (-0.04, 0.78)	5.83 (3.28, 9.12)	5.81 (3.12, 9.54)	0.00 (-0.22, 0.32)	0.64 (0.35, 1.02)	0.88 (0.48, 1.45)	0.37 (0.03, 0.84)
**South Africa**	0.678	37.75 (20.63, 66.07)	53.58 (29.57, 90.89)	0.42 (0.21, 0.70)	30.23 (15.27, 57.62)	44.27 (21.51, 80.34)	0.46 (0.23, 0.76)	6.70 (3.83, 10.44)	8.13 (4.67, 12.67)	0.21 (0.00, 0.47)	0.81 (0.47, 1.30)	1.18 (0.67, 1.89)	0.45 (0.22, 0.75)
**South Sudan**	0.363	23.35 (12.73, 42.78)	28.65 (15.40, 51.20)	0.23 (-0.04, 0.57)	17.27 (8.10, 36.26)	21.79 (10.22, 41.82)	0.26 (-0.05, 0.66)	5.48 (3.01, 8.47)	6.07 (3.30, 9.93)	0.11 (-0.12, 0.46)	0.60 (0.33, 1.00)	0.79 (0.43, 1.29)	0.31 (0.02, 0.71)
**Spain**	0.767	32.61 (16.79, 60.24)	24.06 (13.48, 41.29)	-0.26 (-0.40, -0.05)	27.03 (12.63, 54.03)	18.99 (9.07, 34.80)	-0.30 (-0.44, -0.09)	3.61 (2.06, 5.84)	2.72 (1.56, 4.27)	-0.25 (-0.41, -0.02)	1.97 (1.10, 3.07)	2.35 (1.34, 3.73)	0.19 (-0.06, 0.52)
**Sri Lanka**	0.690	58.28 (30.49, 104.91)	73.18 (40.36, 118.68)	0.26 (0.07, 0.53)	45.37 (19.62, 88.92)	56.13 (26.69, 100.53)	0.24 (0.04, 0.56)	8.89 (4.60, 14.36)	11.49 (6.24, 17.82)	0.29 (0.06, 0.60)	4.02 (2.18, 6.51)	5.56 (3.19, 8.69)	0.38 (0.10, 0.81)

**Sudan**	0.515	35.82 (20.09, 61.37)	82.74 (45.13, 136.26)	1.31 (0.82, 1.98)	27.78 (13.98, 50.54)	71.02 (35.52, 122.55)	1.56 (0.97, 2.34)	6.85 (3.79, 10.62)	9.82 (5.23, 15.34)	0.43 (0.14, 0.83)	1.20 (0.62, 2.00)	1.89 (1.01, 3.14)	0.58 (0.22, 1.07)
**Suriname**	0.636	30.16 (17.49, 49.46)	40.38 (24.34, 62.68)	0.34 (0.09, 0.68)	22.06 (11.10, 38.32)	29.40 (15.86, 49.49)	0.33 (0.08, 0.70)	6.36 (3.65, 10.08)	8.65 (4.93, 13.26)	0.36 (0.05, 0.75)	1.73 (1.01, 2.80)	2.32 (1.35, 3.67)	0.34 (0.03, 0.75)
**Sweden**	0.872	19.73 (11.00, 34.81)	27.63 (14.97, 47.36)	0.40 (0.15, 0.73)	16.47 (8.60, 30.57)	22.92 (11.69, 43.00)	0.39 (0.11, 0.73)	1.95 (1.14, 3.04)	2.89 (1.64, 4.60)	0.48 (0.16, 0.87)	1.30 (0.74, 2.09)	1.82 (1.02, 2.96)	0.40 (0.11, 0.86)
**Switzerland**	0.929	18.24 (9.99, 31.75)	16.13 (9.12, 29.29)	-0.12 (-0.30, 0.11)	14.41 (7.01, 27.79)	12.33 (6.16, 24.28)	-0.14 (-0.33, 0.10)	1.85 (1.08, 2.87)	1.53 (0.88, 2.41)	-0.17 (-0.34, 0.06)	1.97 (1.10, 3.13)	2.27 (1.33, 3.56)	0.15 (-0.06, 0.42)
**Syrian Arab Republic**	0.619	44.42 (24.66, 76.03)	67.12 (37.66, 112.26)	0.51 (0.20, 0.93)	35.11 (18.22, 62.45)	55.63 (28.27, 97.74)	0.58 (0.25, 1.04)	8.09 (4.55, 12.89)	9.86 (5.28, 15.20)	0.22 (-0.03, 0.57)	1.23 (0.63, 2.04)	1.63 (0.83, 2.73)	0.33 (-0.03, 0.76)
**Taiwan (Province of China)**	0.868	45.19 (26.77, 75.26)	48.90 (27.32, 83.48)	0.08 (-0.11, 0.35)	34.32 (18.16, 57.91)	39.98 (20.04, 73.58)	0.16 (-0.06, 0.45)	9.02 (5.06, 13.81)	6.34 (3.68, 9.94)	-0.30 (-0.43, -0.13)	1.85 (1.06, 2.93)	2.57 (1.43, 4.09)	0.39 (0.12, 0.78)
**Tajikistan**	0.539	13.50 (8.14, 20.79)	39.09 (22.79, 60.84)	1.90 (1.26, 2.73)	5.93 (3.08, 10.23)	19.79 (9.38, 36.60)	2.34 (1.44, 3.38)	6.09 (3.39, 9.64)	15.21 (7.70, 25.12)	1.50 (0.84, 2.31)	1.48 (0.80, 2.47)	4.10 (2.16, 6.89)	1.77 (1.04, 2.68)
**Thailand**	0.687	34.11 (19.71, 53.58)	43.61 (24.61, 73.80)	0.28 (0.02, 0.61)	22.65 (11.83, 40.09)	31.17 (15.34, 58.13)	0.38 (0.08, 0.77)	8.62 (4.77, 13.41)	8.76 (4.96, 13.72)	0.02 (-0.17, 0.29)	2.83 (1.62, 4.34)	3.67 (2.05, 5.81)	0.30 (0.03, 0.68)
**Timor-Leste**	0.514	33.37 (19.52, 57.04)	82.50 (45.12, 143.71)	1.47 (0.92, 2.17)	22.07 (11.03, 43.00)	58.80 (26.18, 112.75)	1.66 (1.01, 2.44)	9.29 (5.14, 14.79)	19.83 (10.28, 33.24)	1.14 (0.60, 1.78)	2.02 (1.12, 3.28)	3.86 (2.09, 6.22)	0.91 (0.44, 1.55)

Togo	0.417	17.41 (10.13, 28.03)	21.39 (12.72, 33.84)	0.23 (0.07, 0.41)	12.09 (6.34, 20.50)	14.91 (7.99, 25.80)	0.23 (0.07, 0.44)	4.86 (2.66, 7.50)	5.86 (3.36, 8.97)	0.21 (0.02, 0.44)	0.47 (0.25, 0.76)	0.62 (0.34, 0.99)	0.32 (0.06, 0.68)
Tokelau	0.626	76.48 (45.28, 125.07)	106.00 (65.37, 164.33)	0.39 (0.14, 0.72)	47.97 (23.44, 85.58)	66.47 (35.43, 114.41)	0.39 (0.12, 0.76)	22.18 (12.65, 34.52)	31.67 (17.99, 49.45)	0.43 (0.15, 0.78)	6.34 (3.54, 10.15)	7.86 (4.37, 12.34)	0.24 (-0.03, 0.60)
Tonga	0.636	76.09 (45.37, 124.14)	89.81 (54.30, 138.73)	0.18 (0.00, 0.39)	49.44 (25.49, 89.81)	59.08 (31.36, 102.14)	0.20 (0.01, 0.43)	20.12 (11.24, 31.98)	22.67 (12.84, 34.65)	0.13 (-0.09, 0.41)	6.53 (3.78, 10.32)	8.06 (4.62, 12.54)	0.23 (-0.01, 0.56)
Trinidad and Tobago	0.757	46.00 (26.54, 73.45)	37.27 (21.63, 58.88)	-0.19 (-0.29, -0.07)	36.46 (18.85, 63.85)	29.59 (16.06, 49.62)	-0.19 (-0.30, -0.07)	7.17 (3.82, 11.23)	5.37 (3.12, 8.49)	-0.25 (-0.38, -0.08)	2.37 (1.33, 3.77)	2.31 (1.28, 3.72)	-0.02 (-0.21, 0.20)
Tunisia	0.672	30.62 (16.16, 54.36)	60.27 (32.43, 102.26)	0.97 (0.59, 1.46)	26.87 (13.35, 49.70)	53.75 (27.28, 94.05)	1.00 (0.60, 1.53)	2.68 (1.46, 4.29)	4.67 (2.49, 7.24)	0.74 (0.35, 1.26)	1.07 (0.57, 1.75)	1.85 (0.98, 3.16)	0.73 (0.29, 1.30)
Turkey	0.748	28.18 (16.55, 46.01)	38.70 (21.62, 65.53)	0.37 (0.10, 0.77)	21.87 (11.53, 37.67)	31.23 (15.75, 56.71)	0.43 (0.14, 0.88)	4.85 (2.76, 7.50)	5.93 (3.49, 9.30)	0.22 (-0.02, 0.60)	1.45 (0.74, 2.47)	1.55 (0.86, 2.52)	0.07 (-0.15, 0.38)
Turkmenistan	0.670	24.04 (14.28, 37.59)	53.85 (29.49, 93.10)	1.24 (0.62, 2.07)	15.55 (8.22, 26.77)	36.81 (17.04, 67.72)	1.37 (0.71, 2.31)	6.50 (3.56, 10.31)	12.97 (6.59, 21.66)	1.00 (0.42, 1.75)	2.00 (1.05, 3.30)	4.06 (2.12, 6.82)	1.03 (0.52, 1.83)
Tuvalu	0.589	75.58 (44.60, 123.64)	112.56 (68.18, 172.11)	0.49 (0.21, 0.89)	45.43 (22.73, 85.15)	68.50 (35.83, 117.78)	0.51 (0.19, 0.98)	23.81 (13.33, 38.26)	35.12 (18.95, 55.61)	0.48 (0.17, 0.88)	6.34 (3.53, 9.98)	8.94 (5.10, 14.32)	0.41 (0.08, 0.84)
Uganda	0.404	26.07 (13.85, 48.84)	32.77 (17.15, 59.24)	0.26 (0.00, 0.63)	20.25 (9.64, 42.14)	26.15 (11.86, 50.44)	0.29 (-0.01, 0.71)	5.13 (2.84, 8.10)	5.70 (3.10, 9.35)	0.11 (-0.13, 0.45)	0.69 (0.38, 1.11)	0.92 (0.52, 1.52)	0.33 (0.03, 0.74)

Ukraine	0.736	32.64 (19.06, 52.60)	33.99 (19.88, 52.92)	0.04 (-0.11, 0.25)	24.93 (12.89, 41.56)	25.14 (13.53, 41.84)	0.01 (-0.16, 0.23)	4.70 (2.57, 7.52)	5.35 (2.97, 8.59)	0.14 (-0.05, 0.36)	3.01 (1.69, 4.80)	3.50 (1.98, 5.73)	0.16 (-0.03, 0.42)
United Arab Emirates	0.880	112.83 (61.98, 182.98)	101.93 (58.44, 161.18)	-0.10 (-0.26, 0.10)	96.05 (47.54, 164.27)	89.62 (49.74, 146.22)	-0.07 (-0.25, 0.17)	14.91 (8.26, 22.96)	10.51 (6.05, 15.70)	-0.29 (-0.44, -0.11)	1.87 (1.02, 3.09)	1.80 (0.96, 3.02)	-0.04 (-0.23, 0.22)
United Kingdom	0.847	22.93 (12.51, 40.15)	26.43 (15.04, 43.56)	0.15 (0.00, 0.35)	18.68 (9.18, 35.16)	20.51 (10.35, 36.26)	0.10 (-0.05, 0.30)	2.17 (1.26, 3.35)	2.96 (1.75, 4.74)	0.36 (0.17, 0.58)	2.08 (1.19, 3.22)	2.97 (1.73, 4.64)	0.43 (0.25, 0.65)
United Republic of Tanzania	0.423	12.60 (6.99, 22.71)	24.87 (13.66, 46.68)	0.97 (0.50, 1.62)	9.38 (4.60, 19.28)	19.86 (9.48, 40.97)	1.12 (0.59, 1.85)	2.80 (1.55, 4.37)	4.32 (2.28, 7.36)	0.54 (0.20, 1.06)	0.41 (0.23, 0.66)	0.70 (0.38, 1.15)	0.68 (0.28, 1.25)
United States of America	0.859	42.33 (24.37, 69.19)	57.80 (33.47, 91.89)	0.37 (0.21, 0.55)	34.63 (18.27, 60.72)	46.42 (24.25, 79.50)	0.34 (0.18, 0.53)	3.43 (2.06, 5.19)	4.85 (2.93, 7.34)	0.41 (0.26, 0.59)	4.27 (2.49, 6.44)	6.52 (3.84, 9.80)	0.53 (0.33, 0.79)
United States Virgin Islands	0.799	17.03 (9.79, 27.83)	26.69 (15.34, 42.18)	0.57 (0.24, 0.97)	12.63 (6.57, 22.40)	19.74 (10.17, 34.07)	0.56 (0.22, 1.01)	3.11 (1.83, 4.94)	5.06 (2.84, 7.99)	0.63 (0.28, 1.10)	1.29 (0.75, 2.03)	1.89 (1.05, 3.00)	0.47 (0.12, 0.96)
Uruguay	0.697	13.99 (7.80, 23.22)	22.76 (12.72, 38.15)	0.63 (0.31, 1.07)	11.06 (5.73, 19.43)	18.06 (9.36, 32.30)	0.63 (0.29, 1.11)	1.90 (1.02, 3.16)	3.08 (1.80, 4.88)	0.62 (0.23, 1.13)	1.02 (0.49, 1.80)	1.62 (0.88, 2.68)	0.59 (0.23, 1.11)
Uzbekistan	0.631	19.65 (11.40, 31.58)	62.00 (35.46, 104.94)	2.15 (1.39, 3.16)	13.41 (6.93, 23.11)	42.21 (20.18, 78.31)	2.15 (1.29, 3.25)	4.99 (2.78, 7.76)	16.31 (8.63, 26.31)	2.27 (1.36, 3.37)	1.26 (0.67, 2.08)	3.49 (1.85, 5.63)	1.78 (1.00, 2.97)
Vanuatu	0.485	84.90 (50.55, 134.36)	137.98 (85.04, 205.48)	0.63 (0.33, 1.02)	49.04 (26.09, 85.24)	82.43 (45.50, 141.10)	0.68 (0.33, 1.15)	30.38 (17.44, 47.39)	46.63 (25.94, 73.30)	0.53 (0.23, 0.93)	5.48 (3.01, 8.84)	8.92 (5.05, 13.91)	0.63 (0.31, 1.06)

Venezuela (Bolivarian Republic of)	0.607	27.05 (15.10, 45.52)	26.99 (15.64, 43.10)	0.00 (-0.15, 0.21)	19.59 (9.03, 35.69)	18.97 (9.64, 33.32)	-0.03 (-0.18, 0.19)	5.75 (3.18, 9.41)	5.83 (3.21, 9.21)	0.01 (-0.18, 0.28)	1.71 (0.96, 2.77)	2.19 (1.23, 3.51)	0.28 (0.00, 0.66)
Viet Nam	0.617	31.78 (17.57, 56.03)	70.04 (38.52, 119.86)	1.20 (0.72, 1.84)	22.15 (10.63, 42.42)	52.36 (24.13, 98.09)	1.36 (0.78, 2.10)	7.60 (4.16, 12.07)	14.48 (7.62, 24.04)	0.91 (0.49, 1.47)	2.03 (1.15, 3.25)	3.21 (1.77, 5.27)	0.58 (0.25, 1.07)
Yemen	0.412	25.89 (14.96, 43.17)	54.06 (27.32, 95.10)	1.09 (0.52, 1.76)	19.57 (10.37, 35.02)	46.52 (22.14, 86.27)	1.38 (0.75, 2.12)	5.38 (3.01, 8.46)	6.23 (3.24, 10.26)	0.16 (-0.13, 0.54)	0.93 (0.48, 1.59)	1.31 (0.69, 2.20)	0.41 (0.06, 0.95)
Zambia	0.505	20.83 (11.48, 38.48)	26.56 (14.30, 46.60)	0.28 (0.01, 0.65)	15.57 (7.30, 32.33)	20.62 (9.54, 39.35)	0.32 (0.03, 0.74)	4.70 (2.63, 7.47)	5.23 (2.82, 8.73)	0.11 (-0.13, 0.46)	0.56 (0.31, 0.90)	0.70 (0.38, 1.17)	0.26 (-0.03, 0.67)
Zimbabwe	0.476	27.28 (13.95, 50.29)	50.21 (26.80, 87.01)	0.84 (0.43, 1.45)	23.09 (10.56, 45.99)	41.91 (20.64, 78.90)	0.82 (0.39, 1.48)	3.32 (1.85, 5.40)	6.64 (3.57, 10.75)	1.00 (0.50, 1.60)	0.87 (0.47, 1.41)	1.66 (0.90, 2.69)	0.91 (0.41, 1.61)
YLLs attributable to HFPG													
Afghanistan	0.343	740.27 (435.01, 1166.14)	1067.05 (635.24, 1721.16)	0.44 (-0.01, 1.08)	264.80 (119.00, 558.21)	581.98 (289.38, 1104.65)	1.20 (0.54, 2.21)	410.72 (224.71, 658.24)	416.94 (223.81, 661.92)	0.02 (-0.30, 0.49)	64.75 (12.97, 145.33)	68.12 (18.58, 140.83)	0.05 (-0.32, 0.81)
Albania	0.681	304.23 (198.73, 447.52)	260.96 (155.32, 413.88)	-0.14 (-0.36, 0.16)	61.85 (32.65, 115.98)	60.92 (29.96, 113.90)	-0.02 (-0.30, 0.35)	231.65 (146.89, 347.35)	190.34 (109.00, 315.07)	-0.18 (-0.39, 0.11)	10.74 (6.71, 16.71)	9.70 (5.29, 16.82)	-0.10 (-0.48, 0.43)
Algeria	0.652	463.39 (286.02, 779.71)	463.42 (262.57, 805.21)	0.00 (-0.30, 0.41)	261.77 (133.30, 552.64)	344.72 (167.12, 672.03)	0.32 (-0.07, 0.89)	175.27 (106.80, 272.10)	101.86 (59.63, 160.27)	-0.42 (-0.60, -0.14)	26.35 (13.18, 42.98)	16.85 (9.32, 26.87)	-0.36 (-0.60, 0.08)
American Samoa	0.712	934.98 (621.58,	670.92 (445.78,	-0.28 (-0.45,	220.74 (114.44,	164.66 (94.93,	-0.25 (-0.44,	655.78 (406.70,	452.83 (280.65,	-0.31 (-0.48,	58.46 (34.78,	53.43 (30.16,	-0.09 (-0.39,

		1279.59)	940.00)	-0.04)	407.72)	288.43)	0.02)	936.42)	653.73)	-0.06)	90.02)	87.66)	0.36)
**Andorra**	0.894	64.77 (38.99, 113.65)	70.12 (38.37, 125.01)	0.08 (-0.29, 0.60)	39.14 (18.97, 80.53)	39.86 (16.02, 89.65)	0.02 (-0.36, 0.48)	17.49 (10.20, 28.93)	20.42 (10.76, 36.19)	0.17 (-0.29, 0.82)	8.14 (4.53, 14.44)	9.84 (5.33, 16.66)	0.21 (-0.25, 0.93)
**Angola**	0.470	494.14 (291.69, 766.88)	517.85 (319.21, 793.04)	0.05 (-0.27, 0.56)	142.30 (61.01, 305.22)	196.99 (87.31, 404.23)	0.38 (-0.06, 1.12)	339.07 (185.05, 550.99)	307.66 (162.21, 490.74)	-0.09 (-0.37, 0.34)	12.77 (4.18, 30.85)	13.21 (5.38, 28.47)	0.03 (-0.37, 1.02)
**Antigua and Barbuda**	0.743	509.33 (324.18, 761.78)	406.00 (266.01, 604.40)	-0.20 (-0.37, 0.04)	203.77 (97.11, 400.20)	168.74 (84.05, 320.68)	-0.17 (-0.35, 0.09)	272.24 (157.49, 421.04)	203.16 (115.89, 303.92)	-0.25 (-0.43, 0.00)	33.32 (19.99, 50.73)	34.10 (20.35, 51.21)	0.02 (-0.26, 0.45)
**Argentina**	0.708	256.42 (174.70, 358.15)	190.52 (124.29, 287.07)	-0.26 (-0.42, -0.05)	89.66 (48.64, 165.05)	75.11 (34.05, 147.21)	-0.16 (-0.39, 0.15)	136.14 (89.48, 192.98)	93.15 (56.78, 141.72)	-0.32 (-0.48, -0.09)	30.62 (18.41, 49.16)	22.25 (13.88, 32.11)	-0.27 (-0.45, 0.01)
**Armenia**	0.689	278.27 (162.81, 490.43)	307.23 (180.48, 525.49)	0.10 (-0.23, 0.60)	167.12 (78.28, 348.27)	190.36 (86.71, 395.54)	0.14 (-0.23, 0.70)	95.63 (53.21, 161.81)	93.98 (51.18, 150.54)	-0.02 (-0.34, 0.49)	15.51 (8.29, 27.10)	22.88 (13.02, 35.15)	0.47 (-0.09, 1.40)
**Australia**	0.839	98.70 (57.41, 180.74)	67.74 (38.16, 123.75)	-0.31 (-0.47, -0.13)	65.91 (31.99, 143.06)	37.94 (15.86, 90.59)	-0.42 (-0.57, -0.26)	24.72 (15.29, 37.45)	21.52 (11.64, 35.22)	-0.13 (-0.33, 0.13)	8.07 (5.06, 12.23)	8.27 (5.04, 12.34)	0.02 (-0.20, 0.36)
**Austria**	0.849	127.58 (74.86, 218.89)	76.01 (44.29, 133.36)	-0.40 (-0.53, -0.25)	87.70 (42.10, 181.75)	43.77 (19.15, 98.45)	-0.50 (-0.63, -0.37)	32.81 (20.63, 48.25)	22.96 (12.68, 36.80)	-0.30 (-0.49, -0.08)	7.07 (4.42, 10.52)	9.28 (5.71, 14.17)	0.31 (0.00, 0.79)
**Azerbaijan**	0.683	232.36 (157.38, 334.08)	750.41 (455.21, 1166.62)	2.23 (1.28, 3.42)	63.16 (35.05, 115.63)	260.02 (117.37, 540.31)	3.12 (1.72, 4.87)	164.29 (101.82, 240.26)	480.56 (260.32, 800.91)	1.93 (1.05, 3.20)	4.91 (2.68, 8.50)	9.83 (4.93, 18.15)	1.00 (0.18, 2.24)

**Bahamas**	0.796	362.07 (240.38, 540.24)	364.60 (234.32, 536.22)	0.01 (-0.26, 0.36)	150.54 (72.74, 298.92)	142.62 (70.89, 267.00)	-0.05 (-0.30, 0.30)	190.28 (114.90, 292.11)	194.90 (114.13, 289.09)	0.02 (-0.25, 0.43)	21.25 (12.91, 32.20)	27.08 (16.08, 41.11)	0.27 (-0.12, 0.83)
**Bahrain**	0.751	548.74 (349.31, 861.84)	237.21 (145.70, 399.76)	-0.57 (-0.68, -0.43)	307.45 (146.10, 588.24)	150.01 (71.94, 291.04)	-0.51 (-0.65, -0.35)	214.28 (122.83, 317.69)	73.65 (44.02, 113.50)	-0.66 (-0.75, -0.52)	27.01 (15.00, 46.11)	13.55 (7.82, 23.60)	-0.50 (-0.70, -0.21)
**Bangladesh**	0.483	401.39 (269.94, 579.28)	454.90 (273.58, 706.21)	0.13 (-0.18, 0.57)	110.69 (54.57, 226.71)	168.09 (74.92, 346.40)	0.52 (0.03, 1.19)	245.66 (150.75, 360.78)	242.78 (136.89, 389.27)	-0.01 (-0.30, 0.40)	45.05 (13.30, 91.75)	44.03 (18.39, 82.92)	-0.02 (-0.36, 1.04)
**Barbados**	0.742	405.87 (260.63, 640.78)	310.58 (196.20, 470.24)	-0.23 (-0.43, 0.02)	226.84 (113.49, 449.32)	160.79 (79.96, 292.75)	-0.29 (-0.48, -0.03)	151.48 (97.75, 219.12)	122.95 (71.97, 182.07)	-0.19 (-0.42, 0.11)	27.55 (17.56, 40.43)	26.84 (16.47, 38.99)	-0.03 (-0.32, 0.39)
**Belarus**	0.745	258.99 (168.29, 395.37)	237.44 (148.25, 378.39)	-0.08 (-0.29, 0.19)	166.23 (90.71, 288.93)	153.03 (81.43, 269.92)	-0.08 (-0.31, 0.22)	76.17 (49.49, 110.78)	67.64 (38.66, 104.95)	-0.11 (-0.36, 0.20)	16.60 (9.38, 26.39)	16.77 (8.86, 27.95)	0.01 (-0.29, 0.46)
**Belgium**	0.851	137.38 (86.98, 230.14)	99.81 (59.53, 172.31)	-0.27 (-0.46, -0.06)	84.70 (42.68, 168.58)	51.80 (22.69, 113.49)	-0.39 (-0.56, -0.17)	44.63 (28.60, 63.86)	37.45 (21.14, 60.98)	-0.16 (-0.38, 0.13)	8.05 (5.05, 12.01)	10.56 (6.58, 15.91)	0.31 (-0.02, 0.80)
**Belize**	0.603	199.69 (135.03, 290.81)	257.70 (166.73, 376.76)	0.29 (-0.01, 0.72)	77.28 (39.97, 143.55)	99.70 (50.05, 187.68)	0.29 (-0.06, 0.80)	108.69 (70.37, 153.12)	139.45 (81.18, 216.64)	0.28 (-0.04, 0.73)	13.72 (8.54, 20.93)	18.55 (11.03, 27.31)	0.35 (0.00, 0.87)
**Benin**	0.352	315.36 (192.90, 498.92)	499.51 (292.40, 784.40)	0.58 (0.13, 1.22)	102.48 (47.71, 223.46)	160.97 (73.04, 336.45)	0.57 (0.06, 1.33)	206.50 (118.87, 335.05)	330.26 (174.47, 535.81)	0.60 (0.12, 1.33)	6.38 (2.19, 14.11)	8.27 (2.86, 19.23)	0.30 (-0.14, 0.99)
**Bermuda**	0.813	195.17 (125.87, 307.53)	116.05 (70.50, 186.10)	-0.41 (-0.56, -0.21)	108.70 (56.62, 221.99)	65.31 (29.66, 127.48)	-0.40 (-0.56, -0.18)	71.32 (45.88, 108.75)	38.90 (22.99, 60.44)	-0.45 (-0.62, -0.23)	15.15 (9.37, 23.74)	11.84 (6.86, 18.20)	-0.22 (-0.46, 0.15)

**Bhutan**	0.455	228.56 (138.43, 351.18)	273.70 (160.11, 443.92)	0.20 (-0.21, 0.82)	66.69 (31.71, 139.99)	117.63 (51.13, 235.05)	0.76 (0.09, 1.68)	135.12 (75.36, 214.62)	133.67 (72.62, 222.13)	-0.01 (-0.36, 0.59)	26.76 (8.15, 55.84)	22.40 (10.66, 40.80)	-0.16 (-0.51, 0.81)
**Bolivia (Plurinational State of)**	0.566	283.15 (183.10, 424.63)	281.99 (164.48, 451.64)	0.00 (-0.30, 0.43)	90.71 (45.21, 171.78)	114.71 (50.33, 224.10)	0.26 (-0.15, 0.84)	147.27 (89.32, 230.23)	122.16 (66.68, 205.42)	-0.17 (-0.45, 0.21)	45.18 (20.57, 80.70)	45.12 (23.95, 73.29)	0.00 (-0.37, 0.69)
**Bosnia and Herzegovina**	0.718	393.49 (234.95, 661.30)	646.31 (364.24, 1152.72)	0.64 (0.16, 1.27)	288.77 (148.54, 544.44)	507.07 (247.94, 988.20)	0.76 (0.23, 1.46)	73.06 (44.47, 110.50)	103.19 (57.55, 163.12)	0.41 (-0.01, 1.00)	31.65 (16.09, 53.65)	36.05 (20.32, 58.38)	0.14 (-0.29, 1.04)
**Botswana**	0.634	525.18 (308.89, 841.00)	700.44 (407.66, 1140.84)	0.33 (-0.09, 1.01)	235.82 (108.68, 505.56)	346.77 (146.66, 674.44)	0.47 (-0.01, 1.30)	278.79 (157.34, 442.75)	342.82 (176.96, 561.09)	0.23 (-0.21, 0.88)	10.56 (5.49, 19.43)	10.85 (4.97, 21.98)	0.03 (-0.44, 0.81)
**Brazil**	0.640	549.84 (358.80, 861.15)	256.27 (167.08, 386.23)	-0.53 (-0.58, -0.48)	285.06 (138.76, 561.89)	131.30 (63.35, 251.29)	-0.54 (-0.59, -0.48)	229.81 (142.79, 327.28)	97.82 (60.12, 143.29)	-0.57 (-0.62, -0.52)	34.97 (20.65, 52.42)	27.14 (16.77, 38.87)	-0.22 (-0.34, -0.08)
**Brunei Darussalam**	0.823	1014.79 (686.04, 1438.02)	428.48 (284.44, 638.76)	-0.58 (-0.66, -0.46)	412.13 (198.75, 755.42)	186.42 (90.95, 355.98)	-0.55 (-0.66, -0.40)	480.09 (287.26, 711.46)	181.08 (107.99, 265.95)	-0.62 (-0.70, -0.50)	122.57 (71.07, 179.87)	60.98 (37.68, 88.04)	-0.50 (-0.63, -0.30)
**Bulgaria**	0.764	602.59 (396.72, 897.58)	717.56 (405.90, 1260.44)	0.19 (-0.17, 0.63)	296.48 (154.08, 569.41)	465.66 (211.14, 956.58)	0.57 (0.11, 1.14)	289.92 (188.39, 418.98)	225.13 (127.08, 363.77)	-0.22 (-0.46, 0.08)	16.19 (10.02, 24.05)	26.77 (15.48, 42.00)	0.65 (0.16, 1.36)
**Burkina Faso**	0.257	210.76 (123.08, 340.51)	391.40 (232.97, 619.32)	0.86 (0.28, 1.73)	59.28 (25.38, 139.69)	98.44 (42.00, 202.73)	0.66 (0.15, 1.46)	146.93 (80.17, 248.39)	286.31 (147.59, 487.32)	0.95 (0.33, 2.01)	4.55 (1.48, 11.08)	6.65 (2.39, 16.02)	0.46 (-0.04, 1.47)
**Burundi**	0.284	428.00 (258.63, 673.36)	359.59 (205.51, 588.86)	-0.16 (-0.43, 0.27)	102.03 (44.11, 231.97)	109.44 (45.08, 230.71)	0.07 (-0.28, 0.64)	313.18 (180.39, 489.02)	237.96 (122.32, 404.84)	-0.24 (-0.49, 0.18)	12.79 (3.90, 32.84)	12.19 (3.37, 38.63)	-0.05 (-0.49, 0.62)

**Cabo Verde**	0.525	168.17 (110.88, 242.22)	357.42 (221.13, 548.95)	1.13 (0.53, 2.08)	40.94 (21.54, 82.95)	127.79 (58.55, 264.73)	2.12 (1.11, 3.54)	123.15 (78.07, 181.90)	224.67 (131.80, 371.76)	0.82 (0.29, 1.72)	4.07 (1.43, 9.23)	4.96 (2.13, 10.50)	0.22 (-0.27, 1.37)
**Cambodia**	0.469	335.53 (221.99, 494.11)	780.71 (487.79, 1183.45)	1.33 (0.60, 2.13)	94.05 (45.01, 190.28)	268.21 (115.22, 557.94)	1.85 (0.85, 2.97)	222.36 (140.38, 340.30)	481.89 (261.64, 778.69)	1.17 (0.47, 1.98)	19.12 (8.72, 33.96)	30.61 (16.33, 52.67)	0.60 (-0.02, 1.76)
**Cameroon**	0.490	244.86 (159.93, 367.12)	343.28 (207.09, 525.92)	0.40 (-0.02, 1.03)	67.81 (32.74, 132.74)	101.59 (49.01, 196.24)	0.50 (0.01, 1.32)	172.15 (107.09, 261.54)	236.08 (135.61, 379.42)	0.37 (-0.05, 0.98)	4.89 (1.91, 10.35)	5.61 (2.15, 12.92)	0.15 (-0.26, 0.95)
**Canada**	0.873	65.12 (39.73, 113.82)	65.75 (37.56, 120.71)	0.01 (-0.26, 0.33)	39.74 (18.85, 86.43)	34.54 (14.09, 83.53)	-0.13 (-0.40, 0.19)	17.72 (10.79, 26.87)	21.65 (11.48, 35.21)	0.22 (-0.11, 0.60)	7.66 (4.86, 11.48)	9.56 (5.63, 14.67)	0.25 (-0.08, 0.69)
**Central African Republic**	0.274	575.55 (359.91, 883.73)	813.62 (471.06, 1242.41)	0.41 (0.01, 1.05)	143.07 (66.70, 287.72)	233.36 (98.03, 494.57)	0.63 (0.13, 1.36)	416.11 (248.36, 638.12)	554.06 (293.07, 904.76)	0.33 (-0.05, 0.96)	16.37 (5.12, 36.66)	26.20 (7.06, 70.37)	0.60 (-0.05, 1.42)
**Chad**	0.238	248.11 (165.45, 368.69)	414.33 (252.09, 641.58)	0.67 (0.23, 1.28)	70.11 (35.88, 139.06)	119.35 (53.78, 244.32)	0.70 (0.19, 1.37)	172.03 (107.33, 256.79)	287.33 (168.63, 458.15)	0.67 (0.21, 1.33)	5.97 (2.09, 12.65)	7.65 (2.78, 16.59)	0.28 (-0.10, 0.92)
**Chile**	0.759	264.95 (164.67, 411.25)	210.49 (136.00, 337.13)	-0.21 (-0.38, 0.03)	129.94 (63.02, 267.07)	109.75 (51.53, 220.03)	-0.16 (-0.35, 0.16)	116.85 (72.15, 173.50)	76.15 (45.21, 113.18)	-0.35 (-0.50, -0.14)	18.16 (11.25, 26.67)	24.59 (14.88, 35.35)	0.35 (0.00, 0.84)
**China**	0.686	539.53 (357.49, 791.13)	336.24 (225.09, 505.46)	-0.38 (-0.51, -0.19)	155.49 (79.83, 291.56)	150.77 (78.44, 283.55)	-0.03 (-0.27, 0.23)	300.38 (185.48, 443.46)	169.83 (107.30, 257.29)	-0.43 (-0.56, -0.29)	83.66 (44.21, 131.73)	15.64 (9.39, 23.83)	-0.81 (-0.88, -0.64)
**Colombia**	0.633	375.29 (245.05, 565.19)	160.39 (97.78, 250.20)	-0.57 (-0.69, -0.41)	179.88 (82.19, 348.47)	63.44 (29.21, 126.31)	-0.65 (-0.74, -0.53)	162.12 (95.73, 244.78)	59.66 (32.94, 95.35)	-0.63 (-0.73, -0.49)	33.29 (20.53, 49.55)	37.29 (21.34, 59.48)	0.12 (-0.27, 0.61)

Comoros	0.455	264.34 (141.72, 428.55)	285.47 (159.33, 495.62)	0.08 (-0.30, 0.78)	87.77 (36.29, 203.12)	115.83 (45.29, 266.10)	0.32 (-0.15, 1.20)	168.29 (89.87, 276.61)	160.69 (77.61, 282.06)	-0.05 (-0.41, 0.70)	8.28 (2.35, 19.65)	8.94 (2.59, 22.20)	0.08 (-0.39, 1.30)
Congo	0.568	603.43 (372.40, 913.45)	571.60 (355.32, 884.20)	-0.05 (-0.31, 0.32)	187.71 (87.95, 388.85)	231.76 (106.42, 474.72)	0.23 (-0.14, 0.74)	402.33 (237.33, 618.05)	326.62 (184.05, 515.64)	-0.19 (-0.42, 0.14)	13.40 (5.61, 32.04)	13.21 (5.57, 29.43)	-0.01 (-0.38, 0.61)
Cook Islands	0.764	554.84 (361.68, 800.10)	393.41 (264.39, 557.28)	-0.29 (-0.48, -0.01)	161.96 (80.67, 304.69)	123.11 (66.78, 227.08)	-0.24 (-0.45, 0.09)	356.10 (217.58, 544.25)	245.90 (150.85, 362.94)	-0.31 (-0.50, -0.01)	36.78 (21.82, 57.26)	24.40 (12.21, 41.77)	-0.34 (-0.59, 0.08)
Costa Rica	0.680	209.12 (132.66, 328.95)	136.86 (84.71, 218.87)	-0.35 (-0.51, -0.12)	101.78 (46.75, 215.66)	63.36 (29.96, 124.26)	-0.38 (-0.55, -0.14)	84.85 (51.79, 127.75)	51.82 (29.87, 82.32)	-0.39 (-0.57, -0.16)	22.49 (13.85, 33.50)	21.67 (12.56, 34.04)	-0.04 (-0.31, 0.35)
Croatia	0.794	481.62 (291.87, 805.58)	335.57 (197.42, 582.67)	-0.30 (-0.49, -0.04)	296.01 (139.87, 604.88)	215.82 (96.40, 445.54)	-0.27 (-0.48, 0.02)	162.47 (100.16, 237.77)	96.99 (53.13, 153.02)	-0.40 (-0.58, -0.15)	23.13 (14.28, 33.98)	22.76 (13.25, 35.37)	-0.02 (-0.30, 0.42)
Cuba	0.668	282.95 (186.84, 428.73)	256.62 (158.11, 406.90)	-0.09 (-0.33, 0.22)	141.05 (72.33, 264.85)	143.64 (68.36, 270.18)	0.02 (-0.25, 0.39)	122.29 (79.17, 177.18)	96.18 (55.09, 145.50)	-0.21 (-0.43, 0.08)	19.61 (11.79, 29.48)	16.80 (10.24, 25.18)	-0.14 (-0.39, 0.25)
Cyprus	0.841	471.50 (274.88, 849.28)	167.93 (97.94, 309.08)	-0.64 (-0.71, -0.57)	276.42 (116.99, 639.51)	96.64 (40.14, 227.32)	-0.65 (-0.73, -0.57)	159.07 (87.17, 250.91)	56.26 (31.19, 90.70)	-0.65 (-0.72, -0.55)	36.01 (18.89, 59.39)	15.03 (9.26, 23.13)	-0.58 (-0.70, -0.38)
Czechia	0.828	700.06 (386.69, 1280.46)	237.33 (143.41, 392.94)	-0.66 (-0.75, -0.54)	535.24 (251.51, 1102.07)	161.47 (79.32, 315.00)	-0.70 (-0.77, -0.59)	137.86 (84.53, 209.06)	54.73 (33.08, 83.14)	-0.60 (-0.71, -0.46)	26.95 (16.85, 39.41)	21.14 (12.54, 32.88)	-0.22 (-0.44, 0.11)
Côte d'Ivoire	0.408	262.06 (169.21, 419.63)	403.74 (238.29, 628.29)	0.54 (0.09, 1.17)	87.70 (43.10, 178.78)	137.17 (61.74, 286.71)	0.56 (0.08, 1.24)	169.78 (102.26, 270.81)	260.57 (144.37, 419.41)	0.53 (0.05, 1.18)	4.58 (1.80, 10.25)	6.00 (2.32, 12.37)	0.31 (-0.16, 1.08)

**Democratic People's Republic of Korea**	0.558	441.89 (288.40, 651.28)	542.94 (360.90, 817.33)	0.23 (-0.07, 0.64)	132.65 (74.75, 237.02)	174.43 (95.69, 322.18)	0.31 (-0.02, 0.81)	280.41 (172.17, 420.25)	343.66 (207.68, 515.14)	0.23 (-0.08, 0.64)	28.83 (15.97, 45.15)	24.85 (12.90, 43.64)	-0.14 (-0.42, 0.29)
**Democratic Republic of the Congo**	0.382	424.44 (271.69, 652.57)	494.27 (300.66, 773.47)	0.16 (-0.18, 0.66)	124.20 (58.43, 271.05)	162.92 (71.42, 338.38)	0.31 (-0.10, 0.89)	287.52 (174.16, 432.66)	314.56 (176.79, 507.83)	0.09 (-0.24, 0.59)	12.72 (4.60, 30.25)	16.80 (5.18, 45.57)	0.32 (-0.23, 1.18)
**Denmark**	0.890	104.90 (65.97, 169.78)	92.09 (54.41, 155.41)	-0.12 (-0.34, 0.18)	58.86 (30.37, 117.76)	51.84 (23.12, 113.13)	-0.12 (-0.38, 0.20)	34.28 (22.00, 49.60)	29.22 (16.21, 48.45)	-0.15 (-0.38, 0.17)	11.76 (7.13, 18.71)	11.03 (6.60, 16.61)	-0.06 (-0.30, 0.35)
**Djibouti**	0.459	278.89 (166.26, 451.34)	344.83 (196.50, 571.05)	0.24 (-0.16, 0.90)	92.98 (38.62, 204.92)	151.42 (66.68, 347.16)	0.63 (0.06, 1.58)	176.97 (98.45, 286.75)	183.21 (94.32, 302.77)	0.04 (-0.33, 0.59)	8.94 (3.28, 17.55)	10.20 (3.22, 22.21)	0.14 (-0.36, 1.03)
**Dominica**	0.729	429.10 (276.74, 643.57)	428.18 (279.64, 640.11)	0.00 (-0.25, 0.33)	195.54 (90.42, 373.92)	200.23 (100.11, 365.16)	0.02 (-0.20, 0.38)	204.78 (118.27, 324.09)	194.01 (113.75, 290.22)	-0.05 (-0.33, 0.34)	28.78 (16.94, 43.82)	33.94 (19.40, 52.77)	0.18 (-0.18, 0.68)
**Dominican Republic**	0.592	138.76 (91.53, 203.96)	340.79 (195.57, 571.22)	1.46 (0.63, 2.59)	50.60 (26.49, 97.54)	146.07 (62.79, 301.55)	1.89 (0.87, 3.24)	79.92 (49.67, 116.91)	175.50 (91.23, 301.33)	1.20 (0.45, 2.29)	8.25 (4.76, 13.19)	19.22 (10.48, 33.28)	1.33 (0.41, 2.79)
**Ecuador**	0.640	194.52 (124.29, 306.39)	197.19 (120.26, 317.19)	0.01 (-0.25, 0.43)	80.70 (39.34, 174.99)	91.37 (42.39, 192.30)	0.13 (-0.19, 0.61)	85.95 (51.84, 126.70)	71.52 (41.22, 113.60)	-0.17 (-0.41, 0.20)	27.88 (15.80, 44.11)	34.31 (20.50, 53.69)	0.23 (-0.19, 0.92)
**Egypt**	0.658	201.96 (126.80, 329.01)	449.63 (244.07, 799.44)	1.23 (0.41, 2.28)	101.65 (48.61, 205.72)	320.66 (145.01, 621.33)	2.15 (0.95, 3.71)	87.69 (53.15, 136.10)	116.55 (58.19, 218.26)	0.33 (-0.18, 1.08)	12.63 (4.86, 23.58)	12.41 (5.99, 22.25)	-0.02 (-0.51, 1.18)
**El Salvador**	0.573	225.93 (147.25, 332.79)	189.46 (114.74, 305.35)	-0.16 (-0.43, 0.27)	72.82 (34.91, 147.14)	74.62 (35.33, 153.42)	0.02 (-0.33, 0.62)	124.63 (77.55, 179.39)	90.64 (49.33, 147.40)	-0.27 (-0.51, 0.12)	28.48 (16.18, 46.08)	24.20 (13.38, 39.91)	-0.15 (-0.51, 0.47)

**Equatorial Guinea**	0.685	526.42 (321.09, 827.89)	408.80 (237.69, 643.31)	-0.22 (-0.52, 0.22)	138.87 (63.55, 291.17)	173.05 (76.43, 352.15)	0.25 (-0.28, 0.96)	372.62 (210.05, 588.10)	227.40 (112.64, 389.19)	-0.39 (-0.64, -0.01)	14.94 (4.81, 35.96)	8.35 (3.85, 16.21)	-0.44 (-0.74, 0.48)
**Eritrea**	0.396	320.08 (194.78, 520.98)	402.43 (238.41, 658.53)	0.26 (-0.16, 0.92)	67.85 (29.49, 154.66)	121.55 (53.08, 249.12)	0.79 (0.08, 2.00)	240.09 (140.83, 378.01)	268.00 (147.04, 441.88)	0.12 (-0.26, 0.68)	12.14 (3.53, 28.37)	12.89 (4.72, 29.05)	0.06 (-0.35, 1.05)
**Estonia**	0.835	339.41 (215.43, 525.20)	97.37 (58.42, 159.77)	-0.71 (-0.78, -0.61)	248.54 (135.99, 435.81)	65.25 (33.35, 120.00)	-0.74 (-0.80, -0.64)	75.02 (49.07, 107.37)	21.92 (12.97, 33.40)	-0.71 (-0.78, -0.60)	15.85 (9.04, 25.15)	10.20 (5.83, 16.87)	-0.36 (-0.56, -0.02)
**Eswatini**	0.577	522.88 (343.80, 786.48)	686.55 (410.19, 1051.89)	0.31 (-0.11, 0.91)	205.21 (103.76, 413.53)	304.30 (138.88, 569.10)	0.48 (0.01, 1.23)	306.10 (193.43, 450.97)	369.23 (201.92, 584.87)	0.21 (-0.21, 0.79)	11.58 (6.15, 21.22)	13.02 (6.87, 23.20)	0.12 (-0.30, 0.84)
**Ethiopia**	0.343	367.67 (230.61, 553.37)	188.59 (116.52, 289.27)	-0.49 (-0.65, -0.27)	58.09 (25.97, 128.91)	51.49 (24.63, 105.58)	-0.11 (-0.44, 0.34)	294.08 (178.82, 447.99)	128.98 (73.16, 194.43)	-0.56 (-0.70, -0.38)	15.50 (4.06, 38.16)	8.13 (2.61, 20.36)	-0.48 (-0.65, 0.21)
**Fiji**	0.664	918.15 (593.28, 1259.57)	684.44 (456.13, 996.78)	-0.25 (-0.48, 0.09)	268.57 (139.59, 490.19)	231.42 (130.14, 406.65)	-0.14 (-0.39, 0.32)	538.70 (324.26, 785.31)	365.47 (221.80, 553.23)	-0.32 (-0.53, 0.03)	110.89 (66.82, 171.45)	87.56 (51.08, 135.17)	-0.21 (-0.48, 0.23)
**Finland**	0.856	236.07 (142.29, 402.03)	149.34 (89.90, 260.74)	-0.37 (-0.50, -0.19)	155.92 (78.76, 319.58)	90.90 (41.38, 194.88)	-0.42 (-0.56, -0.23)	51.12 (33.27, 75.17)	39.45 (23.30, 60.20)	-0.23 (-0.43, 0.03)	29.03 (16.47, 45.61)	18.99 (12.14, 27.20)	-0.35 (-0.51, -0.03)
**France**	0.834	82.50 (52.07, 135.10)	42.25 (27.29, 67.13)	-0.49 (-0.56, -0.38)	46.71 (23.90, 91.89)	21.50 (10.80, 43.62)	-0.54 (-0.61, -0.43)	29.65 (19.13, 43.75)	14.62 (9.33, 21.83)	-0.51 (-0.60, -0.40)	6.14 (3.75, 9.35)	6.13 (3.81, 8.94)	0.00 (-0.17, 0.23)
**Gabon**	0.656	515.45 (327.81, 788.68)	522.84 (324.46, 786.38)	0.01 (-0.28, 0.37)	167.65 (78.75, 336.78)	215.49 (101.24, 412.07)	0.29 (-0.08, 0.78)	337.05 (194.47, 536.17)	295.69 (165.50, 484.31)	-0.12 (-0.39, 0.21)	10.76 (5.03, 24.10)	11.66 (5.81, 23.13)	0.08 (-0.34, 0.82)

**Gambia**	0.399	199.38 (122.81, 307.60)	398.02 (234.43, 613.72)	1.00 (0.35, 1.90)	67.15 (33.91, 132.34)	138.53 (61.86, 283.59)	1.06 (0.41, 1.90)	128.25 (76.68, 200.66)	253.22 (134.98, 401.93)	0.97 (0.29, 1.96)	3.98 (1.41, 8.97)	6.26 (2.28, 14.14)	0.58 (-0.04, 1.50)
**Georgia**	0.702	431.77 (290.18, 622.10)	865.24 (559.15, 1311.16)	1.00 (0.53, 1.59)	149.23 (75.12, 284.54)	337.27 (158.32, 680.55)	1.26 (0.62, 2.04)	258.50 (165.57, 380.82)	440.88 (252.67, 676.22)	0.71 (0.27, 1.25)	24.04 (15.07, 36.14)	87.09 (51.31, 131.82)	2.62 (1.58, 3.94)
**Germany**	0.898	332.32 (184.22, 603.73)	133.41 (82.09, 232.10)	-0.60 (-0.65, -0.51)	231.70 (100.55, 494.68)	81.65 (37.53, 175.10)	-0.65 (-0.70, -0.58)	81.11 (44.57, 128.11)	37.92 (21.40, 57.24)	-0.53 (-0.62, -0.41)	19.51 (11.66, 30.17)	13.83 (8.22, 20.11)	-0.29 (-0.45, -0.05)
**Ghana**	0.557	356.27 (231.39, 567.48)	546.97 (341.20, 854.98)	0.54 (0.10, 1.16)	145.51 (71.44, 309.86)	251.86 (115.90, 500.05)	0.73 (0.23, 1.49)	204.63 (121.97, 323.44)	287.08 (165.75, 457.40)	0.40 (0.01, 1.00)	6.13 (2.48, 13.14)	8.03 (3.76, 15.49)	0.31 (-0.09, 1.06)
**Greece**	0.794	249.72 (156.87, 412.02)	183.51 (111.94, 301.33)	-0.27 (-0.43, -0.07)	141.80 (73.24, 287.43)	91.70 (40.51, 209.59)	-0.35 (-0.53, -0.14)	100.79 (65.02, 149.21)	81.15 (47.38, 128.52)	-0.19 (-0.38, 0.03)	7.13 (4.62, 10.30)	10.67 (6.70, 16.16)	0.50 (0.15, 0.91)
**Greenland**	0.761	205.42 (134.27, 312.11)	307.93 (186.19, 497.11)	0.50 (0.09, 1.02)	90.64 (44.89, 179.05)	129.48 (56.02, 288.42)	0.43 (-0.02, 0.98)	85.24 (53.01, 127.39)	137.76 (72.74, 216.49)	0.62 (0.13, 1.25)	29.54 (17.56, 46.86)	40.69 (22.27, 66.05)	0.38 (-0.09, 1.02)
**Grenada**	0.669	836.24 (537.11, 1261.49)	572.72 (378.76, 844.98)	-0.32 (-0.44, -0.13)	421.88 (202.39, 833.08)	281.39 (142.32, 536.11)	-0.33 (-0.47, -0.13)	368.59 (212.57, 563.86)	250.14 (147.45, 362.36)	-0.32 (-0.48, -0.09)	45.77 (27.18, 71.42)	41.19 (24.42, 61.22)	-0.10 (-0.36, 0.26)
**Guam**	0.813	311.12 (202.31, 463.67)	272.76 (182.39, 387.24)	-0.12 (-0.35, 0.20)	120.17 (60.70, 248.78)	98.22 (53.24, 174.57)	-0.18 (-0.41, 0.15)	165.12 (103.88, 247.91)	152.07 (93.48, 227.37)	-0.08 (-0.33, 0.29)	25.83 (15.76, 39.25)	22.48 (12.76, 34.55)	-0.13 (-0.40, 0.22)
**Guatemala**	0.526	224.30 (140.65, 346.26)	263.15 (160.22, 397.91)	0.17 (-0.14, 0.65)	92.27 (40.74, 191.43)	90.43 (41.99, 180.18)	-0.02 (-0.32, 0.50)	120.41 (71.79, 186.78)	148.99 (83.98, 232.26)	0.24 (-0.13, 0.81)	11.62 (6.41, 19.27)	23.73 (12.87, 37.70)	1.04 (0.29, 2.29)

**Guinea**	0.325	211.01 (138.90, 324.34)	408.35 (247.53, 650.15)	0.94 (0.37, 1.74)	59.40 (30.31, 120.45)	123.29 (54.09, 255.18)	1.08 (0.40, 1.97)	146.04 (89.93, 224.64)	277.37 (154.76, 449.57)	0.90 (0.31, 1.77)	5.57 (1.70, 12.24)	7.69 (2.62, 17.84)	0.38 (-0.10, 1.23)
**Guinea-Bissau**	0.355	354.47 (230.11, 544.84)	568.13 (339.59, 881.68)	0.60 (0.10, 1.37)	91.90 (46.22, 186.40)	171.41 (78.06, 338.43)	0.87 (0.23, 1.83)	255.65 (157.17, 400.82)	387.45 (216.59, 636.76)	0.52 (0.03, 1.27)	6.92 (2.35, 15.05)	9.27 (3.37, 21.44)	0.34 (-0.17, 1.25)
**Guyana**	0.618	1836.06 (1191.80, 2612.20)	1102.34 (701.78, 1629.00)	-0.40 (-0.57, -0.17)	677.33 (321.68, 1241.87)	440.91 (225.82, 790.20)	-0.35 (-0.52, -0.09)	1093.87 (640.41, 1647.23)	600.44 (335.69, 931.21)	-0.45 (-0.62, -0.21)	64.86 (38.11, 100.27)	60.99 (34.56, 96.88)	-0.06 (-0.37, 0.42)
**Haiti**	0.432	1087.24 (646.29, 1685.00)	1108.36 (623.41, 1772.19)	0.02 (-0.26, 0.44)	360.05 (167.57, 762.55)	402.05 (178.90, 829.26)	0.12 (-0.19, 0.59)	624.17 (327.33, 965.79)	601.20 (294.62, 994.68)	-0.04 (-0.32, 0.38)	103.02 (33.86, 211.43)	105.11 (38.93, 222.52)	0.02 (-0.37, 0.74)
**Honduras**	0.496	372.64 (237.46, 582.22)	628.37 (392.71, 949.52)	0.69 (0.24, 1.34)	159.99 (75.66, 312.83)	272.30 (131.02, 507.34)	0.70 (0.22, 1.41)	170.32 (95.81, 281.80)	278.36 (153.65, 464.32)	0.63 (0.15, 1.36)	42.33 (19.48, 78.99)	77.70 (41.27, 128.67)	0.84 (0.18, 1.91)
**Hungary**	0.791	483.99 (299.13, 778.84)	296.65 (177.48, 486.15)	-0.39 (-0.55, -0.17)	311.08 (154.64, 597.51)	206.23 (102.34, 397.25)	-0.34 (-0.51, -0.09)	155.25 (99.77, 231.47)	71.61 (41.84, 108.85)	-0.54 (-0.67, -0.37)	17.66 (10.84, 26.58)	18.81 (11.15, 28.09)	0.07 (-0.22, 0.50)
**Iceland**	0.869	93.85 (60.45, 148.99)	65.79 (38.22, 116.57)	-0.30 (-0.48, -0.07)	54.86 (28.58, 102.81)	37.39 (16.20, 83.76)	-0.32 (-0.52, -0.06)	29.76 (19.07, 43.66)	19.88 (11.09, 32.85)	-0.33 (-0.51, -0.10)	9.23 (5.91, 14.02)	8.51 (5.14, 12.91)	-0.08 (-0.32, 0.27)
**India**	0.566	338.17 (227.95, 492.51)	385.39 (253.82, 553.69)	0.14 (-0.07, 0.42)	111.95 (55.44, 221.05)	139.80 (69.93, 263.19)	0.25 (-0.01, 0.60)	196.54 (120.29, 288.72)	215.92 (124.15, 329.38)	0.10 (-0.13, 0.38)	29.68 (9.66, 57.18)	29.67 (14.59, 50.30)	0.00 (-0.29, 0.70)
**Indonesia**	0.660	396.35 (264.76, 578.75)	679.37 (416.04, 1057.73)	0.71 (0.31, 1.15)	131.58 (63.23, 255.74)	297.81 (131.79, 620.34)	1.26 (0.72, 1.82)	244.99 (155.73, 360.80)	358.29 (202.81, 550.94)	0.46 (0.13, 0.85)	19.79 (10.54, 34.84)	23.26 (10.59, 41.86)	0.18 (-0.22, 0.81)

**Iran (Islamic Republic of)**	0.670	301.53 (187.53, 502.47)	299.57 (177.18, 510.02)	-0.01 (-0.19, 0.23)	229.00 (124.90, 419.53)	239.93 (121.93, 452.36)	0.05 (-0.16, 0.29)	56.48 (34.90, 80.15)	48.27 (30.43, 70.70)	-0.15 (-0.32, 0.09)	16.06 (8.33, 26.70)	11.37 (7.01, 16.76)	-0.29 (-0.53, 0.20)
**Iraq**	0.671	641.36 (423.66, 998.79)	794.58 (499.16, 1199.00)	0.24 (-0.15, 0.76)	361.79 (201.84, 647.85)	509.70 (286.53, 900.92)	0.41 (-0.02, 1.01)	249.30 (151.85, 376.60)	269.72 (159.68, 391.83)	0.08 (-0.28, 0.59)	30.27 (17.10, 49.06)	15.16 (8.53, 24.24)	-0.50 (-0.70, -0.06)
**Ireland**	0.867	112.19 (69.69, 182.19)	90.88 (53.15, 162.97)	-0.19 (-0.41, 0.10)	69.32 (35.16, 130.81)	54.21 (23.18, 126.32)	-0.22 (-0.47, 0.12)	28.39 (18.08, 41.43)	23.58 (13.22, 38.73)	-0.17 (-0.40, 0.14)	14.48 (8.68, 22.11)	13.09 (8.09, 19.29)	-0.10 (-0.35, 0.31)
**Israel**	0.803	139.66 (89.98, 223.25)	96.94 (57.27, 158.99)	-0.31 (-0.47, -0.12)	68.81 (33.66, 141.84)	46.46 (19.89, 104.00)	-0.32 (-0.51, -0.08)	63.35 (39.36, 97.12)	42.39 (23.98, 66.49)	-0.33 (-0.50, -0.12)	7.50 (4.63, 11.57)	8.09 (4.90, 12.57)	0.08 (-0.24, 0.53)
**Italy**	0.801	252.34 (145.27, 433.72)	124.12 (76.22, 215.97)	-0.51 (-0.59, -0.41)	168.78 (77.04, 346.97)	65.27 (28.28, 152.77)	-0.61 (-0.69, -0.51)	71.04 (42.87, 104.38)	47.75 (28.53, 72.56)	-0.33 (-0.42, -0.20)	12.51 (8.08, 18.13)	11.09 (6.88, 16.28)	-0.11 (-0.23, 0.04)
**Jamaica**	0.684	490.87 (305.18, 794.67)	535.10 (324.75, 809.59)	0.09 (-0.19, 0.47)	231.74 (102.90, 508.89)	215.76 (98.57, 428.86)	-0.07 (-0.29, 0.28)	228.75 (132.81, 364.74)	277.10 (148.08, 434.45)	0.21 (-0.15, 0.68)	30.39 (17.29, 48.08)	42.24 (24.14, 67.46)	0.39 (-0.06, 1.04)
**Japan**	0.870	170.45 (109.53, 275.29)	70.91 (45.87, 109.32)	-0.58 (-0.64, -0.52)	95.68 (47.49, 196.12)	30.55 (14.56, 62.91)	-0.68 (-0.72, -0.64)	53.62 (34.85, 76.96)	26.89 (17.37, 39.62)	-0.50 (-0.55, -0.43)	21.15 (13.31, 30.69)	13.47 (8.38, 19.75)	-0.36 (-0.43, -0.27)
**Jordan**	0.731	638.98 (386.99, 1044.82)	353.46 (209.81, 562.75)	-0.45 (-0.59, -0.25)	450.99 (227.67, 835.02)	261.51 (131.80, 464.31)	-0.42 (-0.58, -0.21)	174.72 (103.74, 260.77)	84.78 (49.29, 128.85)	-0.51 (-0.65, -0.32)	13.28 (7.34, 21.58)	7.16 (4.18, 11.16)	-0.46 (-0.66, -0.15)
**Kazakhstan**	0.723	436.88 (269.24, 723.39)	847.28 (531.84, 1350.37)	0.94 (0.42, 1.62)	254.11 (122.87, 498.97)	481.81 (221.94, 953.58)	0.90 (0.36, 1.68)	161.51 (95.68, 262.92)	319.70 (184.95, 484.51)	0.98 (0.38, 1.87)	21.26 (12.07, 35.24)	45.77 (26.00, 71.89)	1.15 (0.41, 2.29)

Kenya	0.508	217.70 (128.90, 327.74)	285.75 (170.30, 428.12)	0.31 (0.10, 0.59)	65.59 (28.60, 144.84)	91.98 (41.70, 191.25)	0.40 (0.14, 0.72)	145.46 (78.91, 228.78)	184.63 (98.70, 289.14)	0.27 (0.05, 0.55)	6.66 (2.48, 14.85)	9.15 (3.54, 20.67)	0.37 (0.11, 0.83)
Kiribati	0.527	1943.51 (1239.85, 2803.94)	1913.74 (1263.53, 2736.49)	-0.02 (-0.32, 0.37)	367.75 (205.59, 628.99)	414.21 (225.34, 685.44)	0.13 (-0.25, 0.64)	1445.66 (890.09, 2189.76)	1344.26 (825.10, 2022.35)	-0.07 (-0.36, 0.31)	130.10 (71.91, 204.85)	155.27 (87.14, 248.05)	0.19 (-0.21, 0.76)
Kuwait	0.851	244.06 (148.29, 407.88)	227.05 (142.67, 366.69)	-0.07 (-0.29, 0.25)	176.60 (87.37, 334.98)	155.56 (82.84, 280.60)	-0.12 (-0.33, 0.19)	56.01 (34.52, 86.48)	61.82 (37.95, 93.47)	0.10 (-0.20, 0.53)	11.45 (7.22, 17.47)	9.68 (5.98, 14.51)	-0.16 (-0.40, 0.24)
Kyrgyzstan	0.596	303.00 (197.51, 450.77)	268.20 (173.30, 421.51)	-0.11 (-0.28, 0.13)	158.44 (86.78, 286.54)	143.07 (73.59, 267.25)	-0.10 (-0.29, 0.18)	128.85 (80.64, 187.96)	106.94 (65.34, 155.29)	-0.17 (-0.34, 0.08)	15.70 (8.79, 25.26)	18.19 (10.57, 27.63)	0.16 (-0.13, 0.61)
Lao People's Democratic Republic	0.490	607.52 (378.49, 943.82)	909.43 (582.99, 1331.14)	0.50 (0.07, 1.09)	189.16 (92.31, 392.23)	334.71 (151.83, 629.49)	0.77 (0.20, 1.61)	387.82 (226.73, 597.80)	540.96 (295.10, 828.10)	0.39 (-0.04, 0.97)	30.54 (12.16, 59.82)	33.77 (16.68, 64.31)	0.11 (-0.30, 1.02)
Latvia	0.820	358.70 (233.52, 556.20)	247.34 (152.21, 400.30)	-0.31 (-0.45, -0.13)	241.59 (131.05, 431.60)	183.08 (97.81, 329.48)	-0.24 (-0.39, -0.05)	99.96 (65.09, 140.22)	50.38 (31.83, 73.10)	-0.50 (-0.61, -0.35)	17.15 (10.17, 26.37)	13.88 (7.89, 21.39)	-0.19 (-0.41, 0.22)
Lebanon	0.708	179.09 (102.85, 326.36)	167.17 (81.17, 293.36)	-0.07 (-0.40, 0.37)	115.01 (51.21, 263.24)	134.57 (54.13, 258.19)	0.17 (-0.25, 0.77)	55.04 (31.61, 91.21)	28.55 (15.04, 49.55)	-0.48 (-0.66, -0.19)	9.04 (4.61, 16.18)	4.05 (1.98, 7.19)	-0.55 (-0.79, -0.06)
Lesotho	0.507	459.20 (264.19, 788.40)	886.07 (521.66, 1407.74)	0.93 (0.28, 1.99)	168.50 (61.79, 387.09)	305.78 (123.68, 669.95)	0.81 (0.25, 1.86)	278.29 (143.78, 489.02)	560.20 (285.91, 926.67)	1.01 (0.28, 2.14)	12.41 (4.69, 29.06)	20.09 (9.28, 38.25)	0.62 (-0.08, 1.86)
Liberia	0.370	341.95 (203.76, 553.77)	426.72 (252.46, 661.76)	0.25 (-0.13, 0.82)	113.95 (51.52, 249.67)	140.05 (59.96, 278.32)	0.23 (-0.14, 0.79)	220.82 (122.85, 351.24)	278.14 (145.60, 456.00)	0.26 (-0.14, 0.86)	7.18 (2.26, 18.14)	8.53 (2.43, 24.41)	0.19 (-0.30, 0.98)

**Libya**	0.709	363.06 (209.62, 618.57)	421.13 (235.43, 722.84)	0.16 (-0.17, 0.68)	215.10 (94.51, 461.53)	311.65 (154.15, 605.04)	0.45 (0.02, 1.17)	128.32 (69.12, 204.96)	95.97 (52.74, 153.84)	-0.25 (-0.49, 0.14)	19.64 (9.48, 36.21)	13.51 (7.02, 23.48)	-0.31 (-0.59, 0.24)
**Lithuania**	0.843	162.12 (105.59, 256.06)	144.26 (90.98, 237.44)	-0.11 (-0.29, 0.11)	112.39 (62.49, 203.78)	102.34 (54.95, 190.58)	-0.09 (-0.28, 0.15)	37.83 (23.32, 55.88)	30.38 (18.53, 45.03)	-0.20 (-0.37, 0.01)	11.91 (6.78, 18.37)	11.54 (6.67, 18.39)	-0.03 (-0.27, 0.29)
**Luxembourg**	0.895	161.73 (99.41, 265.46)	126.06 (76.93, 212.45)	-0.22 (-0.41, 0.03)	101.87 (52.73, 194.85)	67.80 (30.13, 147.49)	-0.33 (-0.52, -0.10)	52.34 (32.83, 75.78)	47.08 (26.86, 74.13)	-0.10 (-0.35, 0.23)	7.52 (4.67, 11.13)	11.19 (6.77, 16.82)	0.49 (0.04, 1.11)
**Madagascar**	0.396	348.48 (224.04, 529.97)	444.70 (265.21, 702.38)	0.28 (-0.10, 0.83)	87.36 (40.78, 196.08)	136.95 (60.28, 284.83)	0.57 (0.05, 1.36)	247.58 (149.32, 373.99)	291.88 (162.99, 476.36)	0.18 (-0.17, 0.70)	13.53 (4.61, 30.36)	15.87 (5.19, 38.91)	0.17 (-0.24, 0.77)
**Malawi**	0.384	367.21 (210.88, 614.60)	449.56 (268.33, 690.24)	0.22 (-0.13, 0.81)	134.71 (53.80, 309.75)	177.32 (78.38, 372.43)	0.32 (-0.08, 0.99)	222.71 (117.99, 387.49)	259.05 (133.58, 421.60)	0.16 (-0.20, 0.76)	9.79 (2.98, 26.10)	13.19 (3.62, 36.07)	0.35 (-0.17, 1.11)
**Malaysia**	0.737	548.99 (381.42, 765.89)	415.51 (264.80, 641.38)	-0.24 (-0.45, 0.07)	168.96 (91.08, 295.86)	171.90 (82.83, 328.78)	0.02 (-0.30, 0.47)	347.56 (227.75, 490.54)	224.94 (131.46, 345.49)	-0.35 (-0.53, -0.09)	32.47 (19.16, 49.64)	18.67 (10.81, 30.01)	-0.43 (-0.64, -0.04)
**Maldives**	0.562	403.21 (257.48, 601.33)	250.13 (152.23, 411.19)	-0.38 (-0.55, -0.14)	128.45 (62.44, 270.02)	113.92 (50.37, 248.40)	-0.11 (-0.41, 0.29)	247.96 (156.69, 376.89)	122.27 (71.14, 195.27)	-0.51 (-0.65, -0.31)	26.79 (10.43, 49.33)	13.93 (7.85, 22.67)	-0.48 (-0.71, 0.26)
**Mali**	0.263	243.96 (155.64, 365.62)	369.03 (213.24, 575.94)	0.51 (0.06, 1.15)	68.20 (34.62, 133.33)	111.44 (50.57, 243.23)	0.63 (0.11, 1.33)	169.98 (104.02, 257.61)	250.64 (135.01, 414.54)	0.47 (0.03, 1.17)	5.78 (1.97, 12.67)	6.95 (2.45, 16.64)	0.20 (-0.21, 0.89)
**Malta**	0.801	324.19 (184.81, 536.84)	134.57 (79.16, 227.90)	-0.58 (-0.68, -0.47)	197.69 (83.38, 402.19)	79.88 (35.00, 173.53)	-0.60 (-0.69, -0.47)	113.17 (62.33, 179.36)	45.69 (26.07, 69.84)	-0.60 (-0.69, -0.47)	13.33 (8.12, 20.26)	9.00 (5.54, 13.18)	-0.32 (-0.49, -0.12)

**Marshall Islands**	0.544	1612.10 (1020.79, 2306.75)	1482.49 (916.05, 2272.16)	-0.08 (-0.32, 0.26)	309.43 (156.97, 578.11)	288.91 (162.06, 514.95)	-0.07 (-0.32, 0.29)	1145.49 (688.37, 1725.01)	1049.41 (597.55, 1708.50)	-0.08 (-0.35, 0.28)	157.19 (66.66, 292.10)	144.17 (64.25, 262.41)	-0.08 (-0.39, 0.43)
**Mauritania**	0.496	222.09 (148.89, 333.10)	183.95 (108.86, 299.91)	-0.17 (-0.43, 0.20)	75.46 (40.52, 149.01)	70.30 (32.13, 159.82)	-0.07 (-0.38, 0.36)	141.95 (88.94, 213.32)	110.45 (60.70, 180.26)	-0.22 (-0.47, 0.14)	4.68 (1.98, 8.97)	3.21 (1.36, 6.38)	-0.32 (-0.59, 0.13)
**Mauritius**	0.705	724.39 (470.21, 1098.78)	350.17 (234.21, 506.09)	-0.52 (-0.66, -0.34)	375.50 (185.24, 711.68)	153.59 (82.10, 271.09)	-0.59 (-0.71, -0.42)	317.56 (200.10, 471.92)	162.48 (100.57, 236.28)	-0.49 (-0.64, -0.30)	31.33 (19.18, 46.73)	34.11 (20.02, 50.81)	0.09 (-0.23, 0.53)
**Mexico**	0.649	302.62 (205.94, 453.66)	198.37 (135.52, 289.02)	-0.34 (-0.44, -0.22)	159.31 (83.48, 294.90)	88.78 (45.12, 167.36)	-0.44 (-0.53, -0.35)	121.36 (77.31, 167.62)	80.88 (49.68, 115.71)	-0.33 (-0.44, -0.20)	21.94 (14.16, 31.15)	28.71 (17.73, 41.35)	0.31 (-0.09, 0.64)
**Micronesia (Federated States of)**	0.580	889.64 (550.16, 1364.80)	1382.36 (826.80, 2076.55)	0.55 (0.01, 1.30)	181.53 (88.18, 367.87)	296.89 (148.46, 536.87)	0.64 (0.04, 1.61)	623.79 (374.70, 967.58)	980.10 (559.43, 1548.59)	0.57 (0.00, 1.33)	84.32 (34.52, 158.19)	105.38 (46.87, 190.19)	0.25 (-0.23, 1.08)
**Monaco**	0.902	127.55 (74.67, 212.65)	96.66 (53.83, 175.19)	-0.24 (-0.48, 0.19)	81.66 (40.76, 158.11)	62.78 (28.02, 134.66)	-0.23 (-0.48, 0.23)	34.38 (20.57, 51.62)	26.11 (14.96, 42.77)	-0.24 (-0.51, 0.23)	11.51 (6.39, 18.82)	7.77 (4.40, 12.36)	-0.32 (-0.57, 0.11)
**Mongolia**	0.606	246.95 (145.40, 400.88)	366.88 (226.54, 561.30)	0.49 (0.07, 1.05)	20.30 (10.54, 43.24)	40.72 (21.56, 77.13)	1.01 (0.35, 1.90)	210.48 (122.86, 346.78)	301.70 (180.16, 473.06)	0.43 (0.02, 1.02)	16.17 (8.21, 29.21)	24.46 (14.08, 39.89)	0.51 (-0.08, 1.61)
**Montenegro**	0.791	581.41 (365.09, 891.31)	940.26 (581.96, 1422.71)	0.62 (0.19, 1.19)	87.56 (43.11, 178.22)	191.40 (86.81, 387.17)	1.19 (0.57, 2.14)	484.51 (282.27, 772.92)	733.95 (419.55, 1201.37)	0.51 (0.12, 1.05)	9.34 (5.78, 14.44)	14.91 (8.79, 23.12)	0.60 (0.11, 1.35)
**Morocco**	0.548	330.99 (211.84, 554.77)	640.89 (378.26, 1048.24)	0.94 (0.35, 1.70)	172.86 (84.49, 348.41)	465.46 (230.37, 880.24)	1.69 (0.88, 2.77)	133.54 (75.76, 206.97)	151.56 (85.20, 233.89)	0.13 (-0.23, 0.69)	24.58 (9.57, 45.82)	23.87 (12.07, 39.58)	-0.03 (-0.41, 0.90)

**Mozambique**	0.307	348.21 (216.62, 535.73)	654.97 (392.88, 1033.51)	0.88 (0.29, 1.79)	115.88 (50.82, 256.02)	248.65 (107.65, 502.64)	1.15 (0.39, 2.37)	219.72 (125.63, 350.92)	389.62 (199.45, 649.67)	0.77 (0.21, 1.67)	12.60 (3.22, 34.63)	16.70 (5.50, 43.06)	0.33 (-0.16, 1.64)
**Myanmar**	0.521	842.58 (535.66, 1273.63)	1019.68 (672.72, 1517.66)	0.21 (-0.11, 0.71)	252.89 (124.07, 515.69)	405.67 (182.60, 796.25)	0.60 (0.11, 1.31)	544.73 (318.16, 811.45)	578.33 (341.99, 900.19)	0.06 (-0.25, 0.50)	44.96 (20.13, 83.72)	35.67 (19.93, 58.92)	-0.21 (-0.52, 0.40)
**Namibia**	0.612	556.94 (340.40, 883.81)	563.02 (331.79, 890.85)	0.01 (-0.27, 0.42)	237.72 (108.88, 505.32)	269.98 (116.90, 556.61)	0.14 (-0.20, 0.67)	306.90 (172.91, 498.15)	282.69 (148.18, 446.16)	-0.08 (-0.38, 0.34)	12.32 (6.29, 22.22)	10.36 (5.39, 19.46)	-0.16 (-0.49, 0.42)
**Nauru**	0.618	1052.59 (679.74, 1559.22)	1530.66 (972.77, 2263.10)	0.45 (0.12, 0.92)	293.72 (144.18, 544.87)	435.26 (231.58, 743.94)	0.48 (0.09, 1.07)	696.35 (425.18, 1130.81)	1010.87 (592.16, 1578.07)	0.45 (0.08, 0.93)	62.52 (33.19, 100.48)	84.53 (43.00, 139.88)	0.35 (-0.08, 1.00)
**Nepal**	0.422	247.12 (156.65, 372.45)	307.04 (188.18, 488.44)	0.24 (-0.16, 0.79)	74.19 (33.31, 152.66)	118.18 (51.85, 241.62)	0.59 (0.04, 1.33)	144.51 (80.09, 224.67)	161.02 (91.62, 267.56)	0.11 (-0.25, 0.65)	28.42 (8.64, 60.70)	27.83 (10.59, 56.33)	-0.02 (-0.37, 0.69)
**Netherlands**	0.883	131.37 (74.90, 238.37)	85.74 (49.17, 154.98)	-0.35 (-0.51, -0.08)	83.92 (37.92, 188.84)	51.30 (22.24, 117.03)	-0.39 (-0.56, -0.14)	35.95 (21.20, 58.22)	25.64 (14.71, 42.84)	-0.29 (-0.47, -0.01)	11.50 (7.03, 17.40)	8.81 (5.36, 13.30)	-0.23 (-0.42, 0.06)
**New Zealand**	0.840	102.69 (63.75, 168.78)	74.19 (43.79, 142.41)	-0.28 (-0.44, -0.04)	61.66 (30.59, 120.59)	42.93 (18.47, 105.16)	-0.30 (-0.50, 0.00)	26.88 (16.96, 39.37)	19.88 (11.68, 31.45)	-0.26 (-0.43, -0.01)	14.16 (8.32, 21.23)	11.39 (6.93, 16.91)	-0.20 (-0.36, 0.05)
**Nicaragua**	0.517	338.49 (214.45, 507.21)	291.00 (180.70, 441.82)	-0.14 (-0.34, 0.15)	146.26 (66.17, 291.38)	138.33 (64.95, 277.87)	-0.05 (-0.28, 0.29)	169.06 (95.87, 253.97)	128.58 (73.37, 194.89)	-0.24 (-0.43, 0.05)	23.17 (13.32, 34.86)	24.08 (13.76, 36.65)	0.04 (-0.31, 0.55)
**Niger**	0.162	120.88 (76.59, 187.98)	362.61 (186.56, 601.21)	2.00 (0.94, 3.39)	36.11 (16.77, 78.27)	110.82 (44.40, 261.08)	2.07 (1.01, 3.44)	82.10 (49.05, 123.28)	245.10 (114.33, 426.94)	1.99 (0.84, 3.39)	2.68 (0.84, 6.21)	6.69 (1.87, 18.31)	1.50 (0.38, 3.18)

Nigeria	0.515	262.70 (151.15, 431.27)	254.88 (145.71, 413.80)	-0.03 (-0.34, 0.38)	87.08 (38.23, 210.38)	98.61 (42.38, 220.67)	0.13 (-0.25, 0.64)	170.81 (91.00, 279.03)	152.57 (79.83, 256.45)	-0.11 (-0.39, 0.29)	4.81 (1.70, 11.39)	3.70 (1.33, 7.83)	-0.23 (-0.50, 0.21)
Niue	0.711	989.78 (627.01, 1396.24)	801.40 (512.72, 1161.88)	-0.19 (-0.42, 0.12)	270.33 (134.42, 499.16)	223.58 (118.70, 393.05)	-0.17 (-0.39, 0.16)	648.94 (380.79, 956.33)	524.57 (316.63, 803.83)	-0.19 (-0.43, 0.16)	70.51 (39.84, 110.68)	53.25 (26.39, 94.01)	-0.24 (-0.57, 0.24)
North Macedonia	0.744	892.09 (542.12, 1458.18)	1293.56 (753.55, 2211.97)	0.45 (0.01, 0.99)	573.64 (274.38, 1097.65)	878.28 (407.85, 1745.36)	0.53 (0.07, 1.15)	279.77 (164.54, 416.82)	347.23 (196.95, 551.92)	0.24 (-0.14, 0.74)	38.68 (22.75, 58.45)	68.05 (38.71, 105.29)	0.76 (0.09, 1.69)
Northern Mariana Islands	0.771	666.50 (433.34, 985.23)	611.43 (409.13, 860.37)	-0.08 (-0.30, 0.21)	180.66 (94.93, 370.48)	155.91 (84.72, 286.01)	-0.14 (-0.35, 0.21)	435.17 (274.75, 655.69)	408.59 (248.30, 594.62)	-0.06 (-0.31, 0.26)	50.67 (29.84, 81.03)	46.93 (28.29, 72.82)	-0.07 (-0.37, 0.35)
Norway	0.913	199.63 (118.33, 346.69)	97.35 (57.68, 174.63)	-0.51 (-0.58, -0.43)	135.41 (62.68, 280.17)	56.88 (25.01, 127.15)	-0.58 (-0.65, -0.50)	44.48 (27.42, 67.19)	27.84 (15.93, 43.77)	-0.37 (-0.48, -0.24)	19.74 (12.28, 28.89)	12.63 (8.05, 18.42)	-0.36 (-0.49, -0.18)
Oman	0.783	475.41 (296.18, 768.82)	528.72 (327.20, 867.50)	0.11 (-0.25, 0.71)	253.50 (129.83, 480.38)	370.84 (183.04, 705.73)	0.46 (-0.02, 1.22)	197.02 (114.52, 313.74)	140.26 (82.70, 209.37)	-0.29 (-0.51, 0.09)	24.89 (12.62, 42.64)	17.62 (7.85, 31.58)	-0.29 (-0.63, 0.39)
Pakistan	0.449	345.95 (222.70, 510.56)	552.30 (358.88, 824.99)	0.60 (0.20, 1.21)	110.36 (54.65, 222.11)	215.72 (105.35, 403.18)	0.95 (0.40, 1.88)	199.86 (117.58, 289.66)	293.05 (180.23, 440.93)	0.47 (0.08, 1.07)	35.73 (12.85, 66.35)	43.53 (21.16, 74.38)	0.22 (-0.15, 0.95)
Palau	0.738	781.48 (497.60, 1177.85)	894.19 (574.93, 1274.89)	0.14 (-0.22, 0.68)	259.24 (132.99, 496.10)	308.78 (171.10, 527.11)	0.19 (-0.20, 0.85)	479.84 (283.95, 750.75)	536.39 (312.78, 821.58)	0.12 (-0.26, 0.68)	42.40 (22.76, 69.55)	49.02 (27.37, 80.06)	0.16 (-0.31, 0.93)
Palestine	0.588	709.27 (391.97, 1143.80)	658.13 (388.55, 1123.89)	-0.07 (-0.34, 0.32)	445.30 (199.20, 867.11)	492.10 (241.85, 942.89)	0.11 (-0.20, 0.58)	247.89 (132.50, 403.80)	154.16 (88.79, 235.96)	-0.38 (-0.57, -0.08)	16.09 (8.17, 27.85)	11.87 (7.06, 18.11)	-0.26 (-0.53, 0.27)

**Panama**	0.686	319.16 (195.88, 501.69)	221.80 (134.66, 343.03)	-0.31 (-0.49, -0.02)	143.78 (63.57, 292.94)	92.83 (40.99, 192.61)	-0.35 (-0.54, -0.10)	150.26 (86.98, 242.19)	99.26 (54.16, 156.22)	-0.34 (-0.54, -0.02)	25.13 (14.94, 40.26)	29.70 (17.13, 45.62)	0.18 (-0.21, 0.82)
**Papua New Guinea**	0.394	613.07 (351.11, 998.89)	996.84 (592.00, 1521.42)	0.63 (0.17, 1.34)	93.98 (39.29, 193.39)	159.81 (72.89, 303.39)	0.70 (0.20, 1.51)	465.85 (261.23, 790.38)	756.67 (412.96, 1181.60)	0.62 (0.14, 1.37)	53.24 (18.46, 114.26)	80.35 (32.50, 161.45)	0.51 (0.03, 1.41)
**Paraguay**	0.638	259.30 (164.33, 408.92)	296.05 (170.38, 487.99)	0.14 (-0.23, 0.72)	110.28 (49.44, 241.05)	145.75 (63.03, 305.74)	0.32 (-0.13, 1.06)	127.39 (76.96, 191.06)	121.59 (62.81, 196.33)	-0.05 (-0.37, 0.47)	21.62 (12.77, 33.08)	28.70 (15.48, 49.31)	0.33 (-0.18, 1.19)
**Peru**	0.648	118.16 (77.90, 175.38)	74.48 (42.03, 129.12)	-0.37 (-0.59, -0.02)	43.13 (22.40, 85.44)	33.29 (14.66, 73.81)	-0.23 (-0.51, 0.23)	60.69 (37.86, 90.13)	30.84 (16.53, 52.75)	-0.49 (-0.67, -0.22)	14.34 (8.05, 23.38)	10.34 (5.46, 17.97)	-0.28 (-0.56, 0.19)
**Philippines**	0.623	225.45 (143.13, 355.62)	400.60 (262.37, 602.52)	0.78 (0.27, 1.34)	95.46 (45.86, 205.94)	145.30 (72.98, 265.97)	0.52 (0.04, 1.08)	122.94 (73.00, 186.64)	239.61 (137.63, 366.06)	0.95 (0.34, 1.57)	7.05 (3.94, 12.22)	15.69 (9.26, 23.72)	1.23 (0.49, 2.26)
**Poland**	0.802	368.62 (248.40, 559.24)	267.25 (162.24, 423.07)	-0.28 (-0.42, -0.10)	220.53 (121.44, 394.51)	169.26 (84.00, 320.17)	-0.23 (-0.40, -0.05)	121.40 (80.92, 169.65)	76.78 (46.77, 113.94)	-0.37 (-0.51, -0.21)	26.69 (16.91, 39.19)	21.21 (12.97, 30.91)	-0.21 (-0.39, 0.05)
**Portugal**	0.743	553.86 (334.03, 920.74)	224.01 (139.61, 371.68)	-0.60 (-0.70, -0.46)	350.08 (164.13, 726.74)	124.51 (57.47, 266.12)	-0.64 (-0.74, -0.51)	185.43 (115.23, 283.19)	82.09 (50.72, 122.93)	-0.56 (-0.66, -0.43)	18.34 (11.84, 27.17)	17.41 (10.93, 25.62)	-0.05 (-0.26, 0.25)
**Puerto Rico**	0.814	197.38 (130.07, 302.66)	111.04 (71.58, 167.09)	-0.44 (-0.59, -0.21)	93.88 (45.84, 183.78)	42.17 (21.17, 78.37)	-0.55 (-0.67, -0.37)	85.32 (55.75, 121.71)	53.53 (31.35, 81.30)	-0.37 (-0.56, -0.10)	18.19 (11.34, 26.40)	15.34 (9.10, 23.20)	-0.16 (-0.42, 0.25)
**Qatar**	0.830	425.21 (263.35, 693.05)	213.33 (127.10, 365.58)	-0.50 (-0.66, -0.28)	236.29 (112.11, 476.14)	129.17 (63.65, 266.22)	-0.45 (-0.63, -0.19)	146.60 (84.37, 231.42)	63.14 (38.53, 103.27)	-0.57 (-0.72, -0.35)	42.32 (21.45, 77.04)	21.02 (11.99, 33.64)	-0.50 (-0.73, -0.09)

**Republic of Korea**	0.878	510.84 (350.88, 726.17)	143.55 (85.31, 245.16)	-0.72 (-0.80, -0.54)	214.89 (114.07, 388.99)	81.49 (35.34, 179.79)	-0.62 (-0.74, -0.34)	255.33 (160.12, 359.96)	47.55 (28.09, 74.07)	-0.81 (-0.86, -0.71)	40.61 (23.63, 61.47)	14.51 (8.74, 22.65)	-0.64 (-0.74, -0.46)
**Republic of Moldova**	0.696	367.33 (247.54, 535.95)	290.76 (187.36, 444.48)	-0.21 (-0.38, 0.01)	153.32 (79.25, 287.32)	166.10 (87.61, 298.62)	0.08 (-0.18, 0.45)	203.52 (132.06, 295.92)	115.16 (70.54, 170.14)	-0.43 (-0.56, -0.28)	10.50 (6.33, 16.33)	9.50 (5.66, 13.96)	-0.09 (-0.33, 0.31)
**Romania**	0.760	403.91 (262.65, 616.83)	331.28 (211.23, 530.52)	-0.18 (-0.35, 0.05)	235.25 (126.20, 437.37)	195.42 (99.81, 361.92)	-0.17 (-0.37, 0.10)	145.07 (95.25, 214.91)	108.14 (67.39, 161.81)	-0.25 (-0.43, 0.00)	23.59 (14.38, 37.35)	27.72 (17.45, 41.48)	0.18 (-0.19, 0.71)
**Russian Federation**	0.805	526.30 (344.62, 802.69)	417.56 (270.40, 638.95)	-0.21 (-0.36, -0.02)	370.72 (214.58, 628.64)	277.02 (156.84, 474.32)	-0.25 (-0.40, -0.07)	130.05 (86.52, 180.86)	108.48 (69.26, 155.89)	-0.17 (-0.35, 0.07)	25.53 (15.49, 37.02)	32.06 (19.53, 47.16)	0.26 (0.01, 0.58)
**Rwanda**	0.429	495.90 (252.86, 835.84)	398.74 (218.46, 634.24)	-0.20 (-0.46, 0.26)	122.05 (43.57, 287.90)	123.20 (47.63, 285.06)	0.01 (-0.35, 0.58)	358.92 (172.65, 640.37)	262.84 (120.63, 452.78)	-0.27 (-0.53, 0.18)	14.93 (3.95, 39.11)	12.69 (3.68, 32.20)	-0.15 (-0.51, 0.72)
**Saint Kitts and Nevis**	0.746	1340.28 (862.72, 1998.94)	727.36 (470.01, 1076.08)	-0.46 (-0.56, -0.32)	632.75 (302.30, 1277.35)	349.58 (175.59, 652.22)	-0.45 (-0.55, -0.30)	658.15 (391.24, 994.50)	339.72 (201.25, 508.69)	-0.48 (-0.61, -0.30)	49.38 (29.83, 74.88)	38.05 (22.07, 60.24)	-0.23 (-0.47, 0.14)
**Saint Lucia**	0.670	927.02 (608.17, 1361.45)	491.39 (325.01, 736.02)	-0.47 (-0.57, -0.34)	438.57 (214.01, 833.53)	219.13 (111.13, 406.37)	-0.50 (-0.60, -0.35)	438.42 (260.33, 662.00)	236.85 (140.29, 358.45)	-0.46 (-0.58, -0.30)	50.03 (30.13, 77.29)	35.41 (21.05, 52.73)	-0.29 (-0.49, -0.02)
**Saint Vincent and the Grenadines**	0.627	629.09 (383.50, 996.32)	524.10 (352.52, 758.84)	-0.17 (-0.35, 0.07)	403.38 (199.73, 766.01)	229.10 (121.06, 428.88)	-0.43 (-0.54, -0.31)	188.91 (112.56, 282.30)	266.69 (158.59, 386.98)	0.41 (0.11, 0.81)	36.80 (21.98, 56.92)	28.31 (16.92, 42.46)	-0.23 (-0.45, 0.04)
**Samoa**	0.641	936.32 (572.21, 1372.92)	971.34 (623.65, 1414.80)	0.04 (-0.22, 0.43)	227.40 (104.61, 450.48)	239.12 (122.32, 453.49)	0.05 (-0.21, 0.47)	640.96 (364.46, 982.30)	661.16 (390.99, 1001.23)	0.03 (-0.24, 0.47)	67.96 (35.91, 123.68)	71.06 (38.08, 120.23)	0.05 (-0.34, 0.66)

**San Marino**	0.884	92.47 (55.55, 160.04)	114.00 (56.52, 212.78)	0.23 (-0.24, 0.90)	58.79 (29.10, 120.50)	73.83 (27.95, 169.18)	0.26 (-0.23, 0.93)	27.02 (16.41, 41.31)	32.09 (16.05, 59.41)	0.19 (-0.31, 0.88)	6.67 (3.96, 9.98)	8.07 (4.07, 13.79)	0.21 (-0.30, 1.00)
**Sao Tome and Principe**	0.502	301.93 (185.00, 475.23)	471.78 (286.36, 755.29)	0.56 (0.14, 1.16)	110.76 (50.52, 249.65)	185.60 (82.92, 364.42)	0.68 (0.19, 1.42)	185.32 (109.47, 291.21)	279.33 (150.86, 468.09)	0.51 (0.06, 1.12)	5.84 (2.18, 12.37)	6.86 (2.91, 14.22)	0.17 (-0.25, 0.90)
**Saudi Arabia**	0.805	611.40 (373.30, 976.82)	546.66 (346.41, 802.46)	-0.11 (-0.40, 0.34)	337.16 (172.84, 644.47)	333.12 (179.81, 566.29)	-0.01 (-0.36, 0.51)	258.15 (148.57, 390.49)	203.81 (123.28, 304.19)	-0.21 (-0.49, 0.22)	16.10 (8.53, 27.31)	9.74 (5.35, 15.34)	-0.39 (-0.70, 0.15)
**Senegal**	0.389	341.50 (222.72, 516.26)	434.49 (265.87, 664.67)	0.27 (-0.07, 0.77)	118.72 (58.00, 232.66)	148.79 (70.27, 281.91)	0.25 (-0.08, 0.73)	216.08 (132.23, 326.28)	278.04 (159.51, 443.25)	0.29 (-0.11, 0.85)	6.70 (2.45, 14.41)	7.67 (2.70, 16.75)	0.14 (-0.25, 0.73)
**Serbia**	0.767	754.06 (450.02, 1277.80)	770.59 (446.94, 1346.57)	0.02 (-0.30, 0.45)	487.33 (228.40, 987.94)	566.99 (267.23, 1113.86)	0.16 (-0.22, 0.67)	223.57 (136.28, 333.60)	158.48 (92.03, 253.60)	-0.29 (-0.52, 0.00)	43.17 (25.50, 66.17)	45.12 (26.05, 70.58)	0.05 (-0.32, 0.58)
**Seychelles**	0.724	433.47 (289.04, 668.33)	390.81 (259.82, 588.17)	-0.10 (-0.29, 0.18)	188.42 (94.27, 387.17)	177.94 (92.53, 336.96)	-0.06 (-0.30, 0.32)	227.57 (144.03, 332.31)	200.15 (124.59, 299.14)	-0.12 (-0.33, 0.18)	17.48 (7.96, 28.68)	12.72 (4.84, 23.45)	-0.27 (-0.52, 0.09)
**Sierra Leone**	0.347	165.25 (99.60, 257.69)	238.18 (140.81, 388.14)	0.44 (0.04, 1.02)	55.56 (28.12, 107.95)	80.54 (36.87, 172.09)	0.45 (0.01, 1.10)	106.42 (59.70, 165.77)	153.45 (82.87, 250.69)	0.44 (0.02, 1.06)	3.28 (1.10, 7.84)	4.19 (1.43, 9.93)	0.28 (-0.18, 1.05)
**Singapore**	0.861	320.43 (216.04, 468.92)	71.74 (46.48, 116.10)	-0.78 (-0.83, -0.71)	172.53 (92.09, 302.61)	33.57 (16.70, 71.87)	-0.81 (-0.86, -0.73)	127.84 (85.68, 177.02)	30.39 (18.93, 45.40)	-0.76 (-0.81, -0.69)	20.07 (12.87, 27.81)	7.79 (4.76, 11.58)	-0.61 (-0.69, -0.47)
**Slovakia**	0.812	305.79 (204.45, 459.79)	204.10 (120.89, 350.90)	-0.33 (-0.53, -0.09)	173.71 (98.67, 310.49)	138.60 (69.73, 277.30)	-0.20 (-0.44, 0.08)	112.48 (72.36, 157.06)	54.42 (31.69, 84.96)	-0.52 (-0.66, -0.32)	19.60 (12.26, 28.05)	11.09 (6.37, 18.00)	-0.43 (-0.60, -0.17)

**Slovenia**	0.840	319.71 (182.63, 580.34)	120.61 (67.82, 207.02)	-0.62 (-0.76, -0.44)	228.24 (109.96, 480.36)	74.73 (33.94, 157.87)	-0.67 (-0.79, -0.51)	77.66 (46.14, 118.84)	36.70 (19.85, 60.17)	-0.53 (-0.70, -0.27)	13.81 (7.89, 21.79)	9.17 (5.31, 14.49)	-0.34 (-0.58, 0.03)
**Solomon Islands**	0.407	1076.39 (701.81, 1555.19)	2191.48 (1406.61, 3168.02)	1.04 (0.44, 1.81)	169.33 (92.39, 297.48)	397.78 (207.96, 692.05)	1.35 (0.58, 2.31)	821.10 (507.06, 1197.01)	1648.57 (959.10, 2560.52)	1.01 (0.40, 1.81)	85.97 (38.09, 160.73)	145.13 (69.57, 272.02)	0.69 (0.11, 1.61)
**Somalia**	0.081	385.61 (230.47, 610.64)	465.71 (258.64, 748.70)	0.21 (-0.18, 0.89)	93.25 (41.21, 207.88)	137.50 (56.73, 305.94)	0.47 (-0.06, 1.35)	276.41 (152.94, 445.09)	306.10 (148.09, 525.84)	0.11 (-0.28, 0.72)	15.94 (3.72, 47.23)	22.12 (4.36, 76.04)	0.39 (-0.26, 1.27)
**South Africa**	0.678	277.40 (179.09, 435.98)	385.31 (250.68, 581.03)	0.39 (0.16, 0.71)	124.02 (59.74, 269.87)	204.89 (100.84, 396.49)	0.65 (0.36, 1.10)	146.63 (92.87, 208.52)	172.46 (102.10, 248.42)	0.18 (-0.10, 0.48)	6.75 (4.08, 10.10)	7.96 (4.92, 11.70)	0.18 (-0.08, 0.49)
**South Sudan**	0.363	277.95 (165.55, 452.89)	283.29 (156.91, 462.36)	0.02 (-0.30, 0.56)	80.47 (33.59, 181.02)	98.89 (39.67, 220.49)	0.23 (-0.20, 1.04)	189.55 (105.39, 304.10)	175.97 (89.25, 290.78)	-0.07 (-0.39, 0.41)	7.93 (2.80, 18.75)	8.44 (2.57, 21.33)	0.06 (-0.35, 0.77)
**Spain**	0.767	300.73 (173.20, 554.71)	111.48 (69.52, 183.51)	-0.63 (-0.70, -0.52)	195.33 (85.95, 440.82)	55.00 (24.61, 123.73)	-0.72 (-0.78, -0.64)	94.32 (55.97, 145.32)	44.78 (26.49, 68.98)	-0.53 (-0.62, -0.40)	11.07 (6.88, 16.44)	11.70 (7.29, 17.88)	0.06 (-0.18, 0.37)
**Sri Lanka**	0.690	456.80 (273.97, 806.86)	345.10 (202.86, 594.26)	-0.24 (-0.45, 0.03)	260.28 (108.79, 599.67)	201.73 (91.10, 418.33)	-0.22 (-0.45, 0.11)	147.35 (81.17, 238.44)	108.87 (59.63, 175.21)	-0.26 (-0.51, 0.09)	49.17 (24.00, 86.18)	34.51 (18.44, 58.89)	-0.30 (-0.61, 0.29)
**Sudan**	0.515	504.67 (290.60, 866.35)	707.21 (403.41, 1205.50)	0.40 (-0.01, 1.05)	229.49 (106.36, 516.48)	483.31 (231.62, 960.96)	1.11 (0.46, 2.17)	238.87 (130.82, 374.57)	196.33 (104.34, 320.74)	-0.18 (-0.43, 0.20)	36.32 (11.02, 77.49)	27.57 (11.86, 52.51)	-0.24 (-0.54, 0.55)
**Suriname**	0.636	599.64 (401.44, 888.19)	681.49 (459.89, 983.16)	0.14 (-0.13, 0.52)	231.45 (114.40, 455.86)	241.72 (125.12, 450.57)	0.04 (-0.20, 0.43)	336.23 (205.91, 503.93)	398.91 (239.27, 598.34)	0.19 (-0.11, 0.64)	31.96 (19.56, 47.10)	40.86 (24.80, 62.66)	0.28 (-0.09, 0.82)

Sweden	0.872	119.89 (71.79, 207.52)	96.30 (54.65, 177.85)	-0.20 (-0.38, 0.05)	76.29 (37.15, 159.01)	58.10 (25.06, 136.89)	-0.24 (-0.44, 0.06)	32.08 (20.25, 47.86)	28.66 (16.41, 46.64)	-0.11 (-0.31, 0.16)	11.52 (7.45, 16.78)	9.54 (5.95, 14.19)	-0.17 (-0.37, 0.15)
Switzerland	0.929	128.06 (76.59, 236.23)	58.53 (34.41, 112.43)	-0.54 (-0.66, -0.39)	80.68 (39.07, 177.57)	35.48 (15.56, 87.02)	-0.56 (-0.69, -0.39)	36.23 (22.21, 56.88)	15.56 (8.81, 26.26)	-0.57 (-0.69, -0.42)	11.15 (6.95, 16.96)	7.50 (4.62, 11.45)	-0.33 (-0.47, -0.10)
Syrian Arab Republic	0.619	461.40 (290.58, 740.05)	522.74 (313.57, 846.68)	0.13 (-0.24, 0.71)	223.65 (114.03, 450.92)	292.32 (140.03, 590.15)	0.31 (-0.15, 0.95)	221.35 (131.84, 340.99)	216.95 (123.94, 350.34)	-0.02 (-0.36, 0.50)	16.40 (8.07, 28.82)	13.47 (7.72, 22.59)	-0.18 (-0.52, 0.55)
Taiwan (Province of China)	0.868	346.80 (240.96, 493.47)	141.25 (87.90, 217.33)	-0.59 (-0.70, -0.46)	120.88 (65.58, 220.29)	52.65 (24.93, 107.59)	-0.56 (-0.69, -0.38)	214.63 (140.65, 299.37)	75.68 (45.58, 117.18)	-0.65 (-0.74, -0.53)	11.28 (7.38, 15.96)	12.93 (7.60, 20.11)	0.15 (-0.18, 0.63)
Tajikistan	0.539	233.63 (153.75, 327.35)	931.39 (546.63, 1420.78)	2.99 (1.81, 4.49)	29.65 (15.41, 54.02)	176.67 (79.72, 371.16)	4.96 (2.95, 7.79)	199.35 (124.41, 287.13)	737.59 (380.86, 1212.31)	2.70 (1.55, 4.14)	4.63 (2.12, 8.19)	17.14 (7.98, 29.27)	2.70 (1.38, 4.88)
Thailand	0.687	254.25 (167.98, 364.38)	197.33 (115.65, 321.49)	-0.22 (-0.47, 0.11)	71.44 (38.12, 138.45)	75.28 (35.50, 155.52)	0.05 (-0.33, 0.56)	151.38 (96.72, 220.11)	94.48 (54.64, 153.41)	-0.38 (-0.58, -0.09)	31.43 (17.63, 49.52)	27.56 (15.48, 44.14)	-0.12 (-0.46, 0.43)
Timor-Leste	0.514	284.83 (169.96, 461.37)	787.09 (453.80, 1221.62)	1.76 (0.93, 2.94)	95.87 (43.76, 207.57)	308.49 (126.16, 619.70)	2.22 (1.13, 3.90)	172.89 (94.54, 273.15)	451.18 (229.92, 728.22)	1.61 (0.69, 2.77)	16.06 (6.96, 33.19)	27.41 (12.30, 55.05)	0.71 (0.06, 1.99)
Togo	0.417	204.09 (134.97, 302.39)	238.35 (154.40, 358.08)	0.17 (-0.10, 0.53)	68.33 (35.98, 129.01)	78.50 (40.44, 143.73)	0.15 (-0.13, 0.52)	131.76 (80.91, 194.96)	155.67 (92.72, 238.28)	0.18 (-0.10, 0.57)	4.00 (1.54, 9.21)	4.18 (1.58, 10.12)	0.05 (-0.30, 0.51)
Tokelau	0.626	771.68 (483.29, 1159.17)	770.97 (480.92, 1125.85)	0.00 (-0.28, 0.43)	177.98 (84.88, 361.14)	210.07 (112.64, 381.41)	0.18 (-0.17, 0.70)	520.12 (312.55, 813.12)	502.06 (290.76, 750.80)	-0.03 (-0.31, 0.46)	73.58 (36.20, 131.27)	58.84 (32.59, 94.20)	-0.20 (-0.53, 0.42)

**Tonga**	0.636	409.61 (274.46, 594.26)	444.62 (291.89, 644.90)	0.09 (-0.22, 0.54)	116.41 (59.72, 220.41)	143.27 (74.12, 258.61)	0.23 (-0.11, 0.77)	259.83 (156.73, 387.55)	269.39 (159.77, 396.11)	0.04 (-0.26, 0.48)	33.38 (18.12, 57.68)	31.95 (17.35, 56.36)	-0.04 (-0.34, 0.42)
**Trinidad and Tobago**	0.757	833.41 (558.28, 1222.82)	408.23 (252.81, 638.37)	-0.51 (-0.65, -0.35)	413.80 (213.54, 753.38)	202.40 (103.49, 377.48)	-0.51 (-0.64, -0.34)	367.84 (220.44, 537.89)	165.51 (94.67, 260.28)	-0.55 (-0.68, -0.37)	51.77 (30.53, 77.70)	40.32 (22.02, 63.01)	-0.22 (-0.50, 0.16)
**Tunisia**	0.672	370.60 (218.97, 642.41)	410.06 (241.01, 703.95)	0.11 (-0.26, 0.64)	215.20 (100.10, 475.85)	307.48 (151.46, 579.12)	0.43 (-0.04, 1.11)	131.43 (73.18, 205.78)	88.44 (48.76, 140.04)	-0.33 (-0.55, 0.06)	23.97 (10.77, 45.68)	14.14 (7.95, 23.93)	-0.41 (-0.67, 0.18)
**Turkey**	0.748	178.26 (113.53, 298.41)	220.26 (127.84, 364.00)	0.24 (-0.20, 0.78)	86.44 (43.14, 173.43)	128.56 (59.33, 263.38)	0.49 (-0.07, 1.19)	74.56 (46.16, 116.37)	76.12 (44.71, 117.71)	0.02 (-0.35, 0.52)	17.25 (8.27, 30.73)	15.58 (9.34, 23.81)	-0.10 (-0.49, 0.83)
**Turkmenistan**	0.670	274.14 (186.81, 395.78)	651.84 (372.63, 1085.32)	1.38 (0.60, 2.38)	119.80 (62.16, 215.55)	326.12 (150.83, 661.29)	1.72 (0.73, 3.11)	138.18 (85.46, 201.14)	282.84 (150.19, 484.95)	1.05 (0.34, 2.00)	16.16 (8.89, 26.84)	42.88 (22.50, 71.06)	1.65 (0.75, 3.02)
**Tuvalu**	0.589	985.69 (629.45, 1467.79)	1115.56 (722.13, 1637.47)	0.13 (-0.21, 0.66)	205.79 (104.84, 390.24)	275.13 (147.96, 493.10)	0.34 (-0.09, 1.03)	687.10 (414.62, 1069.73)	751.97 (458.42, 1141.66)	0.09 (-0.26, 0.63)	92.80 (39.09, 180.17)	88.45 (45.43, 159.28)	-0.05 (-0.42, 0.70)
**Uganda**	0.404	325.73 (193.28, 517.15)	397.45 (235.52, 615.02)	0.22 (-0.12, 0.71)	98.52 (40.78, 217.66)	132.53 (57.17, 268.47)	0.35 (-0.08, 0.94)	216.14 (119.59, 354.42)	252.71 (133.25, 418.04)	0.17 (-0.18, 0.71)	11.08 (2.92, 29.26)	12.20 (3.89, 30.34)	0.10 (-0.24, 0.93)
**Ukraine**	0.736	331.15 (209.58, 523.71)	309.42 (201.61, 471.73)	-0.07 (-0.25, 0.19)	244.11 (135.56, 426.21)	204.45 (115.84, 344.99)	-0.16 (-0.33, 0.07)	70.27 (43.97, 103.14)	85.85 (54.53, 125.91)	0.22 (-0.07, 0.70)	16.78 (9.95, 27.13)	19.13 (11.65, 29.47)	0.14 (-0.18, 0.66)
**United Arab Emirates**	0.880	960.53 (567.71, 1543.63)	486.37 (287.41, 776.21)	-0.49 (-0.65, -0.29)	590.91 (283.56, 1096.26)	352.70 (189.21, 611.45)	-0.40 (-0.59, -0.11)	335.98 (187.60, 524.83)	120.54 (66.88, 206.94)	-0.64 (-0.77, -0.44)	33.64 (14.93, 61.77)	13.13 (5.12, 24.73)	-0.61 (-0.77, -0.30)

United Kingdom	0.847	220.82 (129.74, 382.99)	141.29 (90.86, 236.16)	-0.36 (-0.46, -0.23)	150.12 (69.39, 316.52)	72.52 (33.91, 160.40)	-0.52 (-0.60, -0.40)	49.09 (30.35, 71.60)	45.80 (28.88, 67.10)	-0.07 (-0.19, 0.09)	21.60 (13.64, 31.47)	22.97 (14.79, 33.24)	0.06 (-0.09, 0.30)
United Republic of Tanzania	0.423	179.94 (112.26, 283.54)	307.02 (170.17, 505.63)	0.71 (0.17, 1.51)	52.58 (23.42, 115.26)	131.62 (53.75, 300.27)	1.50 (0.54, 2.84)	121.15 (69.99, 183.97)	166.94 (82.83, 291.66)	0.38 (-0.08, 1.06)	6.20 (2.07, 14.54)	8.45 (2.72, 20.07)	0.36 (-0.13, 1.42)
United States of America	0.859	152.50 (101.38, 231.36)	142.94 (100.19, 204.69)	-0.06 (-0.19, 0.09)	82.02 (43.98, 154.43)	58.41 (29.52, 114.26)	-0.29 (-0.40, -0.14)	50.46 (33.37, 69.68)	60.59 (39.93, 81.82)	0.20 (0.06, 0.37)	20.03 (12.98, 27.96)	23.94 (15.75, 32.95)	0.20 (0.03, 0.41)
United States Virgin Islands	0.799	244.70 (156.68, 373.40)	324.45 (218.06, 473.53)	0.33 (-0.01, 0.78)	106.81 (51.70, 217.12)	138.34 (67.28, 262.08)	0.30 (-0.05, 0.79)	111.57 (68.58, 166.88)	155.71 (90.74, 232.24)	0.40 (0.00, 0.93)	26.33 (15.93, 40.78)	30.40 (18.06, 45.67)	0.15 (-0.23, 0.69)
Uruguay	0.697	140.35 (87.02, 219.51)	169.76 (106.20, 267.19)	0.21 (-0.08, 0.60)	81.42 (44.22, 148.93)	92.90 (44.32, 184.92)	0.14 (-0.18, 0.53)	40.11 (25.10, 60.29)	52.71 (31.29, 80.62)	0.31 (-0.08, 0.90)	18.82 (10.26, 30.42)	24.15 (15.41, 35.16)	0.28 (-0.14, 0.85)
Uzbekistan	0.631	226.00 (153.26, 326.46)	954.38 (610.96, 1442.68)	3.22 (2.16, 4.73)	103.29 (57.30, 181.75)	457.34 (216.34, 886.55)	3.43 (1.91, 5.31)	116.05 (72.10, 170.49)	472.74 (262.92, 740.26)	3.07 (1.88, 4.88)	6.65 (3.67, 10.68)	24.30 (14.03, 37.29)	2.65 (1.36, 5.17)
Vanuatu	0.485	811.00 (495.97, 1238.54)	1315.53 (816.30, 1945.73)	0.62 (0.17, 1.36)	192.31 (100.69, 364.25)	324.07 (170.11, 567.12)	0.69 (0.21, 1.48)	558.55 (325.36, 876.59)	902.76 (523.27, 1402.75)	0.62 (0.15, 1.39)	60.14 (26.78, 113.62)	88.70 (41.22, 163.42)	0.47 (-0.01, 1.28)
Venezuela (Bolivarian Republic of)	0.607	357.64 (229.31, 527.86)	329.01 (191.98, 505.89)	-0.08 (-0.32, 0.26)	138.38 (59.69, 290.48)	116.03 (52.44, 235.05)	-0.16 (-0.39, 0.17)	189.40 (108.96, 293.79)	177.96 (98.22, 284.54)	-0.06 (-0.33, 0.35)	29.86 (17.73, 45.96)	35.02 (19.21, 55.61)	0.17 (-0.23, 0.73)
Viet Nam	0.617	423.84 (263.63, 692.40)	760.24 (462.12, 1227.27)	0.79 (0.24, 1.63)	161.25 (75.35, 355.64)	357.77 (154.67, 735.99)	1.22 (0.45, 2.46)	242.07 (139.66, 387.63)	381.69 (213.90, 628.12)	0.58 (0.06, 1.34)	20.52 (10.23, 39.65)	20.79 (10.23, 38.37)	0.01 (-0.46, 0.94)

**Yemen**	0.412	370.71 (227.96, 573.86)	572.83 (314.35, 978.08)	0.55 (0.03, 1.29)	153.57 (78.04, 290.02)	375.95 (168.56, 763.09)	1.45 (0.68, 2.56)	188.09 (104.38, 293.67)	167.99 (83.45, 274.24)	-0.11 (-0.41, 0.38)	29.05 (9.25, 61.25)	28.89 (11.14, 60.24)	-0.01 (-0.36, 0.70)
**Zambia**	0.505	334.50 (201.43, 544.57)	552.64 (330.01, 849.60)	0.65 (0.14, 1.43)	95.28 (39.28, 235.92)	182.25 (75.98, 400.08)	0.91 (0.24, 2.02)	229.11 (128.20, 365.41)	355.42 (183.60, 585.75)	0.55 (0.04, 1.29)	10.12 (3.59, 23.40)	14.96 (6.08, 32.14)	0.48 (-0.14, 1.73)
**Zimbabwe**	0.476	299.67 (177.99, 523.88)	504.81 (304.02, 837.79)	0.68 (0.21, 1.37)	169.88 (76.10, 386.16)	279.76 (125.54, 560.36)	0.65 (0.17, 1.41)	115.56 (71.27, 189.89)	200.18 (108.29, 319.88)	0.73 (0.20, 1.49)	14.24 (7.45, 30.47)	24.87 (12.02, 52.52)	0.75 (0.20, 1.55)

Abbreviations: ASR, age-stanrdardized rate; SDI, sociodemographic index; DALYs, disability-adjusted life-years; YLDs, years lived with disability; YLLs, years of life lost; HFPG, high fasting plasma glucose; UI, uncertainty interval.

Table S3. 21 GBD regions and their country composition.

GBD regions	Countries
**Central Asia**	Armenia, Azerbaijan, Georgia, Kazakhstan, Kyrgyzstan, Mongolia, Tajikistan, Turkmenistan, Uzbekistan
**Central Europe**	Albania, Bosnia and Herzegovina, Bulgaria, Croatia, Czech Republic, Hungary, Montenegro, North Macedonia, Poland, Romania, Serbia, Slovakia, Slovenia
**Eastern Europe**	Belarus, Estonia, Latvia, Lithuania, Moldova, Russia, Ukraine
**Australasia**	Australia, New Zealand
**High-income Asia Pacific**	Brunei, Japan, South Korea, Singapore
**High-income North America**	Canada, Greenland, USA
**Southern Latin America**	Argentina, Chile, Uruguay
**Western Europe**	Andorra, Austria, Belgium, Sweden, Switzerland, UK Austria, Belgium, Cyprus, Denmark, Finland, France, Germany, Greece, Iceland, Ireland, Israel, Italy, Luxembourg, Malta, Monaco, Netherlands, Norway, Portugal, San Marino, Spain
**Andean Latin America**	Bolivia, Ecuador, Peru
**Caribbean**	Antigua and Barbuda, Grenada, Guyana, Haiti, Jamaica, Puerto Rico, Saint Kitts and Nevis, , The Bahamas, Barbados, Belize, Bermuda, Cuba, Dominica, Dominican Republic, Saint Lucia, Saint Vincent and the Grenadines, Suriname, Trinidad and Tobago, Virgin Islands
**Central Latin America**	Colombia, Costa Rica, Venezuela, Panama, Honduras, El Salvador, Guatemala, Mexico, Nicaragua
**Tropical Latin America**	Brazil, Paraguay
**North Africa and Middle East**	Afghanistan, Algeria, Bahrain, Egypt, Iran, Iraq, Jordan, Kuwait, Lebanon, Libya, Morocco, Oman, Palestine, Qatar, Saudi Arabia, Sudan, Syria, Tunisia, Turkey, United Arab Emirates, Yemen
**South Asia**	Bangladesh, Bhutan, India, Nepal, Pakistan
**East Asia**	China, North Korea, Taiwan (province of China)

**Oceania**	American Samoa, Northern Mariana Islands, Palau, Papua New Guinea, Samoa, Solomon Islands, Tokelau, Tonga, Tuvalu, VanuatuCook Islands, Fiji, Guam, Kiribati, Marshall Islands, Federated States of Micronesia, Nauru, Niue
**Southeast Asia**	Cambodia, Thailand, Timor-Leste, Vietnam, Sri Lanka, Indonesia, Laos, Malaysia, Maldives, Mauritius, Myanmar, Philippines, Seychelles
**Central sub-Saharan Africa**	Angola, Central African Republic, Congo (Brazzaville), DR Congo, Equatorial Guinea, Gabon
**Eastern sub-Saharan Africa**	Burundi, Comoros, Djibouti, Eritrea, Ethiopia, Kenya, Madagascar, Malawi, Mozambique, Rwanda, Somalia, South Sudan, Uganda, Tanzania, Zambia
**Southern sub-Saharan Africa**	Botswana, eSwatini, Lesotho, Namibia, South Africa, Zimbabwe
**Western sub-Saharan Africa**	Benin, Burkina Faso, Cape Verde, Cameroon, Chad, Côte d'Ivoire, The Gambia, Ghana, Guinea-Bissau, Guinea, Liberia, Mali, Mauritania, Niger, Nigeria, São Tomé and PrÍncipe, Senegal, Sierra Leone, Togo
